# Sustainable Development in Surgery: The Health, Poverty, and Equity Impacts of Charitable Surgery in Uganda

**DOI:** 10.1371/journal.pone.0168867

**Published:** 2016-12-30

**Authors:** Mark G. Shrime, Serufusa Sekidde, Allison Linden, Jessica L. Cohen, Milton C. Weinstein, Joshua A. Salomon

**Affiliations:** 1 Program in Global Surgery and Social Change, Department of Global Health and Social Medicine, Harvard Medical School, Boston, MA, United States of America; 2 Office of Global Surgery, Massachusetts Eye and Ear Infirmary, Boston, MA, United States of America; 3 Aspen Global Health and Development, Aspen Institute, Aspen, CO, United States of America; 4 Division of Pediatric Surgery, Children’s Hospital Los Angeles, Los Angeles, CA, United States of America; 5 Department of Global Health and Population, Harvard T.H. Chan School of Public Health, Boston, MA, United States of America; 6 Center for Health Decision Science, Harvard T.H. Chan School of Public Health, Boston, MA, United States of America; 7 Department of Health Policy and Management, Harvard T.H. Chan School of Public Health, Boston, MA, United States of America; Johns Hopkins University Bloomberg School of Public Health, UNITED STATES

## Abstract

**Background:**

The recently adopted Sustainable Development Goals call for the end of poverty and the equitable provision of healthcare. These goals are often at odds, however: health seeking can lead to catastrophic spending, an outcome for which cancer patients and the poor in resource-limited settings are at particularly high risk. How various health policies affect the additional aims of financial wellbeing and equity is poorly understood. This paper evaluates the health, financial, and equity impacts of governmental and charitable policies for surgical oncology in a resource-limited setting.

**Methods:**

Three charitable platforms for surgical oncology delivery in Uganda were compared to six governmental policies aimed at improving healthcare access. An extended cost-effectiveness analysis using an agent-based simulation model examined the numbers of lives saved, catastrophic expenditure averted, impoverishment averted, costs, and the distribution of benefits across the wealth spectrum.

**Findings:**

Of the nine policies and platforms evaluated, two were able to provide simultaneous health and financial benefits efficiently and equitably: mobile surgical units and governmental policies that simultaneously address surgical scaleup, the cost of surgery, and the cost of transportation. Policies that only remove user fees are dominated, as is the commonly employed short-term “surgical mission trip”. These results are robust to scenario and sensitivity analyses.

**Interpretation:**

The most common platforms for increasing access to surgical care appear unable to provide health and financial risk protection equitably. On the other hand, mobile surgical units, to date an underutilized delivery platform, are able to deliver surgical oncology in a manner that meets sustainable development goals by improving health, financial solvency, and equity. These platforms compare favorably with policies that holistically address surgical delivery and should be considered as countries strengthen health systems.

## Introduction

Health and financial hardship are inextricably linked. Poverty can be both a barrier to healthcare [1] and its result, especially in low- and middle-income countries (LMICs), where catastrophic spending on health is common [1–4]. For cancer patients, this financial burden also drives poor health outcomes. In Nigeria, for example, one-fifth of children presenting with a rapidly lethal tumor did not get treatment because of cost [5].

Unfortunately, improving health and alleviating impoverishment do not always align. Policies that alleviate medically-related financial burden have shown only minimal impacts on health [1, 6]. On the other hand, increasing the number of healthcare providers may, by inducing demand for services with significant costs, counterintuitively increase the risk of financial catastrophe. [1]

In Uganda, an East African country of 37·5 million people, [7] cancer causes an estimated 11,000 deaths and a loss of 350,000 disability adjusted life years annually. [8] As of 2010, only 15% of districts met the Ministry of Health’s minimum standards for staffing, and only 11% of the population lived within 5km of any hospital. [9] In addition, despite an official abolition of medical user fees in 2001, nearly half of healthcare financing comes from out-of-pocket expenditure, [9] putting patients at risk for financial hardship.

Although the treatment of cancer itself is complex and multimodal, surgery is involved in 60% of oncologic disease. [8] In LMICs, however, the absence of multimodal therapy often consigns cancer patients to a complete lack of treatment, even when surgery is available. [10] Compounding this issue is the fact that the surgical capacity of many first-level hospitals in LMICs tends to be devoted to the treatment of low-burden surgical conditions, leaving patients with more serious surgical conditions like cancer without treatment. [11] In Uganda, only 221 surgeons or anaesthesiologists serve the entire 236,000-km^2^ country,[12] and medical trainees tend to avoid careers in surgery because of perceived excessive workloads, the risk of contracting HIV, low financial returns, and a poor learning environment.[13]

Simultaneously, a large and rapidly growing charitable sector has set itself up as a parallel, fragmented surgical delivery system in many LMICs.[14, 15] These charities, some of which perform cancer surgery, operate under three basic delivery models: short-term surgical trips, self-contained mobile surgical units, and free-standing specialized surgical hospitals.[16] Despite a stated preference by patients in at least some LMICs for government health services,[17] up to 20% of healthcare and 55% of surgery may be provided by the charitable sector.[18–20]

The ability, then, to address equitably the overlapping issues of health and impoverishment intrinsic to medical care will require a holistic evaluation of an entire health system, accounting for the various barriers to care mentioned above—distance, cost, poverty—and the multiple governmental and non-governmental platforms involved. To date, however, no such evaluation has been undertaken. Agent-based simulation modeling, discussed below, is well suited to encompass the various barriers, platforms, and outcomes of interest in a health system, and will be used here.

The goal of this paper, then, is to compare charitable surgical platforms with governmental policies for surgical oncology in Uganda. The hypothesis is that, when the domains of health, impoverishment, and equity are simultaneously considered, charitable platforms will perform as well as, if not better than, many government policies.

## Methods

This paper follows a simulated cohort of patients through 50 years in the Ugandan public healthcare system. Details on model construction are given in S1 Appendix; a brief summary follows.

### Platforms examined

Nine policies and platforms for surgical oncology delivery in Uganda, chosen for their relative frequency of use in LMICs,[21–25] are examined. Six governmental policies focus on public sector hospitals:

1 UPF: Universal public financing, which makes surgery free at the point of care, but does not pay for non-medical costs necessary to seek care
2 TS: Task shifting of surgery to non-surgeon providers,[26] which increases the supply of surgical providers but does not address costs
3 UPFTS—in which non-surgeon providers are trained in surgery, and the medical costs of care are free to the user
4-6 Combinations of each of the above with vouchers to pay for non-medical costs such as transportation (UPF^V^, TS^V^, and UPFTS^V^)[27]

On the non-governmental organization (NGO) side, three charitable platforms are examined:[16]

7 Two-week surgical “mission trips” (2W)
8 Self-contained mobile surgical units (MS)
9 Free-standing cancer hospitals (CH)

Data from private-sector hospitals in Uganda are unavailable.

### Model design

Agent-based models have been proposed as instructive representations of human-human interaction [28–30] because they allow the modeler to place patients and providers within physical space and to model their interpersonal networks explicitly.[31] By design, these models are stochastic, facilitating the incorporation of uncertainty (around, for example, estimates of cost and mortality). They also relax the assumption, made in prior policy analyses,[1, 32–34] that individuals are independent of each other—a feature necessary for the measurement of impoverishment (as defined below).

A synthetic, open-cohort population of 10,000 individuals was constructed. At instantiation, the cohort mirrored the demographic and socioeconomic profile of the Uganda 2011 Demographic and Health Survey.[35] The population was stochastically geopositioned onto a map of Uganda, using GPS data,[36] and was connected into family-level and distance-based networks.[28, 29] Population growth parameters were modeled using published background mortality and fertility rates.[37] Individual income was drawn from a gamma distribution, parameterized for Uganda.[7, 38] The national poverty line was defined as the income below which 19·5% of the population lived, to match Uganda’s poverty headcount.[7] Model parameterization is given in **Table A** in S1 Appendix.

The seven most common cancers in Uganda were evaluated in the model (**Table B**, S1 Appendix). Modeled incidence was based on published country-specific 2012 data.[39, 40] When an individual became sick, he or she chose whether or not, and where, to seek care, based on a Ugandan multivariate nested logit model of patient and healthcare provider characteristics.[30, 41, 42] Untreated individuals faced mortality from untreated cancer,[43–45] while treated individuals faced mortality and complication rates conditional on the type of provider chosen.

The network of 53 public Ugandan hospitals was also geolocalized.[12] Hospital quality metrics were derived from previously published data on surgical delivery in Uganda.[12] The NGO platforms were modeled as follows: Because short-term mission trips operate within pre-existing structures,[16] 2W was modeled as semi-annual, two-week-long trips to one of the regional referral hospitals. MS was modeled after CinterAndes, a truck-based surgical delivery system in Ecuador.[46] CH was modeled as an NGO-run cancer hospital in Mbarara, where a cancer hospital has been proposed. Reported costs for the reference NGOs were divided by the reported numbers of cases performed by each NGO to determine a cost per case. The full code of the model is made available in S2 Appendix.

The model cycled daily for 50 years. One hundred runs of the model were performed, each randomly drawing a different parameter set from the parameter distributions shown in **Table A,** S2 Appendix. Accounting for births and deaths, this produced on average 2·7 trillion person-years of data for comparison.

### Outcome definitions, model validation, and sensitivity analyses

The Ugandan status quo was modeled first; each policy’s results represent incremental outcomes against this status quo. Health benefit was measured as the number of cancer deaths averted by an intervention. Catastrophic expenditure was defined as any expenditure that was more than 10% of income prior to the cancer-related illness.[47–49]

Because catastrophic expenditure only captures the financial burden incurred by people actually getting care, a second metric—“impoverishment”—was defined to capture the financial burden of a lack of access to care. People were said to be impoverished if a health expense pushed an individual below the national poverty line [7], *or* if the head of a household succumbed to cancer—in this case, the remaining family members were counted. This construction was tested in sensitivity analyses.

To measure the distribution of benefits, an “equity index” was constructed for each of the three outcomes (deaths, catastrophic expenditure, and impoverishment), analogous to a Gini coefficient.[50] Details on its construction are given in S1 Appendix.

In the base case, the model takes societal perspective, with outcomes reported as yearly averages per 100,000 individuals. Efficiency was defined according to the principles of cost-effectiveness analysis and data envelopment analysis,[51] whereby programs that achieve inferior outcomes at equal or higher cost than other program options are said to be inefficient, or “dominated”.

Validation was performed against the following known metrics, which were not directly input into the model: predicted population density, predicted 2050 national population, overall cancer incidence, and cancer incidence:mortality ratio. Utilization was also validated by location and by wealth quintile. Details of the validation are given in S1 Appendix.

In addition to the evaluation of heterogeneity and parameter uncertainty, the following sensitivity and scenario analyses were performed:

- Results presented from the perspective of the Ministry of Health,
- Results presented as discounted aggregate streams, instead of yearly averages,
- An increase in the cost of MS by twelve fold, to match the United States IRS Form 990-reported expenses of other mobile NGOs, and
- A more conservative definition of the “impoverishment” metric, in which impoverishment was counted only if the loss of income from a family member who succumbed to cancer pushed the entire family below a synthetic familial poverty line.

The model was constructed in Java, using the AnyLogic modeling platform (The AnyLogic Company, St. Petersburg, Russia); data analysis was performed in R v3·0 (www.r-project.org). Because publicly available data were used, ethics approval was not necessary.

## Results

### Validation and summary results

**Fig 1:**
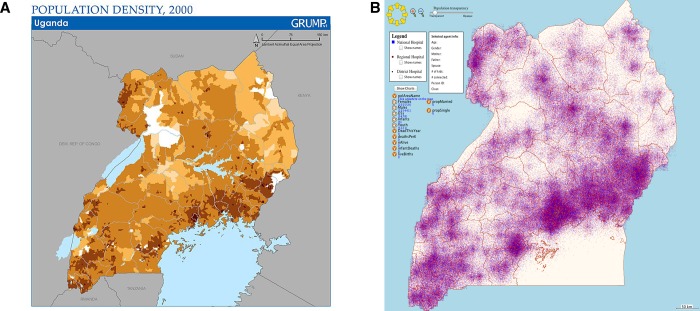
Population density validation. Actual[52] vs. modeled population density in Uganda. In the model, each red and blue dot represents one person.

The model proved to be representative of the population of Uganda. An example validation result is given in **Fig 1** with further validation in S1 Appendix. Health and financial risk protection results are given in **Table 1**. Incremental cost-effectiveness ratios for each outcome (deaths, catastrophic expenditure, and impoverishment) are given in **Table 2**. Acceptability curves, displaying the effects of uncertainty across 100 randomly chosen parameter sets, are given in S1 Appendix.

**Table 1:** Incremental outcomes, compared against the status quo.

		Platforms and Policies								
		UPF	TS	UPFTS	UPF^V^	TS^V^	UPFTS^V^	2W	MS	CH
System cost/100,000 people		$3,320*	$301	$3,670*	$24,470*	$13,701*	$25,009*	$40,438*	$7,047*	$54,431*
Cancer Deaths Averted / 100,000 people	Poorest	4·9	-3·7	4·7	51·2*	30·0	51·8*	2·4	68·2*	35·8
	Poor	5·7	7·1	14·0	29·7	27·9	34·5*	2·6	42·2*	27·9
	Middle	1·5	4·6	11·2	26·5	17·3	30·1*	-0·1	36·9*	30·3
	Rich	2·9	6·9	8·7	23·7	13·4	27·1	1·9	35·2*	32·2*
	Richest	-0·1	1·1	5·1	22·6	4·6	24·5	0·6	31·6*	25·2
	***Overall***	***3·0***	***3·2***	***8·7***	30·7*	18·7*	33·6*	***1·5***	42·8*	30·3*
Catastrophic Expenditure Averted / 100,000 people	Poorest	-22·7	-29·3	-57·7	147·7*	-95·5	147·7*	7·5	87·6	59·3
	Poor	-13·7	-40·7	-53·9	194·9*	-31·7	194·9*	10·1	102·9	77·8
	Middle	-2·0	-45·3	-41·5	226·0*	58·1	226·0*	1·6	106·5*	89·2
	Rich	16·9	-46·5	-4·5	260·8*	151·6*	260·8*	10·8	112·2	104·8
	Richest	42·7	-12·0	41·9	263·8*	202·7*	263·8*	6·1	87·9	75·1
	***Overall***	***4·2***	***-34·8***	***-23·1***	218·6*	***57·1***	218·6*	***7·2***	99·4*	81·2*
Impoverishment Averted / 100,000 people	Poorest	-12·6	-76·3	-79·0	516·7*	-19·0	518·6*	12·8	321·2*	214·9*
	Poor	10·2	13·9	24·3	34·7	53·0	43·5	-0·3	63·0	43·1
	Middle	3·3	6·1	19·0	35·5	33·2	36·6	0·4	57·1	47·4
	Rich	5·5	16·2	16·6	21·4	22·8	28·3	1·5	53·7	45·0
	Richest	-2·9	-0·7	10·2	10·7	-0·2	9·0	-2·5	38·2	24·0
	***Overall***	***0·7***	***-8·1***	***-1·8***	123·8*	***18·0***	127·2*	***2·4***	106·6*	74·9*
Treatment probability, given a cancer diagnosis	Poorest	5·4	4·9	11·9	39·1*	27·6*	46·5*	1·1	24·2*	9·9
	Poor	5·0	6·6	12·0	32·4*	24·8*	40·4*	0·9	20·0*	12·0
	Middle	3·6	7·4	11·6	27·7*	22·2*	34·5*	1·2	17·2*	14·1
	Rich	3·0	6·6	10·1	22·5*	17·5*	28·3*	0·3	14·1	13·4
	Richest	1·9	5·9	7·9	19·7*	12·9	25·5*	1·1	13·5	12·2
	***Overall***	***3·8***	***6·3***	***10·7***	28·3*	21·0*	35·0*	***0·9***	17·8*	12·3*
Incidence: Mortality ratio^a^	Poorest	1·03	0·98	1·04	1·35*	1·18*	1·35*	1·02	1·57*	1·24*
	Poor	1·06	1·06	1·12	1·29*	1·25*	1·39*	1·01	1·46*	1·27*
	Middle	1·03	1·06	1·10	1·31*	1·16	1·37*	1·02	1·45*	1·36*
	Rich	1·02	1·06	1·08	1·28*	1·12	1·33*	1·02	1·43*	1·35*
	Richest	1·00	1·02	1·05	1·30*	1·05	1·34*	1·03	1·44*	1·34*
	***Overall***	***1·03***	***1·04***	***1·08***	1·30*	1·15*	1·35*	***1·02***	1·46*	1·32*

Incremental results over the status quo, per 100,000 in the population, on average, per year; positive numbers indicate improvement over the status quo.

^a^Relative improvement: numbers >1·0 indicate an improvement over the status quo. UPF = universal public financing, TS = task shifting, V = vouchers, 2W = two-week mission trip, MS = mobile surgical platform, CH = cancer hospital.

* = *p* < 0·05.

**Table 2:** Incremental cost-effectiveness ratios.

		Incremental cost-effectiveness ratios		
Sector	Policy	Deaths	Catastrophic expenditure	Impoverishment
Governmental policies	TS	$88·65	*—*	*—*
	UPF^V^	—	$111·88	*—*
	UPFTS	$631·41	*—*	*—*
	UPFTS^V^	$662·35	*—*	$197·06
Non-governmental organizations	MS	$154·78	$66·34	$62·02
All policies and platforms	TS	$88·65	*—*	*—*
	MS	$160·29	$66·34	$62·02
	UPF^V^	*—*	$150·48	*—*
	UPFTS^V^	$6022·33	*—*	$897·31

Incremental cost-effectiveness ratios (ICERs) for governmental policies, non-governmental platforms, and both, in dollars per case averted. A long dash signifies that the policy is dominated by other policies for that outcome—that is, that other policies deliver more benefit at a lower cost. Strategies dominated in all three columns are not shown. TS = task shifting, UPF = universal public finance, V = vouchers, MS = mobile surgical unit, CH = cancer hospital.

### Efficiency frontiers

Efficiency frontiers plot benefit against cost. On these graphs, policies to the upper-left provide the most benefit for the lowest cost. (A more detailed explanation is given in S1 Appendix).

If a decision-maker is willing to consider both governmental and NGO policies, only MS is efficient at averting deaths while simultaneously preventing catastrophic expenditure and impoverishment. The incremental cost per death averted is $160 (or, approximately 25% of Uganda’s GDP/capita), while the cost per case of catastrophic expenditure or impoverishment averted is each approximately $60.

**Fig 2:**
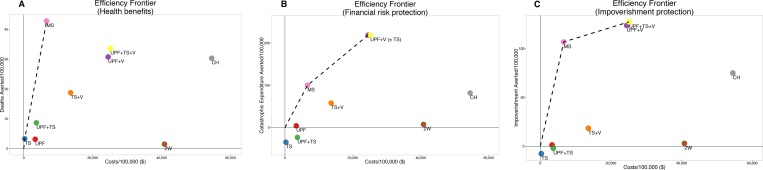
Efficiency frontiers for all policies and platforms. Incremental cost-effectiveness ratios are given in Table 2. A) Deaths averted per 100,000 people. B) Catastrophic expenditure averted per 100,000 people. C) Impoverishment averted per 100,000 people. UPF = universal public finance. TS = task shifting. V = vouchers. MS = mobile surgical unit. CH = cancer hospital. 2W = two-week surgical mission. The interpretation of efficiency frontiers is explained in detail in S1 Appendix.

Other policies can deliver isolated benefits efficiently: TS and UPFTS^V^ efficiently prevent cancer deaths; UPF^V^ efficiently prevents catastrophic expenditure; and UPFTS^V^ efficiently prevents impoverishment (**Fig 2**).

Intrasectoral comparisons (ie, NGOs alone or government policies alone) are given in **Table 2** and in **Figs I** and **J** in S1 Appendix.

### Standardized outcomes

**Fig 3:**
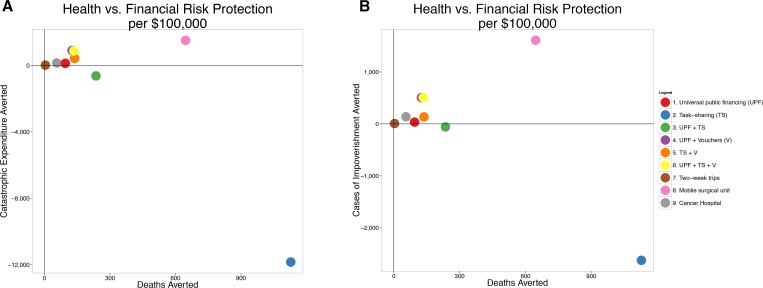
Health and financial risk protection per $100,000 spent. Policies closest to the upper right are most efficient. A) Deaths averted vs. catastrophic expenditure averted. B) Deaths averted vs. impoverishment averted. For both financial risk protection outcomes, the mobile surgical unit is dominant. Note that negative cases of catastrophic risk protection and impoverishment averted imply cases *created* by the respective policies. UPF = universal public finance. TS = task shifting. V = vouchers. MS = mobile surgical unit. CH = cancer hospital. 2W = two-week surgical mission. The interpretation of these standardized outcomes panels is explained in detail in S1 Appendix.

To aid in decision-making across health and financial domains, **Fig 3** standardizes outcomes against the cost of each policy.[53] Policies toward the right are the most efficient at delivering health per dollar, with those in the upper right delivering the most health *and* financial benefits per dollar. In this formulation, both TS and MS are efficient.

### Equity

The equitable distribution of health and impoverishment benefits for each of the nine policies and platforms is summarized by the equity index (**Table 3**), in which a more positive value indicates a more pro-poor outcome distribution. All NGO platforms, as well as UPF^V^ and UPFTS^V^, demonstrate a pro-poor distribution. The remaining policies tend to provide benefit preferentially to the more wealthy.

**Table 3:** Equity.

		Equity index			
		Deaths	Catastrophic expenditure	Impoverishment	Average
Governmental policies	UPF	0·332	-0·479	-0·088	-0·079
	TS	-0·109	-0·196	-0·180	-0·162
	UPFTS	0·041	-0·575	-0·177	-0·237
	UPF^V^	0·164	-0·109	0·663	0·239
	TS^V^	0·280	-0·409	-0·016	-0·048
	UPFTS^V^	0·148	-0·109	0·650	0·230
Non-governmental organizations	2W	0·203	0·023	0·472	0·233
	MS	0·150	-0·008	0·432	0·191
	CH	0·044	-0·058	0·406	0·131

Equity index of benefits, measuring how pro-poor an intervention is. The more positive the number, the higher the concentration of the benefits accruing to the poorest patients. CH = cancer hospital, MS = mobile surgical unit, TS = task shifting, UPF = universal public finance, V = vouchers, 2W = two-week surgical “mission trips”.

### Sensitivity analysis on the cost of mobile surgery

**Fig 4:**
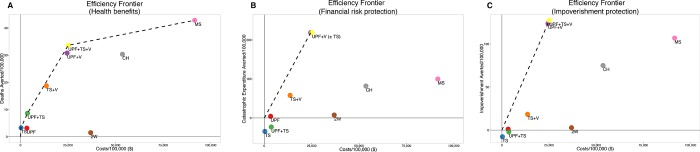
Efficiency frontiers when the cost of the mobile surgical unit is increased. A) Deaths averted per 100,000 people. B) Catastrophic expenditure averted per 100,000 people. C) Impoverishment averted per 100,000 people. The platform falls off the efficiency frontier for the financial risk protective outcomes.

Mobile surgical platforms vary immensely in cost. To evaluate the sensitivity of the above results to this variability, we increased the unit cost per surgery twelvefold for MS, to match the expenses reported to the United States IRS by other mobile surgical NGOs. With this large increase, MS remained an efficient strategy for saving lives, but became dominated as a strategy to prevent catastrophic expenditure or impoverishment (**Fig 4**). Other sensitivity analyses are shown in S1 Appendix.

## Discussion

The Sustainable Development Goals, adopted in September 2015, propose an end to all poverty (Goal 1) while simultaneously ensuring health and wellbeing, equitably for all (Goal 3).[54] These goals are intertwined: to create sustainable development, health systems must do more than simply improve health; they must provide financial risk protection and do so equitably.[55] However, most policy evaluations to date have focused only on medical incomes, and most policymakers have traditionally looked to the public sector alone to accomplish these aims.[56]

Our results indicate that the exclusion of NGO platforms in health system evaluations may be unwarranted.

In the setting of surgical oncology in Uganda, the only governmental policies that were able provide health benefits, protection against impoverishment, and equity were those that simultaneously addressed the lack of surgical supply, the out-of-pocket medical costs of a procedure, *and* the often ignored non-medical costs of transportation. These tend to be very expensive policies. On the other hand, mobile surgical units—of the sorts already in place in other countries[46]—can meet sustainable development goals efficiently and equitably.

The dominance of these mobile platforms seems initially counterintuitive—they are, after all, uncommon modalities for healthcare delivery in general, and surgery in specific. However, they address at least two of what have been called the “three delays” in healthcare.[57] By decreasing the distance from the patient to the provider, they shorten the “second delay” of getting to care. This decreased distance, combined with the charitable model of low-to-nil out-of-pocket payments,[46] also shortens the “first delay” of deciding to seek care in the first place. (The “third delay”—getting care once the provider is reached—is not explicitly examined in this paper.)

Notably, our results do not support the use of very common policies and platforms, such as the isolated removal of user fees. Earlier evidence has been mixed for the health and financial benefits of user fee removal,[58–60] despite its relative ease of implementation. Our results indicate that it is dominated—that is, other policies provide more benefit for a lower cost. This conclusion is strengthened by the fact that the UPF policy is evaluated in the best case scenario, when *all* direct medical costs are shifted off the patient. In settings in which residual costs still exist (such as registration fees, informal payments, etc), the results here are overestimates of the actual effects.

Also not supported is the most common charitable platform—the short-term “mission trip.”[16] These mission trips are more expensive and less effective than almost every other policy or platform, NGO or otherwise. Other evidence has shown that these trips do not deliver good health outcomes;[16] our results also suggest that these relatively poor outcomes are delivered at high cost.

### Limitations and strengths

This model, like all models, has limitations. First, all models are limited by data. Uganda was chosen because of the higher availability of data compared to other sub-Saharan African contexts, but these data, including the future incidence of cancer, may change and such changes may change our findings. However, the cancer incidence in the country is derived from hospital records, pharmacies, laboratories, and death registries. In addition, the model proved well calibrated to measurable outcomes in Uganda, which, combined with the fact that modeling assumptions introduced to overcome data limitations will underestimate benefits (as discussed in S1 Appendix), reinforces the results.

Secondly, although this model supports the role of an NGO platform, it cannot examine the externalities of the NGO sector—brain drain, the establishment of parallel markets, and engendering dependency of countries on external aid.[61] It also does not measure the persistence of impoverishment into the future, instead measuring the *incidence* of financial catastrophe, as opposed to its prevalence. Finally, there are potential economies of scale that cannot be elucidated by a model limited to a starting population of 10,000 individauls. These are left for future considerations.

The mobile NGO chosen for analysis is an in-country NGO—staffed by local physicians, run locally, and without a large international presence [46]. However, when costs for the mobile NGO are increased to match those of a large international NGO, the platform remains efficient at saving lives, although it loses its financial risk protection efficiency.

Finally, this paper looks at surgical oncology delivered in one country, by a limited bundle of platforms. Because of a lack of available data, the private sector in Uganda had to be excluded, as well as the costs of adjuvant therapy. Generalizability of the results to other contexts must therefore be done with caution, and the policy conclusions must necessarily be viewed as suggestive rather than definitive.

Despite these limitations, this paper, however, accomplishes two things. Methodologically, it presents a novel technique for holistic health system evaluation, able to address multiple barriers to care, platforms of delivery, and domains of outcome simultaneously, with uncertainty incorporated directly into the model, and does so in a way that validates strongly against known population and economic parameters. As such, it overcomes some limitations inherent to prior extended cost-effectiveness analyses,[1, 32–34] on which it is built. Although this paper is obviously limited to surgical oncology in one sub-Saharan African country, the technique developed here can be easily extended to other countries and other conditions.

More importantly, however, the results suggest concrete policy recommendations. If policymakers want to jointly address the sustainable development goals of health, poverty, and equity, mobile surgical units are worth considering. Importantly, although they are examined here as a non-governmental platform, there is no reason that mobile surgical units must remain the purview of the charitable sector. Some countries have already incorporated them into their national surgical delivery plans.[62] Mobile surgical units cannot replace full health-system and surgical system strengthening, and should not be considered the answer to the problem of surgical delivery. However, this model suggests that they can be considered along an expansion path toward full surgical scaleup.

## Conclusion

In cancer patients, an inability to access the surgical system can be lethal, but accessing it can be impoverishing. This paper demonstrates that mobile surgical units are an efficient and equitable method for improving health and protecting against medical impoverishment. These platforms compare well with comprehensive health-systems-strengthening policies. Short-term surgical mission trips, as well as policies that only remove the out-of-pocket cost for care, appear neither efficient nor equitable.

## Supporting Information

S1 Appendix Supplemental methods, results, and figures. (DOCX)

S2 Appendix Model code. (DOCX)

## References

[1] Shrime MG, Verguet S, Johansson KA, Jamison DT, Kruk ME. Task-shifting, universal public finance, or both for the expansion of surgical access in rural Ethiopia: an extended cost-effectiveness analysis. Health policy and planning. 2015:Accepted for publication. 10.1093/heapol/czv121 26719347

[2] Kruk ME, Goldmann E, Galea S. Borrowing and selling to pay for health care in low- and middle-income countries. Health affairs (Project Hope). 2009;28(4):1056–66. Epub 2009/07/15. 19597204 10.1377/hlthaff.28.4.1056

[3] Xu K, Evans DB, Carrin G, Aguilar-Rivera AM, Musgrove P, Evans T. Protecting households from catastrophic health spending. Health affairs (Project Hope). 2007;26(4):972–83. Epub 2007/07/17. 17630440 10.1377/hlthaff.26.4.972

[4] Xu K, Evans DB, Kawabata K, Zeramdini R, Klavus J, Murray CJL. Household catastrophic health expenditure: a multicountry analysis. Lancet. 2003;362:111–7. 10.1016/S0140-6736(03)13861-5 12867110

[5] Meremikwu MM, Ehiri JE, Nkanga DG, Udoh EE, Ikpatt OF, Alaje EO. Socioeconomic constraints to effective management of Burkitt's lymphoma in south-eastern Nigeria. Tropical medicine & international health: TM & IH. 2005;10(1):92–8. Epub 2005/01/19. 15655018 10.1111/j.1365-3156.2004.01348.x

[6] Baicker K, Taubman SL, Allen HL, Bernstein M, Gruber JH, Newhouse JP, et al The Oregon experiment—Effects of Medicaid on clinical outcomes. NEJM. 2013;368:1713–22. 10.1056/NEJMsa1212321 23635051 PMC3701298

[7] World Bank. World Development Indicators 2013 [14 November 2014]. Available from: http://data.worldbank.org/.

[8] Institute for Health Metrics and Evaluation. Global Burden of Disease, 2010. 2012.

[9] Orem JN, Zikusooka CM. Health financing reform in Uganda: How equitable is the proposed National Health Insurance scheme? International journal for equity in health. 2010;9:23 10.1186/1475-9276-9-23 20942899 PMC2967551

[10] Farmer P, Frenk J, Knaul FM, Shulman LN, Alleyne G, Armstrong L, et al Expansion of cancer care and control in countries of low and middle income: a call to action. Lancet. 2010;376:1186–93. 10.1016/S0140-6736(10)61152-X 20709386

[11] Ilbawi AM, Einterz EM, Nkusu D. Obstacles to surgical services in a rural cameroonian district hospital. World journal of surgery. 2013;37(6):1208–15. Epub 2013/03/07. 10.1007/s00268-013-1977-x 23463397

[12] Linden AF, Sekidde FS, Galukande M, Knowlton LM, Chackungal S, McQueen KA. Challenges of surgery in developing countries: a survey of surgical and anesthesia capacity in Uganda's public hospitals. World journal of surgery. 2012;36(5):1056–65. Epub 2012/03/10. 10.1007/s00268-012-1482-7 22402968

[13] Galukande M, Luboga S, Kijjambu SC. Improving recruitment of surgical trainees and training of surgeons in Uganda. East and Central African Journal Surgery. 2006;11(1):17–24.

[14] Casey KM. The global impact of surgical volunteerism. The Surgical clinics of North America. 2007;87(4):949–60, ix. Epub 2007/09/25. 10.1016/j.suc.2007.07.018 17888791

[15] McQueen KA, Hyder JA, Taira BR, Semer N, Burkle FM Jr., Casey KM. The provision of surgical care by international organizations in developing countries: a preliminary report. World journal of surgery. 2010;34(3):397–402. Epub 2009/08/18. 10.1007/s00268-009-0181-5 19685261

[16] Shrime MG, Sleemi A, Thulasiraj RD. Charitable platforms in global surgery: A systematic review of their effectiveness, cost-effectiveness, sustainability, and role in training. World journal of surgery. 2014. 10.1007/s00268-014-2516-0 PMC4179995 24682278

[17] Kruk ME, Rockers PC, Varpilah T, Macauley R. Population preferences for health care in Liberia: Insights for rebuilding a health system. HSR: Health Serv Res. 2011;46(6pt2):2057–78. 10.1111/j.1475-6773.2011.01266.x 21517835 PMC3392998

[18] Flessa S, Moeller M, Ensor T, Hornetz K. Basing care reforms on evidence: the Kenya health sector costing model. BMC health services research. 2011;11:128 Epub 2011/05/31. PubMed Central PMCID: PMCPMC3129293. 10.1186/1472-6963-11-128 21619567 PMC3129293

[19] Gosselin RA, Thind A, Bellardinelli A. Cost/DALY averted in a small hospital in Sierra Leone: What is the relative contribution of different services. World journal of surgery. 2006;30:505–11. 10.1007/s00268-005-0609-5 16528459

[20] Bolkan H, von Schreeb J, Samai M, Bash-Taqi D, Kamara TB, Salvesen Ø, et al. More than 90% of surgical need is unmet in Sierra Leone: Results from a 2013 countrywide survey 2014.

[21] Ministry of Health of Ethiopia. Integrated emergency surgical officers 2015 [5 August 2015]. Available from: http://www.moh.gov.et/ieso.

[22] Hodges AM, Hodges SC. A rural cleft project in Uganda. British journal of plastic surgery. 2000;53(1):7–11. Epub 2000/02/05. 10.1054/bjps.1999.3238 10657442

[23] Hughes CD, Babigian A, McCormack S, Alkire BC, Wong A, Pap SA, et al The clinical and economic impact of a sustained program in global plastic surgery: valuing cleft care in resource-poor settings. Plastic and reconstructive surgery. 2012;130(1):87e–94e. Epub 2012/06/30. 10.1097/PRS.0b013e318254b2a2 22743958

[24] McKinnon B, Harper S, Kaufman JS, Bergevin Y. Removing user fees for facility-based delivery services: a difference-in-differences evaluation from ten sub-Saharan African countries. Health policy and planning. 2014:10.1093/heapol/czu027 PMC4385820 24816570

[25] Pearson L, Shoo R. Availability and use of emergency obstetric services: Kenya, Rwanda, Southern Sudan, and Uganda. International Journal of Gynecology and Obstetrics. 2005;88:208–15. 10.1016/j.ijgo.2004.09.027 15694109

[26] De Brouwere V, Dieng T, Diadhiou M, Witter S, Denerville E. Task shifting for emergency obstetric surgery in district hospitals in Senegal. Reprod Health Matters. 2009;17(33):32–44. 10.1016/S0968-8080(09)33437-0 19523580

[27] Kowalewski M, Mujinja P, Jahn A. Can mothers afford maternal health care costs? User costs of maternity services in rural Tanzania. African journal of reproductive health. 2002;6(1):65–73. Epub 2002/12/13. 12476730

[28] Bonabeau E. Agent-based modeling: Methods and techniques for simulating human systems. Proc Natl Acad Sci U S A. 2002;99(Suppl 3):7280–7. 12011407 10.1073/pnas.082080899 PMC128598

[29] Macal CM, North MJ. Tutorial on agent-based modeling and simulation. Proceedings of the 37th conference on Winter Simulation [Internet]. 2005:[2–15 pp.].

[30] Nandi A, Megiddo I, Prabharakan D, Laxminarayan R. An agent-based simulation modelling approach to extended cost-effectiveness analysis of health interventions. Lancet. 2013;381:S96.

[31] Brown DG, Riolo R, Robinson DT, North M, Rand W. Spatial process and data models: Toward integration of agent-based models and GIS. J Geograph Syst. 2005;7:25–47.

[32] Verguet S, Gauvreau C, Mishra S, MacLennan M, Murphy S, Brouwer E, et al. Tobacco taxation in China: An extended cost-effectiveness analysis. Disease Control Priorities in Developing Countries, 3d edition, Working Paper #5 [Internet]. 2013 18 April 2014. Available from: http://www.dcp-3.org/resources/tobacco-taxation-china-extended-cost-effectiveness-analysis.

[33] Verguet S, Laxminarayan R, Jamison DT. Universal public finance of tuberculosis treatment in India: an extended cost-effectiveness analysis. Health economics. 2014. 10.1002/hec.3019 24497185

[34] Verguet S, Murphy S, Anderson B, Johansson KA, Glass R, Rhenigans R. Public finance of rotavirus vaccination in India and Ethiopia: an extended cost-effectiveness analysis. Vaccine. 2013;31(42):4902–10. 10.1016/j.vaccine.2013.07.014 23871824

[35] Uganda Bureau of Statistics [UBOS], ICF International. Uganda Demographic and Health Survey. Kampala, Uganda and Calverton, Maryland, USA2012. Available from: http://www.measuredhs.com/pubs/pdf/FR264/FR264.pdf.

[36] WorldPop project. Uganda population dataset. In: WorldPop, editor. 2010.

[37] World Population Prospects: The 2012 revision [Internet]. United NAtions. 2012 [cited 4 December 2013]. Available from: http://esa.un.org/unpd/wpp/fertility_figures/data/WPP2012_FERT_PPP_TOTAL_FERTILITY.XLS.

[38] Salem ABZ, Mount TD. A convenient descriptive model of income distribution: the Gamma distribution. Econometrica. 1974;42(6):1115–27.

[39] Cancer Incidence in Five Continents, Volumes I to IX [Internet]. International Agency for Research on Cancer. 2010 [cited 13 December 2013]. Available from: http://ci5.iarc.fr/.

[40] Ferlay J, Shin HR, Bray F, Forman D, Mathers C, Parkin DM. GLOBOCAN 2008 v2.0, Cancer Incidence and Mortality Worldwide: IARC CancerBase No. 102010 10 December 2013. Available from: http://globocan.iarc.fr/.

[41] Hotchkiss DR. The tradeoff between price and quality of services in the Philippines. Social science & medicine (1982). 1998;46(2):227–42. Epub 1998/02/03. 9447645 10.1016/s0277-9536(97)00152-4

[42] McFadden D. Modeling the choice of residential location. In: Yale University CFfRiE, editor. Yale University 1977.

[43] Shimkin MB, Griswold MH, Cutler SJ. Survival in untreated and treated cancer. Annals of Internal Medicine. 1956;45(2):255–67. 13355149 10.7326/0003-4819-45-2-255

[44] Stell PM, Morton RP, Singh SD. Squamous carcinoma of the head and neck: the untreated patient. Clin Otolaryngol 1983;8:7–13. 6831756 10.1111/j.1365-2273.1983.tb01665.x

[45] Bloom HJG, Richardson WW, Harries EJ. Natural history of untreated breast cancer (1805–1933). BMJ. 1962;2(5299):213–21. 13870135 10.1136/bmj.2.5299.213 PMC1925646

[46] Rodas E, Vicuña A, Merrell RC. Intermittent and mobile surgical services: Logistics and outcomes. World journal of surgery. 2005;29:1335–9. 10.1007/s00268-005-7632-4 16151667

[47] Pradhan M, Prescott N. Social risk management options for medical care in Indonesia. Health economics. 2002;11:431–46. 10.1002/hec.689 12112492

[48] Wagstaff A, van Doorslaer E. Catastrophe and impoverishment in paying for health care: with applications to Vietnam 1993–1998. Health economics. 2003;12:921–34. 10.1002/hec.776 14601155

[49] Shrime MG, Dare A, Alkire BC, O'Neill K, Meara JG. Catastrophic expenditure to pay for surgery: a global estimate. Lancet Global Health. 2015;3(Suppl 2):S38–S44. 25926319 10.1016/S2214-109X(15)70085-9 PMC4428601

[50] GBD 2013 Mortality and Causes of Death Collaborators. Global, regional, and national age-sex specific all-cause and cause-specific mortality for 240 causes of death, 1990–2013: a systematic analysis for the Global Burden of Disease Study 2013. Lancet. 2015;385(9963):117–71. 10.1016/S0140-6736(14)61682-2 25530442 PMC4340604

[51] Charnes A, Cooper WW, Rhodes E. Evaluating program and managerial efficiency: an application of data envelopment analysis to program follow through. Management Science. 1981;27(6):668–97.

[52] Center for International Earth Science Information Network—CIESIN—Columbia University, International Food Policy Research Institute—IFPRI, The World Bank, Centro Internacional de Agricultura Tropical—CIAT. Gridded population map, Uganda2011 30 November 2014. Available from: http://sedac.ciesin.columbia.edu/gpw.

[53] Charnes A, Cooper WW, Rhodes E. Measuring the efficiency of decision making units. European Journal of Operational Research. 1978;2(6):429–44.

[54] Horton R, Mullan Z. Health and sustainable development: a call for papers. Lancet. 2015;385:1710–1. 10.1016/S2214-109X(15)00002-9 25937454

[55] World Health Organization. Everybody's business: strengthening health systems to improve health outcomes: WHO's framework for action2007 9 December 2013. Available from: http://www.who.int/healthsystems/strategy/everybodys_business.pdf.

[56] Maclure R. Primary health care and donor dependency: a case study of nongovernment assistance in Burkina Faso. Int J Health Serv. 1995;25(3):539–58. 7591380 10.2190/X4E7-P8LN-3NHR-B6GF

[57] Thaddeus S, Maine D. Too far to walk: maternal mortality in context. Social science & medicine (1982). 1994;38(8):1091–110. 8042057 10.1016/0277-9536(94)90226-7

[58] Finger RP, Kupitz DG, Fenwick E, Balasubramaniam B, Ramani RV, Holz FG, et al The impact of successful cataract surgery on quality of life, household income and social status in South India. PloS one. 2012;7(8):e44268 10.1371/journal.pone.0044268 22952945 PMC3432104

[59] Kuper H, Polack S, Mathenge W, Eusebio C, Wadud Z, Rashid M, et al Does cataract surgery alleviate poverty? Evidence from a multi-centre intervention study conducted in Kenya, the Philippines and Bangladesh. PloS one. 2010;5(11):e15431 10.1371/journal.pone.0015431 21085697 PMC2976760

[60] Xu K, Evans DB, Kadama P, Nabyonga J, Ogwal PO, Nabukhonzo P, et al Understanding the impact of eliminating user fees: Utilization and catastrophic health expenditures in Uganda. Social science & medicine (1982). 2006;62(4):866–76. 16139936 10.1016/j.socscimed.2005.07.004

[61] Maclure R. Primary health care and donor dependency: a case study of nongovernment assistance in Burkina Faso. International Journal of Health Services. 1995;25(3):539–58. 7591380 10.2190/X4E7-P8LN-3NHR-B6GF

[62] Meulenbeek J. The future of Zambia's health care on wheels. International Network of Street Papers [Internet]. 2012 8 November 2015. Available from: http://www.streetnewsservice.org/news/2012/january/feed-314/the-future-of-zambia%E2%80%99s-health-care-on-wheels.aspx.

