# Supplementary material for: Sustainable Development in Surgery: The Health, Poverty, and Equity Impacts of Charitable Surgery in Uganda
Source: PLoS One. 2016 Dec 30;11(12):e0168867. doi: 10.1371/journal.pone.0168867 (PMC5201287; doi:10.1371/journal.pone.0168867)
Supplement: S1 Appendix — (DOCX) [file pone.0168867.s001.docx]

Supplementary Appendix

Model design 2

Individual agents and population parameters 2

Cancer incidence and care-seeking behavior 2

Healthcare delivery in the status quo 4

Policy and platform parameters 4

Outcomes 5

Sensitivity analyses 6

Interpreting efficiency frontiers 7

Interpreting the standardized outcomes panels 7

Supplemental Results 8

Model validation 8

Sector-specific comparisons 8

Acceptability curves 9

Sensitivity analyses 9

Tables 10

Figures 13

Figure S9 21

Figure S10 22

Works Cited 31

# Model design

## Individual agents and population parameters

Model parameters are given in **Table A**. The model was constructed around a synthetic population of 10,000 individuals mimicking that of Uganda. The starting population was matched to the current age, gender, education, income level, and urban/rural divide of Uganda, derived from the Ugandan 2011 Demographic and Health Survey [1]. Individuals in the population faced a baseline mortality rate (absent the cancer-specific mortality rate) and baseline fertility rate [2]. Individuals were connected to multilevel networks: each individual was connected to a simulated “village,” using a distance-based network, to parents, and to spouse and kids, if these existed. On migration and/or death, these connections were broken and re-established, as appropriate.

Individuals were placed on a map of Uganda stochastically, with the probability of occupying any latitude/longitude combination proportional to the population density at that latitude and longitude in Uganda [3]. Internal migration was allowed. Individual income was drawn from a Gamma distribution, parameterized by Ugandan region and for urban or rural dwelling [4-6]. Wealth quintiles were constructed such that 20% of the population fell into each quintile, in expectation. The dollar-value of the national poverty line was calculated such that 24.5% of the population fell below it [5, 7].

Except at model instantiation, individuals entered the population at birth. Pediatric cancers were not modeled; as a result, an individual only faced baseline mortality during the first fifteen years of life. On reaching adulthood, each individual agent was modeled as shown in **Figure A**. While healthy, an individual is allowed to migrate within or out of Uganda. Males in the population sought wives among unmarried women in their networks. Women had children at an exponential rate, such that the average family size in the model was 5 [6]. The total fertility rate was not directly input into the model and was, instead, used as a validation metric.

Validation was performed against the following metrics, which were not directly input into the model: population density, predicted 2050 national population, total fertility rate, cancer incidence, cancer incidence : mortality ratio. Utilization was also validated by location and by wealth quintile. Agents were localized stochastically, with the probability of occupying any point on the map inversely proportional to the population density of that location. Population density was validated graphically, as shown in **Figure 1** of the main text. Fertility was modeled as an exponential random variable set such that the mean household size matched that in Uganda. Total fertility rate was then calculated. Similarly, baseline mortality was the model input and population growth was validated by comparing the model’s predicted 2050 population with published data. Overall cancer incidence was not an input into the model—rather, the incidence of individual cancers was input, and overall cancer incidence and incidence : mortality ratios were output by the model. These were validated against published metrics.

## Cancer incidence and care-seeking behavior

Cancer incidence was modeled after data from the International Agency for Research on Cancer, a branch of the World Health Organization [8, 9]. The age-adjusted cancer incidence in Uganda is given in **Table B**. Per 100,000 in the population, men have an average incidence of 175.7, and women 167.4. Cancer rate was modeled to meet this incidence. The outcomes were modeled as outcomes from the seven most common cancers, shown in **Table B**. This is a simplifying assumption that necessarily omits certain cancers; these cancers are, on average, much rarer and slightly less fatal than the included cancers. The expected bias from this assumption, then, is small.

If an individual gets cancer, he has to decide whether and where to seek care. This was modeled based on a utilization function for healthcare in Uganda published as a white paper in 2006 [10]. Conditional on getting sick, the utility for the agent, *i*, seeking care at hospital *j*, was calculated as follows:

$$U_{ij}=\alpha+\beta X_{i}+\gamma Q_{j}+\delta D_{ij}+\lambda c\left( Y_{i}-P_{j} \right)+\varepsilon_{ij}$$

where *X­_i_* represents a vector of individual and household characteristics. *Q_j_* represents a vector of indicators of quality for facility *j*, and *D_ij_* is the distance from the agent to the facility. The error term follows a standard Gumbel type 1 distribution [11], and coefficient vectors for all other independent variables are taken from previously published surveys in Uganda [10]. These coefficients were varied stochastically, using beta distributions for all values between 0 and 1, and uniform distributions for coefficient values greater than 1.

The cost function was non-linear, to allow for price elasticities to vary with income. Specifically,

$$c\left( Y_{i}-P_{j} \right)=\eta_{1}\ln\left( Y_{i}-P_{j} \right)+\eta_{2}\left[ \ln\left( Y_{i}-P_{j} \right) \right]^{2}$$

The value function *V_ij_* is the deterministic portion of *U_ij_*—$V_{ij}=U_{ij}-\varepsilon_{ij}$—and the value for *V_i0_* (that is, individual *i* chooses not to seek care) was normalized to 0. The choice probabilities follow a nested multinomial logit model, wherein the first choice was whether to seek care at all, and, if so, whether to do so from a public provider or an NGO. Conditional on choosing to seek care, the actual location was chosen by recalculating the value function for each of the possible providers within the chosen nest (public or private). Following Ssewanyana [10], an individual forgoes care with the following probability:

$$P\left( j=0 \right)=\frac{\exp\left( V_{i0} \right)}{\exp\left( V_{i0} \right)+\left[ \sum_{j} \exp\left( \frac{V_{ij}}{\sigma} \right) \right]^{\sigma}}$$

and will choose between public and NGO-provided care with probability

$$P\left( j=K \right)=\frac{\exp\left( \frac{V_{iK}}{\sigma} \right)\left[ \sum_{j\neq K} \exp\left( \frac{V_{ij}}{\sigma} \right) \right]^{\sigma-1}}{\exp\left( V_{i0} \right)+\left[ \sum_{j} \exp\left( \frac{V_{ij}}{\sigma} \right) \right]^{\sigma}}$$

where sigma represents the in-nest correlation.

If an individual chose not to seek care, she remained in her location and faced a cancer-specific untreated mortality rate [12-14]. If she chose to seek care, she traveled to the location of the chosen provider. Because of the large network of roads and buses in Uganda, 80% of individuals were assumed to travel by vehicular transport and the remaining walked. This likely underestimates the actual time necessary to reach care, with a consequent bias of the results toward the null. At any point before care was actually rendered, the individual faced a cancer-specific untreated mortality rate. Once she arrived at the location, she underwent treatment. Treatment length averaged 14 days, with actual treatment completion drawn from a geometric distribution. While under treatment, the individual faced success, mortality, and complication rates that varied by the level of the hospital at which care was received and whether the provider was a surgeon or a task-shifted provider. Conditional on surviving treatment, the individual then returned to her original position. Of note, recurrences were not modeled.

## Healthcare delivery in the status quo

The 53 known national, regional, and district-level hospitals were located on the same map, utilizing latitude and longitude data from previously published data [15] and from Google maps. If the exact location of the district hospital could not be found, the latitude and longitude of the geographic center of its containing town were utilized. Two national hospitals (Mulago and Mbarara) were included in this model. A third national referral hospital named Butabika exists, but this hospital serves as a referral center for mental health patients. The assumption was made that no cancer surgery is undertaken at Butabika. Hospital quality metrics are derived from previously published data [15]. Because the quality score in the utility function above is out of eight possible points, the derived hospital quality score was also corrected to a maximum of eight.

The cost of all surgical procedures in Uganda is not available. The only surgical procedure for which data exist is Caesarian section [16]. Although this is likely an underestimate for cancer surgery, it was used as the cost of surgery in the absence of other costs; the actual cost was drawn from a very wide distribution around this average number. If this cost truly is an underestimate, catastrophic expenditure at baseline would be underestimated, thereby underestimating the financial impact of any policy which removes medical costs, including many of the NGOs. The financial impact of task-sharing is difficult to predict—an underestimate of the cost of surgery would overestimate the induced demand by task-shifting, but the amount of catastrophic expenditure *conditional* on care-seeking would be underestimated.

Non-medical costs of care access (including the costs of transportation, lodging, and food) were calculated as a multiple of the medical cost, following data from multiple sources [13-24]. Indirect costs (for example, lost wages) were not included. On average, 49.3% of health expenditure in Uganda is out-of-pocket [5, 7], not including non-medical costs. At baseline, individuals were assumed to spend 49.3% of the medical cost of any intervention and 100% of the non-medical costs. The remaining 50.7% of medical costs were accounted to the government. The actual out-of-pocket proportion was drawn stochastically from a beta distribution.

## Policy and platform parameters

For policies that included universal public financing (UPF), the full burden of medical costs was transferred to the government; individuals paid nothing out-of-pocket for medical costs, but were still responsible for non-medical costs. Policies that included vouchers transferred the non-medical costs to the governmental sector but did not affect medical costs. For task-shifting, an additional surgical provider was added to each of the district and regional referral hospitals in the country—a change that was reflected in the quality vector above. The scale-up and maintenance costs of a program of task-shifting is given in **Table S1**. These costs come from Mozambique [17], a country of similar population size, and are scaled linearly to match the population of Uganda. These costs include the costs of training, including libraries, books, and computers, and the salaries of task-shifted providers.

The costs faced by a patient seeking care from a at a regional or district hospital or from a task-shifted provider were assumed to be lower than those from a trained surgeon [18, 19]. Controversy exists about how the outcomes of care from a task-shifted provider compare to those from a trained surgeon [18, 19]. To remain conservative, complication and mortality rates were assumed to be slightly higher. This assumption would bias the health benefit from task-shifting toward zero. Similar to the above, its effect on catastrophic expenditure is uncertain—utilization may be over- or under-estimated, depending on the relative elasticities for price and quality by wealth quintile.

Combinations of the three governmental policies were also included, leading to a total of six governmental policies: UPF, task-shifting (TS), UPF + TS, UPF + vouchers (V), TS + V, and UPF + TS + V.

Platforms of surgical delivery by non-governmental organizations were modeled after published data. The “surgical mission trip” (2W) was modeled as a trip to one of the district hospitals [20]. In keeping with previously published data [21-23], the complication rates achieved by those trips were made equivalent to those found at a regional hospital. These trips lasted on average two weeks [20], and recurred on average twice a year. Trip arrival and departure times were drawn randomly from a geometric distribution parameterized to yield these average results. The costs of each trip are given in **Table A** and include the costs of maintaining a sending organization as well as the opportunity costs of individuals going on these trips (measured as their deferred salaries) [24-27].

The mobile surgical unit (MS) was modeled after a similar surgical organization in Ecuador [28]. These costs are extremely small compared to the costs of many other policies. As a result, costs from similar organizations that provide mobile surgical platforms [20] were extracted from their IRS Form 990s and used as sensitivity analyses. This mobile surgical unit was assumed to travel to a different location in Uganda every three months on average. As with the surgical mission trip, the time of departure from each location was drawn from a geometric distribution whose mean was three months.

Finally, the construction of a cancer hospital (CH) at Mbarara was modeled, as is currently proposed (P. Firth, personal communication). Costs were modeled after the construction costs of an NGO hospital in Haiti (unpublished data). The construction costs were amortized over the fifty-year model run. Salaries, education costs, and upkeep accrued yearly.

Reported surgical cases done per year (or per trip) for the reference NGOs were extracted from the literature when available [28], or from annual reports of the reference organizations. These were used to calculate a cost-per-case. Although this assumes that amortized fixed costs scale linearly with the number of cases done—which is incorrect—the results of such an assumption would bias the benefit-per-cost for any of the NGOs toward the null.

## Outcomes

Each policy was run 100 times, over 50 years, with a starting population of 10,000 individuals; the model cycled daily. Accounting for population growth, then, results for the status quo and for the modeled policies were derived from approximately 2.7 trillion person-years of observation each.

Health benefits were measured as the number of deaths averted. Catastrophic expenditure wes counted when individuals spent more than 10% of their pre-health-shock expenditure [29] to access care. Impoverishment was measured as a joint outcome: individuals were counted as impoverished if their expenditure pushed them below the national poverty line *or* if they were in a family whose head of household succumbed to his or her cancer. The system costs and outcomes for delivery under the status quo were calculated first. The results presented in **Table 1** of the main text are incremental over the status quo baseline. That is, the total deaths averted by UPF, *TDA_UPF_­*, is the difference between the deaths experienced under UPF and those experienced in the absence of any policy:

$${TDA}_{UPF}=\frac{1}{Y}\left[ \sum_{y} \sum_{i} D_{i,y,SQ}-\sum_{y} \sum_{i} D_{i,y,UPF} \right]$$

where *y* represents each year, *Y* represents the total number of years, *i* represents individuals in the population, and *D_i,y,p_* represents an indicator which takes the value of 1 if individual *i* died in year *y* under policy *p*. Incremental benefits for each of the policies and for each of the measured benefits and costs were calculated identically. All outcomes were counted for the population as a whole, and by wealth quintile.

In addition to representing the equitable distribution of outcomes graphically (as is done in **Figure 8** in the main paper), a concentration index was constructed for each of the benefits. Concentration indices measure the cumulative proportion of a benefit accrued by cumulative proportions of the population. The best-known concentration index—and the after which the following was modeled—is the Gini index [30].

In this model, income quintiles were normalized such that they each contained 20% of the population. As a result, the concentration index in this paper was calculated as a segmental (trapezoidal) approximation of what would otherwise be a continuous distribution. To derive the index, *G*, let $B_{j}$ be the benefit of interest, accrued by quintile *j*, and let $B_{T}$ be the total benefit accrued across the entire population.

$$G=1-\left[ \frac{0.2}{B_{T}} \right]\sum_{k=1}^{5} \sum_{j=1}^{k-1} \left( B_{j}+B_{k} \right)$$

with $B_{j}=0$. In cases in which some quintiles had negative impacts while others saw benefits (*eg*, some quintiles faced increased catastrophic expenditure while others derived financial risk protection), $B_{j}$ was standardized such that $\min B_{j}=0$, with the remaining benefits scaled commensurately upward.

## Sensitivity analyses

Heterogeneity is inherent to an agent-based model, and probabilistic sensitivity analysis distributions are given in **Table A**. In addition to addressing the uncertainty with parameter variation and heterogeneity, four other sensitivity analyses were performed.

In the first sensitivity analysis, the perspective was changed to that of the ministry of health. For the ministry, the NGOs are essentially cost-less; their costs are exogenous to the country itself, while the benefits accrue directly to the population.

As mentioned above, the costs of the mobile surgical platform are significantly smaller than the costs of other policies and platforms. As a result, with the second sensitivity analysis, we used the costs from other NGOs that man mobile surgical units [20], derived from their IRS filings.

Our base-case measure of impoverishment counts impoverishment for any member of a family whose head of household succumbed to his or her cancer. This is a relatively aggressive measure of impoverishment. In a third sensitivity analysis, we constructed a synthetic poverty line by multiplying the GDP/capita-calculated poverty line in our base case by the average family size in each simulation. In this sensitivity analysis, family members were impoverished only if the loss of income from the late head of household was enough to push the entire family’s wealth below this synthetic poverty line.

Finally, the results in our base-case analysis are presented as yearly averages per 100,000 in the population. In the fourth sensitivity analysis, we presented costs and outcomes as the sum of a discounted stream. The total deaths averted in this sensitivity analysis, then, were calculated as

$${TDA}_{UPF}=\sum_{y} \frac{\sum_{i} D_{i,y,SQ}-\sum_{i} D_{i,y,UPF}}{\left( 1+r \right)^{y}}$$

## Interpreting efficiency frontiers

Results in the main paper and in this Appendix are primarily reported as efficiency frontiers. These show the cost of an intervention along the horizontal axis and the effectiveness (defined by any one of the three outcomes of interest in this paper) along the vertical axis. The “ideal” point on such a graph is the northwest corner—an intervention in that corner provides the most benefit for the least cost. Similarly, the worst place for an intervention to land is in the southeast corner. These interventions cost a lot, relative to the other interventions, and do not provide much benefit.

The dashed line drawn in these figures represents a “frontier” connecting all efficient policies. A policymaker is best served not choosing any intervention below and to the right of this line because these interventions are “dominated” by interventions on the frontier itself. That is, interventions on the frontier will provide more health for less money than dominated interventions.

When only one intervention is on the frontier, it is the single “dominant” alternative for that particular outcome. If more than one intervention is on the frontier, then the choice among the efficient interventions depends on society’s willingness to pay for the outcome of interest. **Table 2** in the main text gives the incremental cost-effectiveness ratio (ICER) of each efficient policy for the outcome of interest. For example, **Table 2** lists an ICER for MS of $160 for health protection, $66 for the protection the protection against catastrophic expenditure, and $62 for protection against impoverishment. In this case, Uganda would choose MS as a policy if it is willing to pay at least that amount for each of these benefits. These ICERs represent the slope of the efficiency frontier between each policy and the one below it.

## Interpreting the standardized outcomes panels

Efficiency frontiers compare intervention effectiveness in only one domain. As an aid to decision-making across both health and financial risk protection domains, a page is borrowed from data envelopment analysis to construct these figures.

With small assumptions on returns to scale, the outcomes of any policy can be standardized by the policy’s cost. That is, by dividing the three outcomes by each intervention’s cost, interventions can be directly compared across more than one outcome. In these graphs, the “ideal “ point is in the northeast corner. An intervention there provides—per dollar spent—the most health benefit (on the horizontal axis) and the most financial benefit (on the vertical axis).

Any policy to the right of another policy provides more health benefit, and any policy to the north of another provides more financial risk protection benefit. Choosing between any two policies that can be connected by a diagonal line running northwest-to-southeast requires determining how much of one outcome society is willing to trade off for the other. That choice is beyond the scope of this paper.

# Supplemental Results

## Model validation

The model was validated against the following known metrics: population density, predicted 2050 national population, total fertility rate, cancer incidence, cancer incidence : mortality ratio. Utilization was also validated by location and by wealth quintile.

### Population density

The population density of Uganda by geographic location is shown in **Figure B**. Actual population density is taken from Columbia University’s Gridded Population of the World project [31].

### Predicted 2050 national population

The Population Reference Bureau predicts that Uganda’s population in 2050 will be 114 million [32]. The distribution of model predictions is shown in **Figure C**. Note that these future population estimates are scaled linearly by the ratio of the current population of Uganda to the initial model population size.

### Total fertility rate

The United Nations estimates the total fertility rate for Uganda to be 6.38 [2]. The model estimated a total fertility rate of 6.46.

### Cancer incidence

Published cancer estimates are shown in Table 2. Overall, the projected incidence of cancer in Uganda is 171 cases per 100,000 individuals [8, 9]. **Figure D** shows the distribution of model predictions of cancer incidence.

### Incidence : mortality ratio

The calculated incidence-to-mortality ratio from published data is approximately 1.268 in Uganda [8, 9]. **Figure E** shows the distribution of model predictions.

### Utilization by location and wealth

Utilization is influenced by multiple factors, including wealth and distance to the nearest provider [10]. **Figure F** shows baseline utilization of services by wealth quintile, and **Figure G** shows the location of the district, regional, and national referral hospitals, juxtaposed with the probability of utilizing care given a cancer diagnosis. The distributions of location, urban/rural living situation, and wealth are not independent, and utilization depends on the joint distribution of the three. This is shown in **Figure H**.

## Sector-specific comparisons

### Government policies alone

If the NGO sector is excluded, cancer deaths are efficiently averted by TS, UPF + TS, and UPF + TS + V. Catastrophic expenditure is only efficiently averted by UPF + V, while UPF + TS + V is the only efficient strategy to avert impoverishment, although the difference between the two strategies for financial outcomes is minimal (**Figure I**).

### NGOs alone

Under base case assumptions, MS is the only efficient NGO strategy (**Figure J**). It provides benefits at a cost of $155 per life saved, $66 per case of catastrophic expenditure, and $62 per case of impoverishment averted. Across all levels of willingness-to-pay for averting deaths, catastrophic expenditure, or impoverishment, MS dominates the other two platforms (see **Figure K**)

## Acceptability curves

### Governmental policies alone

Acceptability curves for government policies are shown in **Figure I**. TS is most likely to be cost-effective if Uganda is willing to pay less than $525 per life saved, $82 per case of catastrophic expenditure averted, or $165 per case of impoverishment averted. If Uganda is willing to pay more than $855 per life saved or more than $470 per case of impoverishment, then UPF + TS + V is preferred.

### NGOs alone

Acceptability curves for the NGO policies are given in **Figure L**. For all three outcomes—health, catastrophic expenditure, and impoverishment—MS dominates.

### All policies and platforms

Acceptability curves for all nine policies and platforms are given in **Figure M**.

## Sensitivity analyses

### Ministry of Health perspective

From the perspective of the Ministry of Health of Uganda, the non-governmental organizations are free. Their actual costs are exogenous to the ministry’s budget, and, as a result, including them in a health delivery platform is relatively costless. Results of an analysis from this perspective are given in **Figure N**. These analyses do not account for downstream costs in terms of dependence on foreign aid with the presence of NGO.

### Increased cost of the mobile surgical platform

An increase in the cost of the mobile surgical platform to match the cost of external NGOs predictably changed its cost-effectiveness ratio. It remained an efficient policy for the prevention of cancer deaths, but lost its dominance over other policies for the provision of all three benefits, as shown in **Figures O** and **P**.

### Re-imagining familial impoverishment

Our findings are robust to how impoverishment is calculated. With a more conservative definition of impoverishment, the findings are unchanged from those in our base case scenario.

### Discounting of future costs and benefits

Results are presented in **Table C** as a discounted sum of a stream of benefits and costs (with a discount rate of 3% annually) As can be seen from **Figures Q** and **R**, the cost-effectiveness relationships of various policies do not actually change.

# Tables

### Table A

| **Variable [Reference]** | **Units** | **Mean** | **Distribution** | **First parameter** | **Second parameter** |
| --- | --- | --- | --- | --- | --- |
| *Population and macroeconomic* |  |  |  |  |  |
| Rural population proportion [5] |  | 0.85 |  |  |  |
| GDP/capita [5, 7] | USD | 352 |  |  |  |
| Monthly income (Mean) [6] | UGX | 303,700 |  |  |  |
| Urban [6] | UGX | 660,200 |  |  |  |
| Rural [6] | UGX | 222,600 |  |  |  |
| Gini coefficient [5] |  | 42.6 |  |  |  |
| Poverty line [5, 7] | % | 24.5 |  |  |  |
| Average family size^‡^ [6] |  | 5 | Uniform | 4 | 6 |
| *Costs and microeconomic* |  |  |  |  |  |
| Catastrophic expenditure threshold [29] |  | 0.1 |  |  |  |
| Average cost of surgery^†^ [16] | USD | 143.43 | Gamma | Gini/10 | 143.43 * 10/Gini |
| Out-of-pocket proportion^‡^ [5, 7] |  | 0.493 | Beta | 49.3 | 50.7 |
| Non-medical multiplier^†^ [18, 33-44] |  | 1.23 | Uniform | 0.615 | 2.46 |
| District cost deflator^‡^ [18, 19] |  | 0.68 | Beta | 6.8 | 3.2 |
| Regional cost deflator^‡^ [18, 19] |  | 0.8 | Uniform | 0.715 | 0.909 |
| *Surgical and disease outcomes* |  |  |  |  |  |
| District complication multiplier^‡^ [18, 19] |  | 1.35 | Uniform | 1.2 | 1.5 |
| Regional complication multiplier^‡^ [18, 19] |  | 1.105 | Uniform | 1.01 | 1.2 |
| Mean treatment length^§^ | days | 14 | Geometric | 0.067 |  |
| Surgical complication rate^‡^ [45-47] |  | 0.157 | Beta | 1.57 | 8.43 |
| Perioperative mortality^‡^ [45-47] |  | 0.03 | Beta | 0.3 | 9.7 |
| Complication mortality^‡^ [19] |  | 0.02 | Beta | 0.2 | 9.8 |
| Five-year untreated survival^§^ [12-14] |  | 0 - 0.161 | Geometric | varies by tumor type | |
| *Task-sharing costs* |  |  |  |  |  |
| Training and deployment costs years 1-3^‡^ [17] | USD | 10,287 | Gamma | Gini/10 | 10,287 * 10/Gini |
| Training and deployment costs, later years^‡^ [17] | USD | 4,935 | Gamma | Gini/10 | 4935 * 10/Gini |
| *Non-governmental organizations* |  |  |  |  |  |
| Short-term trip cost (per trip)^‡^ [24-27] | USD | 207,606 | Gamma | Gini/10 | 207,606 * 10/Gini |
| Mobile surgical platform cost (per year)^‡^ [28] | USD | 165,855 | Gamma | Gini/10 | 165,855 * 10/Gini |
| Specialty cancer hospital^‡^ [Farmer, unpublished] | USD | 481,738 | Gamma | Gini/10 | 481,738 * 10/Gini |

Model parameterization. Mean values and distributions for sensitivity analysis are given. Values for which no distribution is given were not varied across simulation runs. All costs are corrected to 2010 US dollars. USD = US dollar. UGX = Ugandan shillings. Costs of surgical platforms amortize fixed costs over the lifetime of the policy. ^†^Varied at the hospital level. ^‡^Varied at the simulation level. ^§^Varied at the individual level.

### Table B

| **Cancer** | **Incidence / 100,000 M** | **Incidence / 100,000 F** |
| --- | --- | --- |
| Prostate | 48.2 | — |
| Cervix | — | 44.4 |
| Breast | — | 27.5 |
| Esophagus | 24.8 | 10.4 |
| Kaposi’s sarcoma | 21.8 | 10.9 |
| Colorectal | 7.7 | 6.6 |
| Oral cavity | 4.8 | 2.3 |
| **All cancers** | **175.7** | **167.4** |

Age-adjusted cancer incidence in Uganda [8, 9]. See text for details.

### Table C

|  |  | Platforms and Policies | | | | | | | | |
| --- | --- | --- | --- | --- | --- | --- | --- | --- | --- | --- |
|  |  | **UPF** | **TS** | **UPFTS** | **UPF^V^** | **TS^V^** | **UPFTS^V^** | **2W** | **MS** | **CH** |
| System cost/100K |  | $83,324 | $7,061 | $91,933 | $602,283 | $343,549 | $611,920 | $1,021,357 | $142,969 | $1,319,884 |
| Cancer Deaths/ 100K | Poorest | 138 | -82 | 120 | 1396 | 791 | 1382 | 55 | 1700 | 882 |
|  | Poor | 121 | 178 | 333 | 676 | 685 | 784 | 56 | 1017 | 649 |
|  | Middle | 23 | 101 | 269 | 601 | 417 | 683 | -18 | 869 | 713 |
|  | Rich | 55 | 184 | 181 | 500 | 308 | 577 | 34 | 824 | 767 |
|  | Richest | -11 | 19 | 97 | 436 | 64 | 485 | -17 | 712 | 571 |
|  | ***Avg*** | ***65*** | ***80*** | ***200*** | ***722*** | ***453*** | ***782*** | ***22*** | ***1024*** | ***717*** |
| Cat Exp/  100K | Poorest | -628 | -657 | -1466 | 3633 | -2790 | 3633 | 206 | 2204 | 1469 |
|  | Poor | -320 | -1063 | -1406 | 5060 | -957 | 5060 | 278 | 2714 | 2067 |
|  | Middle | -76 | -1156 | -1065 | 5895 | 1514 | 5895 | 11 | 2753 | 2347 |
|  | Rich | 467 | -1260 | -69 | 6841 | 4106 | 6841 | 336 | 2977 | 2727 |
|  | Richest | 1124 | -300 | 1193 | 6823 | 5410 | 6823 | 120 | 2126 | 1898 |
|  | ***Avg*** | ***114*** | ***-887*** | ***-563*** | ***5650*** | ***1457*** | ***5650*** | ***190*** | ***2555*** | ***2102*** |
| Impoverishment  /100K | Poorest | -227 | -1840 | -1952 | 13703 | -480 | 13654 | 436 | 8575 | 5697 |
|  | Poor | 230 | 384 | 614 | 840 | 1359 | 1051 | -8 | 1636 | 1067 |
|  | Middle | 20 | 105 | 460 | 934 | 826 | 912 | -38 | 1447 | 1192 |
|  | Rich | 84 | 442 | 330 | 460 | 501 | 616 | -40 | 1364 | 1141 |
|  | Richest | -79 | 10 | 248 | 157 | -133 | 80 | -118 | 982 | 586 |
|  | ***Avg*** | ***6*** | ***-180*** | ***-60*** | ***3219*** | ***415*** | ***3262*** | ***46*** | ***2801*** | ***1937*** |

Incremental costs and benefits above status quo, presented as the discounted sum of a fifty-year stream. Discount rate = 3%. UPF = universal public finance. TS = task-shifting. V = vouchers. 2W = short-term mission trips. MS = mobile surgical units. CH = cancer hospital. Negative cases of impoverishment or catastrophic expenditure averted implies cases *created* by the policy.

# Figures

### Figure A


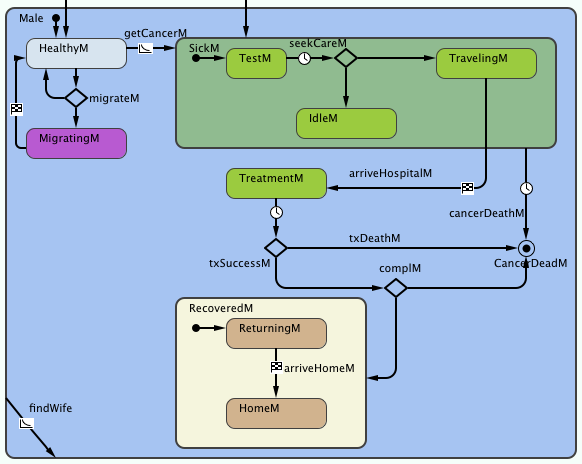


An adult male in the Ugandan population. Each agent remains healthy until he gets cancer (*getCancerM*), at which point he decides whether or not to get care and from where (*TestM* and *seekCareM*). If he chooses not to seek care, he lives with untreated disease (*IdleM*) until he dies. If he seeks care, he travels to get it (*TravelingM* and *TreatmentM*), at which point he faces the risk of complications (*complM*) and peri-operative mortality (*txDeathM*). If he survives these, he returns to his prior position (*ReturningM* and *HomeM*). In the model, men seek wives (*findWife*); children are born to women and included in the male network only if that man is a woman’s spouse. Internal migration (*MigratingM*) is allowed. In this figure, cancer death (*CancerDeadM*) is counted when an individual dies of untreated disease (including while traveling to treatment, *cancerDeathM*), dies as a result of treatment (*txDeathM*), or dies as a result of complications due to treatment (unlabeled arrow from *complM* to *CancerDeadM*). Not shown in this figure is the baseline mortality rate faced by every agent in the model

### Figure B

| Actual population density [31] | **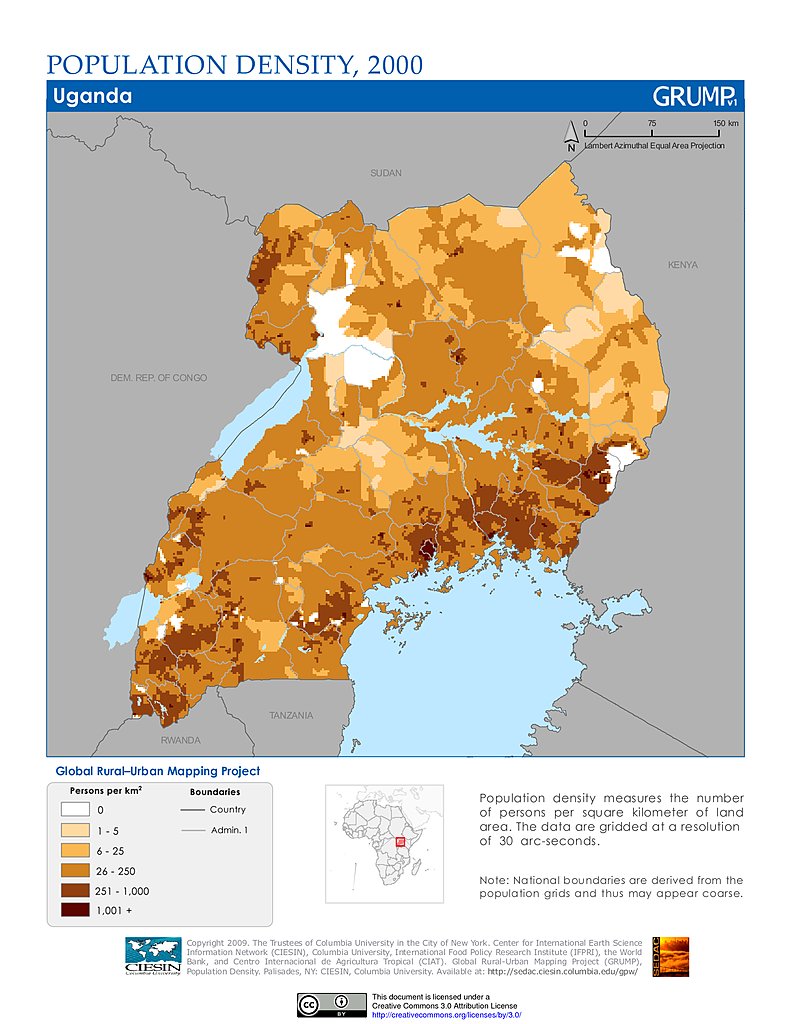** |
| --- | --- |
| Modeled population density | **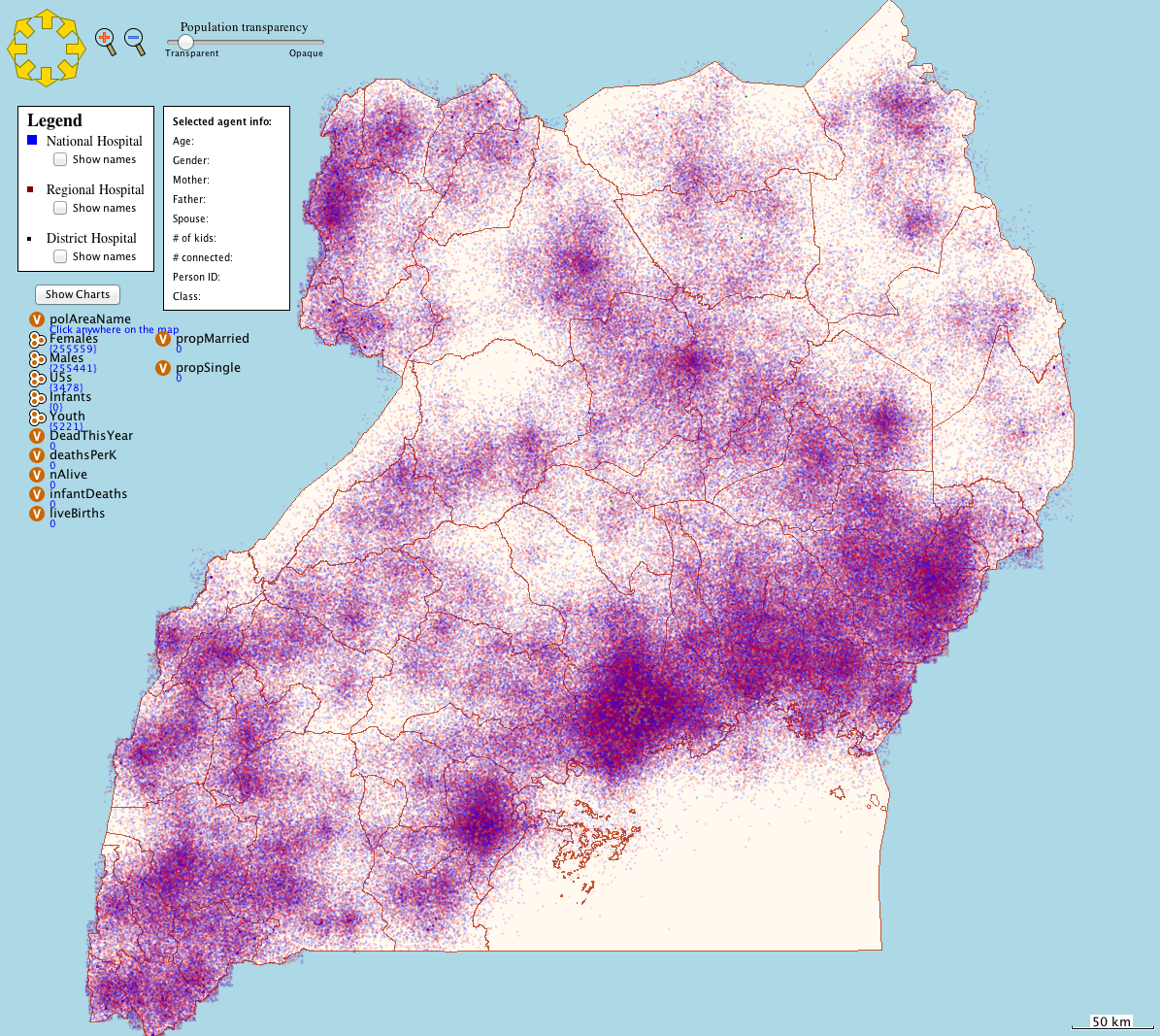** |

Actual vs. modeled population density in Uganda. In the model, each red and blue dot represents one person.

### Figure C


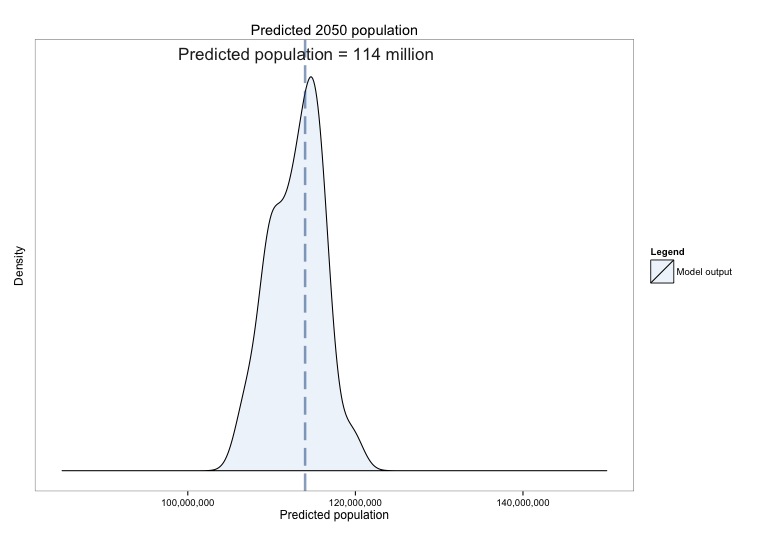


Predicted 2050 Ugandan population. Dashed line = Population Reference Bureau estimate [32]. Shaded region = model output, 100 runs.

### Figure D


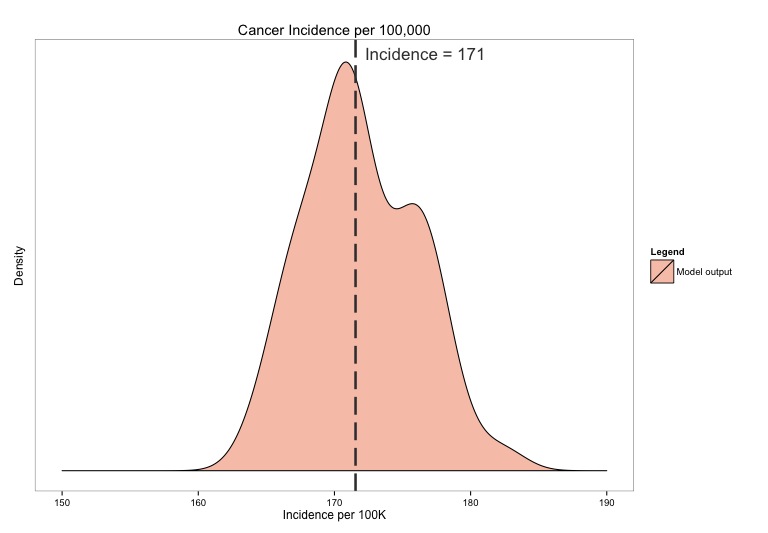


Cancer incidence per 100,000. Dashed line = IARC estimate [8, 9]. Shaded region = model output, 100 runs.

### Figure E


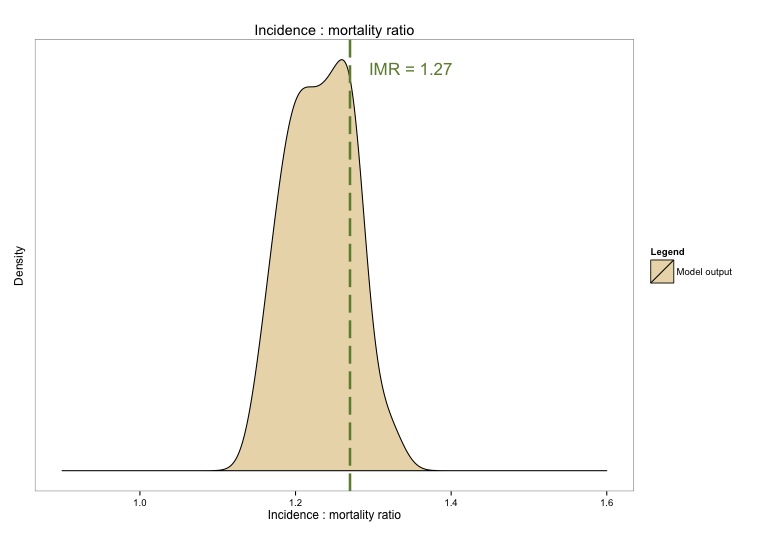


Incidence : mortality ratio for cancer in Uganda. Dashed line represents published rates (1.268). The mean from the model is 1.26, and the distribution is represented above.

### Figure F


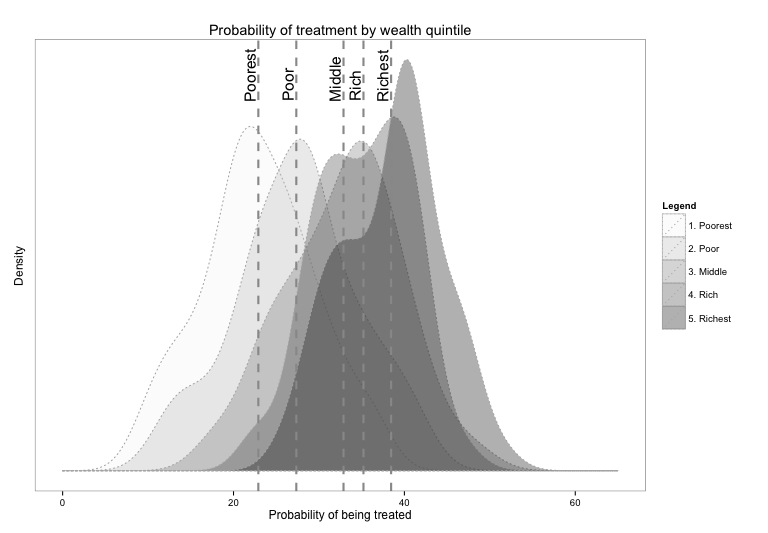


The probability of health utilization by wealth quintile. Dashed lines represent the mean utilization by individuals in each wealth quintile conditional on getting cancer. The distributions represent 100 runs of the model.

### Figure G

| Hospital locations [15] |  |
| --- | --- |
| Probability of utilization by location | 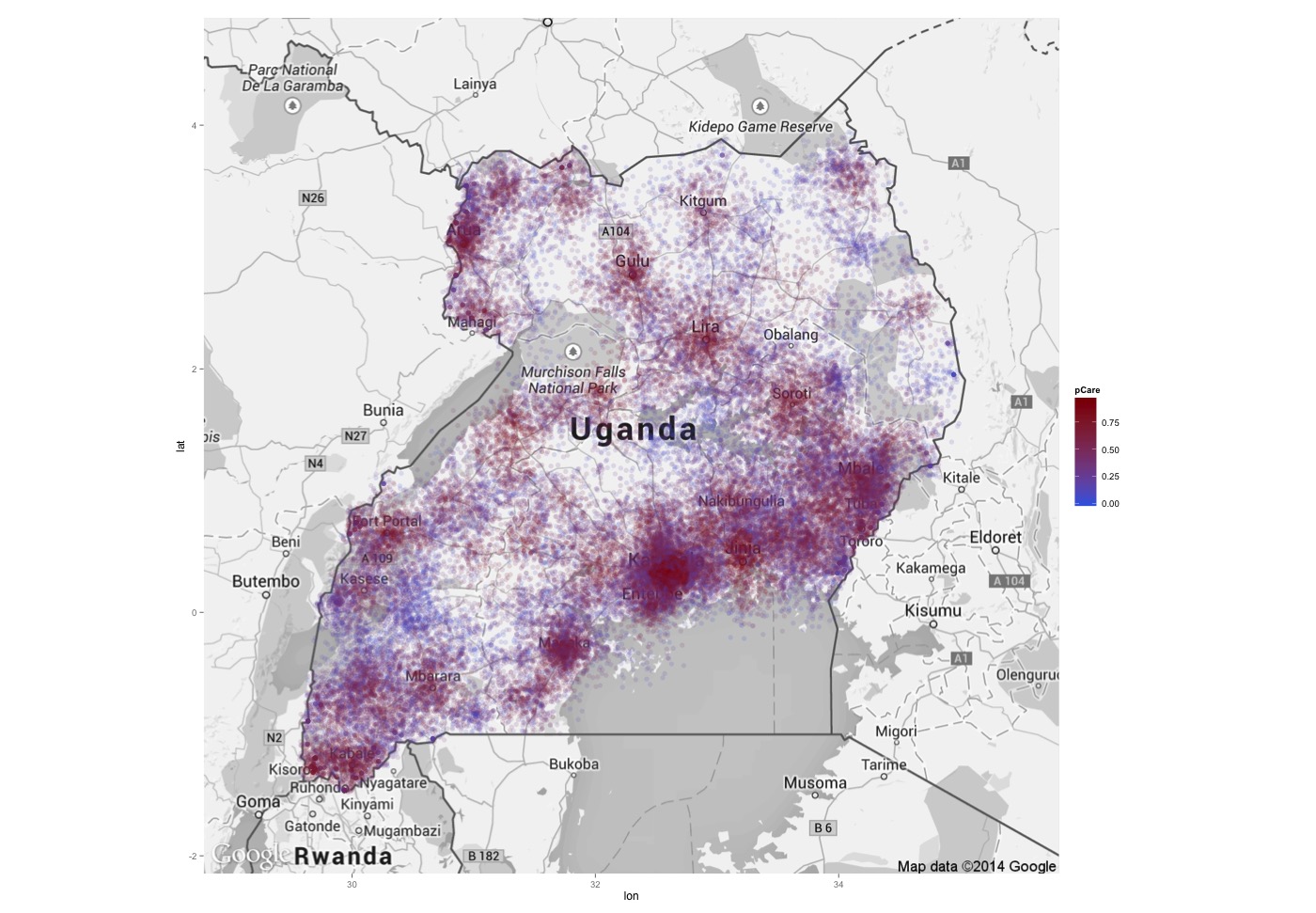 |

Hospital locations in Uganda (top) and probability of healthcare utilization given location in the model (bottom; red = highest, blue = lowest).

### Figure H

| Probability of utilization by location | 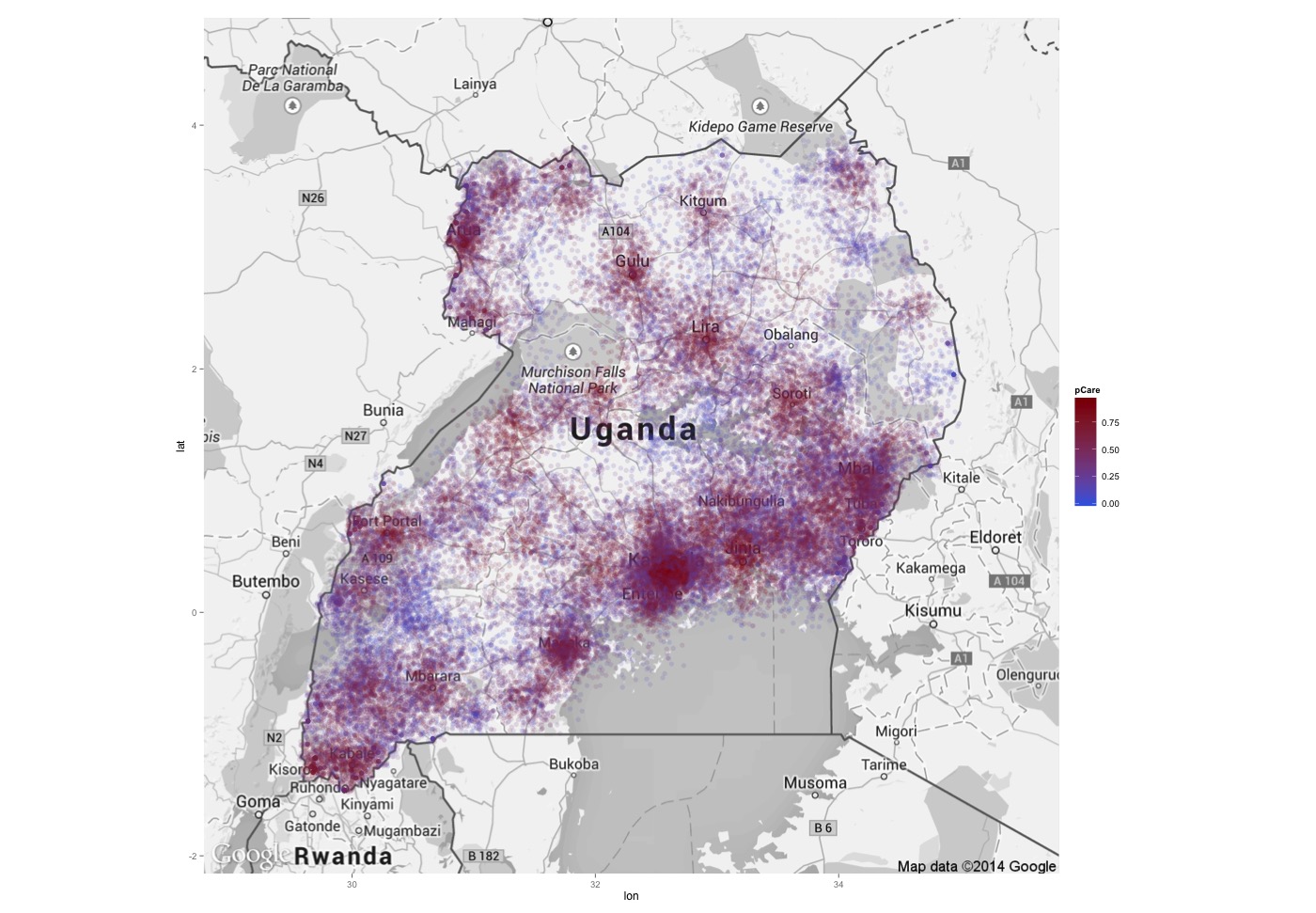 |
| --- | --- |
| Joint probability of utilization by location, urban/rural, and income. | 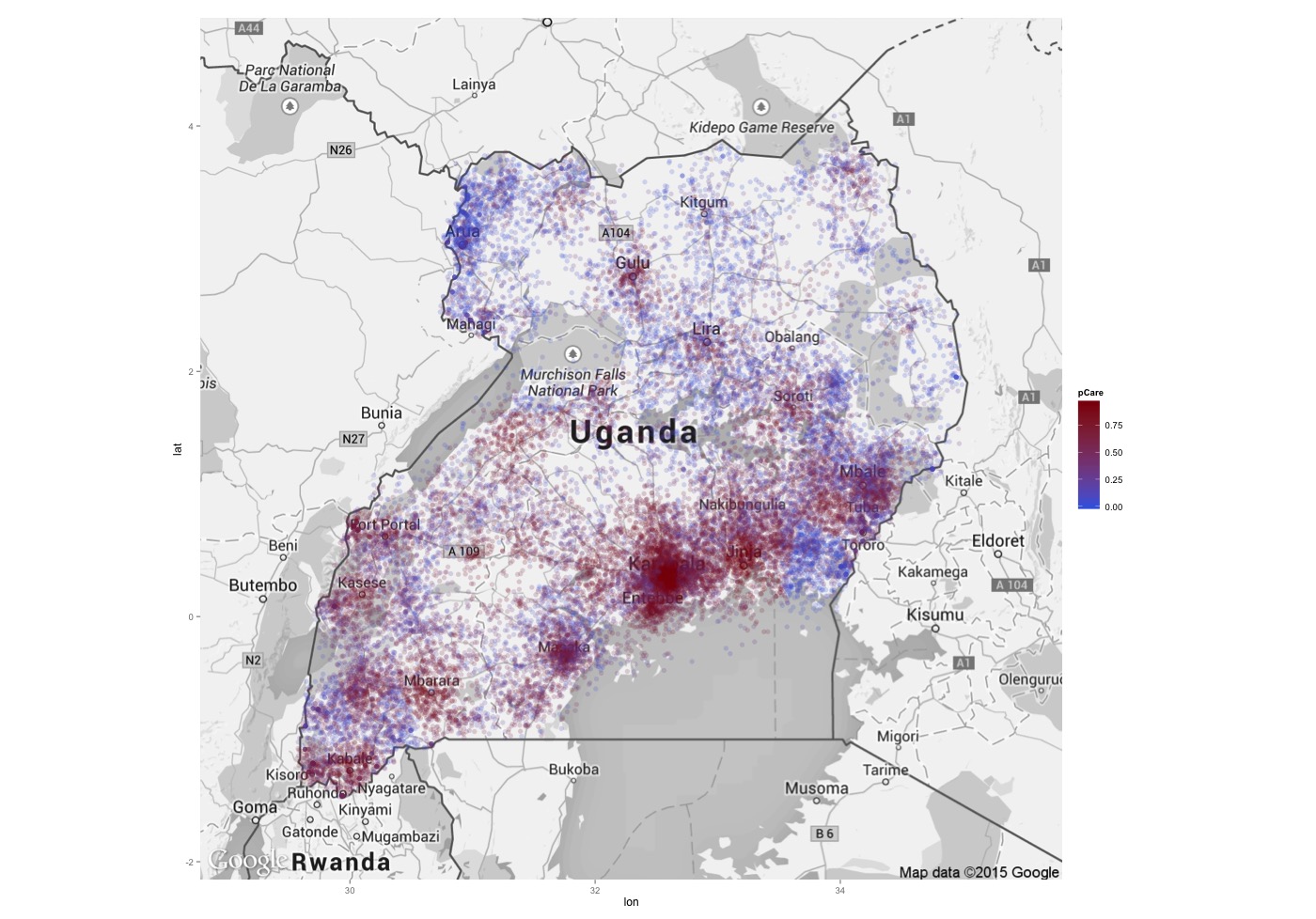 |

Probability of utilization by location alone (top) and the joint probability of utilization by location, wealth, and urban/rural living (bottom; red = highest, blue = lowest).

## Figure I

|  | **a) Deaths averted** |
| --- | --- |
|  | **b) Catastrophic expenditure averted** |
|  | **c) Impoverishment averted** |

Efficiency frontiers for governmental policies. Incremental cost-effectiveness ratios are given in Table 2. All policies are dominated except for UPF + TS + V (all outcomes) and TS alone (death and impoverishment). UPF = universal public financing, TS = task shifting, V = vouchers.

## Figure J

|  | **a) Deaths averted** |
| --- | --- |
|  | **b) Catastrophic expenditure averted** |
|  | **c) Impoverishment averted** |

Efficiency frontiers for NGO platforms. For all outcomes, the mobile surgical unit dominates. Of the remaining two, the two-week mission trip is dominated. MS = mobile surgical unit. CH = cancer hospital. 2W = two-week surgical mission

### Figure K

|  | **a) Deaths averted** |
| --- | --- |
|  | **b) Catastrophic expenditure averted** |
|  | **c) Impoverishment averted** |

Acceptability curves for governmental policies alone. Willingness-to-pay thresholds at which the preferred strategy changes are marked by dashed lines. UPF = universal public financing, TS = task-shifting, V = vouchers.

### Figure L

|  | **a) Deaths averted** |
| --- | --- |
|  | **b) Catastrophic expenditure averted** |
|  | **c) Impoverishment averted** |

Acceptability curves for NGO platforms. For all outcomes, the mobile surgical unit dominates. MS = mobile surgical unit. CH = cancer hospital. 2W = two-week surgical mission.

### Figure M

|  | **a) Deaths averted** |
| --- | --- |
|  | **b) Catastrophic expenditure averted** |
|  | **c) Impoverishment averted** |

Acceptability curves for all policies and platforms. Willingness-to-pay thresholds at which the preferred strategy changes are marked by dashed lines. UPF = universal public finance. TS = task-shifting. V = vouchers. MS = mobile surgical unit. CH = cancer hospital. 2W = two-week surgical mission

### Figure N

Standardized health and financial risk protection benefits of the six governmental policies and three NGO platforms from the standpoint of the ministry of health. Policies to the upper right are preferred. All governmental platforms are concentrated near the origin. From left-to-right, the three NGO platforms are two-week trips, cancer hospital, and the mobile surgical unit. UPF = universal public finance, TS = task-sharing, V = vouchers.

### Figure O

|  | **a) Deaths averted** |
| --- | --- |
|  | **b) Catastrophic expenditure averted** |
|  | **c) Impoverishment averted** |

Acceptability curves for all policies and platforms with an assumption of increased cost for MS Willingness-to-pay thresholds at which the preferred strategy changes are marked by dashed lines. UPF = universal public finance. TS = task shifting. V = vouchers. MS = mobile surgical unit. CH = cancer hospital. 2W = two-week surgical mission

### Figure P

Health vs. financial risk protection when the cost of MS is increased. The platform is no longer dominant.

### Figure Q

|  | **a) Deaths averted** |
| --- | --- |
|  | **b) Catastrophic expenditure averted** |
|  | **c) Impoverishment averted** |

Efficiency frontiers when results are presented as discounted streams instead of yearly averages. No difference is noted between these results and the base case.

### Figure R

|  | **a) Catastrophic expenditure averted** |
| --- | --- |
|  | **b) Impoverishment averted** |

Health vs. financial risk protection when results are presented as discounted streams instead of yearly averages. No difference is noted between these graphs and the base case results.

# Works Cited

1. Uganda Bureau of Statistics [UBOS] and ICF International, *Uganda Demographic and Health Survey*. 2012: Kampala, Uganda and Calverton, Maryland, USA.

2. United Nations Department of Economic and Social Affairs, *World Population Prospects: The 2012 revision*. 2012, United NAtions.

3. WorldPop project, *Uganda population dataset*, WorldPop, Editor. 2010.

4. Salem, A.B.Z. and T.D. Mount, *A convenient descriptive model of income distribution: the Gamma distribution.* Econometrica, 1974. **42**(6): p. 1115-1127.

5. World Bank. *World Development Indicators*. 2013 14 November 2014]; Available from: <http://data.worldbank.org/>.

6. Uganda Bureau of Statistics [UBOS] *Uganda National Household Survey Report*. 2010.

7. Orem, J.N. and C.M. Zikusooka, *Health financing reform in Uganda: How equitable is the proposed National Health Insurance scheme?* Int J Equity Health, 2010. **9**: p. 23.

8. Ferlay, J., et al., *Cancer Incidence in Five Continents, Volumes I to IX*, in *IARC CancerBase No 9*. 2010, International Agency for Research on Cancer: Lyons, France.

9. Ferlay, J., et al. *GLOBOCAN 2008 v2.0, Cancer Incidence and Mortality Worldwide: IARC CancerBase No. 10*. 2010.

10. Ssewanyana, S., et al., *Demand for health care services in Uganda: Implications for poverty reduction*. 2006, Center for the Study of African Economies.

11. McFadden, D., *Modeling the choice of residential location*, C.F.f.R.i.E. Yale University, Editor. 1977: Yale University.

12. Shimkin, M.B., M.H. Griswold, and S.J. Cutler, *Survival in untreated and treated cancer.* Annals of Internal Medicine, 1956. **45**(2): p. 255-267.

13. Stell, P.M., R.P. Morton, and S.D. Singh, *Squamous carcinoma of the head and neck: the untreated patient.* Clin Otolaryngol 1983. **8**: p. 7-13.

14. Bloom, H.J.G., W.W. Richardson, and E.J. Harries, *Natural history of untreated breast cancer (1805-1933).* BMJ, 1962. **2**(5299): p. 213-221.

15. Linden, A.F., et al., *Challenges of surgery in developing countries: a survey of surgical and anesthesia capacity in Uganda's public hospitals.* World J Surg, 2012. **36**(5): p. 1056-65.

16. World Health Organization. *WHO-CHOICE, Quantities, units, and prices*. 4 July 2014]; Available from: <http://www.who.int/choice/cost-effectiveness/inputs/en/>.

17. Kruk, M.E., et al., *Economic evaluation of surgically trained assistant medical officers in performing major obstetric surgery in Mozambique.* BJOG, 2007. **114**(10): p. 1253-1260.

18. Kifle, Y.A. and T.H. Nigatu, *Cost-effectiveness analysis of clinical specialist outreach as compared to referral system in Ethiopia: an economic evaluation.* Cost Effectiveness and Resource Allocation, 2010. **8**(1): p. 13.

19. Shrime, M.G., et al., *Task-shifting, universal public finance, or both for the expansion of surgical access in rural Ethiopia: an extended cost-effectiveness analysis*, in *Disease Control Priorities in Developing Countries, 3rd edition*, D.T. Jamison, Editor. 2014.

20. Shrime, M.G., A. Sleemi, and R.D. Thulasiraj, *Charitable platforms in global surgery: A systematic review of their effectiveness, cost-effectiveness, sustainability, and role in training.* World J Surg, 2014.

21. Huijing, M.A., et al., *Facial reconstruction in the developing world: a complicated matter.* Br J Oral Maxillofac Surg, 2011. **49**(4): p. 292-6.

22. Maine, R.G., et al., *Comparison of fistula rates after palatoplasty for international and local surgeons on surgical missions in Ecuador with rates at a craniofacial center in the United States.* Plast Reconstr Surg, 2012. **129**: p. 319e.

23. Marck, R., et al., *Early outcome of facial reconstructive surgery abroad: a comparative study.* Eur J Plast Surg, 2010. **33**(4): p. 193-197.

24. Gosselin, R.A., G. Gialamas, and D.M. Atkin, *Comparing the cost-effectiveness of short orthopedic missions in elective and relief situations in developing countries.* World J Surg, 2011. **35**(5): p. 951-5.

25. Hodges, A.M. and S.C. Hodges, *A rural cleft project in Uganda.* Br J Plast Surg, 2000. **53**(1): p. 7-11.

26. Magee, W.P., R. Vander Burg, and K.W. Hatcher, *Cleft lip and palate as a cost-effective health care treatment in the developing world.* World J Surg, 2010. **34**(3): p. 420-7.

27. Moon, W., H. Perry, and R.M. Baek, *Is international volunteer surgery for cleft lip and cleft palate a cost-effective and justifiable intervention? A case study from East Asia.* World J Surg, 2012. **36**(12): p. 2819-30.

28. Rodas, E., A. Vicuña, and R.C. Merrell, *Intermittent and mobile surgical services: Logistics and outcomes.* World J Surg, 2005. **29**: p. 1335-1339.

29. Xu, K., et al., *Household catastrophic health expenditure: a multicountry analysis.* Lancet, 2003. **362**: p. 111-117.

30. GBD 2013 Mortality and Causes of Death Collaborators, *Global, regional, and national age-sex specific all-cause and cause-specific mortality for 240 causes of death, 1990-2013: a systematic analysis for the Global Burden of Disease Study 2013.* Lancet, 2015. **385**(9963): p. 117-71.

31. Center for International Earth Science Information Network *Gridded population map, Uganda*. 2005.

32. Population Reference Bureau *World Population Data Sheet*. 2013.

33. Afsana, K., *The Tremendous Cost of Seeking Hospital Obstetric Care in Bangladesh.* Reproductive Health Matters, 2004. **12**(24): p. 171-180.

34. Borghi, J., et al., *Household costs of healthcare during pregnancy, delivery, and the postpartum period: a case study from Matlab, Bangladesh.* Journal of Health Population and Nutrition, 2006. **24**(4): p. 446-455.

35. Dhar, R.S., et al., *Direct cost of maternity-care services in south Delhi: a community survey.* Journal of Health Population and Nutrition, 2009. **27**(3): p. 368-378.

36. Hamid, S.A., S.M. Ahsan, and A. Begum, *Disease-specific impoverishment impact of out-of-pocket payments for health care: evidence from rural Bangladesh.* Applied Health Economics and Health Policy, 2014. **doi: 10.1007/s40258-014-0100-2**.

37. Khan, S.H., *Free does not mean affordable: maternity patient expenditures in a public hospital in Bangladesh.* Cost Eff Resour Alloc, 2005. **3**(1): p. 1.

38. Kumar, G.A., et al., *Burden of out-of-pocket expenditure for road traffic injuries in urban India.* BMC Health Services Research, 2012. **12**: p. 285.

39. Mashreky, S.R., et al., *Hospital burden of road traffic injury: major concern in primary and secondary level hospitals in Bangladesh.* Public Health, 2010. **124**(4): p. 185-9.

40. Mohanty, S.K. and A. Srivastava, *Out-of-pocket expenditure on institutional delivery in India.* Health Policy Plan, 2013. **28**(3): p. 247-62.

41. Pakseresht, S., et al., *Expenditure audit of women with breast cancer in a tertiary care hospital of Delhi.* Indian J Cancer, 2011. **48**(4): p. 428-37.

42. Reddy, G.M., et al., *Extent and determinants of cost of road traffic injuries in an Indian city.* Indian J Med Sci, 2009. **63**(12): p. 549-56.

43. Reddy, G.M., A. Singh, and D. Singh, *Community based estimation of extent and determinants of cost of injuries in a north Indian city.* Indian J Med Sci, 2012. **66**(1-2): p. 23-9.

44. Shrime, M.G., et al., *Catastrophic expenditure to pay for surgery: a global estimate.* Lancet Commission on Global Surgery, 2015.

45. Pearson, L. and R. Shoo, *Availability and use of emergency obstetric services: Kenya, Rwanda, Southern Sudan, and Uganda.* International Journal of Gynecology and Obstetrics, 2005. **88**: p. 208-215.

46. Kayondo, M., et al., *Predictors and outcome of surgical repair of obstetric fistula at a regional referral hospital, Mbarara, western Uganda.* BMC Urology, 2011. **11**: p. 23.

47. Warf, B., *Comparison of 1-year outcomes for the Chhabra and Codman-Hakim Micro Precision shunt systems in Uganda: a prospective study in 195 children.* J Neurosurg, 2005. **102**: p. 358-362.
