# Supplementary material for: Sustainable Development in Surgery: The Health, Poverty, and Equity Impacts of Charitable Surgery in Uganda
Source: PLoS One. 2016 Dec 30;11(12):e0168867. doi: 10.1371/journal.pone.0168867 (PMC5201287; doi:10.1371/journal.pone.0168867)
Supplement: S2 Appendix — (DOCX) [file pone.0168867.s002.docx]

<?xml version="1.0" encoding="UTF-8"?>

<!--

*************************************************

AnyLogic Project File

*************************************************

-->

<AnyLogicWorkspace WorkspaceVersion="1.9" AnyLogicVersion="7.0.3.201406021411" AlpVersion="7.0.2">

<Model>

<Id>1390440632826</Id>

<Name><![CDATA[15-03-01 Income by region]]></Name>

<EngineVersion>6</EngineVersion>

<JavaPackageName><![CDATA[_14_01_21_trial_uganda_model]]></JavaPackageName>

<ModelTimeUnit><![CDATA[Day]]></ModelTimeUnit>

<SystemOfMeasurement><![CDATA[METRIC]]></SystemOfMeasurement>

<ActiveObjectClasses>

<!-- ========= Active Object Class ======== -->

<ActiveObjectClass>

<Id>1390440632827</Id>

<Name><![CDATA[Main]]></Name>

<ClientAreaTopLeft><X>0</X><Y>0</Y></ClientAreaTopLeft>

<PresentationTopGroupPersistent>true</PresentationTopGroupPersistent>

<IconTopGroupPersistent>true</IconTopGroupPersistent>

<Import><![CDATA[import umontreal.iro.lecuyer.probdist.ContinuousDistribution;

import umontreal.iro.lecuyer.probdist.GammaDist;]]></Import>

<StartupCode><![CDATA[//Determine which policy lever is pulled and set parameters

/*

policy = count / nIter;

UPF = (policy == 1 || policy == 3 || policy == 4 || policy == 6) ? true : false;

TS = (policy == 2 || policy == 3 || policy == 5 || policy == 6) ? true : false;

vouchers = (policy == 4 || policy == 5 || policy == 6) ? true : false;

twoWeekTrips = (policy == 7) ? true : false;

mercyShips = (policy == 8) ? true : false;

cancerHospital = (policy == 9) ? true : false;

*/

vouchersMult = vouchers ? 0.0 : 1.0;

systemCost = 0;

OOP = UPF ? 0.0 : beta(100*0.493288938, 100*(1-0.493288938));

/*

//establish the cutoffs

double shape = urbanGini;

double scale = shape / (meanGDP * conversion);

cutoff1 = GammaDist.inverseF(shape, scale, 3, 0.2);

cutoff2 = GammaDist.inverseF(shape, scale, 3, 0.4);

cutoff3 = GammaDist.inverseF(shape, scale, 3, 0.6);

cutoff4 = GammaDist.inverseF(shape, scale, 3, 0.8);

povertyLine = GammaDist.inverseF(shape, scale, 3, povPct);

*/

/*

GammaDist.inverseF(alpha, lambda, d, u)

where:

alpha = shape = Gini

lambda = 1/beta = Gini/income

d = number of decimal places of tolerance

u = U[0,1]

*/

//establish network size

double villageRadius = placementRadius * 1609.34;

//add people within villageRadius of each other are connected

//note setNetworkAllInRange() uses meters returns in meters; hence the calculation above

setNetworkAllInRange(villageRadius);

//read life table data into LifeTable functions

cancerMort.readTableFunction(maleLifeTable,"Total Deaths!F3",18);

cancerMort.readTableFunction(femaleLifeTable,"Total Deaths!M3",18);

cancerMort.readTableFunction(maleBackoutMort,"Cancer Deaths!E3",18);

cancerMort.readTableFunction(femaleBackoutMort,"Cancer Deaths!L3",14);

ASRmortality.readTableFunction(maleInitLifeTable,"Country!N2",22);

ASRmortality.readTableFunction(femaleInitLifeTable,"Country!N24",22);

//set starting model speed

if(modelSpeed == 4) getEngine().setRealTimeMode(false);

else {

getEngine().setRealTimeMode(true);

switch(modelSpeed) {

case 3:

getEngine().setRealTimeScale(500);

break;

case 2:

getEngine().setRealTimeScale(100);

break;

case 1:

getEngine().setRealTimeScale(10);

break;

default:

getEngine().setRealTimeScale(1);

break;

}

}

//populate map

createHospitals();

createRegionals();

createDistricts();

createPeople(popSize);

]]></StartupCode>

<DestroyCode><![CDATA[/*

excelPersCost.writeDataSet(dsPersCost, 1, 2, 1);

excelPersCost.writeDataSet(dsCatExpPoorest, 1, 2, 3);

excelPersCost.writeDataSet(cancTxSize, 1, 2, 5);

excelPersCost.writeDataSet(cancIncSize, 1, 2, 7);

excelPersCost.setCellValue(meanQual, 1, 2, 11);

excelPersCost.writeDataSet(systCostDataset, 1, 2, 12);

excelPersCost.writeDataSet(popOutput, 1, 2, 15);

excelPersCost.writeDataSet(dsCancIncPoorest, 2, 2, 1);

excelPersCost.writeDataSet(dsCancIncPoor, 2, 2, 3);

excelPersCost.writeDataSet(dsCancIncMiddle, 2, 2, 5);

excelPersCost.writeDataSet(dsCancIncRich, 2, 2, 7);

excelPersCost.writeDataSet(dsCancIncRichest, 2, 2, 9);

excelPersCost.writeDataSet(dsCancDeathPoorest, 2, 2,11);

excelPersCost.writeDataSet(dsCancDeathPoor, 2, 2,13);

excelPersCost.writeDataSet(dsCancDeathMiddle, 2, 2,15);

excelPersCost.writeDataSet(dsCancDeathRich, 2, 2,17);

excelPersCost.writeDataSet(dsCancDeathRichest, 2, 2,19);

int a = 0;

if(cancerHospital) a = 4;

else if(TS) a = 2;

excelPersCost.writeDataSet(dsMadePoorPoorest, 3, 2, 1+a);

excelPersCost.writeDataSet(dsCancDeathPoorest, 4, 2, 1+a);

excelPersCost.writeDataSet(dsCancIncPoorest, 5, 2, 1+a);

excelPersCost.writeDataSet(popOutput, 5, 2, 7+a);

excelPersCost.evaluateFormulas();

*/]]></DestroyCode>

<Generic>false</Generic>

<GenericParameters><![CDATA[T]]></GenericParameters>

<GenericParametersLabel><![CDATA[Generic parameters:]]></GenericParametersLabel>

<FlowChartsUsage>ENTITY</FlowChartsUsage>

<SamplesToKeep>100</SamplesToKeep>

<LimitNumberOfArrayElements>false</LimitNumberOfArrayElements>

<ElementsLimitValue>100</ElementsLimitValue>

<MakeDefaultViewArea>true</MakeDefaultViewArea>

<SceneGridColor/>

<SceneBackgroundColor/>

<AgentProperties>

<SpaceType>CONTINUOUS</SpaceType>

<EnvironmentDefinesInitialLocation>true</EnvironmentDefinesInitialLocation>

<RotateAnimationTowardsMovement>true</RotateAnimationTowardsMovement>

<RotateAnimationVertically>false</RotateAnimationVertically>

<VelocityCode><![CDATA[10]]></VelocityCode>

<VelocityGISCode><![CDATA[10]]></VelocityGISCode>

</AgentProperties>

<EnvironmentProperties>

<EnableSteps>false</EnableSteps>

<StepDurationCode><![CDATA[1.0]]></StepDurationCode>

<SpaceType>GIS</SpaceType>

<WidthCode><![CDATA[500]]></WidthCode>

<HeightCode><![CDATA[500]]></HeightCode>

<ZHeightCode><![CDATA[0]]></ZHeightCode>

<ColumnsCountCode><![CDATA[100]]></ColumnsCountCode>

<RowsCountCode><![CDATA[100]]></RowsCountCode>

<NeigborhoodType>MOORE</NeigborhoodType>

<LayoutType>USER_DEF</LayoutType>

<LayoutTypeApplyOnStartup>true</LayoutTypeApplyOnStartup>

<NetworkType>USER_DEF</NetworkType>

<NetworkTypeApplyOnStartup>true</NetworkTypeApplyOnStartup>

<ConnectionsPerAgentCode><![CDATA[2]]></ConnectionsPerAgentCode>

<ConnectionsRangeCode><![CDATA[50]]></ConnectionsRangeCode>

<NeighborLinkFractionCode><![CDATA[0.95]]></NeighborLinkFractionCode>

<MCode><![CDATA[10]]></MCode>

<GisMapCode><![CDATA[Uganda]]></GisMapCode>

</EnvironmentProperties>

<DatasetsCreationProperties>

<AutoCreate>true</AutoCreate>

<RecurrenceCode><![CDATA[1]]></RecurrenceCode>

</DatasetsCreationProperties>

<ConnectionsId>1392508625677</ConnectionsId>

<Variables>

<Variable Class="PlainVariable">

<Id>1390791263289</Id>

<Name><![CDATA[polAreaName]]></Name>

<X>-150</X><Y>490</Y>

<Label><X>10</X><Y>0</Y></Label>

<PublicFlag>false</PublicFlag>

<PresentationFlag>false</PresentationFlag>

<ShowLabel>true</ShowLabel>

<Properties SaveInSnapshot="true" Constant="false" AccessType="public" StaticVariable="false">

<Type><![CDATA[String]]></Type>

<InitialValue><![CDATA["Click anywhere on the map"]]></InitialValue>

</Properties>

</Variable>

<Variable Class="PlainVariable">

<Id>1392239946559</Id>

<Name><![CDATA[selectedPerson]]></Name>

<X>-150</X><Y>250</Y>

<Label><X>10</X><Y>0</Y></Label>

<PublicFlag>false</PublicFlag>

<PresentationFlag>false</PresentationFlag>

<ShowLabel>true</ShowLabel>

<Properties SaveInSnapshot="true" Constant="false" AccessType="public" StaticVariable="false">

<Type><![CDATA[Person]]></Type>

</Properties>

</Variable>

<Variable Class="PlainVariable">

<Id>1392502405534</Id>

<Name><![CDATA[birthsPerK]]></Name>

<X>-150</X><Y>270</Y>

<Label><X>10</X><Y>0</Y></Label>

<PublicFlag>false</PublicFlag>

<PresentationFlag>false</PresentationFlag>

<ShowLabel>true</ShowLabel>

<Properties SaveInSnapshot="true" Constant="false" AccessType="public" StaticVariable="false">

<Type><![CDATA[double]]></Type>

<InitialValue><![CDATA[0]]></InitialValue>

</Properties>

</Variable>

<Variable Class="PlainVariable">

<Id>1392672219538</Id>

<Name><![CDATA[deathsPerK]]></Name>

<X>-150</X><Y>330</Y>

<Label><X>10</X><Y>0</Y></Label>

<PublicFlag>false</PublicFlag>

<PresentationFlag>false</PresentationFlag>

<ShowLabel>true</ShowLabel>

<Properties SaveInSnapshot="true" Constant="false" AccessType="public" StaticVariable="false">

<Type><![CDATA[double]]></Type>

<InitialValue><![CDATA[0.00000000]]></InitialValue>

</Properties>

</Variable>

<Variable Class="PlainVariable">

<Id>1392672697309</Id>

<Name><![CDATA[DeadThisYear]]></Name>

<X>-150</X><Y>310</Y>

<Label><X>10</X><Y>0</Y></Label>

<PublicFlag>false</PublicFlag>

<PresentationFlag>false</PresentationFlag>

<ShowLabel>true</ShowLabel>

<Properties SaveInSnapshot="true" Constant="false" AccessType="public" StaticVariable="false">

<Type><![CDATA[int]]></Type>

</Properties>

</Variable>

<Variable Class="PlainVariable">

<Id>1392673277341</Id>

<Name><![CDATA[nAlive]]></Name>

<X>-150</X><Y>350</Y>

<Label><X>10</X><Y>0</Y></Label>

<PublicFlag>false</PublicFlag>

<PresentationFlag>false</PresentationFlag>

<ShowLabel>true</ShowLabel>

<Properties SaveInSnapshot="true" Constant="false" AccessType="public" StaticVariable="false">

<Type><![CDATA[double]]></Type>

<InitialValue><![CDATA[persons.nAlive()]]></InitialValue>

</Properties>

</Variable>

<Variable Class="PlainVariable">

<Id>1392836641973</Id>

<Name><![CDATA[infantDeaths]]></Name>

<X>-150</X><Y>370</Y>

<Label><X>10</X><Y>0</Y></Label>

<PublicFlag>false</PublicFlag>

<PresentationFlag>false</PresentationFlag>

<ShowLabel>true</ShowLabel>

<Properties SaveInSnapshot="true" Constant="false" AccessType="public" StaticVariable="false">

<Type><![CDATA[int]]></Type>

</Properties>

</Variable>

<Variable Class="PlainVariable">

<Id>1392836682257</Id>

<Name><![CDATA[infantMortality]]></Name>

<X>-150</X><Y>290</Y>

<Label><X>10</X><Y>0</Y></Label>

<PublicFlag>false</PublicFlag>

<PresentationFlag>false</PresentationFlag>

<ShowLabel>true</ShowLabel>

<Properties SaveInSnapshot="true" Constant="false" AccessType="public" StaticVariable="false">

<Type><![CDATA[double]]></Type>

</Properties>

</Variable>

<Variable Class="PlainVariable">

<Id>1392836999363</Id>

<Name><![CDATA[liveBirths]]></Name>

<X>-150</X><Y>390</Y>

<Label><X>10</X><Y>0</Y></Label>

<PublicFlag>false</PublicFlag>

<PresentationFlag>false</PresentationFlag>

<ShowLabel>true</ShowLabel>

<Properties SaveInSnapshot="true" Constant="false" AccessType="public" StaticVariable="false">

<Type><![CDATA[int]]></Type>

</Properties>

</Variable>

<Variable Class="PlainVariable">

<Id>1410983756137</Id>

<Name><![CDATA[systemCost]]></Name>

<X>-830</X><Y>150</Y>

<Label><X>10</X><Y>0</Y></Label>

<PublicFlag>false</PublicFlag>

<PresentationFlag>false</PresentationFlag>

<ShowLabel>true</ShowLabel>

<Properties SaveInSnapshot="true" Constant="false" AccessType="public" StaticVariable="false">

<Type><![CDATA[double]]></Type>

</Properties>

</Variable>

<Variable Class="PlainVariable">

<Id>1411052405657</Id>

<Name><![CDATA[runCount]]></Name>

<X>-1650</X><Y>130</Y>

<Label><X>10</X><Y>0</Y></Label>

<PublicFlag>false</PublicFlag>

<PresentationFlag>false</PresentationFlag>

<ShowLabel>true</ShowLabel>

<Properties SaveInSnapshot="true" Constant="false" AccessType="public" StaticVariable="false">

<Type><![CDATA[int]]></Type>

</Properties>

</Variable>

<Variable Class="PlainVariable">

<Id>1411572368045</Id>

<Name><![CDATA[cancerDeathPoorest]]></Name>

<X>-1190</X><Y>160</Y>

<Label><X>10</X><Y>0</Y></Label>

<PublicFlag>false</PublicFlag>

<PresentationFlag>false</PresentationFlag>

<ShowLabel>true</ShowLabel>

<Properties SaveInSnapshot="true" Constant="false" AccessType="public" StaticVariable="false">

<Type><![CDATA[int]]></Type>

</Properties>

</Variable>

<Variable Class="PlainVariable">

<Id>1411575897493</Id>

<Name><![CDATA[cancerDeathRichest]]></Name>

<X>-1190</X><Y>240</Y>

<Label><X>10</X><Y>0</Y></Label>

<PublicFlag>false</PublicFlag>

<PresentationFlag>false</PresentationFlag>

<ShowLabel>true</ShowLabel>

<Properties SaveInSnapshot="true" Constant="false" AccessType="public" StaticVariable="false">

<Type><![CDATA[int]]></Type>

</Properties>

</Variable>

<Variable Class="PlainVariable">

<Id>1411575898205</Id>

<Name><![CDATA[cancerDeathRich]]></Name>

<X>-1190</X><Y>220</Y>

<Label><X>10</X><Y>0</Y></Label>

<PublicFlag>false</PublicFlag>

<PresentationFlag>false</PresentationFlag>

<ShowLabel>true</ShowLabel>

<Properties SaveInSnapshot="true" Constant="false" AccessType="public" StaticVariable="false">

<Type><![CDATA[int]]></Type>

</Properties>

</Variable>

<Variable Class="PlainVariable">

<Id>1411575898538</Id>

<Name><![CDATA[cancerDeathMiddle]]></Name>

<X>-1190</X><Y>200</Y>

<Label><X>10</X><Y>0</Y></Label>

<PublicFlag>false</PublicFlag>

<PresentationFlag>false</PresentationFlag>

<ShowLabel>true</ShowLabel>

<Properties SaveInSnapshot="true" Constant="false" AccessType="public" StaticVariable="false">

<Type><![CDATA[int]]></Type>

</Properties>

</Variable>

<Variable Class="PlainVariable">

<Id>1411575899057</Id>

<Name><![CDATA[cancerDeathPoor]]></Name>

<X>-1190</X><Y>180</Y>

<Label><X>10</X><Y>0</Y></Label>

<PublicFlag>false</PublicFlag>

<PresentationFlag>false</PresentationFlag>

<ShowLabel>true</ShowLabel>

<Properties SaveInSnapshot="true" Constant="false" AccessType="public" StaticVariable="false">

<Type><![CDATA[int]]></Type>

</Properties>

</Variable>

<Variable Class="PlainVariable">

<Id>1411582365429</Id>

<Name><![CDATA[madePoorPoorest]]></Name>

<X>-1330</X><Y>160</Y>

<Label><X>10</X><Y>0</Y></Label>

<PublicFlag>false</PublicFlag>

<PresentationFlag>false</PresentationFlag>

<ShowLabel>true</ShowLabel>

<Properties SaveInSnapshot="true" Constant="false" AccessType="public" StaticVariable="false">

<Type><![CDATA[int]]></Type>

</Properties>

</Variable>

<Variable Class="PlainVariable">

<Id>1411582365431</Id>

<Name><![CDATA[madePoorRichest]]></Name>

<X>-1330</X><Y>240</Y>

<Label><X>10</X><Y>0</Y></Label>

<PublicFlag>false</PublicFlag>

<PresentationFlag>false</PresentationFlag>

<ShowLabel>true</ShowLabel>

<Properties SaveInSnapshot="true" Constant="false" AccessType="public" StaticVariable="false">

<Type><![CDATA[int]]></Type>

</Properties>

</Variable>

<Variable Class="PlainVariable">

<Id>1411582365433</Id>

<Name><![CDATA[madePoorRich]]></Name>

<X>-1330</X><Y>220</Y>

<Label><X>10</X><Y>0</Y></Label>

<PublicFlag>false</PublicFlag>

<PresentationFlag>false</PresentationFlag>

<ShowLabel>true</ShowLabel>

<Properties SaveInSnapshot="true" Constant="false" AccessType="public" StaticVariable="false">

<Type><![CDATA[int]]></Type>

</Properties>

</Variable>

<Variable Class="PlainVariable">

<Id>1411582365435</Id>

<Name><![CDATA[madePoorMiddle]]></Name>

<X>-1330</X><Y>200</Y>

<Label><X>10</X><Y>0</Y></Label>

<PublicFlag>false</PublicFlag>

<PresentationFlag>false</PresentationFlag>

<ShowLabel>true</ShowLabel>

<Properties SaveInSnapshot="true" Constant="false" AccessType="public" StaticVariable="false">

<Type><![CDATA[int]]></Type>

</Properties>

</Variable>

<Variable Class="PlainVariable">

<Id>1411582365437</Id>

<Name><![CDATA[madePoorPoor]]></Name>

<X>-1330</X><Y>180</Y>

<Label><X>10</X><Y>0</Y></Label>

<PublicFlag>false</PublicFlag>

<PresentationFlag>false</PresentationFlag>

<ShowLabel>true</ShowLabel>

<Properties SaveInSnapshot="true" Constant="false" AccessType="public" StaticVariable="false">

<Type><![CDATA[int]]></Type>

</Properties>

</Variable>

<Variable Class="PlainVariable">

<Id>1411926255714</Id>

<Name><![CDATA[madePoorNewPoorest]]></Name>

<X>-1510</X><Y>370</Y>

<Label><X>10</X><Y>0</Y></Label>

<PublicFlag>false</PublicFlag>

<PresentationFlag>false</PresentationFlag>

<ShowLabel>true</ShowLabel>

<Properties SaveInSnapshot="true" Constant="false" AccessType="public" StaticVariable="false">

<Type><![CDATA[int]]></Type>

</Properties>

</Variable>

<Variable Class="PlainVariable">

<Id>1411926255719</Id>

<Name><![CDATA[madePoorNewRichest]]></Name>

<X>-1510</X><Y>450</Y>

<Label><X>10</X><Y>0</Y></Label>

<PublicFlag>false</PublicFlag>

<PresentationFlag>false</PresentationFlag>

<ShowLabel>true</ShowLabel>

<Properties SaveInSnapshot="true" Constant="false" AccessType="public" StaticVariable="false">

<Type><![CDATA[int]]></Type>

</Properties>

</Variable>

<Variable Class="PlainVariable">

<Id>1411926255721</Id>

<Name><![CDATA[madePoorNewRich]]></Name>

<X>-1510</X><Y>430</Y>

<Label><X>10</X><Y>0</Y></Label>

<PublicFlag>false</PublicFlag>

<PresentationFlag>false</PresentationFlag>

<ShowLabel>true</ShowLabel>

<Properties SaveInSnapshot="true" Constant="false" AccessType="public" StaticVariable="false">

<Type><![CDATA[int]]></Type>

</Properties>

</Variable>

<Variable Class="PlainVariable">

<Id>1411926255723</Id>

<Name><![CDATA[madePoorNewMiddle]]></Name>

<X>-1510</X><Y>410</Y>

<Label><X>10</X><Y>0</Y></Label>

<PublicFlag>false</PublicFlag>

<PresentationFlag>false</PresentationFlag>

<ShowLabel>true</ShowLabel>

<Properties SaveInSnapshot="true" Constant="false" AccessType="public" StaticVariable="false">

<Type><![CDATA[int]]></Type>

</Properties>

</Variable>

<Variable Class="PlainVariable">

<Id>1411926255725</Id>

<Name><![CDATA[madePoorNewPoor]]></Name>

<X>-1510</X><Y>390</Y>

<Label><X>10</X><Y>0</Y></Label>

<PublicFlag>false</PublicFlag>

<PresentationFlag>false</PresentationFlag>

<ShowLabel>true</ShowLabel>

<Properties SaveInSnapshot="true" Constant="false" AccessType="public" StaticVariable="false">

<Type><![CDATA[int]]></Type>

</Properties>

</Variable>

<Variable Class="PlainVariable">

<Id>1411926255727</Id>

<Name><![CDATA[catExpPoorest]]></Name>

<X>-1650</X><Y>370</Y>

<Label><X>10</X><Y>0</Y></Label>

<PublicFlag>false</PublicFlag>

<PresentationFlag>false</PresentationFlag>

<ShowLabel>true</ShowLabel>

<Properties SaveInSnapshot="true" Constant="false" AccessType="public" StaticVariable="false">

<Type><![CDATA[int]]></Type>

</Properties>

</Variable>

<Variable Class="PlainVariable">

<Id>1411926255729</Id>

<Name><![CDATA[catExpRichest]]></Name>

<X>-1650</X><Y>450</Y>

<Label><X>10</X><Y>0</Y></Label>

<PublicFlag>false</PublicFlag>

<PresentationFlag>false</PresentationFlag>

<ShowLabel>true</ShowLabel>

<Properties SaveInSnapshot="true" Constant="false" AccessType="public" StaticVariable="false">

<Type><![CDATA[int]]></Type>

</Properties>

</Variable>

<Variable Class="PlainVariable">

<Id>1411926255731</Id>

<Name><![CDATA[catExpRich]]></Name>

<X>-1650</X><Y>430</Y>

<Label><X>10</X><Y>0</Y></Label>

<PublicFlag>false</PublicFlag>

<PresentationFlag>false</PresentationFlag>

<ShowLabel>true</ShowLabel>

<Properties SaveInSnapshot="true" Constant="false" AccessType="public" StaticVariable="false">

<Type><![CDATA[int]]></Type>

</Properties>

</Variable>

<Variable Class="PlainVariable">

<Id>1411926255733</Id>

<Name><![CDATA[catExpMiddle]]></Name>

<X>-1650</X><Y>410</Y>

<Label><X>10</X><Y>0</Y></Label>

<PublicFlag>false</PublicFlag>

<PresentationFlag>false</PresentationFlag>

<ShowLabel>true</ShowLabel>

<Properties SaveInSnapshot="true" Constant="false" AccessType="public" StaticVariable="false">

<Type><![CDATA[int]]></Type>

</Properties>

</Variable>

<Variable Class="PlainVariable">

<Id>1411926255735</Id>

<Name><![CDATA[catExpPoor]]></Name>

<X>-1650</X><Y>390</Y>

<Label><X>10</X><Y>0</Y></Label>

<PublicFlag>false</PublicFlag>

<PresentationFlag>false</PresentationFlag>

<ShowLabel>true</ShowLabel>

<Properties SaveInSnapshot="true" Constant="false" AccessType="public" StaticVariable="false">

<Type><![CDATA[int]]></Type>

</Properties>

</Variable>

<Variable Class="PlainVariable">

<Id>1412104712469</Id>

<Name><![CDATA[cancTxPoorest]]></Name>

<X>-1510</X><Y>160</Y>

<Label><X>10</X><Y>0</Y></Label>

<PublicFlag>false</PublicFlag>

<PresentationFlag>false</PresentationFlag>

<ShowLabel>true</ShowLabel>

<Properties SaveInSnapshot="true" Constant="false" AccessType="public" StaticVariable="false">

<Type><![CDATA[int]]></Type>

</Properties>

</Variable>

<Variable Class="PlainVariable">

<Id>1412104712474</Id>

<Name><![CDATA[cancTxRichest]]></Name>

<X>-1510</X><Y>240</Y>

<Label><X>10</X><Y>0</Y></Label>

<PublicFlag>false</PublicFlag>

<PresentationFlag>false</PresentationFlag>

<ShowLabel>true</ShowLabel>

<Properties SaveInSnapshot="true" Constant="false" AccessType="public" StaticVariable="false">

<Type><![CDATA[int]]></Type>

</Properties>

</Variable>

<Variable Class="PlainVariable">

<Id>1412104712476</Id>

<Name><![CDATA[cancTxRich]]></Name>

<X>-1510</X><Y>220</Y>

<Label><X>10</X><Y>0</Y></Label>

<PublicFlag>false</PublicFlag>

<PresentationFlag>false</PresentationFlag>

<ShowLabel>true</ShowLabel>

<Properties SaveInSnapshot="true" Constant="false" AccessType="public" StaticVariable="false">

<Type><![CDATA[int]]></Type>

</Properties>

</Variable>

<Variable Class="PlainVariable">

<Id>1412104712478</Id>

<Name><![CDATA[cancTxMiddle]]></Name>

<X>-1510</X><Y>200</Y>

<Label><X>10</X><Y>0</Y></Label>

<PublicFlag>false</PublicFlag>

<PresentationFlag>false</PresentationFlag>

<ShowLabel>true</ShowLabel>

<Properties SaveInSnapshot="true" Constant="false" AccessType="public" StaticVariable="false">

<Type><![CDATA[int]]></Type>

</Properties>

</Variable>

<Variable Class="PlainVariable">

<Id>1412104712480</Id>

<Name><![CDATA[cancTxPoor]]></Name>

<X>-1510</X><Y>180</Y>

<Label><X>10</X><Y>0</Y></Label>

<PublicFlag>false</PublicFlag>

<PresentationFlag>false</PresentationFlag>

<ShowLabel>true</ShowLabel>

<Properties SaveInSnapshot="true" Constant="false" AccessType="public" StaticVariable="false">

<Type><![CDATA[int]]></Type>

</Properties>

</Variable>

<Variable Class="PlainVariable">

<Id>1412104712482</Id>

<Name><![CDATA[cancIncPoorest]]></Name>

<X>-1650</X><Y>160</Y>

<Label><X>10</X><Y>0</Y></Label>

<PublicFlag>false</PublicFlag>

<PresentationFlag>false</PresentationFlag>

<ShowLabel>true</ShowLabel>

<Properties SaveInSnapshot="true" Constant="false" AccessType="public" StaticVariable="false">

<Type><![CDATA[int]]></Type>

</Properties>

</Variable>

<Variable Class="PlainVariable">

<Id>1412104712484</Id>

<Name><![CDATA[cancIncRichest]]></Name>

<X>-1650</X><Y>240</Y>

<Label><X>10</X><Y>0</Y></Label>

<PublicFlag>false</PublicFlag>

<PresentationFlag>false</PresentationFlag>

<ShowLabel>true</ShowLabel>

<Properties SaveInSnapshot="true" Constant="false" AccessType="public" StaticVariable="false">

<Type><![CDATA[int]]></Type>

</Properties>

</Variable>

<Variable Class="PlainVariable">

<Id>1412104712486</Id>

<Name><![CDATA[cancIncRich]]></Name>

<X>-1650</X><Y>220</Y>

<Label><X>10</X><Y>0</Y></Label>

<PublicFlag>false</PublicFlag>

<PresentationFlag>false</PresentationFlag>

<ShowLabel>true</ShowLabel>

<Properties SaveInSnapshot="true" Constant="false" AccessType="public" StaticVariable="false">

<Type><![CDATA[int]]></Type>

</Properties>

</Variable>

<Variable Class="PlainVariable">

<Id>1412104712488</Id>

<Name><![CDATA[cancIncMiddle]]></Name>

<X>-1650</X><Y>200</Y>

<Label><X>10</X><Y>0</Y></Label>

<PublicFlag>false</PublicFlag>

<PresentationFlag>false</PresentationFlag>

<ShowLabel>true</ShowLabel>

<Properties SaveInSnapshot="true" Constant="false" AccessType="public" StaticVariable="false">

<Type><![CDATA[int]]></Type>

</Properties>

</Variable>

<Variable Class="PlainVariable">

<Id>1412104712490</Id>

<Name><![CDATA[cancIncPoor]]></Name>

<X>-1650</X><Y>180</Y>

<Label><X>10</X><Y>0</Y></Label>

<PublicFlag>false</PublicFlag>

<PresentationFlag>false</PresentationFlag>

<ShowLabel>true</ShowLabel>

<Properties SaveInSnapshot="true" Constant="false" AccessType="public" StaticVariable="false">

<Type><![CDATA[int]]></Type>

</Properties>

</Variable>

<Variable Class="PlainVariable">

<Id>1412805904587</Id>

<Name><![CDATA[persCost]]></Name>

<X>-830</X><Y>170</Y>

<Label><X>10</X><Y>0</Y></Label>

<PublicFlag>false</PublicFlag>

<PresentationFlag>true</PresentationFlag>

<ShowLabel>true</ShowLabel>

<Properties SaveInSnapshot="true" Constant="false" AccessType="public" StaticVariable="false">

<Type><![CDATA[double]]></Type>

</Properties>

</Variable>

<Variable Class="PlainVariable">

<Id>1412878908470</Id>

<Name><![CDATA[pop2050]]></Name>

<X>-410</X><Y>210</Y>

<Label><X>10</X><Y>0</Y></Label>

<PublicFlag>false</PublicFlag>

<PresentationFlag>true</PresentationFlag>

<ShowLabel>true</ShowLabel>

<Properties SaveInSnapshot="true" Constant="false" AccessType="public" StaticVariable="false">

<Type><![CDATA[double]]></Type>

</Properties>

</Variable>

<Variable Class="PlainVariable">

<Id>1425334552724</Id>

<Name><![CDATA[ngoSurgeries]]></Name>

<X>-1330</X><Y>380</Y>

<Label><X>10</X><Y>0</Y></Label>

<PublicFlag>false</PublicFlag>

<PresentationFlag>false</PresentationFlag>

<ShowLabel>true</ShowLabel>

<Properties SaveInSnapshot="true" Constant="false" AccessType="public" StaticVariable="false">

<Type><![CDATA[int]]></Type>

</Properties>

</Variable>

<Variable Class="Parameter">

<Id>1390776969990</Id>

<Name><![CDATA[popSize]]></Name>

<X>-500</X><Y>50</Y>

<Label><X>10</X><Y>0</Y></Label>

<PublicFlag>false</PublicFlag>

<PresentationFlag>false</PresentationFlag>

<ShowLabel>true</ShowLabel>

<Properties SaveInSnapshot="true" ModificatorType="STATIC">

<Type><![CDATA[int]]></Type>

<SdArray>false</SdArray>

<DefaultValue><![CDATA[1000]]></DefaultValue>

<ParameterEditor>

<Id>1390776969988</Id>

<Name><![CDATA[]]></Name>

<EditorContolType>TEXT_BOX</EditorContolType>

<MinSliderValue><![CDATA[0]]></MinSliderValue>

<MaxSliderValue><![CDATA[100]]></MaxSliderValue>

<DelimeterType>NO_DELIMETER</DelimeterType>

</ParameterEditor>

</Properties>

</Variable>

<Variable Class="Parameter">

<Id>1390791986472</Id>

<Name><![CDATA[displayDistNames]]></Name>

<X>-700</X><Y>90</Y>

<Label><X>10</X><Y>0</Y></Label>

<PublicFlag>false</PublicFlag>

<PresentationFlag>false</PresentationFlag>

<ShowLabel>true</ShowLabel>

<Properties SaveInSnapshot="true" ModificatorType="STATIC">

<Type><![CDATA[boolean]]></Type>

<SdArray>false</SdArray>

<DefaultValue><![CDATA[false]]></DefaultValue>

<ParameterEditor>

<Id>1390791986470</Id>

<Name><![CDATA[]]></Name>

<EditorContolType>CHECK_BOX</EditorContolType>

<MinSliderValue><![CDATA[0]]></MinSliderValue>

<MaxSliderValue><![CDATA[100]]></MaxSliderValue>

<DelimeterType>NO_DELIMETER</DelimeterType>

</ParameterEditor>

</Properties>

</Variable>

<Variable Class="Parameter">

<Id>1390792495689</Id>

<Name><![CDATA[displayRegNames]]></Name>

<X>-700</X><Y>70</Y>

<Label><X>10</X><Y>0</Y></Label>

<PublicFlag>false</PublicFlag>

<PresentationFlag>false</PresentationFlag>

<ShowLabel>true</ShowLabel>

<Properties SaveInSnapshot="true" ModificatorType="STATIC">

<Type><![CDATA[boolean]]></Type>

<SdArray>false</SdArray>

<DefaultValue><![CDATA[false]]></DefaultValue>

<ParameterEditor>

<Id>1390792495687</Id>

<Name><![CDATA[]]></Name>

<EditorContolType>CHECK_BOX</EditorContolType>

<MinSliderValue><![CDATA[0]]></MinSliderValue>

<MaxSliderValue><![CDATA[100]]></MaxSliderValue>

<DelimeterType>NO_DELIMETER</DelimeterType>

</ParameterEditor>

</Properties>

</Variable>

<Variable Class="Parameter">

<Id>1390792496073</Id>

<Name><![CDATA[displayNatNames]]></Name>

<X>-700</X><Y>50</Y>

<Label><X>10</X><Y>0</Y></Label>

<PublicFlag>false</PublicFlag>

<PresentationFlag>false</PresentationFlag>

<ShowLabel>true</ShowLabel>

<Properties SaveInSnapshot="true" ModificatorType="STATIC">

<Type><![CDATA[boolean]]></Type>

<SdArray>false</SdArray>

<DefaultValue><![CDATA[false]]></DefaultValue>

<ParameterEditor>

<Id>1390792496071</Id>

<Name><![CDATA[]]></Name>

<EditorContolType>CHECK_BOX</EditorContolType>

<MinSliderValue><![CDATA[0]]></MinSliderValue>

<MaxSliderValue><![CDATA[100]]></MaxSliderValue>

<DelimeterType>NO_DELIMETER</DelimeterType>

</ParameterEditor>

</Properties>

</Variable>

<Variable Class="Parameter">

<Id>1390864737762</Id>

<Name><![CDATA[placementRadius]]></Name>

<Description><![CDATA[Radius, in miles, from the center point of the GPS location chosen for the individual patient.

That is, if the patient gets allotted to (X,Y), the createPeople() function will actually place him randomly around that point. The actual location

he gets placed will be (X+d, Y+e) where d,e ~ N(0,sigma). Sigma is chosen such that 95% of patients fall within the radius set here.]]></Description>

<X>-500</X><Y>70</Y>

<Label><X>10</X><Y>0</Y></Label>

<PublicFlag>false</PublicFlag>

<PresentationFlag>false</PresentationFlag>

<ShowLabel>true</ShowLabel>

<Properties SaveInSnapshot="true" ModificatorType="STATIC">

<Type><![CDATA[int]]></Type>

<SdArray>false</SdArray>

<DefaultValue><![CDATA[5]]></DefaultValue>

<ParameterEditor>

<Id>1390864737760</Id>

<Name><![CDATA[]]></Name>

<EditorContolType>TEXT_BOX</EditorContolType>

<MinSliderValue><![CDATA[0]]></MinSliderValue>

<MaxSliderValue><![CDATA[100]]></MaxSliderValue>

<DelimeterType>NO_DELIMETER</DelimeterType>

</ParameterEditor>

</Properties>

</Variable>

<Variable Class="Parameter">

<Id>1391101309820</Id>

<Name><![CDATA[propKampala]]></Name>

<X>-500</X><Y>110</Y>

<Label><X>10</X><Y>0</Y></Label>

<PublicFlag>false</PublicFlag>

<PresentationFlag>false</PresentationFlag>

<ShowLabel>true</ShowLabel>

<Properties SaveInSnapshot="true" ModificatorType="STATIC">

<Type><![CDATA[double]]></Type>

<SdArray>false</SdArray>

<DefaultValue><![CDATA[0.05]]></DefaultValue>

<ParameterEditor>

<Id>1391101309818</Id>

<Name><![CDATA[]]></Name>

<EditorContolType>TEXT_BOX</EditorContolType>

<MinSliderValue><![CDATA[0]]></MinSliderValue>

<MaxSliderValue><![CDATA[100]]></MaxSliderValue>

<DelimeterType>NO_DELIMETER</DelimeterType>

</ParameterEditor>

</Properties>

</Variable>

<Variable Class="Parameter">

<Id>1391101325507</Id>

<Name><![CDATA[propRural]]></Name>

<X>-500</X><Y>130</Y>

<Label><X>10</X><Y>0</Y></Label>

<PublicFlag>false</PublicFlag>

<PresentationFlag>false</PresentationFlag>

<ShowLabel>true</ShowLabel>

<Properties SaveInSnapshot="true" ModificatorType="STATIC">

<Type><![CDATA[double]]></Type>

<SdArray>false</SdArray>

<DefaultValue><![CDATA[0.853010735]]></DefaultValue>

<ParameterEditor>

<Id>1391101325505</Id>

<Name><![CDATA[]]></Name>

<EditorContolType>TEXT_BOX</EditorContolType>

<MinSliderValue><![CDATA[0]]></MinSliderValue>

<MaxSliderValue><![CDATA[100]]></MaxSliderValue>

<DelimeterType>NO_DELIMETER</DelimeterType>

</ParameterEditor>

</Properties>

</Variable>

<Variable Class="Parameter">

<Id>1391101344893</Id>

<Name><![CDATA[propUrban]]></Name>

<X>-500</X><Y>150</Y>

<Label><X>10</X><Y>0</Y></Label>

<PublicFlag>false</PublicFlag>

<PresentationFlag>false</PresentationFlag>

<ShowLabel>true</ShowLabel>

<Properties SaveInSnapshot="true" ModificatorType="STATIC">

<Type><![CDATA[double]]></Type>

<SdArray>false</SdArray>

<DefaultValue><![CDATA[1.0 - propKampala - propRural]]></DefaultValue>

<ParameterEditor>

<Id>1391101344891</Id>

<Name><![CDATA[]]></Name>

<EditorContolType>TEXT_BOX</EditorContolType>

<MinSliderValue><![CDATA[0]]></MinSliderValue>

<MaxSliderValue><![CDATA[100]]></MaxSliderValue>

<DelimeterType>NO_DELIMETER</DelimeterType>

</ParameterEditor>

</Properties>

</Variable>

<Variable Class="Parameter">

<Id>1391138264899</Id>

<Name><![CDATA[transparency]]></Name>

<Description><![CDATA[Sets the transparency of the individual population based on the population size, such that the bigger the population, the more transparent any one individual is]]></Description>

<X>-700</X><Y>110</Y>

<Label><X>10</X><Y>0</Y></Label>

<PublicFlag>false</PublicFlag>

<PresentationFlag>false</PresentationFlag>

<ShowLabel>true</ShowLabel>

<Properties SaveInSnapshot="true" ModificatorType="STATIC">

<Type><![CDATA[int]]></Type>

<SdArray>false</SdArray>

<DefaultValue><![CDATA[min(255,max(45,255 - roundToInt(255*popSize/50000)))]]></DefaultValue>

<ParameterEditor>

<Id>1391138264897</Id>

<Name><![CDATA[]]></Name>

<EditorContolType>TEXT_BOX</EditorContolType>

<MinSliderValue><![CDATA[0]]></MinSliderValue>

<MaxSliderValue><![CDATA[100]]></MaxSliderValue>

<DelimeterType>NO_DELIMETER</DelimeterType>

</ParameterEditor>

</Properties>

</Variable>

<Variable Class="Parameter">

<Id>1391626801499</Id>

<Name><![CDATA[modelSpeed]]></Name>

<X>-500</X><Y>90</Y>

<Label><X>10</X><Y>0</Y></Label>

<PublicFlag>false</PublicFlag>

<PresentationFlag>false</PresentationFlag>

<ShowLabel>true</ShowLabel>

<Properties SaveInSnapshot="true" ModificatorType="STATIC">

<Type><![CDATA[int]]></Type>

<SdArray>false</SdArray>

<DefaultValue><![CDATA[4]]></DefaultValue>

<ParameterEditor>

<Id>1391626801497</Id>

<Name><![CDATA[]]></Name>

<EditorContolType>TEXT_BOX</EditorContolType>

<MinSliderValue><![CDATA[0]]></MinSliderValue>

<MaxSliderValue><![CDATA[100]]></MaxSliderValue>

<DelimeterType>NO_DELIMETER</DelimeterType>

</ParameterEditor>

</Properties>

</Variable>

<Variable Class="Parameter">

<Id>1392243172897</Id>

<Name><![CDATA[propKids]]></Name>

<Description><![CDATA[Initial proportion of under5s in Uganda]]></Description>

<X>-500</X><Y>170</Y>

<Label><X>10</X><Y>0</Y></Label>

<PublicFlag>false</PublicFlag>

<PresentationFlag>false</PresentationFlag>

<ShowLabel>true</ShowLabel>

<Properties SaveInSnapshot="true" ModificatorType="STATIC">

<Type><![CDATA[double]]></Type>

<SdArray>false</SdArray>

<DefaultValue><![CDATA[0.489]]></DefaultValue>

<ParameterEditor>

<Id>1392243172895</Id>

<Name><![CDATA[]]></Name>

<EditorContolType>TEXT_BOX</EditorContolType>

<MinSliderValue><![CDATA[0]]></MinSliderValue>

<MaxSliderValue><![CDATA[100]]></MaxSliderValue>

<DelimeterType>NO_DELIMETER</DelimeterType>

</ParameterEditor>

</Properties>

</Variable>

<Variable Class="Parameter">

<Id>1392247347605</Id>

<Name><![CDATA[propU5]]></Name>

<X>-500</X><Y>190</Y>

<Label><X>10</X><Y>0</Y></Label>

<PublicFlag>false</PublicFlag>

<PresentationFlag>false</PresentationFlag>

<ShowLabel>true</ShowLabel>

<Properties SaveInSnapshot="true" ModificatorType="STATIC">

<Type><![CDATA[double]]></Type>

<SdArray>false</SdArray>

<DefaultValue><![CDATA[0.192355617]]></DefaultValue>

<ParameterEditor>

<Id>1392247347603</Id>

<Name><![CDATA[]]></Name>

<EditorContolType>TEXT_BOX</EditorContolType>

<MinSliderValue><![CDATA[0]]></MinSliderValue>

<MaxSliderValue><![CDATA[100]]></MaxSliderValue>

<DelimeterType>NO_DELIMETER</DelimeterType>

</ParameterEditor>

</Properties>

</Variable>

<Variable Class="Parameter">

<Id>1392676148989</Id>

<Name><![CDATA[TFRAdjust]]></Name>

<X>-300</X><Y>40</Y>

<Label><X>10</X><Y>0</Y></Label>

<PublicFlag>false</PublicFlag>

<PresentationFlag>false</PresentationFlag>

<ShowLabel>true</ShowLabel>

<Properties SaveInSnapshot="true" ModificatorType="STATIC">

<Type><![CDATA[double]]></Type>

<SdArray>false</SdArray>

<DefaultValue><![CDATA[1]]></DefaultValue>

<ParameterEditor>

<Id>1392676148987</Id>

<Name><![CDATA[]]></Name>

<EditorContolType>TEXT_BOX</EditorContolType>

<MinSliderValue><![CDATA[0]]></MinSliderValue>

<MaxSliderValue><![CDATA[100]]></MaxSliderValue>

<DelimeterType>NO_DELIMETER</DelimeterType>

</ParameterEditor>

</Properties>

</Variable>

<Variable Class="Parameter">

<Id>1392676156776</Id>

<Name><![CDATA[deathAdjust]]></Name>

<X>-300</X><Y>60</Y>

<Label><X>10</X><Y>0</Y></Label>

<PublicFlag>false</PublicFlag>

<PresentationFlag>false</PresentationFlag>

<ShowLabel>true</ShowLabel>

<Properties SaveInSnapshot="true" ModificatorType="STATIC">

<Type><![CDATA[double]]></Type>

<SdArray>false</SdArray>

<DefaultValue><![CDATA[1]]></DefaultValue>

<ParameterEditor>

<Id>1392676156774</Id>

<Name><![CDATA[]]></Name>

<EditorContolType>TEXT_BOX</EditorContolType>

<MinSliderValue><![CDATA[0]]></MinSliderValue>

<MaxSliderValue><![CDATA[100]]></MaxSliderValue>

<DelimeterType>NO_DELIMETER</DelimeterType>

</ParameterEditor>

</Properties>

</Variable>

<Variable Class="Parameter">

<Id>1392827074396</Id>

<Name><![CDATA[adjust]]></Name>

<Description><![CDATA[Adjusts population size to thousands]]></Description>

<X>-410</X><Y>50</Y>

<Label><X>10</X><Y>0</Y></Label>

<PublicFlag>false</PublicFlag>

<PresentationFlag>false</PresentationFlag>

<ShowLabel>true</ShowLabel>

<Properties SaveInSnapshot="true" ModificatorType="STATIC">

<Type><![CDATA[double]]></Type>

<SdArray>false</SdArray>

<DefaultValue><![CDATA[(double) popSize / 34000]]></DefaultValue>

<ParameterEditor>

<Id>1392827074394</Id>

<Name><![CDATA[]]></Name>

<EditorContolType>TEXT_BOX</EditorContolType>

<MinSliderValue><![CDATA[0]]></MinSliderValue>

<MaxSliderValue><![CDATA[100]]></MaxSliderValue>

<DelimeterType>NO_DELIMETER</DelimeterType>

</ParameterEditor>

</Properties>

</Variable>

<Variable Class="Parameter">

<Id>1393271839896</Id>

<Name><![CDATA[urbanGDP]]></Name>

<X>-700</X><Y>170</Y>

<Label><X>10</X><Y>0</Y></Label>

<PublicFlag>false</PublicFlag>

<PresentationFlag>false</PresentationFlag>

<ShowLabel>true</ShowLabel>

<Properties SaveInSnapshot="true" ModificatorType="STATIC">

<Type><![CDATA[double]]></Type>

<SdArray>false</SdArray>

<DefaultValue><![CDATA[660200]]></DefaultValue>

<ParameterEditor>

<Id>1393271839894</Id>

<Name><![CDATA[]]></Name>

<EditorContolType>TEXT_BOX</EditorContolType>

<MinSliderValue><![CDATA[0]]></MinSliderValue>

<MaxSliderValue><![CDATA[100]]></MaxSliderValue>

<DelimeterType>NO_DELIMETER</DelimeterType>

</ParameterEditor>

</Properties>

</Variable>

<Variable Class="Parameter">

<Id>1393271855080</Id>

<Name><![CDATA[ruralGDP]]></Name>

<X>-700</X><Y>190</Y>

<Label><X>10</X><Y>0</Y></Label>

<PublicFlag>false</PublicFlag>

<PresentationFlag>false</PresentationFlag>

<ShowLabel>true</ShowLabel>

<Properties SaveInSnapshot="true" ModificatorType="STATIC">

<Type><![CDATA[double]]></Type>

<SdArray>false</SdArray>

<DefaultValue><![CDATA[222600]]></DefaultValue>

<ParameterEditor>

<Id>1393271855078</Id>

<Name><![CDATA[]]></Name>

<EditorContolType>TEXT_BOX</EditorContolType>

<MinSliderValue><![CDATA[0]]></MinSliderValue>

<MaxSliderValue><![CDATA[100]]></MaxSliderValue>

<DelimeterType>NO_DELIMETER</DelimeterType>

</ParameterEditor>

</Properties>

</Variable>

<Variable Class="Parameter">

<Id>1393271861595</Id>

<Name><![CDATA[urbanGini]]></Name>

<X>-700</X><Y>230</Y>

<Label><X>10</X><Y>0</Y></Label>

<PublicFlag>false</PublicFlag>

<PresentationFlag>false</PresentationFlag>

<ShowLabel>true</ShowLabel>

<Properties SaveInSnapshot="true" ModificatorType="STATIC">

<Type><![CDATA[double]]></Type>

<SdArray>false</SdArray>

<DefaultValue><![CDATA[4.26]]></DefaultValue>

<ParameterEditor>

<Id>1393271861593</Id>

<Name><![CDATA[]]></Name>

<EditorContolType>TEXT_BOX</EditorContolType>

<MinSliderValue><![CDATA[0]]></MinSliderValue>

<MaxSliderValue><![CDATA[100]]></MaxSliderValue>

<DelimeterType>NO_DELIMETER</DelimeterType>

</ParameterEditor>

</Properties>

</Variable>

<Variable Class="Parameter">

<Id>1393271868412</Id>

<Name><![CDATA[ruralGini]]></Name>

<X>-700</X><Y>250</Y>

<Label><X>10</X><Y>0</Y></Label>

<PublicFlag>false</PublicFlag>

<PresentationFlag>false</PresentationFlag>

<ShowLabel>true</ShowLabel>

<Properties SaveInSnapshot="true" ModificatorType="STATIC">

<Type><![CDATA[double]]></Type>

<SdArray>false</SdArray>

<DefaultValue><![CDATA[4.26]]></DefaultValue>

<ParameterEditor>

<Id>1393271868410</Id>

<Name><![CDATA[]]></Name>

<EditorContolType>TEXT_BOX</EditorContolType>

<MinSliderValue><![CDATA[0]]></MinSliderValue>

<MaxSliderValue><![CDATA[100]]></MaxSliderValue>

<DelimeterType>NO_DELIMETER</DelimeterType>

</ParameterEditor>

</Properties>

</Variable>

<Variable Class="Parameter">

<Id>1393271881881</Id>

<Name><![CDATA[cutoff1]]></Name>

<Description><![CDATA[Poorest -> Poor cutoff]]></Description>

<X>-700</X><Y>390</Y>

<Label><X>10</X><Y>0</Y></Label>

<PublicFlag>false</PublicFlag>

<PresentationFlag>false</PresentationFlag>

<ShowLabel>true</ShowLabel>

<Properties SaveInSnapshot="true" ModificatorType="STATIC">

<Type><![CDATA[double]]></Type>

<SdArray>false</SdArray>

<DefaultValue><![CDATA[200.81]]></DefaultValue>

<ParameterEditor>

<Id>1393271881879</Id>

<Name><![CDATA[]]></Name>

<EditorContolType>TEXT_BOX</EditorContolType>

<MinSliderValue><![CDATA[0]]></MinSliderValue>

<MaxSliderValue><![CDATA[100]]></MaxSliderValue>

<DelimeterType>NO_DELIMETER</DelimeterType>

</ParameterEditor>

</Properties>

</Variable>

<Variable Class="Parameter">

<Id>1393271899699</Id>

<Name><![CDATA[cutoff2]]></Name>

<Description><![CDATA[Poor -> Middle cutoff]]></Description>

<X>-640</X><Y>390</Y>

<Label><X>10</X><Y>0</Y></Label>

<PublicFlag>false</PublicFlag>

<PresentationFlag>false</PresentationFlag>

<ShowLabel>true</ShowLabel>

<Properties SaveInSnapshot="true" ModificatorType="STATIC">

<Type><![CDATA[double]]></Type>

<SdArray>false</SdArray>

<DefaultValue><![CDATA[277.39]]></DefaultValue>

<ParameterEditor>

<Id>1393271899697</Id>

<Name><![CDATA[]]></Name>

<EditorContolType>TEXT_BOX</EditorContolType>

<MinSliderValue><![CDATA[0]]></MinSliderValue>

<MaxSliderValue><![CDATA[100]]></MaxSliderValue>

<DelimeterType>NO_DELIMETER</DelimeterType>

</ParameterEditor>

</Properties>

</Variable>

<Variable Class="Parameter">

<Id>1393271900324</Id>

<Name><![CDATA[cutoff3]]></Name>

<Description><![CDATA[Middle -> Rich cutoff]]></Description>

<X>-700</X><Y>410</Y>

<Label><X>10</X><Y>0</Y></Label>

<PublicFlag>false</PublicFlag>

<PresentationFlag>false</PresentationFlag>

<ShowLabel>true</ShowLabel>

<Properties SaveInSnapshot="true" ModificatorType="STATIC">

<Type><![CDATA[double]]></Type>

<SdArray>false</SdArray>

<DefaultValue><![CDATA[357.53]]></DefaultValue>

<ParameterEditor>

<Id>1393271900322</Id>

<Name><![CDATA[]]></Name>

<EditorContolType>TEXT_BOX</EditorContolType>

<MinSliderValue><![CDATA[0]]></MinSliderValue>

<MaxSliderValue><![CDATA[100]]></MaxSliderValue>

<DelimeterType>NO_DELIMETER</DelimeterType>

</ParameterEditor>

</Properties>

</Variable>

<Variable Class="Parameter">

<Id>1393271900668</Id>

<Name><![CDATA[cutoff4]]></Name>

<Description><![CDATA[Rich -> Richest cutoff]]></Description>

<X>-640</X><Y>410</Y>

<Label><X>10</X><Y>0</Y></Label>

<PublicFlag>false</PublicFlag>

<PresentationFlag>false</PresentationFlag>

<ShowLabel>true</ShowLabel>

<Properties SaveInSnapshot="true" ModificatorType="STATIC">

<Type><![CDATA[double]]></Type>

<SdArray>false</SdArray>

<DefaultValue><![CDATA[468.29]]></DefaultValue>

<ParameterEditor>

<Id>1393271900666</Id>

<Name><![CDATA[]]></Name>

<EditorContolType>TEXT_BOX</EditorContolType>

<MinSliderValue><![CDATA[0]]></MinSliderValue>

<MaxSliderValue><![CDATA[100]]></MaxSliderValue>

<DelimeterType>NO_DELIMETER</DelimeterType>

</ParameterEditor>

</Properties>

</Variable>

<Variable Class="Parameter">

<Id>1393272127021</Id>

<Name><![CDATA[urbanIncome]]></Name>

<X>-700</X><Y>290</Y>

<Label><X>10</X><Y>0</Y></Label>

<PublicFlag>false</PublicFlag>

<PresentationFlag>false</PresentationFlag>

<ShowLabel>true</ShowLabel>

<Properties SaveInSnapshot="true" ModificatorType="STATIC">

<Type><![CDATA[double]]></Type>

<SdArray>false</SdArray>

<DefaultValue><![CDATA[268.83]]></DefaultValue>

<ParameterEditor>

<Id>1393272127019</Id>

<Name><![CDATA[]]></Name>

<EditorContolType>TEXT_BOX</EditorContolType>

<MinSliderValue><![CDATA[0]]></MinSliderValue>

<MaxSliderValue><![CDATA[100]]></MaxSliderValue>

<DelimeterType>NO_DELIMETER</DelimeterType>

</ParameterEditor>

</Properties>

</Variable>

<Variable Class="Parameter">

<Id>1393272127024</Id>

<Name><![CDATA[ruralIncome]]></Name>

<X>-700</X><Y>310</Y>

<Label><X>10</X><Y>0</Y></Label>

<PublicFlag>false</PublicFlag>

<PresentationFlag>false</PresentationFlag>

<ShowLabel>true</ShowLabel>

<Properties SaveInSnapshot="true" ModificatorType="STATIC">

<Type><![CDATA[double]]></Type>

<SdArray>false</SdArray>

<DefaultValue><![CDATA[90.67]]></DefaultValue>

<ParameterEditor>

<Id>1393272127022</Id>

<Name><![CDATA[]]></Name>

<EditorContolType>TEXT_BOX</EditorContolType>

<MinSliderValue><![CDATA[0]]></MinSliderValue>

<MaxSliderValue><![CDATA[100]]></MaxSliderValue>

<DelimeterType>NO_DELIMETER</DelimeterType>

</ParameterEditor>

</Properties>

</Variable>

<Variable Class="Parameter">

<Id>1393272127027</Id>

<Name><![CDATA[urbanExpenditure]]></Name>

<X>-700</X><Y>330</Y>

<Label><X>10</X><Y>0</Y></Label>

<PublicFlag>false</PublicFlag>

<PresentationFlag>false</PresentationFlag>

<ShowLabel>true</ShowLabel>

<Properties SaveInSnapshot="true" ModificatorType="STATIC">

<Type><![CDATA[double]]></Type>

<SdArray>false</SdArray>

<DefaultValue><![CDATA[156.56]]></DefaultValue>

<ParameterEditor>

<Id>1393272127025</Id>

<Name><![CDATA[]]></Name>

<EditorContolType>TEXT_BOX</EditorContolType>

<MinSliderValue><![CDATA[0]]></MinSliderValue>

<MaxSliderValue><![CDATA[100]]></MaxSliderValue>

<DelimeterType>NO_DELIMETER</DelimeterType>

</ParameterEditor>

</Properties>

</Variable>

<Variable Class="Parameter">

<Id>1393272127030</Id>

<Name><![CDATA[ruralExpenditure]]></Name>

<X>-700</X><Y>350</Y>

<Label><X>10</X><Y>0</Y></Label>

<PublicFlag>false</PublicFlag>

<PresentationFlag>false</PresentationFlag>

<ShowLabel>true</ShowLabel>

<Properties SaveInSnapshot="true" ModificatorType="STATIC">

<Type><![CDATA[double]]></Type>

<SdArray>false</SdArray>

<DefaultValue><![CDATA[80.49]]></DefaultValue>

<ParameterEditor>

<Id>1393272127028</Id>

<Name><![CDATA[]]></Name>

<EditorContolType>TEXT_BOX</EditorContolType>

<MinSliderValue><![CDATA[0]]></MinSliderValue>

<MaxSliderValue><![CDATA[100]]></MaxSliderValue>

<DelimeterType>NO_DELIMETER</DelimeterType>

</ParameterEditor>

</Properties>

</Variable>

<Variable Class="Parameter">

<Id>1393272987620</Id>

<Name><![CDATA[povertyLine]]></Name>

<X>-640</X><Y>430</Y>

<Label><X>10</X><Y>0</Y></Label>

<PublicFlag>false</PublicFlag>

<PresentationFlag>false</PresentationFlag>

<ShowLabel>true</ShowLabel>

<Properties SaveInSnapshot="true" ModificatorType="STATIC">

<Type><![CDATA[double]]></Type>

<SdArray>false</SdArray>

<DefaultValue><![CDATA[]]></DefaultValue>

<ParameterEditor>

<Id>1393272987618</Id>

<Name><![CDATA[]]></Name>

<EditorContolType>TEXT_BOX</EditorContolType>

<MinSliderValue><![CDATA[0]]></MinSliderValue>

<MaxSliderValue><![CDATA[100]]></MaxSliderValue>

<DelimeterType>NO_DELIMETER</DelimeterType>

</ParameterEditor>

</Properties>

</Variable>

<Variable Class="Parameter">

<Id>1410464091026</Id>

<Name><![CDATA[cSurg]]></Name>

<X>-910</X><Y>70</Y>

<Label><X>10</X><Y>0</Y></Label>

<PublicFlag>false</PublicFlag>

<PresentationFlag>false</PresentationFlag>

<ShowLabel>true</ShowLabel>

<Properties SaveInSnapshot="true" ModificatorType="STATIC">

<Type><![CDATA[double]]></Type>

<SdArray>false</SdArray>

<DefaultValue><![CDATA[143.43]]></DefaultValue>

<ParameterEditor>

<Id>1410464091024</Id>

<Name><![CDATA[]]></Name>

<EditorContolType>TEXT_BOX</EditorContolType>

<MinSliderValue><![CDATA[0]]></MinSliderValue>

<MaxSliderValue><![CDATA[100]]></MaxSliderValue>

<DelimeterType>NO_DELIMETER</DelimeterType>

</ParameterEditor>

</Properties>

</Variable>

<Variable Class="Parameter">

<Id>1410464299622</Id>

<Name><![CDATA[OOP]]></Name>

<X>-910</X><Y>90</Y>

<Label><X>10</X><Y>0</Y></Label>

<PublicFlag>false</PublicFlag>

<PresentationFlag>false</PresentationFlag>

<ShowLabel>true</ShowLabel>

<Properties SaveInSnapshot="true" ModificatorType="STATIC">

<Type><![CDATA[double]]></Type>

<SdArray>false</SdArray>

<DefaultValue><![CDATA[0.493288938]]></DefaultValue>

<ParameterEditor>

<Id>1410464299620</Id>

<Name><![CDATA[]]></Name>

<EditorContolType>TEXT_BOX</EditorContolType>

<MinSliderValue><![CDATA[0]]></MinSliderValue>

<MaxSliderValue><![CDATA[100]]></MaxSliderValue>

<DelimeterType>NO_DELIMETER</DelimeterType>

</ParameterEditor>

</Properties>

</Variable>

<Variable Class="Parameter">

<Id>1410464340506</Id>

<Name><![CDATA[mNonMed]]></Name>

<Description><![CDATA[Non-medical cost multiplier (multiplies surgical cost)]]></Description>

<X>-910</X><Y>110</Y>

<Label><X>10</X><Y>0</Y></Label>

<PublicFlag>false</PublicFlag>

<PresentationFlag>false</PresentationFlag>

<ShowLabel>true</ShowLabel>

<Properties SaveInSnapshot="true" ModificatorType="STATIC">

<Type><![CDATA[double]]></Type>

<SdArray>false</SdArray>

<DefaultValue><![CDATA[1.2354]]></DefaultValue>

<ParameterEditor>

<Id>1410464340504</Id>

<Name><![CDATA[]]></Name>

<EditorContolType>TEXT_BOX</EditorContolType>

<MinSliderValue><![CDATA[0]]></MinSliderValue>

<MaxSliderValue><![CDATA[100]]></MaxSliderValue>

<DelimeterType>NO_DELIMETER</DelimeterType>

</ParameterEditor>

</Properties>

</Variable>

<Variable Class="Parameter">

<Id>1410554121677</Id>

<Name><![CDATA[periopMortality]]></Name>

<X>-910</X><Y>130</Y>

<Label><X>10</X><Y>0</Y></Label>

<PublicFlag>false</PublicFlag>

<PresentationFlag>false</PresentationFlag>

<ShowLabel>true</ShowLabel>

<Properties SaveInSnapshot="true" ModificatorType="STATIC">

<Type><![CDATA[double]]></Type>

<SdArray>false</SdArray>

<DefaultValue><![CDATA[0.03]]></DefaultValue>

<ParameterEditor>

<Id>1410554121675</Id>

<Name><![CDATA[]]></Name>

<EditorContolType>TEXT_BOX</EditorContolType>

<MinSliderValue><![CDATA[0]]></MinSliderValue>

<MaxSliderValue><![CDATA[100]]></MaxSliderValue>

<DelimeterType>NO_DELIMETER</DelimeterType>

</ParameterEditor>

</Properties>

</Variable>

<Variable Class="Parameter">

<Id>1410983891904</Id>

<Name><![CDATA[mDistCost]]></Name>

<Description><![CDATA[Scaling factor for district hospitals]]></Description>

<X>-910</X><Y>150</Y>

<Label><X>10</X><Y>0</Y></Label>

<PublicFlag>false</PublicFlag>

<PresentationFlag>false</PresentationFlag>

<ShowLabel>true</ShowLabel>

<Properties SaveInSnapshot="true" ModificatorType="STATIC">

<Type><![CDATA[double]]></Type>

<SdArray>false</SdArray>

<DefaultValue><![CDATA[1/1.47]]></DefaultValue>

<ParameterEditor>

<Id>1410983891902</Id>

<Name><![CDATA[]]></Name>

<EditorContolType>TEXT_BOX</EditorContolType>

<MinSliderValue><![CDATA[0]]></MinSliderValue>

<MaxSliderValue><![CDATA[100]]></MaxSliderValue>

<DelimeterType>NO_DELIMETER</DelimeterType>

</ParameterEditor>

</Properties>

</Variable>

<Variable Class="Parameter">

<Id>1410983994872</Id>

<Name><![CDATA[mRegCost]]></Name>

<Description><![CDATA[Scaling factor for district hospitals]]></Description>

<X>-910</X><Y>170</Y>

<Label><X>10</X><Y>0</Y></Label>

<PublicFlag>false</PublicFlag>

<PresentationFlag>false</PresentationFlag>

<ShowLabel>true</ShowLabel>

<Properties SaveInSnapshot="true" ModificatorType="STATIC">

<Type><![CDATA[double]]></Type>

<SdArray>false</SdArray>

<DefaultValue><![CDATA[1/uniform(1.1,1.4)]]></DefaultValue>

<ParameterEditor>

<Id>1410983994870</Id>

<Name><![CDATA[]]></Name>

<EditorContolType>TEXT_BOX</EditorContolType>

<MinSliderValue><![CDATA[0]]></MinSliderValue>

<MaxSliderValue><![CDATA[100]]></MaxSliderValue>

<DelimeterType>NO_DELIMETER</DelimeterType>

</ParameterEditor>

</Properties>

</Variable>

<Variable Class="Parameter">

<Id>1410984184227</Id>

<Name><![CDATA[mDistMort]]></Name>

<Description><![CDATA[Multiplier for mortality in a district hospital (multiplied to national hospital mortality rate)]]></Description>

<X>-510</X><Y>530</Y>

<Label><X>10</X><Y>0</Y></Label>

<PublicFlag>false</PublicFlag>

<PresentationFlag>false</PresentationFlag>

<ShowLabel>true</ShowLabel>

<Properties SaveInSnapshot="true" ModificatorType="STATIC">

<Type><![CDATA[double]]></Type>

<SdArray>false</SdArray>

<DefaultValue><![CDATA[1.125]]></DefaultValue>

<ParameterEditor>

<Id>1410984184225</Id>

<Name><![CDATA[]]></Name>

<EditorContolType>TEXT_BOX</EditorContolType>

<MinSliderValue><![CDATA[0]]></MinSliderValue>

<MaxSliderValue><![CDATA[100]]></MaxSliderValue>

<DelimeterType>NO_DELIMETER</DelimeterType>

</ParameterEditor>

</Properties>

</Variable>

<Variable Class="Parameter">

<Id>1410984228229</Id>

<Name><![CDATA[mRegMort]]></Name>

<Description><![CDATA[Multiplier for mortality in a district hospital (multiplied to national hospital mortality rate)]]></Description>

<X>-510</X><Y>550</Y>

<Label><X>10</X><Y>0</Y></Label>

<PublicFlag>false</PublicFlag>

<PresentationFlag>false</PresentationFlag>

<ShowLabel>true</ShowLabel>

<Properties SaveInSnapshot="true" ModificatorType="STATIC">

<Type><![CDATA[double]]></Type>

<SdArray>false</SdArray>

<DefaultValue><![CDATA[uniform(1.01,1.2)]]></DefaultValue>

<ParameterEditor>

<Id>1410984228227</Id>

<Name><![CDATA[]]></Name>

<EditorContolType>TEXT_BOX</EditorContolType>

<MinSliderValue><![CDATA[0]]></MinSliderValue>

<MaxSliderValue><![CDATA[100]]></MaxSliderValue>

<DelimeterType>NO_DELIMETER</DelimeterType>

</ParameterEditor>

</Properties>

</Variable>

<Variable Class="Parameter">

<Id>1411509286787</Id>

<Name><![CDATA[meanTxLength]]></Name>

<X>-510</X><Y>510</Y>

<Label><X>10</X><Y>0</Y></Label>

<PublicFlag>false</PublicFlag>

<PresentationFlag>false</PresentationFlag>

<ShowLabel>true</ShowLabel>

<Properties SaveInSnapshot="true" ModificatorType="STATIC">

<Type><![CDATA[int]]></Type>

<SdArray>false</SdArray>

<DefaultValue><![CDATA[14]]></DefaultValue>

<ParameterEditor>

<Id>1411509286785</Id>

<Name><![CDATA[]]></Name>

<EditorContolType>TEXT_BOX</EditorContolType>

<MinSliderValue><![CDATA[0]]></MinSliderValue>

<MaxSliderValue><![CDATA[100]]></MaxSliderValue>

<DelimeterType>NO_DELIMETER</DelimeterType>

</ParameterEditor>

</Properties>

</Variable>

<Variable Class="Parameter">

<Id>1411580227497</Id>

<Name><![CDATA[catExpThreshold]]></Name>

<X>-700</X><Y>450</Y>

<Label><X>10</X><Y>0</Y></Label>

<PublicFlag>false</PublicFlag>

<PresentationFlag>false</PresentationFlag>

<ShowLabel>true</ShowLabel>

<Properties SaveInSnapshot="true" ModificatorType="STATIC">

<Type><![CDATA[double]]></Type>

<SdArray>false</SdArray>

<DefaultValue><![CDATA[0.1]]></DefaultValue>

<ParameterEditor>

<Id>1411580227495</Id>

<Name><![CDATA[]]></Name>

<EditorContolType>TEXT_BOX</EditorContolType>

<MinSliderValue><![CDATA[0]]></MinSliderValue>

<MaxSliderValue><![CDATA[100]]></MaxSliderValue>

<DelimeterType>NO_DELIMETER</DelimeterType>

</ParameterEditor>

</Properties>

</Variable>

<Variable Class="Parameter">

<Id>1412535055017</Id>

<Name><![CDATA[cancerHospital]]></Name>

<X>-910</X><Y>370</Y>

<Label><X>10</X><Y>0</Y></Label>

<PublicFlag>false</PublicFlag>

<PresentationFlag>false</PresentationFlag>

<ShowLabel>true</ShowLabel>

<Properties SaveInSnapshot="true" ModificatorType="STATIC">

<Type><![CDATA[boolean]]></Type>

<SdArray>false</SdArray>

<DefaultValue><![CDATA[false]]></DefaultValue>

<ParameterEditor>

<Id>1412535055015</Id>

<Name><![CDATA[]]></Name>

<EditorContolType>CHECK_BOX</EditorContolType>

<MinSliderValue><![CDATA[0]]></MinSliderValue>

<MaxSliderValue><![CDATA[100]]></MaxSliderValue>

<DelimeterType>NO_DELIMETER</DelimeterType>

</ParameterEditor>

</Properties>

</Variable>

<Variable Class="Parameter">

<Id>1412535055020</Id>

<Name><![CDATA[mercyShips]]></Name>

<X>-910</X><Y>350</Y>

<Label><X>10</X><Y>0</Y></Label>

<PublicFlag>false</PublicFlag>

<PresentationFlag>false</PresentationFlag>

<ShowLabel>true</ShowLabel>

<Properties SaveInSnapshot="true" ModificatorType="STATIC">

<Type><![CDATA[boolean]]></Type>

<SdArray>false</SdArray>

<DefaultValue><![CDATA[false]]></DefaultValue>

<ParameterEditor>

<Id>1412535055018</Id>

<Name><![CDATA[]]></Name>

<EditorContolType>CHECK_BOX</EditorContolType>

<MinSliderValue><![CDATA[0]]></MinSliderValue>

<MaxSliderValue><![CDATA[100]]></MaxSliderValue>

<DelimeterType>NO_DELIMETER</DelimeterType>

</ParameterEditor>

</Properties>

</Variable>

<Variable Class="Parameter">

<Id>1412535055023</Id>

<Name><![CDATA[twoWeekTrips]]></Name>

<X>-910</X><Y>330</Y>

<Label><X>10</X><Y>0</Y></Label>

<PublicFlag>false</PublicFlag>

<PresentationFlag>false</PresentationFlag>

<ShowLabel>true</ShowLabel>

<Properties SaveInSnapshot="true" ModificatorType="STATIC">

<Type><![CDATA[boolean]]></Type>

<SdArray>false</SdArray>

<DefaultValue><![CDATA[false]]></DefaultValue>

<ParameterEditor>

<Id>1412535055021</Id>

<Name><![CDATA[]]></Name>

<EditorContolType>CHECK_BOX</EditorContolType>

<MinSliderValue><![CDATA[0]]></MinSliderValue>

<MaxSliderValue><![CDATA[100]]></MaxSliderValue>

<DelimeterType>NO_DELIMETER</DelimeterType>

</ParameterEditor>

</Properties>

</Variable>

<Variable Class="Parameter">

<Id>1412535055026</Id>

<Name><![CDATA[UPF]]></Name>

<Description><![CDATA[Sets the transparency of the individual population based on the population size, such that the bigger the population, the more transparent any one individual is]]></Description>

<X>-910</X><Y>390</Y>

<Label><X>10</X><Y>0</Y></Label>

<PublicFlag>false</PublicFlag>

<PresentationFlag>false</PresentationFlag>

<ShowLabel>true</ShowLabel>

<Properties SaveInSnapshot="true" ModificatorType="STATIC">

<Type><![CDATA[boolean]]></Type>

<SdArray>false</SdArray>

<DefaultValue><![CDATA[false]]></DefaultValue>

<ParameterEditor>

<Id>1412535055024</Id>

<Name><![CDATA[]]></Name>

<EditorContolType>CHECK_BOX</EditorContolType>

<MinSliderValue><![CDATA[0]]></MinSliderValue>

<MaxSliderValue><![CDATA[100]]></MaxSliderValue>

<DelimeterType>NO_DELIMETER</DelimeterType>

</ParameterEditor>

</Properties>

</Variable>

<Variable Class="Parameter">

<Id>1412535259186</Id>

<Name><![CDATA[cNonMed]]></Name>

<Description><![CDATA[Non-medical cost baseline (= 134.80 * 1.2354)]]></Description>

<X>-830</X><Y>110</Y>

<Label><X>10</X><Y>0</Y></Label>

<PublicFlag>false</PublicFlag>

<PresentationFlag>false</PresentationFlag>

<ShowLabel>true</ShowLabel>

<Properties SaveInSnapshot="true" ModificatorType="STATIC">

<Type><![CDATA[double]]></Type>

<SdArray>false</SdArray>

<DefaultValue><![CDATA[166.53192]]></DefaultValue>

<ParameterEditor>

<Id>1412535259184</Id>

<Name><![CDATA[]]></Name>

<EditorContolType>TEXT_BOX</EditorContolType>

<MinSliderValue><![CDATA[0]]></MinSliderValue>

<MaxSliderValue><![CDATA[100]]></MaxSliderValue>

<DelimeterType>NO_DELIMETER</DelimeterType>

</ParameterEditor>

</Properties>

</Variable>

<Variable Class="Parameter">

<Id>1412535319868</Id>

<Name><![CDATA[vouchers]]></Name>

<Description><![CDATA[Sets the transparency of the individual population based on the population size, such that the bigger the population, the more transparent any one individual is]]></Description>

<X>-910</X><Y>430</Y>

<Label><X>10</X><Y>0</Y></Label>

<PublicFlag>false</PublicFlag>

<PresentationFlag>false</PresentationFlag>

<ShowLabel>true</ShowLabel>

<Properties SaveInSnapshot="true" ModificatorType="STATIC">

<Type><![CDATA[boolean]]></Type>

<SdArray>false</SdArray>

<DefaultValue><![CDATA[false]]></DefaultValue>

<ParameterEditor>

<Id>1412535319866</Id>

<Name><![CDATA[]]></Name>

<EditorContolType>CHECK_BOX</EditorContolType>

<MinSliderValue><![CDATA[0]]></MinSliderValue>

<MaxSliderValue><![CDATA[100]]></MaxSliderValue>

<DelimeterType>NO_DELIMETER</DelimeterType>

</ParameterEditor>

</Properties>

</Variable>

<Variable Class="Parameter">

<Id>1412535322180</Id>

<Name><![CDATA[TS]]></Name>

<Description><![CDATA[Sets the transparency of the individual population based on the population size, such that the bigger the population, the more transparent any one individual is]]></Description>

<X>-910</X><Y>410</Y>

<Label><X>10</X><Y>0</Y></Label>

<PublicFlag>false</PublicFlag>

<PresentationFlag>false</PresentationFlag>

<ShowLabel>true</ShowLabel>

<Properties SaveInSnapshot="true" ModificatorType="STATIC">

<Type><![CDATA[boolean]]></Type>

<SdArray>false</SdArray>

<DefaultValue><![CDATA[false]]></DefaultValue>

<ParameterEditor>

<Id>1412535322178</Id>

<Name><![CDATA[]]></Name>

<EditorContolType>CHECK_BOX</EditorContolType>

<MinSliderValue><![CDATA[0]]></MinSliderValue>

<MaxSliderValue><![CDATA[100]]></MaxSliderValue>

<DelimeterType>NO_DELIMETER</DelimeterType>

</ParameterEditor>

</Properties>

</Variable>

<Variable Class="Parameter">

<Id>1412538425974</Id>

<Name><![CDATA[meanTripLength]]></Name>

<X>-910</X><Y>470</Y>

<Label><X>10</X><Y>0</Y></Label>

<PublicFlag>false</PublicFlag>

<PresentationFlag>true</PresentationFlag>

<ShowLabel>true</ShowLabel>

<Properties SaveInSnapshot="true" ModificatorType="STATIC">

<Type><![CDATA[double]]></Type>

<SdArray>false</SdArray>

<DefaultValue><![CDATA[14]]></DefaultValue>

<ParameterEditor>

<Id>1412538425972</Id>

<Name><![CDATA[]]></Name>

<EditorContolType>TEXT_BOX</EditorContolType>

<MinSliderValue><![CDATA[0]]></MinSliderValue>

<MaxSliderValue><![CDATA[100]]></MaxSliderValue>

<DelimeterType>NO_DELIMETER</DelimeterType>

</ParameterEditor>

</Properties>

</Variable>

<Variable Class="Parameter">

<Id>1412538455409</Id>

<Name><![CDATA[numTripsPerYear]]></Name>

<X>-910</X><Y>490</Y>

<Label><X>10</X><Y>0</Y></Label>

<PublicFlag>false</PublicFlag>

<PresentationFlag>true</PresentationFlag>

<ShowLabel>true</ShowLabel>

<Properties SaveInSnapshot="true" ModificatorType="STATIC">

<Type><![CDATA[double]]></Type>

<SdArray>false</SdArray>

<DefaultValue><![CDATA[2]]></DefaultValue>

<ParameterEditor>

<Id>1412538455407</Id>

<Name><![CDATA[]]></Name>

<EditorContolType>TEXT_BOX</EditorContolType>

<MinSliderValue><![CDATA[0]]></MinSliderValue>

<MaxSliderValue><![CDATA[100]]></MaxSliderValue>

<DelimeterType>NO_DELIMETER</DelimeterType>

</ParameterEditor>

</Properties>

</Variable>

<Variable Class="Parameter">

<Id>1412610233888</Id>

<Name><![CDATA[yearsBetweenMS]]></Name>

<X>-910</X><Y>510</Y>

<Label><X>10</X><Y>0</Y></Label>

<PublicFlag>false</PublicFlag>

<PresentationFlag>true</PresentationFlag>

<ShowLabel>true</ShowLabel>

<Properties SaveInSnapshot="true" ModificatorType="STATIC">

<Type><![CDATA[double]]></Type>

<SdArray>false</SdArray>

<DefaultValue><![CDATA[]]></DefaultValue>

<ParameterEditor>

<Id>1412610233886</Id>

<Name><![CDATA[]]></Name>

<EditorContolType>TEXT_BOX</EditorContolType>

<MinSliderValue><![CDATA[0]]></MinSliderValue>

<MaxSliderValue><![CDATA[100]]></MaxSliderValue>

<DelimeterType>NO_DELIMETER</DelimeterType>

</ParameterEditor>

</Properties>

</Variable>

<Variable Class="Parameter">

<Id>1412619526593</Id>

<Name><![CDATA[vouchersMult]]></Name>

<X>-910</X><Y>450</Y>

<Label><X>10</X><Y>0</Y></Label>

<PublicFlag>false</PublicFlag>

<PresentationFlag>true</PresentationFlag>

<ShowLabel>true</ShowLabel>

<Properties SaveInSnapshot="true" ModificatorType="STATIC">

<Type><![CDATA[double]]></Type>

<SdArray>false</SdArray>

<DefaultValue><![CDATA[vouchers ? 0.0 : 1.0]]></DefaultValue>

<ParameterEditor>

<Id>1412619526591</Id>

<Name><![CDATA[]]></Name>

<EditorContolType>TEXT_BOX</EditorContolType>

<MinSliderValue><![CDATA[0]]></MinSliderValue>

<MaxSliderValue><![CDATA[100]]></MaxSliderValue>

<DelimeterType>NO_DELIMETER</DelimeterType>

</ParameterEditor>

</Properties>

</Variable>

<Variable Class="Parameter">

<Id>1412736140147</Id>

<Name><![CDATA[mMortTwoWeek]]></Name>

<X>-910</X><Y>530</Y>

<Label><X>10</X><Y>0</Y></Label>

<PublicFlag>false</PublicFlag>

<PresentationFlag>true</PresentationFlag>

<ShowLabel>true</ShowLabel>

<Properties SaveInSnapshot="true" ModificatorType="STATIC">

<Type><![CDATA[double]]></Type>

<SdArray>false</SdArray>

<DefaultValue><![CDATA[1.0]]></DefaultValue>

<ParameterEditor>

<Id>1412736140145</Id>

<Name><![CDATA[]]></Name>

<EditorContolType>TEXT_BOX</EditorContolType>

<MinSliderValue><![CDATA[0]]></MinSliderValue>

<MaxSliderValue><![CDATA[100]]></MaxSliderValue>

<DelimeterType>NO_DELIMETER</DelimeterType>

</ParameterEditor>

</Properties>

</Variable>

<Variable Class="Parameter">

<Id>1412736156924</Id>

<Name><![CDATA[mMortMercyShips]]></Name>

<X>-910</X><Y>550</Y>

<Label><X>10</X><Y>0</Y></Label>

<PublicFlag>false</PublicFlag>

<PresentationFlag>true</PresentationFlag>

<ShowLabel>true</ShowLabel>

<Properties SaveInSnapshot="true" ModificatorType="STATIC">

<Type><![CDATA[double]]></Type>

<SdArray>false</SdArray>

<DefaultValue><![CDATA[0.8]]></DefaultValue>

<ParameterEditor>

<Id>1412736156922</Id>

<Name><![CDATA[]]></Name>

<EditorContolType>TEXT_BOX</EditorContolType>

<MinSliderValue><![CDATA[0]]></MinSliderValue>

<MaxSliderValue><![CDATA[100]]></MaxSliderValue>

<DelimeterType>NO_DELIMETER</DelimeterType>

</ParameterEditor>

</Properties>

</Variable>

<Variable Class="Parameter">

<Id>1412785039202</Id>

<Name><![CDATA[complSurgHosp]]></Name>

<X>-360</X><Y>510</Y>

<Label><X>10</X><Y>0</Y></Label>

<PublicFlag>false</PublicFlag>

<PresentationFlag>true</PresentationFlag>

<ShowLabel>true</ShowLabel>

<Properties SaveInSnapshot="true" ModificatorType="STATIC">

<Type><![CDATA[double]]></Type>

<SdArray>false</SdArray>

<DefaultValue><![CDATA[0.14]]></DefaultValue>

<ParameterEditor>

<Id>1412785039194</Id>

<Name><![CDATA[]]></Name>

<EditorContolType>TEXT_BOX</EditorContolType>

<MinSliderValue><![CDATA[0]]></MinSliderValue>

<MaxSliderValue><![CDATA[100]]></MaxSliderValue>

<DelimeterType>NO_DELIMETER</DelimeterType>

</ParameterEditor>

</Properties>

</Variable>

<Variable Class="Parameter">

<Id>1412785059283</Id>

<Name><![CDATA[complSurgRegDist]]></Name>

<X>-360</X><Y>530</Y>

<Label><X>10</X><Y>0</Y></Label>

<PublicFlag>false</PublicFlag>

<PresentationFlag>true</PresentationFlag>

<ShowLabel>true</ShowLabel>

<Properties SaveInSnapshot="true" ModificatorType="STATIC">

<Type><![CDATA[double]]></Type>

<SdArray>false</SdArray>

<DefaultValue><![CDATA[0.1575]]></DefaultValue>

<ParameterEditor>

<Id>1412785059281</Id>

<Name><![CDATA[]]></Name>

<EditorContolType>TEXT_BOX</EditorContolType>

<MinSliderValue><![CDATA[0]]></MinSliderValue>

<MaxSliderValue><![CDATA[100]]></MaxSliderValue>

<DelimeterType>NO_DELIMETER</DelimeterType>

</ParameterEditor>

</Properties>

</Variable>

<Variable Class="Parameter">

<Id>1412785348638</Id>

<Name><![CDATA[cCompl]]></Name>

<X>-830</X><Y>70</Y>

<Label><X>10</X><Y>0</Y></Label>

<PublicFlag>false</PublicFlag>

<PresentationFlag>true</PresentationFlag>

<ShowLabel>true</ShowLabel>

<Properties SaveInSnapshot="true" ModificatorType="STATIC">

<Type><![CDATA[double]]></Type>

<SdArray>false</SdArray>

<DefaultValue><![CDATA[10.56

]]></DefaultValue>

<ParameterEditor>

<Id>1412785348636</Id>

<Name><![CDATA[]]></Name>

<EditorContolType>TEXT_BOX</EditorContolType>

<MinSliderValue><![CDATA[0]]></MinSliderValue>

<MaxSliderValue><![CDATA[100]]></MaxSliderValue>

<DelimeterType>NO_DELIMETER</DelimeterType>

</ParameterEditor>

</Properties>

</Variable>

<Variable Class="Parameter">

<Id>1412785387874</Id>

<Name><![CDATA[pDieCompl]]></Name>

<X>-830</X><Y>90</Y>

<Label><X>10</X><Y>0</Y></Label>

<PublicFlag>false</PublicFlag>

<PresentationFlag>true</PresentationFlag>

<ShowLabel>true</ShowLabel>

<Properties SaveInSnapshot="true" ModificatorType="STATIC">

<Type><![CDATA[double]]></Type>

<SdArray>false</SdArray>

<DefaultValue><![CDATA[0.02]]></DefaultValue>

<ParameterEditor>

<Id>1412785387872</Id>

<Name><![CDATA[]]></Name>

<EditorContolType>TEXT_BOX</EditorContolType>

<MinSliderValue><![CDATA[0]]></MinSliderValue>

<MaxSliderValue><![CDATA[100]]></MaxSliderValue>

<DelimeterType>NO_DELIMETER</DelimeterType>

</ParameterEditor>

</Properties>

</Variable>

<Variable Class="Parameter">

<Id>1412895497924</Id>

<Name><![CDATA[meanQual]]></Name>

<X>-910</X><Y>190</Y>

<Label><X>10</X><Y>0</Y></Label>

<PublicFlag>false</PublicFlag>

<PresentationFlag>true</PresentationFlag>

<ShowLabel>true</ShowLabel>

<Properties SaveInSnapshot="true" ModificatorType="STATIC">

<Type><![CDATA[double]]></Type>

<SdArray>false</SdArray>

<DefaultValue><![CDATA[]]></DefaultValue>

<ParameterEditor>

<Id>1412895497922</Id>

<Name><![CDATA[]]></Name>

<EditorContolType>TEXT_BOX</EditorContolType>

<MinSliderValue><![CDATA[0]]></MinSliderValue>

<MaxSliderValue><![CDATA[100]]></MaxSliderValue>

<DelimeterType>NO_DELIMETER</DelimeterType>

</ParameterEditor>

</Properties>

</Variable>

<Variable Class="Parameter">

<Id>1416594007955</Id>

<Name><![CDATA[meanGDP]]></Name>

<X>-700</X><Y>210</Y>

<Label><X>10</X><Y>0</Y></Label>

<PublicFlag>false</PublicFlag>

<PresentationFlag>true</PresentationFlag>

<ShowLabel>true</ShowLabel>

<Properties SaveInSnapshot="true" ModificatorType="STATIC">

<Type><![CDATA[double]]></Type>

<SdArray>false</SdArray>

<DefaultValue><![CDATA[303700]]></DefaultValue>

<ParameterEditor>

<Id>1416594007953</Id>

<Name><![CDATA[]]></Name>

<EditorContolType>TEXT_BOX</EditorContolType>

<MinSliderValue><![CDATA[0]]></MinSliderValue>

<MaxSliderValue><![CDATA[100]]></MaxSliderValue>

<DelimeterType>NO_DELIMETER</DelimeterType>

</ParameterEditor>

</Properties>

</Variable>

<Variable Class="Parameter">

<Id>1416594386451</Id>

<Name><![CDATA[povPct]]></Name>

<X>-700</X><Y>430</Y>

<Label><X>10</X><Y>0</Y></Label>

<PublicFlag>false</PublicFlag>

<PresentationFlag>true</PresentationFlag>

<ShowLabel>true</ShowLabel>

<Properties SaveInSnapshot="true" ModificatorType="STATIC">

<Type><![CDATA[double]]></Type>

<SdArray>false</SdArray>

<DefaultValue><![CDATA[0.245]]></DefaultValue>

<ParameterEditor>

<Id>1416594386449</Id>

<Name><![CDATA[]]></Name>

<EditorContolType>TEXT_BOX</EditorContolType>

<MinSliderValue><![CDATA[0]]></MinSliderValue>

<MaxSliderValue><![CDATA[100]]></MaxSliderValue>

<DelimeterType>NO_DELIMETER</DelimeterType>

</ParameterEditor>

</Properties>

</Variable>

<Variable Class="Parameter">

<Id>1416782335012</Id>

<Name><![CDATA[conversion]]></Name>

<Description><![CDATA[See Income Calculation.xlsx]]></Description>

<X>-700</X><Y>500</Y>

<Label><X>10</X><Y>0</Y></Label>

<PublicFlag>false</PublicFlag>

<PresentationFlag>true</PresentationFlag>

<ShowLabel>true</ShowLabel>

<Properties SaveInSnapshot="true" ModificatorType="STATIC">

<Type><![CDATA[double]]></Type>

<SdArray>false</SdArray>

<DefaultValue><![CDATA[0.001423738]]></DefaultValue>

<ParameterEditor>

<Id>1416782335010</Id>

<Name><![CDATA[]]></Name>

<EditorContolType>TEXT_BOX</EditorContolType>

<MinSliderValue><![CDATA[0]]></MinSliderValue>

<MaxSliderValue><![CDATA[100]]></MaxSliderValue>

<DelimeterType>NO_DELIMETER</DelimeterType>

</ParameterEditor>

</Properties>

</Variable>

<Variable Class="Parameter">

<Id>1417019910231</Id>

<Name><![CDATA[famSize]]></Name>

<X>-410</X><Y>190</Y>

<Label><X>10</X><Y>0</Y></Label>

<PublicFlag>false</PublicFlag>

<PresentationFlag>false</PresentationFlag>

<ShowLabel>true</ShowLabel>

<Properties SaveInSnapshot="true" ModificatorType="STATIC">

<Type><![CDATA[double]]></Type>

<SdArray>false</SdArray>

<DefaultValue><![CDATA[5.0]]></DefaultValue>

<ParameterEditor>

<Id>1417019910224</Id>

<Name><![CDATA[]]></Name>

<EditorContolType>TEXT_BOX</EditorContolType>

<MinSliderValue><![CDATA[0]]></MinSliderValue>

<MaxSliderValue><![CDATA[100]]></MaxSliderValue>

<DelimeterType>NO_DELIMETER</DelimeterType>

</ParameterEditor>

</Properties>

</Variable>

<Variable Class="Parameter">

<Id>1417202487969</Id>

<Name><![CDATA[count]]></Name>

<X>-150</X><Y>410</Y>

<Label><X>10</X><Y>0</Y></Label>

<PublicFlag>false</PublicFlag>

<PresentationFlag>true</PresentationFlag>

<ShowLabel>true</ShowLabel>

<Properties SaveInSnapshot="true" ModificatorType="STATIC">

<Type><![CDATA[int]]></Type>

<SdArray>false</SdArray>

<DefaultValue><![CDATA[]]></DefaultValue>

<ParameterEditor>

<Id>1417202487967</Id>

<Name><![CDATA[]]></Name>

<EditorContolType>TEXT_BOX</EditorContolType>

<MinSliderValue><![CDATA[0]]></MinSliderValue>

<MaxSliderValue><![CDATA[100]]></MaxSliderValue>

<DelimeterType>NO_DELIMETER</DelimeterType>

</ParameterEditor>

</Properties>

</Variable>

<Variable Class="Parameter">

<Id>1417203295516</Id>

<Name><![CDATA[policy]]></Name>

<X>-150</X><Y>430</Y>

<Label><X>10</X><Y>0</Y></Label>

<PublicFlag>false</PublicFlag>

<PresentationFlag>true</PresentationFlag>

<ShowLabel>true</ShowLabel>

<Properties SaveInSnapshot="true" ModificatorType="STATIC">

<Type><![CDATA[int]]></Type>

<SdArray>false</SdArray>

<DefaultValue><![CDATA[count / 100]]></DefaultValue>

<ParameterEditor>

<Id>1417203295514</Id>

<Name><![CDATA[]]></Name>

<EditorContolType>TEXT_BOX</EditorContolType>

<MinSliderValue><![CDATA[0]]></MinSliderValue>

<MaxSliderValue><![CDATA[100]]></MaxSliderValue>

<DelimeterType>NO_DELIMETER</DelimeterType>

</ParameterEditor>

</Properties>

</Variable>

<Variable Class="Parameter">

<Id>1417203555008</Id>

<Name><![CDATA[nIter]]></Name>

<X>-150</X><Y>450</Y>

<Label><X>10</X><Y>0</Y></Label>

<PublicFlag>false</PublicFlag>

<PresentationFlag>true</PresentationFlag>

<ShowLabel>true</ShowLabel>

<Properties SaveInSnapshot="true" ModificatorType="STATIC">

<Type><![CDATA[int]]></Type>

<SdArray>false</SdArray>

<DefaultValue><![CDATA[]]></DefaultValue>

<ParameterEditor>

<Id>1417203555006</Id>

<Name><![CDATA[]]></Name>

<EditorContolType>TEXT_BOX</EditorContolType>

<MinSliderValue><![CDATA[0]]></MinSliderValue>

<MaxSliderValue><![CDATA[100]]></MaxSliderValue>

<DelimeterType>NO_DELIMETER</DelimeterType>

</ParameterEditor>

</Properties>

</Variable>

<Variable Class="CollectionVariable">

<Id>1392234429795</Id>

<Name><![CDATA[Females]]></Name>

<X>-150</X><Y>510</Y>

<Label><X>10</X><Y>0</Y></Label>

<PublicFlag>false</PublicFlag>

<PresentationFlag>false</PresentationFlag>

<ShowLabel>true</ShowLabel>

<Properties SaveInSnapshot="true" AccessType="public" StaticVariable="false">

<CollectionClass><![CDATA[java.util.LinkedList]]></CollectionClass>

<ElementClass><![CDATA[Person]]></ElementClass>

<ValueElementClass><![CDATA[String]]></ValueElementClass>

</Properties>

</Variable>

<Variable Class="CollectionVariable">

<Id>1410734879234</Id>

<Name><![CDATA[allHospitals]]></Name>

<X>-910</X><Y>220</Y>

<Label><X>10</X><Y>0</Y></Label>

<PublicFlag>false</PublicFlag>

<PresentationFlag>false</PresentationFlag>

<ShowLabel>true</ShowLabel>

<Properties SaveInSnapshot="true" AccessType="public" StaticVariable="false">

<CollectionClass><![CDATA[java.util.ArrayList]]></CollectionClass>

<ElementClass><![CDATA[Agent]]></ElementClass>

<ValueElementClass><![CDATA[String]]></ValueElementClass>

</Properties>

</Variable>

<Variable Class="CollectionVariable">

<Id>1410802687838</Id>

<Name><![CDATA[cancerInc]]></Name>

<X>-1390</X><Y>50</Y>

<Label><X>10</X><Y>0</Y></Label>

<PublicFlag>false</PublicFlag>

<PresentationFlag>false</PresentationFlag>

<ShowLabel>true</ShowLabel>

<Properties SaveInSnapshot="true" AccessType="public" StaticVariable="false">

<CollectionClass><![CDATA[java.util.ArrayList]]></CollectionClass>

<ElementClass><![CDATA[Person]]></ElementClass>

<ValueElementClass><![CDATA[String]]></ValueElementClass>

</Properties>

</Variable>

<Variable Class="CollectionVariable">

<Id>1410992662694</Id>

<Name><![CDATA[cancerTx]]></Name>

<X>-1390</X><Y>70</Y>

<Label><X>10</X><Y>0</Y></Label>

<PublicFlag>false</PublicFlag>

<PresentationFlag>false</PresentationFlag>

<ShowLabel>true</ShowLabel>

<Properties SaveInSnapshot="true" AccessType="public" StaticVariable="false">

<CollectionClass><![CDATA[java.util.ArrayList]]></CollectionClass>

<ElementClass><![CDATA[Person]]></ElementClass>

<ValueElementClass><![CDATA[String]]></ValueElementClass>

</Properties>

</Variable>

<Variable Class="CollectionVariable">

<Id>1411571789253</Id>

<Name><![CDATA[catExp]]></Name>

<X>-1390</X><Y>130</Y>

<Label><X>10</X><Y>0</Y></Label>

<PublicFlag>false</PublicFlag>

<PresentationFlag>false</PresentationFlag>

<ShowLabel>true</ShowLabel>

<Properties SaveInSnapshot="true" AccessType="public" StaticVariable="false">

<CollectionClass><![CDATA[java.util.ArrayList]]></CollectionClass>

<ElementClass><![CDATA[Person]]></ElementClass>

<ValueElementClass><![CDATA[String]]></ValueElementClass>

</Properties>

</Variable>

<Variable Class="CollectionVariable">

<Id>1411571790157</Id>

<Name><![CDATA[madePoorNew]]></Name>

<X>-1390</X><Y>110</Y>

<Label><X>10</X><Y>0</Y></Label>

<PublicFlag>false</PublicFlag>

<PresentationFlag>false</PresentationFlag>

<ShowLabel>true</ShowLabel>

<Properties SaveInSnapshot="true" AccessType="public" StaticVariable="false">

<CollectionClass><![CDATA[java.util.ArrayList]]></CollectionClass>

<ElementClass><![CDATA[Person]]></ElementClass>

<ValueElementClass><![CDATA[String]]></ValueElementClass>

</Properties>

</Variable>

<Variable Class="CollectionVariable">

<Id>1411571790560</Id>

<Name><![CDATA[madePoor]]></Name>

<X>-1390</X><Y>90</Y>

<Label><X>10</X><Y>0</Y></Label>

<PublicFlag>false</PublicFlag>

<PresentationFlag>false</PresentationFlag>

<ShowLabel>true</ShowLabel>

<Properties SaveInSnapshot="true" AccessType="public" StaticVariable="false">

<CollectionClass><![CDATA[java.util.ArrayList]]></CollectionClass>

<ElementClass><![CDATA[Person]]></ElementClass>

<ValueElementClass><![CDATA[String]]></ValueElementClass>

</Properties>

</Variable>

<Variable Class="CollectionVariable">

<Id>1413041829608</Id>

<Name><![CDATA[allHospitalsNGO]]></Name>

<X>-910</X><Y>240</Y>

<Label><X>10</X><Y>0</Y></Label>

<PublicFlag>false</PublicFlag>

<PresentationFlag>true</PresentationFlag>

<ShowLabel>true</ShowLabel>

<Properties SaveInSnapshot="true" AccessType="public" StaticVariable="false">

<CollectionClass><![CDATA[java.util.ArrayList]]></CollectionClass>

<ElementClass><![CDATA[Agent]]></ElementClass>

<ValueElementClass><![CDATA[String]]></ValueElementClass>

</Properties>

</Variable>

</Variables>

<Events>

<Event>

<Id>1392672233531</Id>

<Name><![CDATA[updateData]]></Name>

<X>-300</X><Y>300</Y>

<Label><X>10</X><Y>0</Y></Label>

<PublicFlag>false</PublicFlag>

<PresentationFlag>false</PresentationFlag>

<ShowLabel>true</ShowLabel>

<Properties TriggerType="timeout" Mode="cyclic">

<Timeout><![CDATA[1]]></Timeout>

<Rate><![CDATA[1]]></Rate>

<OccurrenceAtTime>true</OccurrenceAtTime>

<OccurrenceDate>1392710400000</OccurrenceDate>

<OccurrenceTime><![CDATA[1]]></OccurrenceTime>

<RecurrenceCode><![CDATA[365]]></RecurrenceCode>

<RecurrenceTimeUnit>MODEL_TIME_UNIT</RecurrenceTimeUnit>

<Condition><![CDATA[false]]></Condition>

<Action><![CDATA[nAlive = persons.nAlive();

deathsPerK = DeadThisYear / nAlive;

if(liveBirths != 0) infantMortality = 1000*infantDeaths / liveBirths;

//clear counters

DeadThisYear = 0;

infantDeaths = 0;

cancerInc.clear();

cancerTx.clear();

madePoor.clear();

madePoorNew.clear();

catExp.clear();

liveBirths = 0;

systemCost = 0;

persCost = 0;

cancerDeathPoorest = 0;

cancerDeathPoor = 0;

cancerDeathMiddle = 0;

cancerDeathRich = 0;

cancerDeathRichest = 0;

madePoorPoorest = 0;

madePoorPoor = 0;

madePoorMiddle = 0;

madePoorRich = 0;

madePoorRichest = 0;

madePoorNewPoorest = 0;

madePoorNewPoor = 0;

madePoorNewMiddle = 0;

madePoorNewRich = 0;

madePoorNewRichest = 0;

catExpPoorest = 0;

catExpPoor = 0;

catExpMiddle = 0;

catExpRich = 0;

catExpRichest = 0;

cancIncPoorest = 0;

cancIncPoor = 0;

cancIncMiddle = 0;

cancIncRich = 0;

cancIncRichest = 0;

cancTxPoorest = 0;

cancTxPoor = 0;

cancTxMiddle = 0;

cancTxRich = 0;

cancTxRichest = 0;

ngoSurgeries = 0;

//propMarried = (double) persons.nMarried() / persons.nAdult();

//propSingle = (double) persons.nSingle() / persons.nAdult();]]></Action>

</Properties>

</Event>

</Events>

<Functions>

<Function AccessType="default" StaticFunction="false">

<ReturnModificator>VOID</ReturnModificator>

<ReturnType><![CDATA[void]]></ReturnType>

<Id>1390441826781</Id>

<Name><![CDATA[createHospitals]]></Name>

<X>-150</X><Y>80</Y>

<Label><X>10</X><Y>0</Y></Label>

<PublicFlag>false</PublicFlag>

<PresentationFlag>false</PresentationFlag>

<ShowLabel>true</ShowLabel>

<Body><![CDATA[//Each hospital: Name, priv, latitude, longitude, quality, NGO, cost, nmed, amIMercyShips, amITwoWeek

//NATIONAL REFERRAL HOSPITALS (Coordinates from Google Maps)

//Mulago National Referral Hospital: 0*20.482', 32*34.628'

Hospital a = add_hospitals("Mulago",0,0.341366667,32.577133333,hospQuality.getCellNumericValue("Quality score!J8"), false, gamma(urbanGini, cSurg/urbanGini), gamma(urbanGini, mNonMed*cSurg/urbanGini), false, false);

allHospitals.add(a);

allHospitalsNGO.add(a);

//Mbarara Hospital: 0*36.698'S, 30*39.084'

a = add_hospitals("Mbarara",0,-0.611633333,30.6514,hospQuality.getCellNumericValue("Quality score!J9"), false, gamma(urbanGini, cSurg/urbanGini), gamma(urbanGini, mNonMed*cSurg/urbanGini), false, false);

allHospitals.add(a);

allHospitalsNGO.add(a);

//De novo cancer hospital attached to Mbarara

if(cancerHospital) {

a = add_hospitals("Cancer Hospital",1,-0.611633333,30.6514, hospQuality.getCellNumericValue("Quality score with NGO!J11"), true, 0.0, 0.0, false, false);

allHospitalsNGO.add(a);

}

//Mercy Ships (starts in Kampala)

if(mercyShips) {

a = add_hospitals("Mobile platform",1,0.341366667,32.577133333,8, true, 0.0, 0.0, true, false);

allHospitalsNGO.add(a);

}

//Two week trip: Will make it go to Masaka--second-lowest quality score of the regional hospitals, but in densely populated area (as opposed to Moroto--lowest quality score but sparse)

if(twoWeekTrips) {

a = add_hospitals("Two week trip",1,-0.329433333,31.734433333, hospQuality.getCellNumericValue("Quality score with NGO!J11"), true, 0.0, 0.0, false, true);

allHospitalsNGO.add(a);

}

//Butabika Hospital: 0*18.849', 32*39.426'

//add_hospitals("Butabika",0,0.31415,32.6571);]]></Body>

</Function>

<Function AccessType="default" StaticFunction="false">

<ReturnModificator>VOID</ReturnModificator>

<ReturnType><![CDATA[void]]></ReturnType>

<Id>1390766283008</Id>

<Name><![CDATA[createRegionals]]></Name>

<X>-150</X><Y>100</Y>

<Label><X>10</X><Y>0</Y></Label>

<PublicFlag>false</PublicFlag>

<PresentationFlag>false</PresentationFlag>

<ShowLabel>true</ShowLabel>

<Body><![CDATA[//Each hospital: Name, quality, latitude, longitude

//REGIONAL REFERRAL HOSPITALS (Coordinates from Google Maps)

//Arua: 3*1.159', 30*54.803

add_regionals("Arua",0,3.0193166667,30.913383333,hospQuality.getCellNumericValue("Quality score!J11"), false, gamma(urbanGini, mRegCost*cSurg/urbanGini), gamma(urbanGini, mRegCost*mNonMed*cSurg/urbanGini));

//Fort Portal Regional Referral Hospital (aka Buhinga): 0*39.328', 30*16.921'

add_regionals("Fort Portal",0,0.65546667,30.28201667,hospQuality.getCellNumericValue("Quality score!J12"), false, gamma(urbanGini, mRegCost*cSurg/urbanGini), gamma(urbanGini, mRegCost*mNonMed*cSurg/urbanGini));

//Gulu Regional Referral Hospital: 2*46.656', 32*17.903'

add_regionals("Gulu",0,2.7776,32.298383333,hospQuality.getCellNumericValue("Quality score!J13"), false, gamma(urbanGini, mRegCost*cSurg/urbanGini), gamma(urbanGini, mRegCost*mNonMed*cSurg/urbanGini));

//Hoima Regional Referral Hospital: 1*25.675', 31*21.264'

add_regionals("Hoima",0,1.427916667,31.3544,hospQuality.getCellNumericValue("Quality score!J14"), false, gamma(urbanGini, mRegCost*cSurg/urbanGini), gamma(urbanGini, mRegCost*mNonMed*cSurg/urbanGini));

//Jinja Regional Referral Hospital 0*25.879', 33*12.308'

add_regionals("Jinja",0,0.431316667,33.205133333,hospQuality.getCellNumericValue("Quality score!J15"), false, gamma(urbanGini, mRegCost*cSurg/urbanGini), gamma(urbanGini, mRegCost*mNonMed*cSurg/urbanGini));

//Kabale Regional Referral Hospital: -1*15.057', 29*59.359'

add_regionals("Kabale",0,-1.25095,29.989316667,hospQuality.getCellNumericValue("Quality score!J16"), false, gamma(urbanGini, mRegCost*cSurg/urbanGini), gamma(urbanGini, mRegCost*mNonMed*cSurg/urbanGini));

//Lira Regional Referral Hospital: 2*15.088', 32*54.112'

add_regionals("Lira",0,2.251466667,32.901866667,hospQuality.getCellNumericValue("Quality score!J17"), false, gamma(urbanGini, mRegCost*cSurg/urbanGini), gamma(urbanGini, mRegCost*mNonMed*cSurg/urbanGini));

//Masaka Regional Referral Hospital: 0*19.766', 31*44.066'

add_regionals("Masaka",0,-0.329433333,31.734433333,hospQuality.getCellNumericValue("Quality score!J18"), false, gamma(urbanGini, mRegCost*cSurg/urbanGini), gamma(urbanGini, mRegCost*mNonMed*cSurg/urbanGini));

//Mbale Regional Referral Hospital: 1*4.589', 34*10.579'

add_regionals("Mbale",0,1.076483333,34.176316667,hospQuality.getCellNumericValue("Quality score!J19"), false, gamma(urbanGini, mRegCost*cSurg/urbanGini), gamma(urbanGini, mRegCost*mNonMed*cSurg/urbanGini));

//Moroto Regional Referral Hospital: 2*31.932', 34*39.670'

add_regionals("Moroto",0,2.5322,34.661166667,hospQuality.getCellNumericValue("Quality score!J20"), false, gamma(urbanGini, mRegCost*cSurg/urbanGini), gamma(urbanGini, mRegCost*mNonMed*cSurg/urbanGini));

//Mubende Regional Referral Hospital: 0*34.032', 31*23.614'

add_regionals("Mubende",0,0.5672,31.393566667,hospQuality.getCellNumericValue("Quality score!J21"), false, gamma(urbanGini, mRegCost*cSurg/urbanGini), gamma(urbanGini, mRegCost*mNonMed*cSurg/urbanGini));

//Soroti Regional Referral Hospital: 1*42.961', 33*36.789'

add_regionals("Soroti",0,1.716016667,33.61315,hospQuality.getCellNumericValue("Quality score!J22"), false, gamma(urbanGini, mRegCost*cSurg/urbanGini), gamma(urbanGini, mRegCost*mNonMed*cSurg/urbanGini));

for(Agent a : regionals) allHospitals.add(a);

for(Agent a : regionals) allHospitalsNGO.add(a);]]></Body>

</Function>

<Function AccessType="default" StaticFunction="false">

<ReturnModificator>VOID</ReturnModificator>

<ReturnType><![CDATA[void]]></ReturnType>

<Id>1390768856542</Id>

<Name><![CDATA[createDistricts]]></Name>

<X>-150</X><Y>120</Y>

<Label><X>10</X><Y>0</Y></Label>

<PublicFlag>false</PublicFlag>

<PresentationFlag>false</PresentationFlag>

<ShowLabel>true</ShowLabel>

<Body><![CDATA[//Each hospital: Name, quality, latitude, longitude

//DISTRICT HOSPITALS (Coordinates from Google Maps--in cases in which I could not find the hospital itself, it was placed at the "polling station" or in the center of its town)

//Abim Hospital: 2*42.501', 33*39.595' (approx)

add_districts("Abim",0,2.70835,33.659916667,hospQuality.getCellNumericValue("Quality score!J24"), false, gamma(urbanGini, mDistCost*cSurg/urbanGini), gamma(urbanGini, mDistCost*mNonMed*cSurg/urbanGini));

//Adjumani Hospital: 3*22.642', 31*47.483' (approx)

add_districts("Adjumani",0,3.377366667,31.791383333,hospQuality.getCellNumericValue("Quality score!J25"), false, gamma(urbanGini, mDistCost*cSurg/urbanGini), gamma(urbanGini, mDistCost*mNonMed*cSurg/urbanGini));

//Anaka Hospital: 2*35.828', 31*57.196' (approx)

add_districts("Anaka",0,2.597133333,31.953266667,hospQuality.getCellNumericValue("Quality score!J26"), false, gamma(urbanGini, mDistCost*cSurg/urbanGini), gamma(urbanGini, mDistCost*mNonMed*cSurg/urbanGini));

//Apac Hospital: 1*58.717', 32*32.019'

add_districts("Apac",0,1.978616667,32.53365,hospQuality.getCellNumericValue("Quality score!J27"), false, gamma(urbanGini, mDistCost*cSurg/urbanGini), gamma(urbanGini, mDistCost*mNonMed*cSurg/urbanGini));

//Bugiri Hospital: 0*34.397', 33*44.591'

add_districts("Bugiri",0,0.5551871,33.7632179,hospQuality.getCellNumericValue("Quality score!J28"), false, gamma(urbanGini, mDistCost*cSurg/urbanGini), gamma(urbanGini, mDistCost*mNonMed*cSurg/urbanGini));

//Bududa Hospital: 1*0.536', 34*19.967'

add_districts("Bududa",0,1.0088394,34.3330049,hospQuality.getCellNumericValue("Quality score!J29"), false, gamma(urbanGini, mDistCost*cSurg/urbanGini), gamma(urbanGini, mDistCost*mNonMed*cSurg/urbanGini));

//Bundibugyo Hospital: 0*42.648', 30*3.700'

add_districts("Bundibugyo",0,0.7108,30.061666667,hospQuality.getCellNumericValue("Quality score!J30"), false, gamma(urbanGini, mDistCost*cSurg/urbanGini), gamma(urbanGini, mDistCost*mNonMed*cSurg/urbanGini));

//Busolwe Hospital: 0*50.951', 33*55.771'

add_districts("Busolwe",0,0.849513,33.9294304,hospQuality.getCellNumericValue("Quality score!J31"), false, gamma(urbanGini, mDistCost*cSurg/urbanGini), gamma(urbanGini, mDistCost*mNonMed*cSurg/urbanGini));

//Entebbe Hospital: 0*3.547', 32*28.275'

add_districts("Entebbe",0,0.057447,32.4667273,hospQuality.getCellNumericValue("Quality score!J32"), false, gamma(urbanGini, mDistCost*cSurg/urbanGini), gamma(urbanGini, mDistCost*mNonMed*cSurg/urbanGini));

//Gombe Hospital: 0*29.041', 32*28.677'

add_districts("Gombe",0,0.4843081,32.4777691,hospQuality.getCellNumericValue("Quality score!J33"), false, gamma(urbanGini, mDistCost*cSurg/urbanGini), gamma(urbanGini, mDistCost*mNonMed*cSurg/urbanGini));

//Iganga Hospital: 0*36.967', 33*29.081'

add_districts("Iganga",0,0.6155886,33.4810834,hospQuality.getCellNumericValue("Quality score!J34"), false, gamma(urbanGini, mDistCost*cSurg/urbanGini), gamma(urbanGini, mDistCost*mNonMed*cSurg/urbanGini));

//Itojo Hospital: -0*47'15", 30*16'49"

add_districts("Itojo",0,-0.7887967,30.275504,hospQuality.getCellNumericValue("Quality score!J35"), false, gamma(urbanGini, mDistCost*cSurg/urbanGini), gamma(urbanGini, mDistCost*mNonMed*cSurg/urbanGini));

//Kaabong Hospital: 3*30.694', 34*8.017' (approx)

add_districts("Kaabong",0,3.511566667,34.133616667,hospQuality.getCellNumericValue("Quality score!J36"), false, gamma(urbanGini, mDistCost*cSurg/urbanGini), gamma(urbanGini, mDistCost*mNonMed*cSurg/urbanGini));

//Kabarole Hospital: (apparently also in Fort Portal) 0*35.3704', 30*15.312'

add_districts("Kabarole",0,0.589507,30.2551997,hospQuality.getCellNumericValue("Quality score!J37"), false, gamma(urbanGini, mDistCost*cSurg/urbanGini), gamma(urbanGini, mDistCost*mNonMed*cSurg/urbanGini));

//Kagadi Hospital: 0*56.516', 30*48.544'

add_districts("Kagadi",0,0.9420515,30.808904,hospQuality.getCellNumericValue("Quality score!J38"), false, gamma(urbanGini, mDistCost*cSurg/urbanGini), gamma(urbanGini, mDistCost*mNonMed*cSurg/urbanGini));

//Kalisizo Hospital: -0*32.127', 31*37.289' (approx)

add_districts("Kalisizo",0,-0.53545,31.621483333,hospQuality.getCellNumericValue("Quality score!J39"), false, gamma(urbanGini, mDistCost*cSurg/urbanGini), gamma(urbanGini, mDistCost*mNonMed*cSurg/urbanGini));

//Kambuga Hospital: -0*48.848', 29*48.030'

add_districts("Kambuga",0,-0.808066667,29.8005,hospQuality.getCellNumericValue("Quality score!J40"), false, gamma(urbanGini, mDistCost*cSurg/urbanGini), gamma(urbanGini, mDistCost*mNonMed*cSurg/urbanGini));

//Kamuli Hospital: 0*56.850', 33*7.302'

add_districts("Kamuli",0,0.9473258,33.1211856,hospQuality.getCellNumericValue("Quality score!J41"), false, gamma(urbanGini, mDistCost*cSurg/urbanGini), gamma(urbanGini, mDistCost*mNonMed*cSurg/urbanGini));

//Kapchorwa Hospital: 1*23.888', 34*26.837'

add_districts("Kapchorwa",0,1.3984078,34.4471383,hospQuality.getCellNumericValue("Quality score!J42"), false, gamma(urbanGini, mDistCost*cSurg/urbanGini), gamma(urbanGini, mDistCost*mNonMed*cSurg/urbanGini));

//Kasese Hospital: 0*10.990', 30*4.997'

add_districts("Kasese",0,0.183166667,30.083283333,hospQuality.getCellNumericValue("Quality score!J43"), false, gamma(urbanGini, mDistCost*cSurg/urbanGini), gamma(urbanGini, mDistCost*mNonMed*cSurg/urbanGini));

//Kawolo Hospital: 0*22.066', 32*56.714'

add_districts("Kawolo",0,0.3676526,32.9451656,hospQuality.getCellNumericValue("Quality score!J44"), false, gamma(urbanGini, mDistCost*cSurg/urbanGini), gamma(urbanGini, mDistCost*mNonMed*cSurg/urbanGini));

//Kayunga Hospital: 0*42.14175', 32*54.249138'

add_districts("Kayunga",0,0.703279,32.9036558,hospQuality.getCellNumericValue("Quality score!J45"), false, gamma(urbanGini, mDistCost*cSurg/urbanGini), gamma(urbanGini, mDistCost*mNonMed*cSurg/urbanGini));

//Kiboga Hospital: 0*54.641', 31*46.617'

add_districts("Kiboga",0,0.9176197,31.7722625,hospQuality.getCellNumericValue("Quality score!J46"), false, gamma(urbanGini, mDistCost*cSurg/urbanGini), gamma(urbanGini, mDistCost*mNonMed*cSurg/urbanGini));

//Kiryandongo Hospital: 1*57'00", 32*08'24" (approx)

add_districts("Kiryandongo",0,1.875366,32.0601654,hospQuality.getCellNumericValue("Quality score!J47"), false, gamma(urbanGini, mDistCost*cSurg/urbanGini), gamma(urbanGini, mDistCost*mNonMed*cSurg/urbanGini));

//Kisoro Hospital: -1*16.904', 29*41.521'

add_districts("Kisoro",0,-1.2816778,29.6918606,hospQuality.getCellNumericValue("Quality score!J48"), false, gamma(urbanGini, mDistCost*cSurg/urbanGini), gamma(urbanGini, mDistCost*mNonMed*cSurg/urbanGini));

//Kitagata Hospital: -0*40.371', 30*9.070'

add_districts("Kitagata",0,-0.6725646,30.1511192,hospQuality.getCellNumericValue("Quality score!J49"), false, gamma(urbanGini, mDistCost*cSurg/urbanGini), gamma(urbanGini, mDistCost*mNonMed*cSurg/urbanGini));

//Kitgum Hospital: 3*17.631', 32*52.880'

add_districts("Kitgum",0,3.2946098,32.8807792,hospQuality.getCellNumericValue("Quality score!J50"), false, gamma(urbanGini, mDistCost*cSurg/urbanGini), gamma(urbanGini, mDistCost*mNonMed*cSurg/urbanGini));

//Kumi Hospital: 1*29.274', 33*56.175'

add_districts("Kumi",0,1.5273264,33.9758849,hospQuality.getCellNumericValue("Quality score!J51"), false, gamma(urbanGini, mDistCost*cSurg/urbanGini), gamma(urbanGini, mDistCost*mNonMed*cSurg/urbanGini));

//Lyantonde Hospital: 0*24.346', 31*9.323'

add_districts("Lyantonde",0,0.405766667,31.155383333,hospQuality.getCellNumericValue("Quality score!J52"), false, gamma(urbanGini, mDistCost*cSurg/urbanGini), gamma(urbanGini, mDistCost*mNonMed*cSurg/urbanGini));

//Masindi Hospital: 1*41.111', 31*42.801'

add_districts("Masindi",0,1.6853108,31.7134094,hospQuality.getCellNumericValue("Quality score!J53"), false, gamma(urbanGini, mDistCost*cSurg/urbanGini), gamma(urbanGini, mDistCost*mNonMed*cSurg/urbanGini));

//Mityana Hospital: 0*23.813', 32*2.545'

add_districts("Mityana",0,0.3969369,32.0423824,hospQuality.getCellNumericValue("Quality score!J54"), false, gamma(urbanGini, mDistCost*cSurg/urbanGini), gamma(urbanGini, mDistCost*mNonMed*cSurg/urbanGini));

//Moyo Hospital: 3*39.244', 31*43.359'

add_districts("Moyo",0,3.654066667,31.72265,hospQuality.getCellNumericValue("Quality score!J55"), false, gamma(urbanGini, mDistCost*cSurg/urbanGini), gamma(urbanGini, mDistCost*mNonMed*cSurg/urbanGini));

//Mpigi Hospital: 0*13.224', 32*19.740'

add_districts("Mpigi",0,0.2269787,32.3281288,hospQuality.getCellNumericValue("Quality score!J56"), false, gamma(urbanGini, mDistCost*cSurg/urbanGini), gamma(urbanGini, mDistCost*mNonMed*cSurg/urbanGini));

//Nakaseke Hospital: 0*43.081', 32*23.942'

add_districts("Nakaseke",0,0.7179026,32.3989095,hospQuality.getCellNumericValue("Quality score!J57"), false, gamma(urbanGini, mDistCost*cSurg/urbanGini), gamma(urbanGini, mDistCost*mNonMed*cSurg/urbanGini));

//Nebbi Hospital: 2.4771921,31.0857134

add_districts("Nebbi",0,2.4771921,31.0857134,hospQuality.getCellNumericValue("Quality score!J58"), false, gamma(urbanGini, mDistCost*cSurg/urbanGini), gamma(urbanGini, mDistCost*mNonMed*cSurg/urbanGini));

//Pallisa Hospital: 1.175165,33.7120843

add_districts("Pallisa",0,1.175165,33.7120843,hospQuality.getCellNumericValue("Quality score!J59"), false, gamma(urbanGini, mDistCost*cSurg/urbanGini), gamma(urbanGini, mDistCost*mNonMed*cSurg/urbanGini));

//Rakai Hospital: -0.7053254,31.4025186

add_districts("Rakai",0,-0.7053254,31.4025186,hospQuality.getCellNumericValue("Quality score!J60"), false, gamma(urbanGini, mDistCost*cSurg/urbanGini), gamma(urbanGini, mDistCost*mNonMed*cSurg/urbanGini));

//Tororo Hospital: 0.6912488,34.187207

add_districts("Tororo",0,0.6912488,34.187207,hospQuality.getCellNumericValue("Quality score!J61"), false, gamma(urbanGini, mDistCost*cSurg/urbanGini), gamma(urbanGini, mDistCost*mNonMed*cSurg/urbanGini));

//Yumbe Hospital: 3*27.820', 31*14.757' (approx)

add_districts("Yumbe",0,3.463666667,31.24595,hospQuality.getCellNumericValue("Quality score!J62"), false, gamma(urbanGini, mDistCost*cSurg/urbanGini), gamma(urbanGini, mDistCost*mNonMed*cSurg/urbanGini));

for(Agent a : districts) allHospitals.add(a);

for(Agent a : districts) allHospitalsNGO.add(a);]]></Body>

</Function>

<Function AccessType="default" StaticFunction="false">

<ReturnModificator>VOID</ReturnModificator>

<ReturnType><![CDATA[void]]></ReturnType>

<Id>1390777163517</Id>

<Name><![CDATA[createPeople]]></Name>

<Description><![CDATA[Creates the initial population

The patient is placed at that GPS coordinate with dispersion distributed normally around a 2mi radius for Kampala, placementRadius/2 for the other towns and placementRadius*8 for the rural districts]]></Description>

<X>-150</X><Y>140</Y>

<Label><X>10</X><Y>0</Y></Label>

<PublicFlag>false</PublicFlag>

<PresentationFlag>false</PresentationFlag>

<ShowLabel>true</ShowLabel>

<Parameter>

<Name><![CDATA[size]]></Name>

<Type><![CDATA[int]]></Type>

</Parameter>

<Body><![CDATA[double dispersion = placementRadius/1.15/60/2;

//this sets up a radius of X miles from the center latitude for 95% of patients

//two standard deviations = miles/1.15/60 degrees, so sigma is half that

//**NOTE: 1 degree = 69 miles; 1 minute = 1.15 miles; 1 second = 100 feet

int rownum = 2;

int pick = 0;

int adults = roundToInt(size*(1.0 - propKids)); //see Assumptions file for this calculation

//create adults

for(int i = 0; i < adults; i++){

double r = uniform(0,1);

if (r < 0.303941182) pick = (int) rowPicker1(r);

else if (r < 0.495710003) pick = (int) rowPicker2(r);

else if (r < 0.684744908) pick = (int) rowPicker3(r);

else if (r < 0.866907023) pick = (int) rowPicker4(r);

else pick = (int) rowPicker5(r);

//find the right row

int k = pick + 20;

if(k == 22) k = 20;

for(int j = pick; j < k; j++) {

if(r > GPSandPopulation.getCellNumericValue(1,j,10)) rownum = j;

else break;

}

//find the GPS coordinates and place the patient

double placementLat = GPSandPopulation.getCellNumericValue(1,rownum,6);

double placementLong = GPSandPopulation.getCellNumericValue(1,rownum,7);

double disperseLat = normal(dispersion*uniform(0.5,5),0);

double disperseLong = normal(dispersion*uniform(0.5,5),0);

if(Uganda.getLayers()[1].findPoliticalArea(placementLong + disperseLong, placementLat + disperseLat) == null) { //makes sure the point is on the map

disperseLat = 0;

disperseLong = 0;

}

String reg = "Central";

if(Uganda.getLayers()[2].findPoliticalArea(placementLong + disperseLong, placementLat + disperseLat) != null) {

reg = Uganda.getLayers()[2].findPoliticalArea(placementLong + disperseLong, placementLat + disperseLat).name;

}

int regnum = 1000;

if(reg.equals("Central")) {

regnum = 1;

} else if(reg.equals("Northern")) {

regnum = 2;

} else if(reg.equals("Eastern")) {

regnum = 3;

} else if(reg.equals("Western")) {

regnum = 4;

}

if(Uganda.getLayers()[1].findPoliticalArea(placementLong + disperseLong, placementLat + disperseLat) != null && Uganda.getLayers()[1].findPoliticalArea(placementLong + disperseLong, placementLat + disperseLat).name.equalsIgnoreCase("kampala city council")) {

regnum = 5;

}

double pUrban = 0.185413809; //See Income by region excel spreadsheet for these calculations

switch(regnum){

case 1:

pUrban = 0.197752809; //Central

break;

case 2:

pUrban = 0.099180328; //Northern

break;

case 3:

pUrban = 0.095896947; //Eastern

break;

case 4:

pUrban = 0.106253177; //Western

break;

case 5:

pUrban = 1.0; //Kampala

break;

default:

pUrban = 0.185413809; //Uganda as a whole

}

//int urban1_0 = (int) GPSandPopulation.getCellNumericValue(1,rownum,11);

//boolean urban = (urban1_0 == 1 ? true : false);

boolean urban = randomTrue(pUrban);

Person p = add_persons(placementLat + disperseLat, placementLong + disperseLong, randomTrue(0.5), 2, urban, 0, false, false);

p.region = regnum;

//latitude, longitude, male?, starting state (0 = U5, 1 = Youth, 2 = Adult), urban?, quintile, poor?, HOH?,

//Note that education is going to be set in the startup code of each agent.

}

//assign villages

applyNetwork();

for(Person p : persons) {

p.spouse.disconnect();

p.father.disconnect();

p.mother.disconnect();

p.kids.disconnectFromAll();

Collection c = new LinkedList<Object>();

for(Object a : p.connections.getConnections()) {

if(hospitals.contains(a) || districts.contains(a) || regionals.contains(a)) c.add(a);

}

for(Object v : c) {

Agent removal = (Agent) v;

p.connections.disconnectFrom(removal);

}

}

//assign spouses

for(Person p : persons) {

if(!p.male) {

for(Person v : p.connections.getConnections()) {

if(v.statechart.isStateActive(v.Male) && !v.spouse.isConnected()) {

p.spouse.connectTo(v);

break;

}

}

}

}

//assign HOH and microeconomic variables <-- CAN YOU COMBINE THIS LOOP WITH THE ONE ABOVE?

for(Person p : persons) {

double pickGini = p.urban ? urbanGini : ruralGini;

double pickGDP = 303700.0; //Uganda as a whole

if(p.urban) {

switch(p.region){

case 1:

pickGDP = 603800.0; //Central

break;

case 2:

pickGDP = 361200.0; //Northern

break;

case 3:

pickGDP = 361000.0; //Eastern

break;

case 4:

pickGDP = 479000.0; //Western

break;

case 5:

pickGDP = 959400.0; //Kampala

break;

default:

pickGDP = 660000.0; //urban Uganda as a whole

}

} else {

switch(p.region){

case 1:

pickGDP = 336800.0; //Central

break;

case 2:

pickGDP = 117200.0; //Northern

break;

case 3:

pickGDP = 151400.0; //Eastern

break;

case 4:

pickGDP = 282300.0; //Western

break;

case 5:

pickGDP = 222600.0; //Kampala

break;

default:

pickGDP = 222600.0; //rural Uganda as a whole

}

}

if(p.male && p.spouse.isConnected()) {

Person wife = p.spouse.getConnectedAgent();

p.HOH = true; //assumes that married couples have male HOH (see LyX file for reasoning)

wife.HOH = false;

p.wealth = startIncome(pickGDP, pickGini); //prevents zero income (which will error out in the choice function)

wife.wealth = p.wealth;

wife.poor = p.poor;

} else {

p.HOH = true;

p.wealth = startIncome(pickGDP, pickGini);

}

}

//figure out quintiles and assign them

double[] incomeArray = new double[persons.size()];

int pline = roundToInt(povPct * persons.size());

int q1 = roundToInt(0.2 * persons.size());

int q2 = roundToInt(0.4 * persons.size());

int q3 = roundToInt(0.6 * persons.size());

int q4 = roundToInt(0.8 * persons.size());

for(Person p : persons) {

incomeArray[p.getIndex()] = p.wealth;

}

Arrays.sort(incomeArray);

povertyLine = incomeArray[pline];

cutoff1 = incomeArray[q1];

cutoff2 = incomeArray[q2];

cutoff3 = incomeArray[q3];

cutoff4 = incomeArray[q4];

for(Person p : persons) {

if(p.wealth <= povertyLine) p.poor = true;

if(p.wealth > cutoff4) p.quintile = 5;

else if(p.wealth > cutoff3) p.quintile = 4;

else if(p.wealth > cutoff2) p.quintile = 3;

else if(p.wealth > cutoff1) p.quintile = 2;

else p.quintile = 1;

if(p.male && p.spouse.isConnected()) {

Person wife = p.spouse.getConnectedAgent();

wife.poor = p.poor;

wife.quintile = p.quintile;

}

}

//create kids per woman--average 3-4 (mean HH size in Uganda is 5)

for(int i = 0; i < Females.size(); i++) {

Person p = Females.get(i);

int kidnum = roundToInt(triangular(0,7)); //have an average of 3.5 kids

p.deliver(kidnum, p);

}

]]></Body>

</Function>

<Function AccessType="default" StaticFunction="false">

<ReturnModificator>RETURNS_VALUE</ReturnModificator>

<ReturnType><![CDATA[double]]></ReturnType>

<Id>1393272265244</Id>

<Name><![CDATA[startIncome]]></Name>

<X>-150</X><Y>170</Y>

<Label><X>10</X><Y>0</Y></Label>

<PublicFlag>false</PublicFlag>

<PresentationFlag>false</PresentationFlag>

<ShowLabel>true</ShowLabel>

<Parameter>

<Name><![CDATA[GDP]]></Name>

<Type><![CDATA[double]]></Type>

</Parameter>

<Parameter>

<Name><![CDATA[Gini]]></Name>

<Type><![CDATA[double]]></Type>

</Parameter>

<Body><![CDATA[//convert UGX to USD

double conversion = excelIncomeCalc.getCellNumericValue(1,1,5) * excelIncomeCalc.getCellNumericValue(1,18,6);

double inc = (double) GDP * conversion * 12.0 / famSize;

double shape = (double) Gini;

double scale = (double) inc/Gini;

double wealth = gamma(shape, scale);

return wealth + 1; //prevents zero wealth (which will error out in the choice function)]]></Body>

</Function>

</Functions>

<TableFunctions>

<TableFunction AccessType="public" StaticFunction="true">

<Id>1391204206647</Id>

<Name><![CDATA[rowPicker1]]></Name>

<X>-500</X><Y>260</Y>

<Label><X>10</X><Y>0</Y></Label>

<PublicFlag>false</PublicFlag>

<PresentationFlag>false</PresentationFlag>

<ShowLabel>true</ShowLabel>

<InterpolationMethod>STEP</InterpolationMethod>

<OutOfRangeBehaviour>NEAREST</OutOfRangeBehaviour>

<OutOfRangeCustomValue><![CDATA[0.0]]></OutOfRangeCustomValue>

<ApproximationOrder><![CDATA[1]]></ApproximationOrder>

// arguments and values

<Argument><![CDATA[0.0]]></Argument>

<Argument><![CDATA[0.080555494]]></Argument>

<Argument><![CDATA[0.096548374]]></Argument>

<Argument><![CDATA[0.119237805]]></Argument>

<Argument><![CDATA[0.141389086]]></Argument>

<Argument><![CDATA[0.163339011]]></Argument>

<Argument><![CDATA[0.186684961]]></Argument>

<Argument><![CDATA[0.208651414]]></Argument>

<Argument><![CDATA[0.220012963]]></Argument>

<Argument><![CDATA[0.237115808]]></Argument>

<Argument><![CDATA[0.275824038]]></Argument>

<Value><![CDATA[2.0]]></Value>

<Value><![CDATA[20.0]]></Value>

<Value><![CDATA[40.0]]></Value>

<Value><![CDATA[60.0]]></Value>

<Value><![CDATA[80.0]]></Value>

<Value><![CDATA[100.0]]></Value>

<Value><![CDATA[120.0]]></Value>

<Value><![CDATA[140.0]]></Value>

<Value><![CDATA[160.0]]></Value>

<Value><![CDATA[180.0]]></Value>

<Value><![CDATA[200.0]]></Value>

</TableFunction>

<TableFunction AccessType="public" StaticFunction="true">

<Id>1391204231337</Id>

<Name><![CDATA[rowPicker2]]></Name>

<X>-500</X><Y>280</Y>

<Label><X>10</X><Y>0</Y></Label>

<PublicFlag>false</PublicFlag>

<PresentationFlag>false</PresentationFlag>

<ShowLabel>true</ShowLabel>

<InterpolationMethod>STEP</InterpolationMethod>

<OutOfRangeBehaviour>NEAREST</OutOfRangeBehaviour>

<OutOfRangeCustomValue><![CDATA[0.0]]></OutOfRangeCustomValue>

<ApproximationOrder><![CDATA[1]]></ApproximationOrder>

// arguments and values

<Argument><![CDATA[0.303941182]]></Argument>

<Argument><![CDATA[0.325061094]]></Argument>

<Argument><![CDATA[0.348936796]]></Argument>

<Argument><![CDATA[0.374679909]]></Argument>

<Argument><![CDATA[0.389506291]]></Argument>

<Argument><![CDATA[0.40811464]]></Argument>

<Argument><![CDATA[0.426867584]]></Argument>

<Argument><![CDATA[0.445586097]]></Argument>

<Argument><![CDATA[0.461006321]]></Argument>

<Argument><![CDATA[0.476205987]]></Argument>

<Value><![CDATA[220.0]]></Value>

<Value><![CDATA[240.0]]></Value>

<Value><![CDATA[260.0]]></Value>

<Value><![CDATA[280.0]]></Value>

<Value><![CDATA[300.0]]></Value>

<Value><![CDATA[320.0]]></Value>

<Value><![CDATA[340.0]]></Value>

<Value><![CDATA[360.0]]></Value>

<Value><![CDATA[380.0]]></Value>

<Value><![CDATA[400.0]]></Value>

</TableFunction>

<TableFunction AccessType="public" StaticFunction="true">

<Id>1391204232182</Id>

<Name><![CDATA[rowPicker3]]></Name>

<X>-500</X><Y>300</Y>

<Label><X>10</X><Y>0</Y></Label>

<PublicFlag>false</PublicFlag>

<PresentationFlag>false</PresentationFlag>

<ShowLabel>true</ShowLabel>

<InterpolationMethod>STEP</InterpolationMethod>

<OutOfRangeBehaviour>NEAREST</OutOfRangeBehaviour>

<OutOfRangeCustomValue><![CDATA[0.0]]></OutOfRangeCustomValue>

<ApproximationOrder><![CDATA[1]]></ApproximationOrder>

// arguments and values

<Argument><![CDATA[0.495710003]]></Argument>

<Argument><![CDATA[0.51304543]]></Argument>

<Argument><![CDATA[0.534363646]]></Argument>

<Argument><![CDATA[0.54864562]]></Argument>

<Argument><![CDATA[0.57241192]]></Argument>

<Argument><![CDATA[0.592063967]]></Argument>

<Argument><![CDATA[0.609422068]]></Argument>

<Argument><![CDATA[0.626742189]]></Argument>

<Argument><![CDATA[0.639216528]]></Argument>

<Argument><![CDATA[0.66164635]]></Argument>

<Value><![CDATA[420.0]]></Value>

<Value><![CDATA[440.0]]></Value>

<Value><![CDATA[460.0]]></Value>

<Value><![CDATA[480.0]]></Value>

<Value><![CDATA[500.0]]></Value>

<Value><![CDATA[520.0]]></Value>

<Value><![CDATA[540.0]]></Value>

<Value><![CDATA[560.0]]></Value>

<Value><![CDATA[580.0]]></Value>

<Value><![CDATA[600.0]]></Value>

</TableFunction>

<TableFunction AccessType="public" StaticFunction="true">

<Id>1391204232646</Id>

<Name><![CDATA[rowPicker4]]></Name>

<X>-500</X><Y>320</Y>

<Label><X>10</X><Y>0</Y></Label>

<PublicFlag>false</PublicFlag>

<PresentationFlag>false</PresentationFlag>

<ShowLabel>true</ShowLabel>

<InterpolationMethod>STEP</InterpolationMethod>

<OutOfRangeBehaviour>NEAREST</OutOfRangeBehaviour>

<OutOfRangeCustomValue><![CDATA[0.0]]></OutOfRangeCustomValue>

<ApproximationOrder><![CDATA[1]]></ApproximationOrder>

// arguments and values

<Argument><![CDATA[0.684744908]]></Argument>

<Argument><![CDATA[0.699523919]]></Argument>

<Argument><![CDATA[0.718551205]]></Argument>

<Argument><![CDATA[0.731923387]]></Argument>

<Argument><![CDATA[0.750515322]]></Argument>

<Argument><![CDATA[0.768425886]]></Argument>

<Argument><![CDATA[0.788667653]]></Argument>

<Argument><![CDATA[0.806888708]]></Argument>

<Argument><![CDATA[0.828664263]]></Argument>

<Argument><![CDATA[0.848092278]]></Argument>

<Value><![CDATA[620.0]]></Value>

<Value><![CDATA[640.0]]></Value>

<Value><![CDATA[660.0]]></Value>

<Value><![CDATA[680.0]]></Value>

<Value><![CDATA[700.0]]></Value>

<Value><![CDATA[720.0]]></Value>

<Value><![CDATA[740.0]]></Value>

<Value><![CDATA[760.0]]></Value>

<Value><![CDATA[780.0]]></Value>

<Value><![CDATA[800.0]]></Value>

</TableFunction>

<TableFunction AccessType="public" StaticFunction="true">

<Id>1391204517886</Id>

<Name><![CDATA[rowPicker5]]></Name>

<X>-500</X><Y>340</Y>

<Label><X>10</X><Y>0</Y></Label>

<PublicFlag>false</PublicFlag>

<PresentationFlag>false</PresentationFlag>

<ShowLabel>true</ShowLabel>

<InterpolationMethod>STEP</InterpolationMethod>

<OutOfRangeBehaviour>NEAREST</OutOfRangeBehaviour>

<OutOfRangeCustomValue><![CDATA[0.0]]></OutOfRangeCustomValue>

<ApproximationOrder><![CDATA[1]]></ApproximationOrder>

// arguments and values

<Argument><![CDATA[0.866907023]]></Argument>

<Argument><![CDATA[0.880298482]]></Argument>

<Argument><![CDATA[0.905277239]]></Argument>

<Argument><![CDATA[0.924742664]]></Argument>

<Argument><![CDATA[0.941109858]]></Argument>

<Argument><![CDATA[0.961664215]]></Argument>

<Argument><![CDATA[0.982235787]]></Argument>

<Value><![CDATA[820.0]]></Value>

<Value><![CDATA[840.0]]></Value>

<Value><![CDATA[860.0]]></Value>

<Value><![CDATA[880.0]]></Value>

<Value><![CDATA[900.0]]></Value>

<Value><![CDATA[920.0]]></Value>

<Value><![CDATA[940.0]]></Value>

</TableFunction>

<TableFunction AccessType="public" StaticFunction="false">

<Id>1391289796106</Id>

<Name><![CDATA[maleLifeTable]]></Name>

<X>-360</X><Y>429</Y>

<Label><X>10</X><Y>0</Y></Label>

<PublicFlag>false</PublicFlag>

<PresentationFlag>false</PresentationFlag>

<ShowLabel>true</ShowLabel>

<InterpolationMethod>LINEAR</InterpolationMethod>

<OutOfRangeBehaviour>NEAREST</OutOfRangeBehaviour>

<OutOfRangeCustomValue><![CDATA[0.0]]></OutOfRangeCustomValue>

<ApproximationOrder><![CDATA[1]]></ApproximationOrder>

// arguments and values

<Argument><![CDATA[100.0]]></Argument>

<Value><![CDATA[1000000.0]]></Value>

</TableFunction>

<TableFunction AccessType="public" StaticFunction="false">

<Id>1391289815329</Id>

<Name><![CDATA[femaleLifeTable]]></Name>

<X>-360</X><Y>449</Y>

<Label><X>10</X><Y>0</Y></Label>

<PublicFlag>false</PublicFlag>

<PresentationFlag>false</PresentationFlag>

<ShowLabel>true</ShowLabel>

<InterpolationMethod>LINEAR</InterpolationMethod>

<OutOfRangeBehaviour>NEAREST</OutOfRangeBehaviour>

<OutOfRangeCustomValue><![CDATA[0.0]]></OutOfRangeCustomValue>

<ApproximationOrder><![CDATA[1]]></ApproximationOrder>

// arguments and values

<Argument><![CDATA[100.0]]></Argument>

<Value><![CDATA[1000000.0]]></Value>

</TableFunction>

<TableFunction AccessType="public" StaticFunction="true">

<Id>1392333422494</Id>

<Name><![CDATA[maleBackoutMort]]></Name>

<X>-510</X><Y>450</Y>

<Label><X>10</X><Y>0</Y></Label>

<PublicFlag>false</PublicFlag>

<PresentationFlag>false</PresentationFlag>

<ShowLabel>true</ShowLabel>

<InterpolationMethod>LINEAR</InterpolationMethod>

<OutOfRangeBehaviour>NEAREST</OutOfRangeBehaviour>

<OutOfRangeCustomValue><![CDATA[0.0]]></OutOfRangeCustomValue>

<ApproximationOrder><![CDATA[1]]></ApproximationOrder>

// arguments and values

<Argument><![CDATA[100.0]]></Argument>

<Value><![CDATA[1000000.0]]></Value>

</TableFunction>

<TableFunction AccessType="public" StaticFunction="true">

<Id>1392333885143</Id>

<Name><![CDATA[femaleBackoutMort]]></Name>

<X>-510</X><Y>470</Y>

<Label><X>10</X><Y>0</Y></Label>

<PublicFlag>false</PublicFlag>

<PresentationFlag>false</PresentationFlag>

<ShowLabel>true</ShowLabel>

<InterpolationMethod>LINEAR</InterpolationMethod>

<OutOfRangeBehaviour>NEAREST</OutOfRangeBehaviour>

<OutOfRangeCustomValue><![CDATA[0.0]]></OutOfRangeCustomValue>

<ApproximationOrder><![CDATA[1]]></ApproximationOrder>

// arguments and values

<Argument><![CDATA[100.0]]></Argument>

<Value><![CDATA[1000000.0]]></Value>

</TableFunction>

<TableFunction AccessType="public" StaticFunction="true">

<Id>1392669437767</Id>

<Name><![CDATA[maleInitLifeTable]]></Name>

<X>-360</X><Y>469</Y>

<Label><X>10</X><Y>0</Y></Label>

<PublicFlag>false</PublicFlag>

<PresentationFlag>false</PresentationFlag>

<ShowLabel>true</ShowLabel>

<InterpolationMethod>STEP</InterpolationMethod>

<OutOfRangeBehaviour>NEAREST</OutOfRangeBehaviour>

<OutOfRangeCustomValue><![CDATA[0.0]]></OutOfRangeCustomValue>

<ApproximationOrder><![CDATA[1]]></ApproximationOrder>

// arguments and values

</TableFunction>

<TableFunction AccessType="public" StaticFunction="true">

<Id>1392669467785</Id>

<Name><![CDATA[femaleInitLifeTable]]></Name>

<X>-360</X><Y>489</Y>

<Label><X>10</X><Y>0</Y></Label>

<PublicFlag>false</PublicFlag>

<PresentationFlag>false</PresentationFlag>

<ShowLabel>true</ShowLabel>

<InterpolationMethod>STEP</InterpolationMethod>

<OutOfRangeBehaviour>NEAREST</OutOfRangeBehaviour>

<OutOfRangeCustomValue><![CDATA[0.0]]></OutOfRangeCustomValue>

<ApproximationOrder><![CDATA[1]]></ApproximationOrder>

// arguments and values

</TableFunction>

<TableFunction AccessType="public" StaticFunction="true">

<Id>1410896576017</Id>

<Name><![CDATA[fertility]]></Name>

<X>-500</X><Y>210</Y>

<Label><X>10</X><Y>0</Y></Label>

<PublicFlag>false</PublicFlag>

<PresentationFlag>false</PresentationFlag>

<ShowLabel>true</ShowLabel>

<InterpolationMethod>STEP</InterpolationMethod>

<OutOfRangeBehaviour>NEAREST</OutOfRangeBehaviour>

<OutOfRangeCustomValue><![CDATA[0.0]]></OutOfRangeCustomValue>

<ApproximationOrder><![CDATA[1]]></ApproximationOrder>

// arguments and values

<Argument><![CDATA[16.0]]></Argument>

<Argument><![CDATA[17.0]]></Argument>

<Argument><![CDATA[18.0]]></Argument>

<Argument><![CDATA[19.0]]></Argument>

<Argument><![CDATA[20.0]]></Argument>

<Argument><![CDATA[21.0]]></Argument>

<Argument><![CDATA[22.0]]></Argument>

<Argument><![CDATA[23.0]]></Argument>

<Argument><![CDATA[24.0]]></Argument>

<Argument><![CDATA[25.0]]></Argument>

<Argument><![CDATA[26.0]]></Argument>

<Argument><![CDATA[27.0]]></Argument>

<Argument><![CDATA[28.0]]></Argument>

<Argument><![CDATA[29.0]]></Argument>

<Argument><![CDATA[30.0]]></Argument>

<Argument><![CDATA[31.0]]></Argument>

<Argument><![CDATA[32.0]]></Argument>

<Argument><![CDATA[33.0]]></Argument>

<Argument><![CDATA[34.0]]></Argument>

<Argument><![CDATA[35.0]]></Argument>

<Argument><![CDATA[36.0]]></Argument>

<Argument><![CDATA[37.0]]></Argument>

<Argument><![CDATA[38.0]]></Argument>

<Argument><![CDATA[39.0]]></Argument>

<Argument><![CDATA[40.0]]></Argument>

<Argument><![CDATA[41.0]]></Argument>

<Argument><![CDATA[42.0]]></Argument>

<Argument><![CDATA[43.0]]></Argument>

<Argument><![CDATA[44.0]]></Argument>

<Argument><![CDATA[45.0]]></Argument>

<Argument><![CDATA[46.0]]></Argument>

<Argument><![CDATA[47.0]]></Argument>

<Argument><![CDATA[48.0]]></Argument>

<Argument><![CDATA[49.0]]></Argument>

<Argument><![CDATA[50.0]]></Argument>

<Value><![CDATA[4.78857E-4]]></Value>

<Value><![CDATA[4.7843E-4]]></Value>

<Value><![CDATA[4.77977E-4]]></Value>

<Value><![CDATA[4.77495E-4]]></Value>

<Value><![CDATA[4.76982E-4]]></Value>

<Value><![CDATA[4.76434E-4]]></Value>

<Value><![CDATA[4.75847E-4]]></Value>

<Value><![CDATA[4.75218E-4]]></Value>

<Value><![CDATA[4.74542E-4]]></Value>

<Value><![CDATA[4.73812E-4]]></Value>

<Value><![CDATA[4.73022E-4]]></Value>

<Value><![CDATA[4.72166E-4]]></Value>

<Value><![CDATA[4.71233E-4]]></Value>

<Value><![CDATA[4.70214E-4]]></Value>

<Value><![CDATA[4.69095E-4]]></Value>

<Value><![CDATA[4.67861E-4]]></Value>

<Value><![CDATA[4.66494E-4]]></Value>

<Value><![CDATA[4.64971E-4]]></Value>

<Value><![CDATA[4.63263E-4]]></Value>

<Value><![CDATA[4.61335E-4]]></Value>

<Value><![CDATA[4.59141E-4]]></Value>

<Value><![CDATA[4.56621E-4]]></Value>

<Value><![CDATA[4.53699E-4]]></Value>

<Value><![CDATA[4.50268E-4]]></Value>

<Value><![CDATA[4.46184E-4]]></Value>

<Value><![CDATA[4.4124E-4]]></Value>

<Value><![CDATA[4.35133E-4]]></Value>

<Value><![CDATA[4.27398E-4]]></Value>

<Value><![CDATA[4.17282E-4]]></Value>

<Value><![CDATA[4.03487E-4]]></Value>

<Value><![CDATA[3.83562E-4]]></Value>

<Value><![CDATA[3.52251E-4]]></Value>

<Value><![CDATA[2.95891E-4]]></Value>

<Value><![CDATA[1.64384E-4]]></Value>

<Value><![CDATA[0.0]]></Value>

</TableFunction>

</TableFunctions>

<CustomDistributions>

<CustomDistribution>

<Id>1411506805153</Id>

<Name><![CDATA[selectCancer]]></Name>

<X>-360</X><Y>550</Y>

<Label><X>15</X><Y>0</Y></Label>

<PublicFlag>false</PublicFlag>

<PresentationFlag>false</PresentationFlag>

<ShowLabel>true</ShowLabel>

<InterpolationMethod>NONE</InterpolationMethod>

<Type>CONTINUOUS</Type>

<DefinitionType>PDF</DefinitionType>

<Static>false</Static>

<Argument><![CDATA[1.0]]></Argument>

<Argument><![CDATA[2.0]]></Argument>

<Argument><![CDATA[3.0]]></Argument>

<Argument><![CDATA[4.0]]></Argument>

<Argument><![CDATA[5.0]]></Argument>

<Argument><![CDATA[6.0]]></Argument>

<Argument><![CDATA[7.0]]></Argument>

<Value><![CDATA[0.230434783]]></Value>

<Value><![CDATA[0.266183575]]></Value>

<Value><![CDATA[0.170048309]]></Value>

<Value><![CDATA[0.069082126]]></Value>

<Value><![CDATA[0.18115942]]></Value>

<Value><![CDATA[0.052898551]]></Value>

<Value><![CDATA[0.030193237]]></Value>

<OptionListID><![CDATA[0]]></OptionListID>

<OptionValues>

</OptionValues>

<Argument><![CDATA[1.0]]></Argument>

<Argument><![CDATA[2.0]]></Argument>

<Argument><![CDATA[3.0]]></Argument>

<Argument><![CDATA[4.0]]></Argument>

<Argument><![CDATA[5.0]]></Argument>

<Argument><![CDATA[6.0]]></Argument>

<Argument><![CDATA[7.0]]></Argument>

</CustomDistribution>

</CustomDistributions>

<AnalysisData>

<DataSet>

<Id>1410914596243</Id>

<Name><![CDATA[popOutput]]></Name>

<X>-1650</X><Y>50</Y>

<Label><X>15</X><Y>0</Y></Label>

<PublicFlag>false</PublicFlag>

<PresentationFlag>false</PresentationFlag>

<ShowLabel>true</ShowLabel>

<AutoUpdate>true</AutoUpdate>

<RecurrenceCode><![CDATA[365]]></RecurrenceCode>

<FreezeXAxis>false</FreezeXAxis>

<HorizontalAxisExpression><![CDATA[getYear()]]></HorizontalAxisExpression>

<VerticalAxisExpression><![CDATA[persons.nAlive()]]></VerticalAxisExpression>

<SamplesToKeep>100</SamplesToKeep>

</DataSet>

<DataSet>

<Id>1410914987857</Id>

<Name><![CDATA[cancIncSize]]></Name>

<X>-1650</X><Y>70</Y>

<Label><X>15</X><Y>0</Y></Label>

<PublicFlag>false</PublicFlag>

<PresentationFlag>false</PresentationFlag>

<ShowLabel>true</ShowLabel>

<AutoUpdate>true</AutoUpdate>

<RecurrenceCode><![CDATA[365]]></RecurrenceCode>

<FreezeXAxis>false</FreezeXAxis>

<HorizontalAxisExpression><![CDATA[getYear()]]></HorizontalAxisExpression>

<VerticalAxisExpression><![CDATA[cancerInc.size()]]></VerticalAxisExpression>

<SamplesToKeep>100</SamplesToKeep>

</DataSet>

<DataSet>

<Id>1410991915537</Id>

<Name><![CDATA[systCostDataset]]></Name>

<X>-1650</X><Y>110</Y>

<Label><X>15</X><Y>0</Y></Label>

<PublicFlag>false</PublicFlag>

<PresentationFlag>false</PresentationFlag>

<ShowLabel>true</ShowLabel>

<AutoUpdate>true</AutoUpdate>

<RecurrenceCode><![CDATA[365]]></RecurrenceCode>

<FreezeXAxis>false</FreezeXAxis>

<HorizontalAxisExpression><![CDATA[getYear()]]></HorizontalAxisExpression>

<VerticalAxisExpression><![CDATA[systemCost]]></VerticalAxisExpression>

<SamplesToKeep>100</SamplesToKeep>

</DataSet>

<DataSet>

<Id>1410992799751</Id>

<Name><![CDATA[cancTxSize]]></Name>

<X>-1650</X><Y>90</Y>

<Label><X>15</X><Y>0</Y></Label>

<PublicFlag>false</PublicFlag>

<PresentationFlag>false</PresentationFlag>

<ShowLabel>true</ShowLabel>

<AutoUpdate>true</AutoUpdate>

<RecurrenceCode><![CDATA[365]]></RecurrenceCode>

<FreezeXAxis>false</FreezeXAxis>

<HorizontalAxisExpression><![CDATA[getYear()]]></HorizontalAxisExpression>

<VerticalAxisExpression><![CDATA[cancerTx.size()]]></VerticalAxisExpression>

<SamplesToKeep>100</SamplesToKeep>

</DataSet>

<DataSet>

<Id>1411573508713</Id>

<Name><![CDATA[dsMadePoorRich]]></Name>

<X>-1330</X><Y>320</Y>

<Label><X>15</X><Y>0</Y></Label>

<PublicFlag>false</PublicFlag>

<PresentationFlag>false</PresentationFlag>

<ShowLabel>true</ShowLabel>

<AutoUpdate>true</AutoUpdate>

<RecurrenceCode><![CDATA[365]]></RecurrenceCode>

<FreezeXAxis>false</FreezeXAxis>

<HorizontalAxisExpression><![CDATA[getYear()]]></HorizontalAxisExpression>

<VerticalAxisExpression><![CDATA[madePoorRich]]></VerticalAxisExpression>

<SamplesToKeep>100</SamplesToKeep>

</DataSet>

<DataSet>

<Id>1411575457099</Id>

<Name><![CDATA[dsPoorest]]></Name>

<X>-1510</X><Y>50</Y>

<Label><X>15</X><Y>0</Y></Label>

<PublicFlag>false</PublicFlag>

<PresentationFlag>false</PresentationFlag>

<ShowLabel>true</ShowLabel>

<AutoUpdate>true</AutoUpdate>

<RecurrenceCode><![CDATA[365]]></RecurrenceCode>

<FreezeXAxis>false</FreezeXAxis>

<HorizontalAxisExpression><![CDATA[getYear()]]></HorizontalAxisExpression>

<VerticalAxisExpression><![CDATA[persons.nQuint1()]]></VerticalAxisExpression>

<SamplesToKeep>100</SamplesToKeep>

</DataSet>

<DataSet>

<Id>1411575506495</Id>

<Name><![CDATA[dsRichest]]></Name>

<X>-1510</X><Y>130</Y>

<Label><X>15</X><Y>0</Y></Label>

<PublicFlag>false</PublicFlag>

<PresentationFlag>false</PresentationFlag>

<ShowLabel>true</ShowLabel>

<AutoUpdate>true</AutoUpdate>

<RecurrenceCode><![CDATA[365]]></RecurrenceCode>

<FreezeXAxis>false</FreezeXAxis>

<HorizontalAxisExpression><![CDATA[getYear()]]></HorizontalAxisExpression>

<VerticalAxisExpression><![CDATA[persons.nQuint5()]]></VerticalAxisExpression>

<SamplesToKeep>100</SamplesToKeep>

</DataSet>

<DataSet>

<Id>1411575506977</Id>

<Name><![CDATA[dsRich]]></Name>

<X>-1510</X><Y>110</Y>

<Label><X>15</X><Y>0</Y></Label>

<PublicFlag>false</PublicFlag>

<PresentationFlag>false</PresentationFlag>

<ShowLabel>true</ShowLabel>

<AutoUpdate>true</AutoUpdate>

<RecurrenceCode><![CDATA[365]]></RecurrenceCode>

<FreezeXAxis>false</FreezeXAxis>

<HorizontalAxisExpression><![CDATA[getYear()]]></HorizontalAxisExpression>

<VerticalAxisExpression><![CDATA[persons.nQuint4()]]></VerticalAxisExpression>

<SamplesToKeep>100</SamplesToKeep>

</DataSet>

<DataSet>

<Id>1411575507611</Id>

<Name><![CDATA[dsMiddle]]></Name>

<X>-1510</X><Y>90</Y>

<Label><X>15</X><Y>0</Y></Label>

<PublicFlag>false</PublicFlag>

<PresentationFlag>false</PresentationFlag>

<ShowLabel>true</ShowLabel>

<AutoUpdate>true</AutoUpdate>

<RecurrenceCode><![CDATA[365]]></RecurrenceCode>

<FreezeXAxis>false</FreezeXAxis>

<HorizontalAxisExpression><![CDATA[getYear()]]></HorizontalAxisExpression>

<VerticalAxisExpression><![CDATA[persons.nQuint3()]]></VerticalAxisExpression>

<SamplesToKeep>100</SamplesToKeep>

</DataSet>

<DataSet>

<Id>1411575508139</Id>

<Name><![CDATA[dsPoor]]></Name>

<X>-1510</X><Y>70</Y>

<Label><X>15</X><Y>0</Y></Label>

<PublicFlag>false</PublicFlag>

<PresentationFlag>false</PresentationFlag>

<ShowLabel>true</ShowLabel>

<AutoUpdate>true</AutoUpdate>

<RecurrenceCode><![CDATA[365]]></RecurrenceCode>

<FreezeXAxis>false</FreezeXAxis>

<HorizontalAxisExpression><![CDATA[getYear()]]></HorizontalAxisExpression>

<VerticalAxisExpression><![CDATA[persons.nQuint2()]]></VerticalAxisExpression>

<SamplesToKeep>100</SamplesToKeep>

</DataSet>

<DataSet>

<Id>1411576353877</Id>

<Name><![CDATA[dsCancDeathRich]]></Name>

<X>-1190</X><Y>320</Y>

<Label><X>15</X><Y>0</Y></Label>

<PublicFlag>false</PublicFlag>

<PresentationFlag>false</PresentationFlag>

<ShowLabel>true</ShowLabel>

<AutoUpdate>true</AutoUpdate>

<RecurrenceCode><![CDATA[365]]></RecurrenceCode>

<FreezeXAxis>false</FreezeXAxis>

<HorizontalAxisExpression><![CDATA[getYear()]]></HorizontalAxisExpression>

<VerticalAxisExpression><![CDATA[cancerDeathRich]]></VerticalAxisExpression>

<SamplesToKeep>100</SamplesToKeep>

</DataSet>

<DataSet>

<Id>1411576353879</Id>

<Name><![CDATA[dsCancDeathMiddle]]></Name>

<X>-1190</X><Y>300</Y>

<Label><X>15</X><Y>0</Y></Label>

<PublicFlag>false</PublicFlag>

<PresentationFlag>false</PresentationFlag>

<ShowLabel>true</ShowLabel>

<AutoUpdate>true</AutoUpdate>

<RecurrenceCode><![CDATA[365]]></RecurrenceCode>

<FreezeXAxis>false</FreezeXAxis>

<HorizontalAxisExpression><![CDATA[getYear()]]></HorizontalAxisExpression>

<VerticalAxisExpression><![CDATA[cancerDeathMiddle]]></VerticalAxisExpression>

<SamplesToKeep>100</SamplesToKeep>

</DataSet>

<DataSet>

<Id>1411576353881</Id>

<Name><![CDATA[dsCancDeathPoor]]></Name>

<X>-1190</X><Y>280</Y>

<Label><X>15</X><Y>0</Y></Label>

<PublicFlag>false</PublicFlag>

<PresentationFlag>false</PresentationFlag>

<ShowLabel>true</ShowLabel>

<AutoUpdate>true</AutoUpdate>

<RecurrenceCode><![CDATA[365]]></RecurrenceCode>

<FreezeXAxis>false</FreezeXAxis>

<HorizontalAxisExpression><![CDATA[getYear()]]></HorizontalAxisExpression>

<VerticalAxisExpression><![CDATA[cancerDeathPoor]]></VerticalAxisExpression>

<SamplesToKeep>100</SamplesToKeep>

</DataSet>

<DataSet>

<Id>1411576363909</Id>

<Name><![CDATA[dsCancDeathPoorest]]></Name>

<X>-1190</X><Y>260</Y>

<Label><X>15</X><Y>0</Y></Label>

<PublicFlag>false</PublicFlag>

<PresentationFlag>false</PresentationFlag>

<ShowLabel>true</ShowLabel>

<AutoUpdate>true</AutoUpdate>

<RecurrenceCode><![CDATA[365]]></RecurrenceCode>

<FreezeXAxis>false</FreezeXAxis>

<HorizontalAxisExpression><![CDATA[getYear()]]></HorizontalAxisExpression>

<VerticalAxisExpression><![CDATA[cancerDeathPoorest]]></VerticalAxisExpression>

<SamplesToKeep>100</SamplesToKeep>

</DataSet>

<DataSet>

<Id>1411576363911</Id>

<Name><![CDATA[dsCancDeathRichest]]></Name>

<X>-1190</X><Y>340</Y>

<Label><X>15</X><Y>0</Y></Label>

<PublicFlag>false</PublicFlag>

<PresentationFlag>false</PresentationFlag>

<ShowLabel>true</ShowLabel>

<AutoUpdate>true</AutoUpdate>

<RecurrenceCode><![CDATA[365]]></RecurrenceCode>

<FreezeXAxis>false</FreezeXAxis>

<HorizontalAxisExpression><![CDATA[getYear()]]></HorizontalAxisExpression>

<VerticalAxisExpression><![CDATA[cancerDeathRichest]]></VerticalAxisExpression>

<SamplesToKeep>100</SamplesToKeep>

</DataSet>

<DataSet>

<Id>1411582446096</Id>

<Name><![CDATA[dsMadePoorMiddle]]></Name>

<X>-1330</X><Y>300</Y>

<Label><X>15</X><Y>0</Y></Label>

<PublicFlag>false</PublicFlag>

<PresentationFlag>false</PresentationFlag>

<ShowLabel>true</ShowLabel>

<AutoUpdate>true</AutoUpdate>

<RecurrenceCode><![CDATA[365]]></RecurrenceCode>

<FreezeXAxis>false</FreezeXAxis>

<HorizontalAxisExpression><![CDATA[getYear()]]></HorizontalAxisExpression>

<VerticalAxisExpression><![CDATA[madePoorMiddle]]></VerticalAxisExpression>

<SamplesToKeep>100</SamplesToKeep>

</DataSet>

<DataSet>

<Id>1411582446098</Id>

<Name><![CDATA[dsMadePoorPoor]]></Name>

<X>-1330</X><Y>280</Y>

<Label><X>15</X><Y>0</Y></Label>

<PublicFlag>false</PublicFlag>

<PresentationFlag>false</PresentationFlag>

<ShowLabel>true</ShowLabel>

<AutoUpdate>true</AutoUpdate>

<RecurrenceCode><![CDATA[365]]></RecurrenceCode>

<FreezeXAxis>false</FreezeXAxis>

<HorizontalAxisExpression><![CDATA[getYear()]]></HorizontalAxisExpression>

<VerticalAxisExpression><![CDATA[madePoorPoor]]></VerticalAxisExpression>

<SamplesToKeep>100</SamplesToKeep>

</DataSet>

<DataSet>

<Id>1411582446100</Id>

<Name><![CDATA[dsMadePoorPoorest]]></Name>

<X>-1330</X><Y>260</Y>

<Label><X>15</X><Y>0</Y></Label>

<PublicFlag>false</PublicFlag>

<PresentationFlag>false</PresentationFlag>

<ShowLabel>true</ShowLabel>

<AutoUpdate>true</AutoUpdate>

<RecurrenceCode><![CDATA[365]]></RecurrenceCode>

<FreezeXAxis>false</FreezeXAxis>

<HorizontalAxisExpression><![CDATA[getYear()]]></HorizontalAxisExpression>

<VerticalAxisExpression><![CDATA[madePoorPoorest]]></VerticalAxisExpression>

<SamplesToKeep>100</SamplesToKeep>

</DataSet>

<DataSet>

<Id>1411582446102</Id>

<Name><![CDATA[dsMadePoorRichest]]></Name>

<X>-1330</X><Y>340</Y>

<Label><X>15</X><Y>0</Y></Label>

<PublicFlag>false</PublicFlag>

<PresentationFlag>false</PresentationFlag>

<ShowLabel>true</ShowLabel>

<AutoUpdate>true</AutoUpdate>

<RecurrenceCode><![CDATA[365]]></RecurrenceCode>

<FreezeXAxis>false</FreezeXAxis>

<HorizontalAxisExpression><![CDATA[getYear()]]></HorizontalAxisExpression>

<VerticalAxisExpression><![CDATA[madePoorRichest]]></VerticalAxisExpression>

<SamplesToKeep>100</SamplesToKeep>

</DataSet>

<DataSet>

<Id>1411926255737</Id>

<Name><![CDATA[dsCatExpRich]]></Name>

<X>-1650</X><Y>530</Y>

<Label><X>15</X><Y>0</Y></Label>

<PublicFlag>false</PublicFlag>

<PresentationFlag>false</PresentationFlag>

<ShowLabel>true</ShowLabel>

<AutoUpdate>true</AutoUpdate>

<RecurrenceCode><![CDATA[365]]></RecurrenceCode>

<FreezeXAxis>false</FreezeXAxis>

<HorizontalAxisExpression><![CDATA[getYear()]]></HorizontalAxisExpression>

<VerticalAxisExpression><![CDATA[catExpRich]]></VerticalAxisExpression>

<SamplesToKeep>100</SamplesToKeep>

</DataSet>

<DataSet>

<Id>1411926255739</Id>

<Name><![CDATA[dsMadePoorNewRich]]></Name>

<X>-1510</X><Y>530</Y>

<Label><X>15</X><Y>0</Y></Label>

<PublicFlag>false</PublicFlag>

<PresentationFlag>false</PresentationFlag>

<ShowLabel>true</ShowLabel>

<AutoUpdate>true</AutoUpdate>

<RecurrenceCode><![CDATA[365]]></RecurrenceCode>

<FreezeXAxis>false</FreezeXAxis>

<HorizontalAxisExpression><![CDATA[getYear()]]></HorizontalAxisExpression>

<VerticalAxisExpression><![CDATA[madePoorNewRich]]></VerticalAxisExpression>

<SamplesToKeep>100</SamplesToKeep>

</DataSet>

<DataSet>

<Id>1411926255741</Id>

<Name><![CDATA[dsMadePoorNewMiddle]]></Name>

<X>-1510</X><Y>510</Y>

<Label><X>15</X><Y>0</Y></Label>

<PublicFlag>false</PublicFlag>

<PresentationFlag>false</PresentationFlag>

<ShowLabel>true</ShowLabel>

<AutoUpdate>true</AutoUpdate>

<RecurrenceCode><![CDATA[365]]></RecurrenceCode>

<FreezeXAxis>false</FreezeXAxis>

<HorizontalAxisExpression><![CDATA[getYear()]]></HorizontalAxisExpression>

<VerticalAxisExpression><![CDATA[madePoorNewMiddle]]></VerticalAxisExpression>

<SamplesToKeep>100</SamplesToKeep>

</DataSet>

<DataSet>

<Id>1411926255743</Id>

<Name><![CDATA[dsMadePoorNewPoor]]></Name>

<X>-1510</X><Y>490</Y>

<Label><X>15</X><Y>0</Y></Label>

<PublicFlag>false</PublicFlag>

<PresentationFlag>false</PresentationFlag>

<ShowLabel>true</ShowLabel>

<AutoUpdate>true</AutoUpdate>

<RecurrenceCode><![CDATA[365]]></RecurrenceCode>

<FreezeXAxis>false</FreezeXAxis>

<HorizontalAxisExpression><![CDATA[getYear()]]></HorizontalAxisExpression>

<VerticalAxisExpression><![CDATA[madePoorNewPoor]]></VerticalAxisExpression>

<SamplesToKeep>100</SamplesToKeep>

</DataSet>

<DataSet>

<Id>1411926255745</Id>

<Name><![CDATA[dsMadePoorNewPoorest]]></Name>

<X>-1510</X><Y>470</Y>

<Label><X>15</X><Y>0</Y></Label>

<PublicFlag>false</PublicFlag>

<PresentationFlag>false</PresentationFlag>

<ShowLabel>true</ShowLabel>

<AutoUpdate>true</AutoUpdate>

<RecurrenceCode><![CDATA[365]]></RecurrenceCode>

<FreezeXAxis>false</FreezeXAxis>

<HorizontalAxisExpression><![CDATA[getYear()]]></HorizontalAxisExpression>

<VerticalAxisExpression><![CDATA[madePoorNewPoorest]]></VerticalAxisExpression>

<SamplesToKeep>100</SamplesToKeep>

</DataSet>

<DataSet>

<Id>1411926255747</Id>

<Name><![CDATA[dsMadePoorNewRichest]]></Name>

<X>-1510</X><Y>550</Y>

<Label><X>15</X><Y>0</Y></Label>

<PublicFlag>false</PublicFlag>

<PresentationFlag>false</PresentationFlag>

<ShowLabel>true</ShowLabel>

<AutoUpdate>true</AutoUpdate>

<RecurrenceCode><![CDATA[365]]></RecurrenceCode>

<FreezeXAxis>false</FreezeXAxis>

<HorizontalAxisExpression><![CDATA[getYear()]]></HorizontalAxisExpression>

<VerticalAxisExpression><![CDATA[madePoorNewRichest]]></VerticalAxisExpression>

<SamplesToKeep>100</SamplesToKeep>

</DataSet>

<DataSet>

<Id>1411926255749</Id>

<Name><![CDATA[dsCatExpMiddle]]></Name>

<X>-1650</X><Y>510</Y>

<Label><X>15</X><Y>0</Y></Label>

<PublicFlag>false</PublicFlag>

<PresentationFlag>false</PresentationFlag>

<ShowLabel>true</ShowLabel>

<AutoUpdate>true</AutoUpdate>

<RecurrenceCode><![CDATA[365]]></RecurrenceCode>

<FreezeXAxis>false</FreezeXAxis>

<HorizontalAxisExpression><![CDATA[getYear()]]></HorizontalAxisExpression>

<VerticalAxisExpression><![CDATA[catExpMiddle]]></VerticalAxisExpression>

<SamplesToKeep>100</SamplesToKeep>

</DataSet>

<DataSet>

<Id>1411926255751</Id>

<Name><![CDATA[dsCatExpPoor]]></Name>

<X>-1650</X><Y>490</Y>

<Label><X>15</X><Y>0</Y></Label>

<PublicFlag>false</PublicFlag>

<PresentationFlag>false</PresentationFlag>

<ShowLabel>true</ShowLabel>

<AutoUpdate>true</AutoUpdate>

<RecurrenceCode><![CDATA[365]]></RecurrenceCode>

<FreezeXAxis>false</FreezeXAxis>

<HorizontalAxisExpression><![CDATA[getYear()]]></HorizontalAxisExpression>

<VerticalAxisExpression><![CDATA[catExpPoor]]></VerticalAxisExpression>

<SamplesToKeep>100</SamplesToKeep>

</DataSet>

<DataSet>

<Id>1411926255753</Id>

<Name><![CDATA[dsCatExpPoorest]]></Name>

<X>-1650</X><Y>470</Y>

<Label><X>15</X><Y>0</Y></Label>

<PublicFlag>false</PublicFlag>

<PresentationFlag>false</PresentationFlag>

<ShowLabel>true</ShowLabel>

<AutoUpdate>true</AutoUpdate>

<RecurrenceCode><![CDATA[365]]></RecurrenceCode>

<FreezeXAxis>false</FreezeXAxis>

<HorizontalAxisExpression><![CDATA[getYear()]]></HorizontalAxisExpression>

<VerticalAxisExpression><![CDATA[catExpPoorest]]></VerticalAxisExpression>

<SamplesToKeep>100</SamplesToKeep>

</DataSet>

<DataSet>

<Id>1411926255755</Id>

<Name><![CDATA[dsCatExpRichest]]></Name>

<X>-1650</X><Y>550</Y>

<Label><X>15</X><Y>0</Y></Label>

<PublicFlag>false</PublicFlag>

<PresentationFlag>false</PresentationFlag>

<ShowLabel>true</ShowLabel>

<AutoUpdate>true</AutoUpdate>

<RecurrenceCode><![CDATA[365]]></RecurrenceCode>

<FreezeXAxis>false</FreezeXAxis>

<HorizontalAxisExpression><![CDATA[getYear()]]></HorizontalAxisExpression>

<VerticalAxisExpression><![CDATA[catExpRichest]]></VerticalAxisExpression>

<SamplesToKeep>100</SamplesToKeep>

</DataSet>

<DataSet>

<Id>1412104712492</Id>

<Name><![CDATA[dsCancIncRich]]></Name>

<X>-1650</X><Y>320</Y>

<Label><X>15</X><Y>0</Y></Label>

<PublicFlag>false</PublicFlag>

<PresentationFlag>false</PresentationFlag>

<ShowLabel>true</ShowLabel>

<AutoUpdate>true</AutoUpdate>

<RecurrenceCode><![CDATA[365]]></RecurrenceCode>

<FreezeXAxis>false</FreezeXAxis>

<HorizontalAxisExpression><![CDATA[getYear()]]></HorizontalAxisExpression>

<VerticalAxisExpression><![CDATA[cancIncRich]]></VerticalAxisExpression>

<SamplesToKeep>100</SamplesToKeep>

</DataSet>

<DataSet>

<Id>1412104712494</Id>

<Name><![CDATA[dsCancTxRich]]></Name>

<X>-1510</X><Y>320</Y>

<Label><X>15</X><Y>0</Y></Label>

<PublicFlag>false</PublicFlag>

<PresentationFlag>false</PresentationFlag>

<ShowLabel>true</ShowLabel>

<AutoUpdate>true</AutoUpdate>

<RecurrenceCode><![CDATA[365]]></RecurrenceCode>

<FreezeXAxis>false</FreezeXAxis>

<HorizontalAxisExpression><![CDATA[getYear()]]></HorizontalAxisExpression>

<VerticalAxisExpression><![CDATA[cancTxRich]]></VerticalAxisExpression>

<SamplesToKeep>100</SamplesToKeep>

</DataSet>

<DataSet>

<Id>1412104712496</Id>

<Name><![CDATA[dsCancTxMiddle]]></Name>

<X>-1510</X><Y>300</Y>

<Label><X>15</X><Y>0</Y></Label>

<PublicFlag>false</PublicFlag>

<PresentationFlag>false</PresentationFlag>

<ShowLabel>true</ShowLabel>

<AutoUpdate>true</AutoUpdate>

<RecurrenceCode><![CDATA[365]]></RecurrenceCode>

<FreezeXAxis>false</FreezeXAxis>

<HorizontalAxisExpression><![CDATA[getYear()]]></HorizontalAxisExpression>

<VerticalAxisExpression><![CDATA[cancTxMiddle]]></VerticalAxisExpression>

<SamplesToKeep>100</SamplesToKeep>

</DataSet>

<DataSet>

<Id>1412104712498</Id>

<Name><![CDATA[dsCancTxPoor]]></Name>

<X>-1510</X><Y>280</Y>

<Label><X>15</X><Y>0</Y></Label>

<PublicFlag>false</PublicFlag>

<PresentationFlag>false</PresentationFlag>

<ShowLabel>true</ShowLabel>

<AutoUpdate>true</AutoUpdate>

<RecurrenceCode><![CDATA[365]]></RecurrenceCode>

<FreezeXAxis>false</FreezeXAxis>

<HorizontalAxisExpression><![CDATA[getYear()]]></HorizontalAxisExpression>

<VerticalAxisExpression><![CDATA[cancTxPoor]]></VerticalAxisExpression>

<SamplesToKeep>100</SamplesToKeep>

</DataSet>

<DataSet>

<Id>1412104712500</Id>

<Name><![CDATA[dsCancTxPoorest]]></Name>

<X>-1510</X><Y>260</Y>

<Label><X>15</X><Y>0</Y></Label>

<PublicFlag>false</PublicFlag>

<PresentationFlag>false</PresentationFlag>

<ShowLabel>true</ShowLabel>

<AutoUpdate>true</AutoUpdate>

<RecurrenceCode><![CDATA[365]]></RecurrenceCode>

<FreezeXAxis>false</FreezeXAxis>

<HorizontalAxisExpression><![CDATA[getYear()]]></HorizontalAxisExpression>

<VerticalAxisExpression><![CDATA[cancTxPoorest]]></VerticalAxisExpression>

<SamplesToKeep>100</SamplesToKeep>

</DataSet>

<DataSet>

<Id>1412104712502</Id>

<Name><![CDATA[dsCancTxRichest]]></Name>

<X>-1510</X><Y>340</Y>

<Label><X>15</X><Y>0</Y></Label>

<PublicFlag>false</PublicFlag>

<PresentationFlag>false</PresentationFlag>

<ShowLabel>true</ShowLabel>

<AutoUpdate>true</AutoUpdate>

<RecurrenceCode><![CDATA[365]]></RecurrenceCode>

<FreezeXAxis>false</FreezeXAxis>

<HorizontalAxisExpression><![CDATA[getYear()]]></HorizontalAxisExpression>

<VerticalAxisExpression><![CDATA[cancTxRichest]]></VerticalAxisExpression>

<SamplesToKeep>100</SamplesToKeep>

</DataSet>

<DataSet>

<Id>1412104712504</Id>

<Name><![CDATA[dsCancIncMiddle]]></Name>

<X>-1650</X><Y>300</Y>

<Label><X>15</X><Y>0</Y></Label>

<PublicFlag>false</PublicFlag>

<PresentationFlag>false</PresentationFlag>

<ShowLabel>true</ShowLabel>

<AutoUpdate>true</AutoUpdate>

<RecurrenceCode><![CDATA[365]]></RecurrenceCode>

<FreezeXAxis>false</FreezeXAxis>

<HorizontalAxisExpression><![CDATA[getYear()]]></HorizontalAxisExpression>

<VerticalAxisExpression><![CDATA[cancIncMiddle]]></VerticalAxisExpression>

<SamplesToKeep>100</SamplesToKeep>

</DataSet>

<DataSet>

<Id>1412104712506</Id>

<Name><![CDATA[dsCancIncPoor]]></Name>

<X>-1650</X><Y>280</Y>

<Label><X>15</X><Y>0</Y></Label>

<PublicFlag>false</PublicFlag>

<PresentationFlag>false</PresentationFlag>

<ShowLabel>true</ShowLabel>

<AutoUpdate>true</AutoUpdate>

<RecurrenceCode><![CDATA[365]]></RecurrenceCode>

<FreezeXAxis>false</FreezeXAxis>

<HorizontalAxisExpression><![CDATA[getYear()]]></HorizontalAxisExpression>

<VerticalAxisExpression><![CDATA[cancIncPoor]]></VerticalAxisExpression>

<SamplesToKeep>100</SamplesToKeep>

</DataSet>

<DataSet>

<Id>1412104712508</Id>

<Name><![CDATA[dsCancIncPoorest]]></Name>

<X>-1650</X><Y>260</Y>

<Label><X>15</X><Y>0</Y></Label>

<PublicFlag>false</PublicFlag>

<PresentationFlag>false</PresentationFlag>

<ShowLabel>true</ShowLabel>

<AutoUpdate>true</AutoUpdate>

<RecurrenceCode><![CDATA[365]]></RecurrenceCode>

<FreezeXAxis>false</FreezeXAxis>

<HorizontalAxisExpression><![CDATA[getYear()]]></HorizontalAxisExpression>

<VerticalAxisExpression><![CDATA[cancIncPoorest]]></VerticalAxisExpression>

<SamplesToKeep>100</SamplesToKeep>

</DataSet>

<DataSet>

<Id>1412104712510</Id>

<Name><![CDATA[dsCancIncRichest]]></Name>

<X>-1650</X><Y>340</Y>

<Label><X>15</X><Y>0</Y></Label>

<PublicFlag>false</PublicFlag>

<PresentationFlag>false</PresentationFlag>

<ShowLabel>true</ShowLabel>

<AutoUpdate>true</AutoUpdate>

<RecurrenceCode><![CDATA[365]]></RecurrenceCode>

<FreezeXAxis>false</FreezeXAxis>

<HorizontalAxisExpression><![CDATA[getYear()]]></HorizontalAxisExpression>

<VerticalAxisExpression><![CDATA[cancIncRichest]]></VerticalAxisExpression>

<SamplesToKeep>100</SamplesToKeep>

</DataSet>

<DataSet>

<Id>1412806026639</Id>

<Name><![CDATA[dsPersCost]]></Name>

<X>-1260</X><Y>50</Y>

<Label><X>15</X><Y>0</Y></Label>

<PublicFlag>false</PublicFlag>

<PresentationFlag>true</PresentationFlag>

<ShowLabel>true</ShowLabel>

<AutoUpdate>true</AutoUpdate>

<RecurrenceCode><![CDATA[365]]></RecurrenceCode>

<FreezeXAxis>false</FreezeXAxis>

<HorizontalAxisExpression><![CDATA[getYear()]]></HorizontalAxisExpression>

<VerticalAxisExpression><![CDATA[persCost]]></VerticalAxisExpression>

<SamplesToKeep>100</SamplesToKeep>

</DataSet>

<DataSet>

<Id>1425335019685</Id>

<Name><![CDATA[dsNGOSurgeries]]></Name>

<X>-1330</X><Y>400</Y>

<Label><X>15</X><Y>0</Y></Label>

<PublicFlag>false</PublicFlag>

<PresentationFlag>false</PresentationFlag>

<ShowLabel>true</ShowLabel>

<AutoUpdate>true</AutoUpdate>

<RecurrenceCode><![CDATA[365]]></RecurrenceCode>

<FreezeXAxis>false</FreezeXAxis>

<HorizontalAxisExpression><![CDATA[getYear()]]></HorizontalAxisExpression>

<VerticalAxisExpression><![CDATA[ngoSurgeries]]></VerticalAxisExpression>

<SamplesToKeep>100</SamplesToKeep>

</DataSet>

</AnalysisData>

<Connectivity>

<ExcelFile>

<Id>1390859945381</Id>

<Name><![CDATA[GPSandPopulation]]></Name>

<X>-500</X><Y>240</Y>

<Label><X>10</X><Y>0</Y></Label>

<PublicFlag>false</PublicFlag>

<PresentationFlag>false</PresentationFlag>

<ShowLabel>true</ShowLabel>

<FileName><![CDATA[/Users/Mark/Dropbox/@PhD/Dissertation/ABM/Data/GPS and population.xlsx]]></FileName>

<SaveInSnapshot>false</SaveInSnapshot>

<LoadOnStartup>true</LoadOnStartup>

<SaveOnDispose>false</SaveOnDispose>

</ExcelFile>

<ExcelFile>

<Id>1391289744769</Id>

<Name><![CDATA[ASRmortality]]></Name>

<X>-360</X><Y>409</Y>

<Label><X>10</X><Y>0</Y></Label>

<PublicFlag>false</PublicFlag>

<PresentationFlag>false</PresentationFlag>

<ShowLabel>true</ShowLabel>

<FileName><![CDATA[/Users/Mark/Dropbox/@PhD/Dissertation/ABM/Data/UGA_Daily_Life_Table.xlsx]]></FileName>

<SaveInSnapshot>false</SaveInSnapshot>

<LoadOnStartup>true</LoadOnStartup>

<SaveOnDispose>true</SaveOnDispose>

</ExcelFile>

<ExcelFile>

<Id>1391297764648</Id>

<Name><![CDATA[incMort]]></Name>

<Description><![CDATA[Incidence and mortality data]]></Description>

<X>-510</X><Y>410</Y>

<Label><X>10</X><Y>0</Y></Label>

<PublicFlag>false</PublicFlag>

<PresentationFlag>false</PresentationFlag>

<ShowLabel>true</ShowLabel>

<FileName><![CDATA[/Users/Mark/Dropbox/@PhD/Dissertation/ABM/Data/Cancer Incidence and Mortality.xlsx]]></FileName>

<SaveInSnapshot>false</SaveInSnapshot>

<LoadOnStartup>true</LoadOnStartup>

<SaveOnDispose>true</SaveOnDispose>

</ExcelFile>

<ExcelFile>

<Id>1392332991133</Id>

<Name><![CDATA[cancerMort]]></Name>

<X>-510</X><Y>430</Y>

<Label><X>10</X><Y>0</Y></Label>

<PublicFlag>false</PublicFlag>

<PresentationFlag>false</PresentationFlag>

<ShowLabel>true</ShowLabel>

<FileName><![CDATA[/Users/Mark/Dropbox/@PhD/Dissertation/ABM/Data/UGA All GBD cancer mortality.xlsx]]></FileName>

<SaveInSnapshot>false</SaveInSnapshot>

<LoadOnStartup>true</LoadOnStartup>

<SaveOnDispose>true</SaveOnDispose>

</ExcelFile>

<ExcelFile>

<Id>1394324398323</Id>

<Name><![CDATA[betas]]></Name>

<Description><![CDATA[Includes the coefficients for the NMLM]]></Description>

<X>-700</X><Y>550</Y>

<Label><X>10</X><Y>0</Y></Label>

<PublicFlag>false</PublicFlag>

<PresentationFlag>false</PresentationFlag>

<ShowLabel>true</ShowLabel>

<FileName><![CDATA[/Users/Mark/Dropbox/@PhD/Dissertation/ABM/Data/Provider choice_for model.xlsx]]></FileName>

<SaveInSnapshot>false</SaveInSnapshot>

<LoadOnStartup>true</LoadOnStartup>

<SaveOnDispose>true</SaveOnDispose>

</ExcelFile>

<ExcelFile>

<Id>1410459065434</Id>

<Name><![CDATA[hospQuality]]></Name>

<X>-910</X><Y>50</Y>

<Label><X>10</X><Y>0</Y></Label>

<PublicFlag>false</PublicFlag>

<PresentationFlag>false</PresentationFlag>

<ShowLabel>true</ShowLabel>

<FileName><![CDATA[/Users/Mark/Dropbox/@PhD/Dissertation/ABM/Data/Serufusa data/All Hospital Data.xlsx]]></FileName>

<SaveInSnapshot>false</SaveInSnapshot>

<LoadOnStartup>true</LoadOnStartup>

<SaveOnDispose>true</SaveOnDispose>

</ExcelFile>

<ExcelFile>

<Id>1416773729907</Id>

<Name><![CDATA[excelPersCost]]></Name>

<X>-1260</X><Y>80</Y>

<Label><X>10</X><Y>0</Y></Label>

<PublicFlag>false</PublicFlag>

<PresentationFlag>false</PresentationFlag>

<ShowLabel>true</ShowLabel>

<FileName><![CDATA[/Users/Mark/Dropbox/@PhD/Dissertation/ABM/Results/Model output/Personal Cost.xlsx]]></FileName>

<SaveInSnapshot>false</SaveInSnapshot>

<LoadOnStartup>true</LoadOnStartup>

<SaveOnDispose>true</SaveOnDispose>

</ExcelFile>

<ExcelFile>

<Id>1417023380834</Id>

<Name><![CDATA[excelIncomeCalc]]></Name>

<X>-700</X><Y>480</Y>

<Label><X>10</X><Y>0</Y></Label>

<PublicFlag>false</PublicFlag>

<PresentationFlag>true</PresentationFlag>

<ShowLabel>true</ShowLabel>

<FileName><![CDATA[/Users/Mark/Dropbox/@PhD/Dissertation/ABM/Data/Income calculation.xlsx]]></FileName>

<SaveInSnapshot>false</SaveInSnapshot>

<LoadOnStartup>true</LoadOnStartup>

<SaveOnDispose>true</SaveOnDispose>

</ExcelFile>

</Connectivity>

<AgentLinks>

<AgentLink>

<Id>1392508625677</Id>

<Name><![CDATA[connections]]></Name>

<X>50</X><Y>-50</Y>

<Label><X>15</X><Y>0</Y></Label>

<PublicFlag>false</PublicFlag>

<PresentationFlag>true</PresentationFlag>

<ShowLabel>true</ShowLabel>

<HandleReceiveInConnections>false</HandleReceiveInConnections>

<AgentLinkType>COLLECTION_OF_LINKS</AgentLinkType>

<AgentLinkBidirectional>true</AgentLinkBidirectional>

<MessageType><![CDATA[Object]]></MessageType>

<LineStyle>SOLID</LineStyle>

<LineWidth>1</LineWidth>

<LineColor>-16777216</LineColor>

<LineZOrder>UNDER_AGENTS</LineZOrder>

<LineArrow>NONE</LineArrow>

<LineArrowPosition>END</LineArrowPosition>

</AgentLink>

</AgentLinks>

<EmbeddedObjects>

<EmbeddedObject>

<Id>1390443417224</Id>

<Name><![CDATA[hospitals]]></Name>

<X>-300</X><Y>150</Y>

<Label><X>20</X><Y>0</Y></Label>

<PublicFlag>false</PublicFlag>

<PresentationFlag>false</PresentationFlag>

<ShowLabel>true</ShowLabel>

<PresentationId>1390443420968</PresentationId>

<ActiveObjectClass>

<PackageName><![CDATA[_14_01_21_trial_uganda_model]]></PackageName>

<ClassName><![CDATA[Hospital]]></ClassName>

</ActiveObjectClass>

<GenericParametersSubstitute><![CDATA[]]></GenericParametersSubstitute>

<Parameters>

<Parameter>

<Name><![CDATA[name]]></Name>

<Value><![CDATA[]]></Value>

</Parameter>

<Parameter>

<Name><![CDATA[priv]]></Name>

<Value><![CDATA[]]></Value>

</Parameter>

<Parameter>

<Name><![CDATA[latitude]]></Name>

<Value><![CDATA[]]></Value>

</Parameter>

<Parameter>

<Name><![CDATA[longitude]]></Name>

<Value><![CDATA[]]></Value>

</Parameter>

<Parameter>

<Name><![CDATA[quality]]></Name>

<Value><![CDATA[]]></Value>

</Parameter>

<Parameter>

<Name><![CDATA[NGO]]></Name>

<Value><![CDATA[]]></Value>

</Parameter>

<Parameter>

<Name><![CDATA[cost]]></Name>

<Value><![CDATA[]]></Value>

</Parameter>

<Parameter>

<Name><![CDATA[nonmed]]></Name>

<Value><![CDATA[]]></Value>

</Parameter>

<Parameter>

<Name><![CDATA[amIMercyShips]]></Name>

<Value><![CDATA[]]></Value>

</Parameter>

<Parameter>

<Name><![CDATA[amITwoWeek]]></Name>

<Value><![CDATA[]]></Value>

</Parameter>

</Parameters>

<ReplicationFlag>true</ReplicationFlag>

<Replication><![CDATA[0]]></Replication>

<EnvironmentCode><![CDATA[this]]></EnvironmentCode>

<CollectionType>ARRAY_LIST_BASED</CollectionType>

<InEnvironment>true</InEnvironment>

<InitialLocationType>AT_ANIMATION_POSITION</InitialLocationType>

<XCode><![CDATA[0]]></XCode>

<YCode><![CDATA[0]]></YCode>

<ZCode><![CDATA[0]]></ZCode>

<ColumnCode><![CDATA[0]]></ColumnCode>

<RowCode><![CDATA[0]]></RowCode>

<LatitudeCode><![CDATA[0]]></LatitudeCode>

<LongitudeCode><![CDATA[0]]></LongitudeCode>

</EmbeddedObject>

<EmbeddedObject>

<Id>0</Id>

<Name><![CDATA[regionals]]></Name>

<X>-300</X><Y>170</Y>

<Label><X>20</X><Y>0</Y></Label>

<PublicFlag>false</PublicFlag>

<PresentationFlag>false</PresentationFlag>

<ShowLabel>true</ShowLabel>

<PresentationId>1390766198906</PresentationId>

<ActiveObjectClass>

<PackageName><![CDATA[_14_01_21_trial_uganda_model]]></PackageName>

<ClassName><![CDATA[Regional]]></ClassName>

</ActiveObjectClass>

<GenericParametersSubstitute><![CDATA[]]></GenericParametersSubstitute>

<Parameters>

<Parameter>

<Name><![CDATA[name]]></Name>

<Value><![CDATA[]]></Value>

</Parameter>

<Parameter>

<Name><![CDATA[priv]]></Name>

<Value><![CDATA[]]></Value>

</Parameter>

<Parameter>

<Name><![CDATA[latitude]]></Name>

<Value><![CDATA[]]></Value>

</Parameter>

<Parameter>

<Name><![CDATA[longitude]]></Name>

<Value><![CDATA[]]></Value>

</Parameter>

<Parameter>

<Name><![CDATA[quality]]></Name>

<Value><![CDATA[]]></Value>

</Parameter>

<Parameter>

<Name><![CDATA[NGO]]></Name>

<Value><![CDATA[]]></Value>

</Parameter>

<Parameter>

<Name><![CDATA[cost]]></Name>

<Value><![CDATA[]]></Value>

</Parameter>

<Parameter>

<Name><![CDATA[nonmed]]></Name>

<Value><![CDATA[]]></Value>

</Parameter>

</Parameters>

<ReplicationFlag>true</ReplicationFlag>

<Replication><![CDATA[0]]></Replication>

<EnvironmentCode><![CDATA[this]]></EnvironmentCode>

<CollectionType>ARRAY_LIST_BASED</CollectionType>

<InEnvironment>true</InEnvironment>

<InitialLocationType>AT_ANIMATION_POSITION</InitialLocationType>

<XCode><![CDATA[0]]></XCode>

<YCode><![CDATA[0]]></YCode>

<ZCode><![CDATA[0]]></ZCode>

<ColumnCode><![CDATA[0]]></ColumnCode>

<RowCode><![CDATA[0]]></RowCode>

<LatitudeCode><![CDATA[0]]></LatitudeCode>

<LongitudeCode><![CDATA[0]]></LongitudeCode>

</EmbeddedObject>

<EmbeddedObject>

<Id>0</Id>

<Name><![CDATA[districts]]></Name>

<X>-300</X><Y>190</Y>

<Label><X>20</X><Y>0</Y></Label>

<PublicFlag>false</PublicFlag>

<PresentationFlag>false</PresentationFlag>

<ShowLabel>true</ShowLabel>

<PresentationId>1390768734138</PresentationId>

<ActiveObjectClass>

<PackageName><![CDATA[_14_01_21_trial_uganda_model]]></PackageName>

<ClassName><![CDATA[District]]></ClassName>

</ActiveObjectClass>

<GenericParametersSubstitute><![CDATA[]]></GenericParametersSubstitute>

<Parameters>

<Parameter>

<Name><![CDATA[name]]></Name>

<Value><![CDATA[]]></Value>

</Parameter>

<Parameter>

<Name><![CDATA[priv]]></Name>

<Value><![CDATA[]]></Value>

</Parameter>

<Parameter>

<Name><![CDATA[latitude]]></Name>

<Value><![CDATA[]]></Value>

</Parameter>

<Parameter>

<Name><![CDATA[longitude]]></Name>

<Value><![CDATA[]]></Value>

</Parameter>

<Parameter>

<Name><![CDATA[quality]]></Name>

<Value><![CDATA[]]></Value>

</Parameter>

<Parameter>

<Name><![CDATA[NGO]]></Name>

<Value><![CDATA[]]></Value>

</Parameter>

<Parameter>

<Name><![CDATA[cost]]></Name>

<Value><![CDATA[]]></Value>

</Parameter>

<Parameter>

<Name><![CDATA[nonmed]]></Name>

<Value><![CDATA[]]></Value>

</Parameter>

</Parameters>

<ReplicationFlag>true</ReplicationFlag>

<Replication><![CDATA[0]]></Replication>

<EnvironmentCode><![CDATA[this]]></EnvironmentCode>

<CollectionType>ARRAY_LIST_BASED</CollectionType>

<InEnvironment>true</InEnvironment>

<InitialLocationType>AT_ANIMATION_POSITION</InitialLocationType>

<XCode><![CDATA[0]]></XCode>

<YCode><![CDATA[0]]></YCode>

<ZCode><![CDATA[0]]></ZCode>

<ColumnCode><![CDATA[0]]></ColumnCode>

<RowCode><![CDATA[0]]></RowCode>

<LatitudeCode><![CDATA[0]]></LatitudeCode>

<LongitudeCode><![CDATA[0]]></LongitudeCode>

</EmbeddedObject>

<EmbeddedObject>

<Id>0</Id>

<Name><![CDATA[persons]]></Name>

<X>-300</X><Y>210</Y>

<Label><X>20</X><Y>0</Y></Label>

<PublicFlag>false</PublicFlag>

<PresentationFlag>false</PresentationFlag>

<ShowLabel>true</ShowLabel>

<PresentationId>1390777039089</PresentationId>

<ActiveObjectClass>

<PackageName><![CDATA[_14_01_21_trial_uganda_model]]></PackageName>

<ClassName><![CDATA[Person]]></ClassName>

</ActiveObjectClass>

<GenericParametersSubstitute><![CDATA[]]></GenericParametersSubstitute>

<Parameters>

<Parameter>

<Name><![CDATA[latitude]]></Name>

<Value><![CDATA[]]></Value>

</Parameter>

<Parameter>

<Name><![CDATA[longitude]]></Name>

<Value><![CDATA[]]></Value>

</Parameter>

<Parameter>

<Name><![CDATA[male]]></Name>

<Value><![CDATA[]]></Value>

</Parameter>

<Parameter>

<Name><![CDATA[startState]]></Name>

<Value><![CDATA[]]></Value>

</Parameter>

<Parameter>

<Name><![CDATA[urban]]></Name>

<Value><![CDATA[]]></Value>

</Parameter>

<Parameter>

<Name><![CDATA[quintile]]></Name>

<Value><![CDATA[]]></Value>

</Parameter>

<Parameter>

<Name><![CDATA[poor]]></Name>

<Value><![CDATA[]]></Value>

</Parameter>

<Parameter>

<Name><![CDATA[HOH]]></Name>

<Value><![CDATA[]]></Value>

</Parameter>

</Parameters>

<ReplicationFlag>true</ReplicationFlag>

<Replication><![CDATA[0]]></Replication>

<EnvironmentCode><![CDATA[this]]></EnvironmentCode>

<CollectionType>ARRAY_LIST_BASED</CollectionType>

<StatisticsItem>

<Name><![CDATA[nAlive]]></Name>

<Type>count</Type>

<Expression><![CDATA[]]></Expression>

<Condition><![CDATA[item.statechart.isStateActive(item.Alive)]]></Condition>

</StatisticsItem>

<StatisticsItem>

<Name><![CDATA[nQuint1]]></Name>

<Type>count</Type>

<Expression><![CDATA[]]></Expression>

<Condition><![CDATA[item.quintile == 1]]></Condition>

</StatisticsItem>

<StatisticsItem>

<Name><![CDATA[nQuint2]]></Name>

<Type>count</Type>

<Expression><![CDATA[]]></Expression>

<Condition><![CDATA[item.quintile == 2]]></Condition>

</StatisticsItem>

<StatisticsItem>

<Name><![CDATA[nQuint3]]></Name>

<Type>count</Type>

<Expression><![CDATA[]]></Expression>

<Condition><![CDATA[item.quintile == 3]]></Condition>

</StatisticsItem>

<StatisticsItem>

<Name><![CDATA[nQuint4]]></Name>

<Type>count</Type>

<Expression><![CDATA[]]></Expression>

<Condition><![CDATA[item.quintile == 4]]></Condition>

</StatisticsItem>

<StatisticsItem>

<Name><![CDATA[nQuint5]]></Name>

<Type>count</Type>

<Expression><![CDATA[]]></Expression>

<Condition><![CDATA[item.quintile == 5]]></Condition>

</StatisticsItem>

<InEnvironment>true</InEnvironment>

<InitialLocationType>AT_ANIMATION_POSITION</InitialLocationType>

<XCode><![CDATA[0]]></XCode>

<YCode><![CDATA[0]]></YCode>

<ZCode><![CDATA[0]]></ZCode>

<ColumnCode><![CDATA[0]]></ColumnCode>

<RowCode><![CDATA[0]]></RowCode>

<LatitudeCode><![CDATA[0]]></LatitudeCode>

<LongitudeCode><![CDATA[0]]></LongitudeCode>

</EmbeddedObject>

</EmbeddedObjects>

<Shapes>

<Rectangle>

<Id>1393271468753</Id>

<Name><![CDATA[rectangle9]]></Name>

<X>-320</X><Y>10</Y>

<Label><X>10</X><Y>10</Y></Label>

<PublicFlag>true</PublicFlag>

<PresentationFlag>false</PresentationFlag>

<ShowLabel>false</ShowLabel>

<DrawMode>SHAPE_DRAW_2D3D</DrawMode>

<AsObject>true</AsObject>

<EmbeddedIcon>false</EmbeddedIcon>

<Z>0</Z>

<ZHeight>10</ZHeight>

<LineWidth>1</LineWidth>

<LineColor>-16777216</LineColor>

<LineMaterial>null</LineMaterial>

<LineStyle>SOLID</LineStyle>

<Width>130</Width>

<Height>60</Height>

<Rotation>0.0</Rotation>

<FillColor>-1</FillColor>

<FillMaterial>null</FillMaterial>

</Rectangle>

<GISMap>

<Id>1390440691067</Id>

<Name><![CDATA[Uganda]]></Name>

<X>0</X><Y>0</Y>

<Label><X>0</X><Y>-10</Y></Label>

<PublicFlag>true</PublicFlag>

<PresentationFlag>true</PresentationFlag>

<ShowLabel>false</ShowLabel>

<DrawMode>SHAPE_DRAW_2D</DrawMode>

<OnClickCode><![CDATA[polAreaName = Uganda.getLayers()[1].findPoliticalArea(clickx, clicky).name;]]></OnClickCode>

<AsObject>true</AsObject>

<EmbeddedIcon>false</EmbeddedIcon>

<Width>1200</Width>

<Height>1070</Height>

<OnClickCode><![CDATA[polAreaName = Uganda.getLayers()[1].findPoliticalArea(clickx, clicky).name;]]></OnClickCode>

<GISShapes>

<GISShapeFile>

<ShapeFileName><![CDATA[UGA_adm1.shp]]></ShapeFileName>

<DbfFileName><![CDATA[UGA_adm1.dbf]]></DbfFileName>

<FillColor>-1553</FillColor>

<LineColor>-4106702</LineColor>

<NameColumnIndex>0</NameColumnIndex>

<Bounds><LonMin>29.57149900000013</LonMin><LatMin>-1.4818227099999604</LatMin><LonMax>35.02303503100007</LonMax><LatMax>4.2344660000000545</LatMax></Bounds>

</GISShapeFile>

<GISShapeFile>

<ShapeFileName><![CDATA[Ug_Rural-Poverty2005.shp]]></ShapeFileName>

<DbfFileName><![CDATA[Ug_Rural-Poverty2005.dbf]]></DbfFileName>

<FillColor/>

<LineColor/>

<NameColumnIndex>5</NameColumnIndex>

<Bounds><LonMin>29.57274236172238</LonMin><LatMin>-1.478793505876424</LatMin><LonMax>35.000308036061</LonMax><LatMax>4.234076631097764</LatMax></Bounds>

</GISShapeFile>

<GISShapeFile>

<ShapeFileName><![CDATA[Uganda_regions_2014.shp]]></ShapeFileName>

<DbfFileName><![CDATA[Uganda_regions_2014.dbf]]></DbfFileName>

<FillColor/>

<LineColor/>

<NameColumnIndex>0</NameColumnIndex>

<Bounds><LonMin>29.572546464000027</LonMin><LatMin>-1.4818227099999604</LatMin><LonMax>35.02303503100007</LonMax><LatMax>4.232322233000022</LatMax></Bounds>

</GISShapeFile>

</GISShapes>

<Latitude>1.38</Latitude>

<Longitude>32.3</Longitude>

<Scale>1945875</Scale>

<BorderColor>-16777216</BorderColor>

<FillColor>-5383962</FillColor>

</GISMap>

<EmbeddedObjectPresentation>

<Id>1390777039089</Id>

<Name><![CDATA[persons_presentation]]></Name>

<X>0</X><Y>0</Y>

<Label><X>10</X><Y>0</Y></Label>

<PublicFlag>true</PublicFlag>

<PresentationFlag>true</PresentationFlag>

<ShowLabel>false</ShowLabel>

<DrawMode>SHAPE_DRAW_2D</DrawMode>

<ReplicationCode><![CDATA[persons.size()]]></ReplicationCode>

<AsObject>true</AsObject>

<EmbeddedIcon>false</EmbeddedIcon>

<Z>0</Z>

<Rotation>0.0</Rotation>

<DrawingMode>AGENT_CURRENT_POSITION</DrawingMode>

<ScaleType>AUTOMATICALLY_CALCULATED</ScaleType>

</EmbeddedObjectPresentation>

<EmbeddedObjectPresentation>

<Id>1390766198906</Id>

<Name><![CDATA[regionals_presentation]]></Name>

<X>0</X><Y>0</Y>

<Label><X>10</X><Y>0</Y></Label>

<PublicFlag>true</PublicFlag>

<PresentationFlag>true</PresentationFlag>

<ShowLabel>false</ShowLabel>

<DrawMode>SHAPE_DRAW_2D</DrawMode>

<ReplicationCode><![CDATA[regionals.size()]]></ReplicationCode>

<AsObject>true</AsObject>

<EmbeddedIcon>false</EmbeddedIcon>

<Z>0</Z>

<Rotation>0.0</Rotation>

<DrawingMode>AGENT_CURRENT_POSITION</DrawingMode>

<ScaleType>AUTOMATICALLY_CALCULATED</ScaleType>

</EmbeddedObjectPresentation>

<EmbeddedObjectPresentation>

<Id>1390768734138</Id>

<Name><![CDATA[districts_presentation]]></Name>

<X>0</X><Y>0</Y>

<Label><X>10</X><Y>0</Y></Label>

<PublicFlag>true</PublicFlag>

<PresentationFlag>true</PresentationFlag>

<ShowLabel>false</ShowLabel>

<DrawMode>SHAPE_DRAW_2D</DrawMode>

<ReplicationCode><![CDATA[districts.size()]]></ReplicationCode>

<AsObject>true</AsObject>

<EmbeddedIcon>false</EmbeddedIcon>

<Z>0</Z>

<Rotation>0.0</Rotation>

<DrawingMode>AGENT_CURRENT_POSITION</DrawingMode>

<ScaleType>AUTOMATICALLY_CALCULATED</ScaleType>

</EmbeddedObjectPresentation>

<EmbeddedObjectPresentation>

<Id>1390443420968</Id>

<Name><![CDATA[hospitals_presentation]]></Name>

<X>0</X><Y>0</Y>

<Label><X>10</X><Y>0</Y></Label>

<PublicFlag>true</PublicFlag>

<PresentationFlag>true</PresentationFlag>

<ShowLabel>false</ShowLabel>

<DrawMode>SHAPE_DRAW_2D</DrawMode>

<ReplicationCode><![CDATA[hospitals.size()]]></ReplicationCode>

<AsObject>true</AsObject>

<EmbeddedIcon>false</EmbeddedIcon>

<Z>0</Z>

<Rotation>0.0</Rotation>

<DrawingMode>AGENT_CURRENT_POSITION</DrawingMode>

<ScaleType>AUTOMATICALLY_CALCULATED</ScaleType>

</EmbeddedObjectPresentation>

<Text>

<Id>1392676556367</Id>

<Name><![CDATA[text21]]></Name>

<X>-310</X><Y>10</Y>

<Label><X>0</X><Y>-10</Y></Label>

<PublicFlag>true</PublicFlag>

<PresentationFlag>false</PresentationFlag>

<ShowLabel>false</ShowLabel>

<DrawMode>SHAPE_DRAW_2D</DrawMode>

<AsObject>true</AsObject>

<EmbeddedIcon>false</EmbeddedIcon>

<Z>0</Z>

<Rotation>0.0</Rotation>

<Color>-65536</Color>

<Text><![CDATA[Don't use:]]></Text>

<Font>

<Name>SansSerif</Name>

<Size>18</Size>

<Style>3</Style>

</Font>

<Alignment>LEFT</Alignment>

</Text>

<Rectangle>

<Id>1393271432083</Id>

<Name><![CDATA[rectangle8]]></Name>

<X>-530</X><Y>10</Y>

<Label><X>10</X><Y>10</Y></Label>

<PublicFlag>true</PublicFlag>

<PresentationFlag>false</PresentationFlag>

<ShowLabel>false</ShowLabel>

<DrawMode>SHAPE_DRAW_2D3D</DrawMode>

<AsObject>true</AsObject>

<EmbeddedIcon>false</EmbeddedIcon>

<Z>0</Z>

<ZHeight>10</ZHeight>

<LineWidth>1</LineWidth>

<LineColor>-16777216</LineColor>

<LineMaterial>null</LineMaterial>

<LineStyle>SOLID</LineStyle>

<Width>200</Width>

<Height>350</Height>

<Rotation>0.0</Rotation>

<FillColor>-1</FillColor>

<FillMaterial>null</FillMaterial>

</Rectangle>

<Rectangle>

<Id>1393271490547</Id>

<Name><![CDATA[rectangle10]]></Name>

<X>-170</X><Y>10</Y>

<Label><X>10</X><Y>10</Y></Label>

<PublicFlag>true</PublicFlag>

<PresentationFlag>false</PresentationFlag>

<ShowLabel>false</ShowLabel>

<DrawMode>SHAPE_DRAW_2D3D</DrawMode>

<AsObject>true</AsObject>

<EmbeddedIcon>false</EmbeddedIcon>

<Z>0</Z>

<ZHeight>10</ZHeight>

<LineWidth>1</LineWidth>

<LineColor>-16777216</LineColor>

<LineMaterial>null</LineMaterial>

<LineStyle>SOLID</LineStyle>

<Width>130</Width>

<Height>190</Height>

<Rotation>0.0</Rotation>

<FillColor>-1</FillColor>

<FillMaterial>null</FillMaterial>

</Rectangle>

<Text>

<Id>1393271572915</Id>

<Name><![CDATA[text23]]></Name>

<X>-520</X><Y>20</Y>

<Label><X>0</X><Y>-10</Y></Label>

<PublicFlag>true</PublicFlag>

<PresentationFlag>false</PresentationFlag>

<ShowLabel>false</ShowLabel>

<DrawMode>SHAPE_DRAW_2D</DrawMode>

<AsObject>true</AsObject>

<EmbeddedIcon>false</EmbeddedIcon>

<Z>0</Z>

<Rotation>0.0</Rotation>

<Color>-16777216</Color>

<Text><![CDATA[Population parameters]]></Text>

<Font>

<Name>SansSerif</Name>

<Size>14</Size>

<Style>1</Style>

</Font>

<Alignment>LEFT</Alignment>

</Text>

<Text>

<Id>1393271606776</Id>

<Name><![CDATA[text24]]></Name>

<X>-160</X><Y>20</Y>

<Label><X>0</X><Y>-10</Y></Label>

<PublicFlag>true</PublicFlag>

<PresentationFlag>false</PresentationFlag>

<ShowLabel>false</ShowLabel>

<DrawMode>SHAPE_DRAW_2D</DrawMode>

<AsObject>true</AsObject>

<EmbeddedIcon>false</EmbeddedIcon>

<Z>0</Z>

<Rotation>0.0</Rotation>

<Color>-16777216</Color>

<Text><![CDATA[Functions]]></Text>

<Font>

<Name>SansSerif</Name>

<Size>14</Size>

<Style>1</Style>

</Font>

<Alignment>LEFT</Alignment>

</Text>

<Rectangle>

<Id>1393271638940</Id>

<Name><![CDATA[rectangle11]]></Name>

<X>-530</X><Y>370</Y>

<Label><X>10</X><Y>10</Y></Label>

<PublicFlag>true</PublicFlag>

<PresentationFlag>false</PresentationFlag>

<ShowLabel>false</ShowLabel>

<DrawMode>SHAPE_DRAW_2D3D</DrawMode>

<AsObject>true</AsObject>

<EmbeddedIcon>false</EmbeddedIcon>

<Z>0</Z>

<ZHeight>10</ZHeight>

<LineWidth>1</LineWidth>

<LineColor>-16777216</LineColor>

<LineMaterial>null</LineMaterial>

<LineStyle>SOLID</LineStyle>

<Width>350</Width>

<Height>200</Height>

<Rotation>0.0</Rotation>

<FillColor>-1</FillColor>

<FillMaterial>null</FillMaterial>

</Rectangle>

<Text>

<Id>1393271641756</Id>

<Name><![CDATA[text25]]></Name>

<X>-520</X><Y>380</Y>

<Label><X>0</X><Y>-10</Y></Label>

<PublicFlag>true</PublicFlag>

<PresentationFlag>false</PresentationFlag>

<ShowLabel>false</ShowLabel>

<DrawMode>SHAPE_DRAW_2D</DrawMode>

<AsObject>true</AsObject>

<EmbeddedIcon>false</EmbeddedIcon>

<Z>0</Z>

<Rotation>0.0</Rotation>

<Color>-16777216</Color>

<Text><![CDATA[Mortality parameters]]></Text>

<Font>

<Name>SansSerif</Name>

<Size>14</Size>

<Style>1</Style>

</Font>

<Alignment>LEFT</Alignment>

</Text>

<Rectangle>

<Id>1393271693707</Id>

<Name><![CDATA[rectangle12]]></Name>

<X>-720</X><Y>10</Y>

<Label><X>10</X><Y>10</Y></Label>

<PublicFlag>true</PublicFlag>

<PresentationFlag>false</PresentationFlag>

<ShowLabel>false</ShowLabel>

<DrawMode>SHAPE_DRAW_2D3D</DrawMode>

<AsObject>true</AsObject>

<EmbeddedIcon>false</EmbeddedIcon>

<Z>0</Z>

<ZHeight>10</ZHeight>

<LineWidth>1</LineWidth>

<LineColor>-16777216</LineColor>

<LineMaterial>null</LineMaterial>

<LineStyle>SOLID</LineStyle>

<Width>170</Width>

<Height>110</Height>

<Rotation>0.0</Rotation>

<FillColor>-1</FillColor>

<FillMaterial>null</FillMaterial>

</Rectangle>

<Text>

<Id>1393271697776</Id>

<Name><![CDATA[text26]]></Name>

<X>-710</X><Y>20</Y>

<Label><X>0</X><Y>-10</Y></Label>

<PublicFlag>true</PublicFlag>

<PresentationFlag>false</PresentationFlag>

<ShowLabel>false</ShowLabel>

<DrawMode>SHAPE_DRAW_2D</DrawMode>

<AsObject>true</AsObject>

<EmbeddedIcon>false</EmbeddedIcon>

<Z>0</Z>

<Rotation>0.0</Rotation>

<Color>-16777216</Color>

<Text><![CDATA[Display controls]]></Text>

<Font>

<Name>SansSerif</Name>

<Size>14</Size>

<Style>1</Style>

</Font>

<Alignment>LEFT</Alignment>

</Text>

<Rectangle>

<Id>1393271755014</Id>

<Name><![CDATA[rectangle13]]></Name>

<X>-320</X><Y>90</Y>

<Label><X>10</X><Y>10</Y></Label>

<PublicFlag>true</PublicFlag>

<PresentationFlag>false</PresentationFlag>

<ShowLabel>false</ShowLabel>

<DrawMode>SHAPE_DRAW_2D3D</DrawMode>

<AsObject>true</AsObject>

<EmbeddedIcon>false</EmbeddedIcon>

<Z>0</Z>

<ZHeight>10</ZHeight>

<LineWidth>1</LineWidth>

<LineColor>-16777216</LineColor>

<LineMaterial>null</LineMaterial>

<LineStyle>SOLID</LineStyle>

<Width>130</Width>

<Height>160</Height>

<Rotation>0.0</Rotation>

<FillColor>-1</FillColor>

<FillMaterial>null</FillMaterial>

</Rectangle>

<Text>

<Id>1393271759155</Id>

<Name><![CDATA[text27]]></Name>

<X>-310</X><Y>93</Y>

<Label><X>0</X><Y>-10</Y></Label>

<PublicFlag>true</PublicFlag>

<PresentationFlag>false</PresentationFlag>

<ShowLabel>false</ShowLabel>

<DrawMode>SHAPE_DRAW_2D</DrawMode>

<AsObject>true</AsObject>

<EmbeddedIcon>false</EmbeddedIcon>

<Z>0</Z>

<Rotation>0.0</Rotation>

<Color>-16777216</Color>

<Text><![CDATA[Embedded

agents]]></Text>

<Font>

<Name>SansSerif</Name>

<Size>14</Size>

<Style>1</Style>

</Font>

<Alignment>LEFT</Alignment>

</Text>

<Rectangle>

<Id>1393271952701</Id>

<Name><![CDATA[rectangle14]]></Name>

<X>-720</X><Y>130.858407079646</Y>

<Label><X>10</X><Y>10</Y></Label>

<PublicFlag>true</PublicFlag>

<PresentationFlag>false</PresentationFlag>

<ShowLabel>false</ShowLabel>

<DrawMode>SHAPE_DRAW_2D3D</DrawMode>

<AsObject>true</AsObject>

<EmbeddedIcon>false</EmbeddedIcon>

<Z>0</Z>

<ZHeight>10</ZHeight>

<LineWidth>1</LineWidth>

<LineColor>-16777216</LineColor>

<LineMaterial>null</LineMaterial>

<LineStyle>SOLID</LineStyle>

<Width>170</Width>

<Height>440</Height>

<Rotation>0.0</Rotation>

<FillColor>-1</FillColor>

<FillMaterial>null</FillMaterial>

</Rectangle>

<Text>

<Id>1393271979552</Id>

<Name><![CDATA[text28]]></Name>

<X>-710</X><Y>140</Y>

<Label><X>0</X><Y>-10</Y></Label>

<PublicFlag>true</PublicFlag>

<PresentationFlag>false</PresentationFlag>

<ShowLabel>false</ShowLabel>

<DrawMode>SHAPE_DRAW_2D</DrawMode>

<AsObject>true</AsObject>

<EmbeddedIcon>false</EmbeddedIcon>

<Z>0</Z>

<Rotation>0.0</Rotation>

<Color>-16777216</Color>

<Text><![CDATA[Economic params]]></Text>

<Font>

<Name>SansSerif</Name>

<Size>14</Size>

<Style>1</Style>

</Font>

<Alignment>LEFT</Alignment>

</Text>

<Text>

<Id>1394324478235</Id>

<Name><![CDATA[text30]]></Name>

<X>-710</X><Y>520</Y>

<Label><X>0</X><Y>-10</Y></Label>

<PublicFlag>true</PublicFlag>

<PresentationFlag>false</PresentationFlag>

<ShowLabel>false</ShowLabel>

<DrawMode>SHAPE_DRAW_2D</DrawMode>

<AsObject>true</AsObject>

<EmbeddedIcon>false</EmbeddedIcon>

<Z>0</Z>

<Rotation>0.0</Rotation>

<Color>-16777216</Color>

<Text><![CDATA[Logit model params]]></Text>

<Font>

<Name>SansSerif</Name>

<Size>14</Size>

<Style>2</Style>

</Font>

<Alignment>LEFT</Alignment>

</Text>

<Rectangle>

<Id>1410458948920</Id>

<Name><![CDATA[rectangle15]]></Name>

<X>-930</X><Y>10</Y>

<Label><X>10</X><Y>10</Y></Label>

<PublicFlag>true</PublicFlag>

<PresentationFlag>false</PresentationFlag>

<ShowLabel>false</ShowLabel>

<DrawMode>SHAPE_DRAW_2D3D</DrawMode>

<AsObject>true</AsObject>

<EmbeddedIcon>false</EmbeddedIcon>

<Z>0</Z>

<ZHeight>10</ZHeight>

<LineWidth>1</LineWidth>

<LineColor>-16777216</LineColor>

<LineMaterial>null</LineMaterial>

<LineStyle>SOLID</LineStyle>

<Width>180</Width>

<Height>270</Height>

<Rotation>0.0</Rotation>

<FillColor>-1</FillColor>

<FillMaterial>null</FillMaterial>

</Rectangle>

<Text>

<Id>1410458958141</Id>

<Name><![CDATA[text31]]></Name>

<X>-920</X><Y>20</Y>

<Label><X>0</X><Y>-10</Y></Label>

<PublicFlag>true</PublicFlag>

<PresentationFlag>false</PresentationFlag>

<ShowLabel>false</ShowLabel>

<DrawMode>SHAPE_DRAW_2D</DrawMode>

<AsObject>true</AsObject>

<EmbeddedIcon>false</EmbeddedIcon>

<Z>0</Z>

<Rotation>0.0</Rotation>

<Color>-16777216</Color>

<Text><![CDATA[Quality and cost]]></Text>

<Font>

<Name>SansSerif</Name>

<Size>14</Size>

<Style>1</Style>

</Font>

<Alignment>LEFT</Alignment>

</Text>

<Rectangle>

<Id>1410916792405</Id>

<Name><![CDATA[rectangle16]]></Name>

<X>-1670</X><Y>10</Y>

<Label><X>10</X><Y>10</Y></Label>

<PublicFlag>true</PublicFlag>

<PresentationFlag>false</PresentationFlag>

<ShowLabel>false</ShowLabel>

<DrawMode>SHAPE_DRAW_2D3D</DrawMode>

<AsObject>true</AsObject>

<EmbeddedIcon>false</EmbeddedIcon>

<Z>0</Z>

<ZHeight>10</ZHeight>

<LineWidth>1</LineWidth>

<LineColor>-16777216</LineColor>

<LineMaterial>null</LineMaterial>

<LineStyle>SOLID</LineStyle>

<Width>710</Width>

<Height>560</Height>

<Rotation>0.0</Rotation>

<FillColor>-1</FillColor>

<FillMaterial>null</FillMaterial>

</Rectangle>

<Text>

<Id>1410916819578</Id>

<Name><![CDATA[text32]]></Name>

<X>-1660</X><Y>20</Y>

<Label><X>0</X><Y>-10</Y></Label>

<PublicFlag>true</PublicFlag>

<PresentationFlag>false</PresentationFlag>

<ShowLabel>false</ShowLabel>

<DrawMode>SHAPE_DRAW_2D</DrawMode>

<AsObject>true</AsObject>

<EmbeddedIcon>false</EmbeddedIcon>

<Z>0</Z>

<Rotation>0.0</Rotation>

<Color>-16777216</Color>

<Text><![CDATA[Model output]]></Text>

<Font>

<Name>SansSerif</Name>

<Size>14</Size>

<Style>1</Style>

</Font>

<Alignment>LEFT</Alignment>

</Text>

<Rectangle>

<Id>1411582074112</Id>

<Name><![CDATA[rectangle17]]></Name>

<X>-320</X><Y>270</Y>

<Label><X>10</X><Y>10</Y></Label>

<PublicFlag>true</PublicFlag>

<PresentationFlag>false</PresentationFlag>

<ShowLabel>false</ShowLabel>

<DrawMode>SHAPE_DRAW_2D3D</DrawMode>

<AsObject>true</AsObject>

<EmbeddedIcon>false</EmbeddedIcon>

<Z>0</Z>

<ZHeight>10</ZHeight>

<LineWidth>1</LineWidth>

<LineColor>-16777216</LineColor>

<LineMaterial>null</LineMaterial>

<LineStyle>SOLID</LineStyle>

<Width>130</Width>

<Height>90</Height>

<Rotation>0.0</Rotation>

<FillColor>-1</FillColor>

<FillMaterial>null</FillMaterial>

</Rectangle>

<Text>

<Id>1411582088206</Id>

<Name><![CDATA[text33]]></Name>

<X>-310</X><Y>270</Y>

<Label><X>0</X><Y>-10</Y></Label>

<PublicFlag>true</PublicFlag>

<PresentationFlag>false</PresentationFlag>

<ShowLabel>false</ShowLabel>

<DrawMode>SHAPE_DRAW_2D</DrawMode>

<AsObject>true</AsObject>

<EmbeddedIcon>false</EmbeddedIcon>

<Z>0</Z>

<Rotation>0.0</Rotation>

<Color>-16777216</Color>

<Text><![CDATA[Events]]></Text>

<Font>

<Name>SansSerif</Name>

<Size>14</Size>

<Style>1</Style>

</Font>

<Alignment>LEFT</Alignment>

</Text>

<Rectangle>

<Id>1411582183482</Id>

<Name><![CDATA[rectangle18]]></Name>

<X>-170</X><Y>220</Y>

<Label><X>10</X><Y>10</Y></Label>

<PublicFlag>true</PublicFlag>

<PresentationFlag>false</PresentationFlag>

<ShowLabel>false</ShowLabel>

<DrawMode>SHAPE_DRAW_2D3D</DrawMode>

<AsObject>true</AsObject>

<EmbeddedIcon>false</EmbeddedIcon>

<Z>0</Z>

<ZHeight>10</ZHeight>

<LineWidth>1</LineWidth>

<LineColor>-16777216</LineColor>

<LineMaterial>null</LineMaterial>

<LineStyle>SOLID</LineStyle>

<Width>130</Width>

<Height>350</Height>

<Rotation>0.0</Rotation>

<FillColor>-1</FillColor>

<FillMaterial>null</FillMaterial>

</Rectangle>

<Text>

<Id>1411582198610</Id>

<Name><![CDATA[text34]]></Name>

<X>-160</X><Y>220</Y>

<Label><X>0</X><Y>-10</Y></Label>

<PublicFlag>true</PublicFlag>

<PresentationFlag>false</PresentationFlag>

<ShowLabel>false</ShowLabel>

<DrawMode>SHAPE_DRAW_2D</DrawMode>

<AsObject>true</AsObject>

<EmbeddedIcon>false</EmbeddedIcon>

<Z>0</Z>

<Rotation>0.0</Rotation>

<Color>-16777216</Color>

<Text><![CDATA[Tracking]]></Text>

<Font>

<Name>SansSerif</Name>

<Size>14</Size>

<Style>1</Style>

</Font>

<Alignment>LEFT</Alignment>

</Text>

<Rectangle>

<Id>1412534985242</Id>

<Name><![CDATA[rectangle19]]></Name>

<X>-930</X><Y>290</Y>

<Label><X>10</X><Y>10</Y></Label>

<PublicFlag>true</PublicFlag>

<PresentationFlag>false</PresentationFlag>

<ShowLabel>false</ShowLabel>

<DrawMode>SHAPE_DRAW_2D3D</DrawMode>

<AsObject>true</AsObject>

<EmbeddedIcon>false</EmbeddedIcon>

<Z>0</Z>

<ZHeight>10</ZHeight>

<LineWidth>1</LineWidth>

<LineColor>-16777216</LineColor>

<LineMaterial>null</LineMaterial>

<LineStyle>SOLID</LineStyle>

<Width>180</Width>

<Height>280</Height>

<Rotation>0.0</Rotation>

<FillColor>-1</FillColor>

<FillMaterial>null</FillMaterial>

</Rectangle>

<Text>

<Id>1412534996253</Id>

<Name><![CDATA[text29]]></Name>

<X>-920</X><Y>300</Y>

<Label><X>0</X><Y>-10</Y></Label>

<PublicFlag>true</PublicFlag>

<PresentationFlag>false</PresentationFlag>

<ShowLabel>false</ShowLabel>

<DrawMode>SHAPE_DRAW_2D</DrawMode>

<AsObject>true</AsObject>

<EmbeddedIcon>false</EmbeddedIcon>

<Z>0</Z>

<Rotation>0.0</Rotation>

<Color>-16777216</Color>

<Text><![CDATA[Policy levers]]></Text>

<Font>

<Name>SansSerif</Name>

<Size>14</Size>

<Style>1</Style>

</Font>

<Alignment>LEFT</Alignment>

</Text>

</Shapes>

</ActiveObjectClass>

<!-- ========= Active Object Class ======== -->

<ActiveObjectClass>

<Id>1390441248426</Id>

<Name><![CDATA[Hospital]]></Name>

<ClientAreaTopLeft><X>0</X><Y>0</Y></ClientAreaTopLeft>

<PresentationTopGroupPersistent>true</PresentationTopGroupPersistent>

<IconTopGroupPersistent>true</IconTopGroupPersistent>

<Import><![CDATA[//import quicktime.streaming.NewPresentationParams;]]></Import>

<StartupCode><![CDATA[jumpTo(longitude,latitude);

]]></StartupCode>

<Generic>false</Generic>

<GenericParameters><![CDATA[T]]></GenericParameters>

<GenericParametersLabel><![CDATA[Generic parameters:]]></GenericParametersLabel>

<FlowChartsUsage>ENTITY</FlowChartsUsage>

<SamplesToKeep>100</SamplesToKeep>

<LimitNumberOfArrayElements>false</LimitNumberOfArrayElements>

<ElementsLimitValue>100</ElementsLimitValue>

<MakeDefaultViewArea>true</MakeDefaultViewArea>

<SceneGridColor/>

<SceneBackgroundColor/>

<AgentProperties>

<SpaceType>GIS</SpaceType>

<EnvironmentDefinesInitialLocation>true</EnvironmentDefinesInitialLocation>

<RotateAnimationTowardsMovement>false</RotateAnimationTowardsMovement>

<RotateAnimationVertically>false</RotateAnimationVertically>

<VelocityCode><![CDATA[10]]></VelocityCode>

<VelocityGISCode><![CDATA[10]]></VelocityGISCode>

</AgentProperties>

<EnvironmentProperties>

<EnableSteps>false</EnableSteps>

<StepDurationCode><![CDATA[1.0]]></StepDurationCode>

<SpaceType>CONTINUOUS</SpaceType>

<WidthCode><![CDATA[500]]></WidthCode>

<HeightCode><![CDATA[500]]></HeightCode>

<ZHeightCode><![CDATA[0]]></ZHeightCode>

<ColumnsCountCode><![CDATA[100]]></ColumnsCountCode>

<RowsCountCode><![CDATA[100]]></RowsCountCode>

<NeigborhoodType>MOORE</NeigborhoodType>

<LayoutType>USER_DEF</LayoutType>

<LayoutTypeApplyOnStartup>true</LayoutTypeApplyOnStartup>

<NetworkType>USER_DEF</NetworkType>

<NetworkTypeApplyOnStartup>true</NetworkTypeApplyOnStartup>

<ConnectionsPerAgentCode><![CDATA[2]]></ConnectionsPerAgentCode>

<ConnectionsRangeCode><![CDATA[50]]></ConnectionsRangeCode>

<NeighborLinkFractionCode><![CDATA[0.95]]></NeighborLinkFractionCode>

<MCode><![CDATA[10]]></MCode>

</EnvironmentProperties>

<DatasetsCreationProperties>

<AutoCreate>true</AutoCreate>

<RecurrenceCode><![CDATA[1]]></RecurrenceCode>

</DatasetsCreationProperties>

<ConnectionsId>1392508625679</ConnectionsId>

<Variables>

<Variable Class="PlainVariable">

<Id>1394323600116</Id>

<Name><![CDATA[agentValue]]></Name>

<X>-110</X><Y>40</Y>

<Label><X>10</X><Y>0</Y></Label>

<PublicFlag>false</PublicFlag>

<PresentationFlag>true</PresentationFlag>

<ShowLabel>true</ShowLabel>

<Properties SaveInSnapshot="true" Constant="false" AccessType="public" StaticVariable="false">

<Type><![CDATA[double]]></Type>

</Properties>

</Variable>

<Variable Class="PlainVariable">

<Id>1412538249815</Id>

<Name><![CDATA[transitionDay]]></Name>

<X>-110</X><Y>60</Y>

<Label><X>10</X><Y>0</Y></Label>

<PublicFlag>false</PublicFlag>

<PresentationFlag>true</PresentationFlag>

<ShowLabel>true</ShowLabel>

<Properties SaveInSnapshot="true" Constant="false" AccessType="public" StaticVariable="false">

<Type><![CDATA[int]]></Type>

</Properties>

</Variable>

<Variable Class="PlainVariable">

<Id>1412538643119</Id>

<Name><![CDATA[baselineCost]]></Name>

<X>-110</X><Y>100</Y>

<Label><X>10</X><Y>0</Y></Label>

<PublicFlag>false</PublicFlag>

<PresentationFlag>true</PresentationFlag>

<ShowLabel>true</ShowLabel>

<Properties SaveInSnapshot="true" Constant="false" AccessType="public" StaticVariable="false">

<Type><![CDATA[double]]></Type>

</Properties>

</Variable>

<Variable Class="PlainVariable">

<Id>1412538652186</Id>

<Name><![CDATA[baselineNonMed]]></Name>

<X>-110</X><Y>120</Y>

<Label><X>10</X><Y>0</Y></Label>

<PublicFlag>false</PublicFlag>

<PresentationFlag>true</PresentationFlag>

<ShowLabel>true</ShowLabel>

<Properties SaveInSnapshot="true" Constant="false" AccessType="public" StaticVariable="false">

<Type><![CDATA[double]]></Type>

</Properties>

</Variable>

<Variable Class="PlainVariable">

<Id>1412538662460</Id>

<Name><![CDATA[baselineQuality]]></Name>

<X>-110</X><Y>140</Y>

<Label><X>10</X><Y>0</Y></Label>

<PublicFlag>false</PublicFlag>

<PresentationFlag>true</PresentationFlag>

<ShowLabel>true</ShowLabel>

<Properties SaveInSnapshot="true" Constant="false" AccessType="public" StaticVariable="false">

<Type><![CDATA[double]]></Type>

</Properties>

</Variable>

<Variable Class="PlainVariable">

<Id>1412538714939</Id>

<Name><![CDATA[baselinePriv]]></Name>

<X>-110</X><Y>160</Y>

<Label><X>10</X><Y>0</Y></Label>

<PublicFlag>false</PublicFlag>

<PresentationFlag>true</PresentationFlag>

<ShowLabel>true</ShowLabel>

<Properties SaveInSnapshot="true" Constant="false" AccessType="public" StaticVariable="false">

<Type><![CDATA[int]]></Type>

</Properties>

</Variable>

<Variable Class="PlainVariable">

<Id>1412538733768</Id>

<Name><![CDATA[baselineNGO]]></Name>

<X>-110</X><Y>180</Y>

<Label><X>10</X><Y>0</Y></Label>

<PublicFlag>false</PublicFlag>

<PresentationFlag>true</PresentationFlag>

<ShowLabel>true</ShowLabel>

<Properties SaveInSnapshot="true" Constant="false" AccessType="public" StaticVariable="false">

<Type><![CDATA[boolean]]></Type>

</Properties>

</Variable>

<Variable Class="PlainVariable">

<Id>1412736087057</Id>

<Name><![CDATA[twoWeek]]></Name>

<Description><![CDATA[1 if a two-week trip is present. 0 otherwise]]></Description>

<X>-110</X><Y>210</Y>

<Label><X>10</X><Y>0</Y></Label>

<PublicFlag>false</PublicFlag>

<PresentationFlag>true</PresentationFlag>

<ShowLabel>true</ShowLabel>

<Properties SaveInSnapshot="true" Constant="false" AccessType="public" StaticVariable="false">

<Type><![CDATA[double]]></Type>

<InitialValue><![CDATA[0.0]]></InitialValue>

</Properties>

</Variable>

<Variable Class="Parameter">

<Id>1390442419034</Id>

<Name><![CDATA[name]]></Name>

<X>-230</X><Y>40</Y>

<Label><X>10</X><Y>0</Y></Label>

<PublicFlag>false</PublicFlag>

<PresentationFlag>true</PresentationFlag>

<ShowLabel>true</ShowLabel>

<Properties SaveInSnapshot="true" ModificatorType="STATIC">

<Type><![CDATA[String]]></Type>

<SdArray>false</SdArray>

<DefaultValue><![CDATA[]]></DefaultValue>

<ParameterEditor>

<Id>1390442419032</Id>

<Name><![CDATA[]]></Name>

<EditorContolType>TEXT_BOX</EditorContolType>

<MinSliderValue><![CDATA[0]]></MinSliderValue>

<MaxSliderValue><![CDATA[100]]></MaxSliderValue>

<DelimeterType>NO_DELIMETER</DelimeterType>

</ParameterEditor>

</Properties>

</Variable>

<Variable Class="Parameter">

<Id>1390442429567</Id>

<Name><![CDATA[priv]]></Name>

<Description><![CDATA[Is the hospital a private hospital?]]></Description>

<X>-230</X><Y>60</Y>

<Label><X>10</X><Y>0</Y></Label>

<PublicFlag>false</PublicFlag>

<PresentationFlag>true</PresentationFlag>

<ShowLabel>true</ShowLabel>

<Properties SaveInSnapshot="true" ModificatorType="STATIC">

<Type><![CDATA[int]]></Type>

<SdArray>false</SdArray>

<DefaultValue><![CDATA[]]></DefaultValue>

<ParameterEditor>

<Id>1390442429565</Id>

<Name><![CDATA[]]></Name>

<EditorContolType>TEXT_BOX</EditorContolType>

<MinSliderValue><![CDATA[0]]></MinSliderValue>

<MaxSliderValue><![CDATA[100]]></MaxSliderValue>

<DelimeterType>NO_DELIMETER</DelimeterType>

</ParameterEditor>

</Properties>

</Variable>

<Variable Class="Parameter">

<Id>1390442439137</Id>

<Name><![CDATA[latitude]]></Name>

<X>-230</X><Y>80</Y>

<Label><X>10</X><Y>0</Y></Label>

<PublicFlag>false</PublicFlag>

<PresentationFlag>true</PresentationFlag>

<ShowLabel>true</ShowLabel>

<Properties SaveInSnapshot="true" ModificatorType="STATIC">

<Type><![CDATA[double]]></Type>

<SdArray>false</SdArray>

<DefaultValue><![CDATA[]]></DefaultValue>

<ParameterEditor>

<Id>1390442439135</Id>

<Name><![CDATA[]]></Name>

<EditorContolType>TEXT_BOX</EditorContolType>

<MinSliderValue><![CDATA[0]]></MinSliderValue>

<MaxSliderValue><![CDATA[100]]></MaxSliderValue>

<DelimeterType>NO_DELIMETER</DelimeterType>

</ParameterEditor>

</Properties>

</Variable>

<Variable Class="Parameter">

<Id>1390442447012</Id>

<Name><![CDATA[longitude]]></Name>

<X>-230</X><Y>100</Y>

<Label><X>10</X><Y>0</Y></Label>

<PublicFlag>false</PublicFlag>

<PresentationFlag>true</PresentationFlag>

<ShowLabel>true</ShowLabel>

<Properties SaveInSnapshot="true" ModificatorType="STATIC">

<Type><![CDATA[double]]></Type>

<SdArray>false</SdArray>

<DefaultValue><![CDATA[]]></DefaultValue>

<ParameterEditor>

<Id>1390442447010</Id>

<Name><![CDATA[]]></Name>

<EditorContolType>TEXT_BOX</EditorContolType>

<MinSliderValue><![CDATA[0]]></MinSliderValue>

<MaxSliderValue><![CDATA[100]]></MaxSliderValue>

<DelimeterType>NO_DELIMETER</DelimeterType>

</ParameterEditor>

</Properties>

</Variable>

<Variable Class="Parameter">

<Id>1410459476484</Id>

<Name><![CDATA[quality]]></Name>

<X>-230</X><Y>120</Y>

<Label><X>10</X><Y>0</Y></Label>

<PublicFlag>false</PublicFlag>

<PresentationFlag>true</PresentationFlag>

<ShowLabel>true</ShowLabel>

<Properties SaveInSnapshot="true" ModificatorType="STATIC">

<Type><![CDATA[double]]></Type>

<SdArray>false</SdArray>

<DefaultValue><![CDATA[0]]></DefaultValue>

<ParameterEditor>

<Id>1410459476481</Id>

<Name><![CDATA[]]></Name>

<EditorContolType>TEXT_BOX</EditorContolType>

<MinSliderValue><![CDATA[0]]></MinSliderValue>

<MaxSliderValue><![CDATA[100]]></MaxSliderValue>

<DelimeterType>NO_DELIMETER</DelimeterType>

</ParameterEditor>

</Properties>

</Variable>

<Variable Class="Parameter">

<Id>1410472481270</Id>

<Name><![CDATA[NGO]]></Name>

<X>-230</X><Y>140</Y>

<Label><X>10</X><Y>0</Y></Label>

<PublicFlag>false</PublicFlag>

<PresentationFlag>true</PresentationFlag>

<ShowLabel>true</ShowLabel>

<Properties SaveInSnapshot="true" ModificatorType="STATIC">

<Type><![CDATA[boolean]]></Type>

<SdArray>false</SdArray>

<DefaultValue><![CDATA[false]]></DefaultValue>

<ParameterEditor>

<Id>1410472481268</Id>

<Name><![CDATA[]]></Name>

<EditorContolType>CHECK_BOX</EditorContolType>

<MinSliderValue><![CDATA[0]]></MinSliderValue>

<MaxSliderValue><![CDATA[100]]></MaxSliderValue>

<DelimeterType>NO_DELIMETER</DelimeterType>

</ParameterEditor>

</Properties>

</Variable>

<Variable Class="Parameter">

<Id>1410472521445</Id>

<Name><![CDATA[cost]]></Name>

<X>-230</X><Y>160</Y>

<Label><X>10</X><Y>0</Y></Label>

<PublicFlag>false</PublicFlag>

<PresentationFlag>true</PresentationFlag>

<ShowLabel>true</ShowLabel>

<Properties SaveInSnapshot="true" ModificatorType="STATIC">

<Type><![CDATA[double]]></Type>

<SdArray>false</SdArray>

<DefaultValue><![CDATA[NGO ? 0 : get_Main().cSurg]]></DefaultValue>

<ParameterEditor>

<Id>1410472521443</Id>

<Name><![CDATA[]]></Name>

<EditorContolType>TEXT_BOX</EditorContolType>

<MinSliderValue><![CDATA[0]]></MinSliderValue>

<MaxSliderValue><![CDATA[100]]></MaxSliderValue>

<DelimeterType>NO_DELIMETER</DelimeterType>

</ParameterEditor>

</Properties>

</Variable>

<Variable Class="Parameter">

<Id>1410472555999</Id>

<Name><![CDATA[nonmed]]></Name>

<X>-230</X><Y>180</Y>

<Label><X>10</X><Y>0</Y></Label>

<PublicFlag>false</PublicFlag>

<PresentationFlag>true</PresentationFlag>

<ShowLabel>true</ShowLabel>

<Properties SaveInSnapshot="true" ModificatorType="STATIC">

<Type><![CDATA[double]]></Type>

<SdArray>false</SdArray>

<DefaultValue><![CDATA[NGO ? 0 : get_Main().mNonMed * get_Main().cSurg]]></DefaultValue>

<ParameterEditor>

<Id>1410472555997</Id>

<Name><![CDATA[]]></Name>

<EditorContolType>TEXT_BOX</EditorContolType>

<MinSliderValue><![CDATA[0]]></MinSliderValue>

<MaxSliderValue><![CDATA[100]]></MaxSliderValue>

<DelimeterType>NO_DELIMETER</DelimeterType>

</ParameterEditor>

</Properties>

</Variable>

<Variable Class="Parameter">

<Id>1412615693393</Id>

<Name><![CDATA[amIMercyShips]]></Name>

<X>-230</X><Y>200</Y>

<Label><X>10</X><Y>0</Y></Label>

<PublicFlag>false</PublicFlag>

<PresentationFlag>true</PresentationFlag>

<ShowLabel>true</ShowLabel>

<Properties SaveInSnapshot="true" ModificatorType="STATIC">

<Type><![CDATA[boolean]]></Type>

<SdArray>false</SdArray>

<DefaultValue><![CDATA[]]></DefaultValue>

<ParameterEditor>

<Id>1412615693391</Id>

<Name><![CDATA[]]></Name>

<EditorContolType>CHECK_BOX</EditorContolType>

<MinSliderValue><![CDATA[0]]></MinSliderValue>

<MaxSliderValue><![CDATA[100]]></MaxSliderValue>

<DelimeterType>NO_DELIMETER</DelimeterType>

</ParameterEditor>

</Properties>

</Variable>

<Variable Class="Parameter">

<Id>1413042559421</Id>

<Name><![CDATA[amITwoWeek]]></Name>

<X>-230</X><Y>220</Y>

<Label><X>10</X><Y>0</Y></Label>

<PublicFlag>false</PublicFlag>

<PresentationFlag>true</PresentationFlag>

<ShowLabel>true</ShowLabel>

<Properties SaveInSnapshot="true" ModificatorType="STATIC">

<Type><![CDATA[boolean]]></Type>

<SdArray>false</SdArray>

<DefaultValue><![CDATA[]]></DefaultValue>

<ParameterEditor>

<Id>1413042559419</Id>

<Name><![CDATA[]]></Name>

<EditorContolType>CHECK_BOX</EditorContolType>

<MinSliderValue><![CDATA[0]]></MinSliderValue>

<MaxSliderValue><![CDATA[100]]></MaxSliderValue>

<DelimeterType>NO_DELIMETER</DelimeterType>

</ParameterEditor>

</Properties>

</Variable>

</Variables>

<StatechartElements>

<StatechartElement Class="State" ParentState="ROOT_NODE">

<Id>1412614421943</Id>

<Name><![CDATA[twoWeekTrip]]></Name>

<X>80</X><Y>40</Y>

<Label><X>10</X><Y>10</Y></Label>

<PublicFlag>false</PublicFlag>

<PresentationFlag>true</PresentationFlag>

<ShowLabel>true</ShowLabel>

<Properties Width="170" Height="210">

<FillColor>-4138560</FillColor>

</Properties>

</StatechartElement>

<StatechartElement Class="State" ParentState="twoWeekTrip" ParentStateId="1412614421943">

<Id>1412538183121</Id>

<Name><![CDATA[Trip]]></Name>

<X>110</X><Y>180</Y>

<Label><X>10</X><Y>10</Y></Label>

<PublicFlag>false</PublicFlag>

<PresentationFlag>true</PresentationFlag>

<ShowLabel>true</ShowLabel>

<Properties Width="100" Height="30">

<EntryAction><![CDATA[//add back to the NGO list

if(!get_Main().allHospitalsNGO.contains(this)) get_Main().allHospitalsNGO.add(this);

this.rectangle.setVisible(true);

this.textName.setColor(black);

this.textName.setText(this.name);

//calculate the day the trip leaves

//(average two weeks)

double p = 1.0 / (1.0 + get_Main().meanTripLength);

transitionDay = geometric(p);]]></EntryAction>

<FillColor>-7357297</FillColor>

</Properties>

</StatechartElement>

<StatechartElement Class="State" ParentState="twoWeekTrip" ParentStateId="1412614421943">

<Id>1412538171855</Id>

<Name><![CDATA[NoTrip]]></Name>

<X>110</X><Y>80</Y>

<Label><X>10</X><Y>10</Y></Label>

<PublicFlag>false</PublicFlag>

<PresentationFlag>true</PresentationFlag>

<ShowLabel>true</ShowLabel>

<Properties Width="100" Height="30">

<EntryAction><![CDATA[//remove from the NGO list

if(get_Main().allHospitalsNGO.contains(this)) get_Main().allHospitalsNGO.remove(this);

this.rectangle.setVisible(false);

this.textName.setColor(new Color(255, 249, 239));

this.textName.setText("");

//calculate the day a trip arrives

if(get_Main().twoWeekTrips) {

double avgWait = (double) 365.0 / get_Main().numTripsPerYear;

double p = (double) 1.0 / ((double) 1.0 + avgWait);

transitionDay = geometric(p);

}

]]></EntryAction>

<FillColor>-7357297</FillColor>

</Properties>

</StatechartElement>

<StatechartElement Class="State" ParentState="ROOT_NODE">

<Id>1412614519305</Id>

<Name><![CDATA[MercyShips]]></Name>

<X>350</X><Y>40</Y>

<Label><X>10</X><Y>10</Y></Label>

<PublicFlag>false</PublicFlag>

<PresentationFlag>true</PresentationFlag>

<ShowLabel>true</ShowLabel>

<Properties Width="400" Height="280">

<FillColor>-5192482</FillColor>

</Properties>

</StatechartElement>

<StatechartElement Class="Branch" ParentState="ROOT_NODE">

<Id>1412614594690</Id>

<Name><![CDATA[branchMercyShips]]></Name>

<X>300</X><Y>60</Y>

<Label><X>10</X><Y>0</Y></Label>

<PublicFlag>false</PublicFlag>

<PresentationFlag>true</PresentationFlag>

<ShowLabel>false</ShowLabel>

<Properties>

</Properties>

</StatechartElement>

<StatechartElement Class="State" ParentState="MercyShips" ParentStateId="1412614519305">

<Id>1412614688306</Id>

<Name><![CDATA[NotPresent]]></Name>

<X>370</X><Y>80</Y>

<Label><X>10</X><Y>10</Y></Label>

<PublicFlag>false</PublicFlag>

<PresentationFlag>true</PresentationFlag>

<ShowLabel>true</ShowLabel>

<Properties Width="100" Height="30">

<EntryAction><![CDATA[this.rectangle.setVisible(false);

this.textName.setColor(new Color(255, 249, 239));

this.textName.setText("")]]></EntryAction>

<ExitAction><![CDATA[this.rectangle.setVisible(true);

this.textName.setColor(black);

this.textName.setText(this.name);

]]></ExitAction>

<FillColor>-7427377</FillColor>

</Properties>

</StatechartElement>

<StatechartElement Class="State" ParentState="MercyShips" ParentStateId="1412614519305">

<Id>1412614906536</Id>

<Name><![CDATA[Present]]></Name>

<X>520</X><Y>80</Y>

<Label><X>10</X><Y>10</Y></Label>

<PublicFlag>false</PublicFlag>

<PresentationFlag>true</PresentationFlag>

<ShowLabel>true</ShowLabel>

<Properties Width="150" Height="210">

<FillColor>-7427377</FillColor>

</Properties>

</StatechartElement>

<StatechartElement Class="State" ParentState="Present" ParentStateId="1412614906536">

<Id>1412615061164</Id>

<Name><![CDATA[Moving]]></Name>

<X>540</X><Y>120</Y>

<Label><X>10</X><Y>10</Y></Label>

<PublicFlag>false</PublicFlag>

<PresentationFlag>true</PresentationFlag>

<ShowLabel>true</ShowLabel>

<Properties Width="100" Height="30">

<EntryAction><![CDATA[//remove this hospital from the list of possible treatment places

if(get_Main().allHospitalsNGO.contains(this)) get_Main().allHospitalsNGO.remove(this);

//pick the location

double moveLat = get_Main().randomAgentInside().getLat();

double moveLon = get_Main().randomAgentInside().getLon();

//make sure the point is on the map

while(get_Main().Uganda.getLayers()[1].findPoliticalArea(moveLon, moveLat) == null) {

moveLat = get_Main().randomAgentInside().getLat();

moveLon = get_Main().randomAgentInside().getLon();

}

//move, and set your new coordinates to where you've moved to

this.moveTo(moveLon, moveLat);

this.latitude = moveLat;

this.longitude = moveLon;]]></EntryAction>

<FillColor>-11765583</FillColor>

</Properties>

</StatechartElement>

<StatechartElement Class="State" ParentState="Present" ParentStateId="1412614906536">

<Id>1412615230678</Id>

<Name><![CDATA[Treating]]></Name>

<X>540</X><Y>210</Y>

<Label><X>10</X><Y>10</Y></Label>

<PublicFlag>false</PublicFlag>

<PresentationFlag>true</PresentationFlag>

<ShowLabel>true</ShowLabel>

<Properties Width="100" Height="30">

<EntryAction><![CDATA[//add this hospital to the list of possible hospitals

if(!get_Main().allHospitalsNGO.contains(this)) get_Main().allHospitalsNGO.add(this);

]]></EntryAction>

<FillColor>-11765583</FillColor>

</Properties>

</StatechartElement>

<StatechartElement Class="State" ParentState="ROOT_NODE">

<Id>1413043285677</Id>

<Name><![CDATA[stationaryHospital]]></Name>

<X>80</X><Y>300</Y>

<Label><X>10</X><Y>10</Y></Label>

<PublicFlag>false</PublicFlag>

<PresentationFlag>true</PresentationFlag>

<ShowLabel>true</ShowLabel>

<Properties Width="170" Height="90">

<FillColor>-657956</FillColor>

</Properties>

</StatechartElement>

<StatechartElement Class="EntryPoint" ParentState="ROOT_NODE">

<Id>1412538193772</Id>

<Name><![CDATA[statechart]]></Name>

<X>300</X><Y>20</Y>

<Label><X>10</X><Y>0</Y></Label>

<PublicFlag>false</PublicFlag>

<PresentationFlag>true</PresentationFlag>

<ShowLabel>true</ShowLabel>

<Points>

<Point><X>0</X><Y>0</Y></Point>

<Point><X>0</X><Y>30</Y></Point>

</Points>

<Properties Target="1412614594690">

<Action><![CDATA[//catalog the baseline parameters before trips arrive

baselineCost = cost;

baselineNonMed = nonmed;

baselineQuality = quality;

baselinePriv = priv;

baselineNGO = NGO;]]></Action>

</Properties>

</StatechartElement>

<StatechartElement Class="Transition" ParentState="twoWeekTrip" ParentStateId="1412614421943">

<Id>1412538198315</Id>

<Name><![CDATA[transition]]></Name>

<X>120</X><Y>110</Y>

<Label><X>10</X><Y>0</Y></Label>

<PublicFlag>false</PublicFlag>

<PresentationFlag>true</PresentationFlag>

<ShowLabel>false</ShowLabel>

<Points>

<Point><X>0</X><Y>0</Y></Point>

<Point><X>0</X><Y>70</Y></Point>

</Points>

<Properties Source="1412538171855" Target="1412538183121" Trigger="timeout">

<Timeout><![CDATA[transitionDay]]></Timeout>

<Condition><![CDATA[true]]></Condition>

<Rate><![CDATA[1]]></Rate>

<MessageType><![CDATA[Object]]></MessageType>

<DefaultTransition>true</DefaultTransition>

<FilterType><![CDATA[unconditionally]]></FilterType>

<EqualsExpression><![CDATA["text"]]></EqualsExpression>

<SatisfiesExpression><![CDATA[true]]></SatisfiesExpression>

</Properties>

</StatechartElement>

<StatechartElement Class="Transition" ParentState="twoWeekTrip" ParentStateId="1412614421943">

<Id>1412538206109</Id>

<Name><![CDATA[transition1]]></Name>

<X>200</X><Y>180</Y>

<Label><X>10</X><Y>0</Y></Label>

<PublicFlag>false</PublicFlag>

<PresentationFlag>true</PresentationFlag>

<ShowLabel>false</ShowLabel>

<Points>

<Point><X>0</X><Y>0</Y></Point>

<Point><X>0</X><Y>-70</Y></Point>

</Points>

<Properties Source="1412538183121" Target="1412538171855" Trigger="timeout">

<Timeout><![CDATA[transitionDay]]></Timeout>

<Condition><![CDATA[true]]></Condition>

<Rate><![CDATA[1]]></Rate>

<MessageType><![CDATA[Object]]></MessageType>

<DefaultTransition>true</DefaultTransition>

<FilterType><![CDATA[unconditionally]]></FilterType>

<EqualsExpression><![CDATA["text"]]></EqualsExpression>

<SatisfiesExpression><![CDATA[true]]></SatisfiesExpression>

</Properties>

</StatechartElement>

<StatechartElement Class="Transition" ParentState="ROOT_NODE">

<Id>1412614598750</Id>

<Name><![CDATA[transition2]]></Name>

<X>288</X><Y>60</Y>

<Label><X>10</X><Y>0</Y></Label>

<PublicFlag>false</PublicFlag>

<PresentationFlag>true</PresentationFlag>

<ShowLabel>false</ShowLabel>

<Points>

<Point><X>0</X><Y>0</Y></Point>

<Point><X>-38</X><Y>0</Y></Point>

</Points>

<Properties Source="1412614594690" Target="1412614421943" Trigger="timeout">

<Timeout><![CDATA[1]]></Timeout>

<Condition><![CDATA[amITwoWeek]]></Condition>

<Rate><![CDATA[1]]></Rate>

<MessageType><![CDATA[Object]]></MessageType>

<DefaultTransition>false</DefaultTransition>

<FilterType><![CDATA[unconditionally]]></FilterType>

<EqualsExpression><![CDATA["text"]]></EqualsExpression>

<SatisfiesExpression><![CDATA[true]]></SatisfiesExpression>

</Properties>

</StatechartElement>

<StatechartElement Class="Transition" ParentState="ROOT_NODE">

<Id>1412614610199</Id>

<Name><![CDATA[transition3]]></Name>

<X>312</X><Y>60</Y>

<Label><X>10</X><Y>0</Y></Label>

<PublicFlag>false</PublicFlag>

<PresentationFlag>true</PresentationFlag>

<ShowLabel>false</ShowLabel>

<Points>

<Point><X>0</X><Y>0</Y></Point>

<Point><X>38</X><Y>0</Y></Point>

</Points>

<Properties Source="1412614594690" Target="1412614519305" Trigger="timeout">

<Timeout><![CDATA[1]]></Timeout>

<Condition><![CDATA[amIMercyShips]]></Condition>

<Rate><![CDATA[1]]></Rate>

<MessageType><![CDATA[Object]]></MessageType>

<DefaultTransition>false</DefaultTransition>

<FilterType><![CDATA[unconditionally]]></FilterType>

<EqualsExpression><![CDATA["text"]]></EqualsExpression>

<SatisfiesExpression><![CDATA[true]]></SatisfiesExpression>

</Properties>

</StatechartElement>

<StatechartElement Class="Transition" ParentState="MercyShips" ParentStateId="1412614519305">

<Id>1412615262898</Id>

<Name><![CDATA[transition4]]></Name>

<X>470</X><Y>90</Y>

<Label><X>10</X><Y>0</Y></Label>

<PublicFlag>false</PublicFlag>

<PresentationFlag>true</PresentationFlag>

<ShowLabel>false</ShowLabel>

<Points>

<Point><X>0</X><Y>0</Y></Point>

<Point><X>20</X><Y>0</Y></Point>

<Point><X>50</X><Y>0</Y></Point>

</Points>

<Properties Source="1412614688306" Target="1412614906536" Trigger="timeout">

<Action><![CDATA[

//add this hospital to the list of possible hospitals

if(!get_Main().allHospitalsNGO.contains(this)) get_Main().allHospitalsNGO.add(this);

//re-set position to Kampala (0.341366667,32.577133333), as long as there is time between the missions.

if(get_Main().yearsBetweenMS > 0) {

this.jumpTo(32.577133333, 0.341366667);

this.latitude = 0.341366667;

this.longitude = 32.577133333;

}]]></Action>

<Timeout><![CDATA[geometric(1.0/(1.0+365*get_Main().yearsBetweenMS))]]></Timeout>

<Condition><![CDATA[true]]></Condition>

<Rate><![CDATA[1]]></Rate>

<MessageType><![CDATA[Object]]></MessageType>

<DefaultTransition>true</DefaultTransition>

<FilterType><![CDATA[unconditionally]]></FilterType>

<EqualsExpression><![CDATA["text"]]></EqualsExpression>

<SatisfiesExpression><![CDATA[true]]></SatisfiesExpression>

</Properties>

</StatechartElement>

<StatechartElement Class="Transition" ParentState="MercyShips" ParentStateId="1412614519305">

<Id>1412615278378</Id>

<Name><![CDATA[transition5]]></Name>

<X>520</X><Y>100</Y>

<Label><X>10</X><Y>0</Y></Label>

<PublicFlag>false</PublicFlag>

<PresentationFlag>true</PresentationFlag>

<ShowLabel>false</ShowLabel>

<Points>

<Point><X>0</X><Y>0</Y></Point>

<Point><X>-50</X><Y>0</Y></Point>

</Points>

<Properties Source="1412614906536" Target="1412614688306" Trigger="timeout">

<Action><![CDATA[//remove this hospital from the list of possible hospitals

if(get_Main().allHospitalsNGO.contains(this)) get_Main().allHospitalsNGO.remove(this);

]]></Action>

<Timeout><![CDATA[geometric(0.00273224)]]></Timeout>

<Condition><![CDATA[true]]></Condition>

<Rate><![CDATA[1]]></Rate>

<MessageType><![CDATA[Object]]></MessageType>

<DefaultTransition>true</DefaultTransition>

<FilterType><![CDATA[unconditionally]]></FilterType>

<EqualsExpression><![CDATA["text"]]></EqualsExpression>

<SatisfiesExpression><![CDATA[true]]></SatisfiesExpression>

</Properties>

</StatechartElement>

<StatechartElement Class="Transition" ParentState="Present" ParentStateId="1412614906536">

<Id>1412615305930</Id>

<Name><![CDATA[transition6]]></Name>

<X>560</X><Y>150</Y>

<Label><X>10</X><Y>0</Y></Label>

<PublicFlag>false</PublicFlag>

<PresentationFlag>true</PresentationFlag>

<ShowLabel>false</ShowLabel>

<Points>

<Point><X>0</X><Y>0</Y></Point>

<Point><X>0</X><Y>60</Y></Point>

</Points>

<Properties Source="1412615061164" Target="1412615230678" Trigger="arrival">

<Timeout><![CDATA[1]]></Timeout>

<Condition><![CDATA[true]]></Condition>

<Rate><![CDATA[1]]></Rate>

<MessageType><![CDATA[Object]]></MessageType>

<DefaultTransition>true</DefaultTransition>

<FilterType><![CDATA[unconditionally]]></FilterType>

<EqualsExpression><![CDATA["text"]]></EqualsExpression>

<SatisfiesExpression><![CDATA[true]]></SatisfiesExpression>

</Properties>

</StatechartElement>

<StatechartElement Class="Transition" ParentState="Present" ParentStateId="1412614906536">

<Id>1412615312926</Id>

<Name><![CDATA[transition7]]></Name>

<X>620</X><Y>210</Y>

<Label><X>10</X><Y>0</Y></Label>

<PublicFlag>false</PublicFlag>

<PresentationFlag>true</PresentationFlag>

<ShowLabel>false</ShowLabel>

<Points>

<Point><X>0</X><Y>0</Y></Point>

<Point><X>0</X><Y>-60</Y></Point>

</Points>

<Properties Source="1412615230678" Target="1412615061164" Trigger="timeout">

<Timeout><![CDATA[geometric(0.010989011)]]></Timeout>

<Condition><![CDATA[true]]></Condition>

<Rate><![CDATA[1]]></Rate>

<MessageType><![CDATA[Object]]></MessageType>

<DefaultTransition>true</DefaultTransition>

<FilterType><![CDATA[unconditionally]]></FilterType>

<EqualsExpression><![CDATA["text"]]></EqualsExpression>

<SatisfiesExpression><![CDATA[true]]></SatisfiesExpression>

</Properties>

</StatechartElement>

<StatechartElement Class="InitialStatePointer" ParentState="twoWeekTrip" ParentStateId="1412614421943">

<Id>1412615566475</Id>

<Name><![CDATA[initialState]]></Name>

<X>190</X><Y>50</Y>

<Label><X>10</X><Y>0</Y></Label>

<PublicFlag>false</PublicFlag>

<PresentationFlag>true</PresentationFlag>

<ShowLabel>false</ShowLabel>

<Points>

<Point><X>0</X><Y>0</Y></Point>

<Point><X>0</X><Y>30</Y></Point>

</Points>

<Properties Target="1412538171855">

</Properties>

</StatechartElement>

<StatechartElement Class="InitialStatePointer" ParentState="MercyShips" ParentStateId="1412614519305">

<Id>1412615574123</Id>

<Name><![CDATA[initialState1]]></Name>

<X>720</X><Y>120</Y>

<Label><X>10</X><Y>0</Y></Label>

<PublicFlag>false</PublicFlag>

<PresentationFlag>true</PresentationFlag>

<ShowLabel>false</ShowLabel>

<Points>

<Point><X>0</X><Y>0</Y></Point>

<Point><X>-50</X><Y>0</Y></Point>

</Points>

<Properties Target="1412614906536">

</Properties>

</StatechartElement>

<StatechartElement Class="InitialStatePointer" ParentState="Present" ParentStateId="1412614906536">

<Id>1412615595267</Id>

<Name><![CDATA[initialState2]]></Name>

<X>590</X><Y>260</Y>

<Label><X>10</X><Y>0</Y></Label>

<PublicFlag>false</PublicFlag>

<PresentationFlag>true</PresentationFlag>

<ShowLabel>false</ShowLabel>

<Points>

<Point><X>0</X><Y>0</Y></Point>

<Point><X>0</X><Y>-20</Y></Point>

</Points>

<Properties Target="1412615230678">

</Properties>

</StatechartElement>

<StatechartElement Class="Transition" ParentState="ROOT_NODE">

<Id>1413043326016</Id>

<Name><![CDATA[transition8]]></Name>

<X>300</X><Y>70</Y>

<Label><X>10</X><Y>0</Y></Label>

<PublicFlag>false</PublicFlag>

<PresentationFlag>true</PresentationFlag>

<ShowLabel>false</ShowLabel>

<Points>

<Point><X>0</X><Y>0</Y></Point>

<Point><X>0</X><Y>280</Y></Point>

<Point><X>-50</X><Y>280</Y></Point>

</Points>

<Properties Source="1412614594690" Target="1413043285677" Trigger="timeout">

<Timeout><![CDATA[1]]></Timeout>

<Condition><![CDATA[true]]></Condition>

<Rate><![CDATA[1]]></Rate>

<MessageType><![CDATA[Object]]></MessageType>

<DefaultTransition>true</DefaultTransition>

<FilterType><![CDATA[unconditionally]]></FilterType>

<EqualsExpression><![CDATA["text"]]></EqualsExpression>

<SatisfiesExpression><![CDATA[true]]></SatisfiesExpression>

</Properties>

</StatechartElement>

</StatechartElements>

<AgentLinks>

<AgentLink>

<Id>1392508625679</Id>

<Name><![CDATA[connections]]></Name>

<X>50</X><Y>-50</Y>

<Label><X>15</X><Y>0</Y></Label>

<PublicFlag>false</PublicFlag>

<PresentationFlag>true</PresentationFlag>

<ShowLabel>true</ShowLabel>

<HandleReceiveInConnections>false</HandleReceiveInConnections>

<AgentLinkType>COLLECTION_OF_LINKS</AgentLinkType>

<AgentLinkBidirectional>true</AgentLinkBidirectional>

<MessageType><![CDATA[Object]]></MessageType>

<StatechartNames>

<Name>statechart</Name>

<Name>statechart</Name>

</StatechartNames>

<LineStyle>SOLID</LineStyle>

<LineWidth>1</LineWidth>

<LineColor>-16777216</LineColor>

<LineZOrder>UNDER_AGENTS</LineZOrder>

<LineArrow>NONE</LineArrow>

<LineArrowPosition>END</LineArrowPosition>

</AgentLink>

</AgentLinks>

<ContainerLinks>

<ContainerLink>

<Id>1392508627985</Id>

<Name><![CDATA[main]]></Name>

<X>50</X><Y>-100</Y>

<Label><X>10</X><Y>0</Y></Label>

<PublicFlag>false</PublicFlag>

<PresentationFlag>true</PresentationFlag>

<ShowLabel>true</ShowLabel>

<ActiveObjectClass>

<PackageName><![CDATA[_14_01_21_trial_uganda_model]]></PackageName>

<ClassName><![CDATA[Main]]></ClassName>

</ActiveObjectClass>

</ContainerLink>

</ContainerLinks>

<Shapes>

<Rectangle>

<Id>1390441248430</Id>

<Name><![CDATA[rectangle]]></Name>

<X>-5</X><Y>-5</Y>

<Label><X>10</X><Y>10</Y></Label>

<PublicFlag>true</PublicFlag>

<PresentationFlag>true</PresentationFlag>

<ShowLabel>false</ShowLabel>

<DrawMode>SHAPE_DRAW_2D</DrawMode>

<AsObject>true</AsObject>

<EmbeddedIcon>false</EmbeddedIcon>

<Z>0</Z>

<ZHeight>10</ZHeight>

<LineWidth>1</LineWidth>

<LineColor/>

<LineMaterial>null</LineMaterial>

<LineStyle>SOLID</LineStyle>

<Width>10</Width>

<Height>10</Height>

<Rotation>0.0</Rotation>

<FillColor>-16776961</FillColor>

<FillMaterial>null</FillMaterial>

</Rectangle>

<Text>

<Id>1390444968578</Id>

<Name><![CDATA[textName]]></Name>

<X>-10</X><Y>-10</Y>

<Label><X>0</X><Y>-10</Y></Label>

<PublicFlag>true</PublicFlag>

<PresentationFlag>true</PresentationFlag>

<ShowLabel>false</ShowLabel>

<DrawMode>SHAPE_DRAW_2D</DrawMode>

<VisibleCode><![CDATA[get_Main().displayNatNames]]></VisibleCode>

<AsObject>true</AsObject>

<EmbeddedIcon>false</EmbeddedIcon>

<Z>0</Z>

<Rotation>0.0</Rotation>

<RotationCode><![CDATA[-45]]></RotationCode>

<Color>-16777216</Color>

<Text><![CDATA[text]]></Text>

<TextCode><![CDATA[name]]></TextCode>

<Font>

<Name>SansSerif</Name>

<Size>14</Size>

<Style>0</Style>

</Font>

<Alignment>LEFT</Alignment>

</Text>

</Shapes>

</ActiveObjectClass>

<!-- ========= Active Object Class ======== -->

<ActiveObjectClass>

<Id>1390766198904</Id>

<Name><![CDATA[Regional]]></Name>

<ClientAreaTopLeft><X>0</X><Y>0</Y></ClientAreaTopLeft>

<PresentationTopGroupPersistent>true</PresentationTopGroupPersistent>

<IconTopGroupPersistent>true</IconTopGroupPersistent>

<StartupCode><![CDATA[jumpTo(longitude,latitude);]]></StartupCode>

<Generic>false</Generic>

<GenericParameters><![CDATA[T]]></GenericParameters>

<GenericParametersLabel><![CDATA[Generic parameters:]]></GenericParametersLabel>

<FlowChartsUsage>ENTITY</FlowChartsUsage>

<SamplesToKeep>100</SamplesToKeep>

<LimitNumberOfArrayElements>false</LimitNumberOfArrayElements>

<ElementsLimitValue>100</ElementsLimitValue>

<MakeDefaultViewArea>true</MakeDefaultViewArea>

<SceneGridColor/>

<SceneBackgroundColor/>

<AgentProperties>

<SpaceType>GIS</SpaceType>

<EnvironmentDefinesInitialLocation>true</EnvironmentDefinesInitialLocation>

<RotateAnimationTowardsMovement>true</RotateAnimationTowardsMovement>

<RotateAnimationVertically>false</RotateAnimationVertically>

<VelocityCode><![CDATA[10]]></VelocityCode>

<VelocityGISCode><![CDATA[10]]></VelocityGISCode>

</AgentProperties>

<EnvironmentProperties>

<EnableSteps>false</EnableSteps>

<StepDurationCode><![CDATA[1.0]]></StepDurationCode>

<SpaceType>CONTINUOUS</SpaceType>

<WidthCode><![CDATA[500]]></WidthCode>

<HeightCode><![CDATA[500]]></HeightCode>

<ZHeightCode><![CDATA[0]]></ZHeightCode>

<ColumnsCountCode><![CDATA[100]]></ColumnsCountCode>

<RowsCountCode><![CDATA[100]]></RowsCountCode>

<NeigborhoodType>MOORE</NeigborhoodType>

<LayoutType>USER_DEF</LayoutType>

<LayoutTypeApplyOnStartup>true</LayoutTypeApplyOnStartup>

<NetworkType>USER_DEF</NetworkType>

<NetworkTypeApplyOnStartup>true</NetworkTypeApplyOnStartup>

<ConnectionsPerAgentCode><![CDATA[2]]></ConnectionsPerAgentCode>

<ConnectionsRangeCode><![CDATA[50]]></ConnectionsRangeCode>

<NeighborLinkFractionCode><![CDATA[0.95]]></NeighborLinkFractionCode>

<MCode><![CDATA[10]]></MCode>

</EnvironmentProperties>

<DatasetsCreationProperties>

<AutoCreate>true</AutoCreate>

<RecurrenceCode><![CDATA[1]]></RecurrenceCode>

</DatasetsCreationProperties>

<ConnectionsId>1392508625680</ConnectionsId>

<Variables>

<Variable Class="PlainVariable">

<Id>1394323624227</Id>

<Name><![CDATA[agentValue]]></Name>

<X>250</X><Y>20</Y>

<Label><X>10</X><Y>0</Y></Label>

<PublicFlag>false</PublicFlag>

<PresentationFlag>true</PresentationFlag>

<ShowLabel>true</ShowLabel>

<Properties SaveInSnapshot="true" Constant="false" AccessType="public" StaticVariable="false">

<Type><![CDATA[double]]></Type>

</Properties>

</Variable>

<Variable Class="Parameter">

<Id>1390766393587</Id>

<Name><![CDATA[name]]></Name>

<X>50</X><Y>20</Y>

<Label><X>10</X><Y>0</Y></Label>

<PublicFlag>false</PublicFlag>

<PresentationFlag>true</PresentationFlag>

<ShowLabel>true</ShowLabel>

<Properties SaveInSnapshot="true" ModificatorType="STATIC">

<Type><![CDATA[String]]></Type>

<SdArray>false</SdArray>

<DefaultValue><![CDATA[]]></DefaultValue>

<ParameterEditor>

<Id>1390766393585</Id>

<Name><![CDATA[]]></Name>

<EditorContolType>TEXT_BOX</EditorContolType>

<MinSliderValue><![CDATA[0]]></MinSliderValue>

<MaxSliderValue><![CDATA[100]]></MaxSliderValue>

<DelimeterType>NO_DELIMETER</DelimeterType>

</ParameterEditor>

</Properties>

</Variable>

<Variable Class="Parameter">

<Id>1390766393590</Id>

<Name><![CDATA[priv]]></Name>

<Description><![CDATA[Is the hospital a private hospital?]]></Description>

<X>50</X><Y>70</Y>

<Label><X>10</X><Y>0</Y></Label>

<PublicFlag>false</PublicFlag>

<PresentationFlag>true</PresentationFlag>

<ShowLabel>true</ShowLabel>

<Properties SaveInSnapshot="true" ModificatorType="STATIC">

<Type><![CDATA[int]]></Type>

<SdArray>false</SdArray>

<DefaultValue><![CDATA[]]></DefaultValue>

<ParameterEditor>

<Id>1390766393588</Id>

<Name><![CDATA[]]></Name>

<EditorContolType>TEXT_BOX</EditorContolType>

<MinSliderValue><![CDATA[0]]></MinSliderValue>

<MaxSliderValue><![CDATA[100]]></MaxSliderValue>

<DelimeterType>NO_DELIMETER</DelimeterType>

</ParameterEditor>

</Properties>

</Variable>

<Variable Class="Parameter">

<Id>1390766393593</Id>

<Name><![CDATA[latitude]]></Name>

<X>50</X><Y>120</Y>

<Label><X>10</X><Y>0</Y></Label>

<PublicFlag>false</PublicFlag>

<PresentationFlag>true</PresentationFlag>

<ShowLabel>true</ShowLabel>

<Properties SaveInSnapshot="true" ModificatorType="STATIC">

<Type><![CDATA[double]]></Type>

<SdArray>false</SdArray>

<DefaultValue><![CDATA[]]></DefaultValue>

<ParameterEditor>

<Id>1390766393591</Id>

<Name><![CDATA[]]></Name>

<EditorContolType>TEXT_BOX</EditorContolType>

<MinSliderValue><![CDATA[0]]></MinSliderValue>

<MaxSliderValue><![CDATA[100]]></MaxSliderValue>

<DelimeterType>NO_DELIMETER</DelimeterType>

</ParameterEditor>

</Properties>

</Variable>

<Variable Class="Parameter">

<Id>1390766393596</Id>

<Name><![CDATA[longitude]]></Name>

<X>50</X><Y>170</Y>

<Label><X>10</X><Y>0</Y></Label>

<PublicFlag>false</PublicFlag>

<PresentationFlag>true</PresentationFlag>

<ShowLabel>true</ShowLabel>

<Properties SaveInSnapshot="true" ModificatorType="STATIC">

<Type><![CDATA[double]]></Type>

<SdArray>false</SdArray>

<DefaultValue><![CDATA[]]></DefaultValue>

<ParameterEditor>

<Id>1390766393594</Id>

<Name><![CDATA[]]></Name>

<EditorContolType>TEXT_BOX</EditorContolType>

<MinSliderValue><![CDATA[0]]></MinSliderValue>

<MaxSliderValue><![CDATA[100]]></MaxSliderValue>

<DelimeterType>NO_DELIMETER</DelimeterType>

</ParameterEditor>

</Properties>

</Variable>

<Variable Class="Parameter">

<Id>1410459510973</Id>

<Name><![CDATA[quality]]></Name>

<X>50</X><Y>210</Y>

<Label><X>10</X><Y>0</Y></Label>

<PublicFlag>false</PublicFlag>

<PresentationFlag>true</PresentationFlag>

<ShowLabel>true</ShowLabel>

<Properties SaveInSnapshot="true" ModificatorType="STATIC">

<Type><![CDATA[double]]></Type>

<SdArray>false</SdArray>

<DefaultValue><![CDATA[0]]></DefaultValue>

<ParameterEditor>

<Id>1410459510971</Id>

<Name><![CDATA[]]></Name>

<EditorContolType>TEXT_BOX</EditorContolType>

<MinSliderValue><![CDATA[0]]></MinSliderValue>

<MaxSliderValue><![CDATA[100]]></MaxSliderValue>

<DelimeterType>NO_DELIMETER</DelimeterType>

</ParameterEditor>

</Properties>

</Variable>

<Variable Class="Parameter">

<Id>1410472613470</Id>

<Name><![CDATA[NGO]]></Name>

<X>50</X><Y>250</Y>

<Label><X>10</X><Y>0</Y></Label>

<PublicFlag>false</PublicFlag>

<PresentationFlag>true</PresentationFlag>

<ShowLabel>true</ShowLabel>

<Properties SaveInSnapshot="true" ModificatorType="STATIC">

<Type><![CDATA[boolean]]></Type>

<SdArray>false</SdArray>

<DefaultValue><![CDATA[false]]></DefaultValue>

<ParameterEditor>

<Id>1410472613468</Id>

<Name><![CDATA[]]></Name>

<EditorContolType>CHECK_BOX</EditorContolType>

<MinSliderValue><![CDATA[0]]></MinSliderValue>

<MaxSliderValue><![CDATA[100]]></MaxSliderValue>

<DelimeterType>NO_DELIMETER</DelimeterType>

</ParameterEditor>

</Properties>

</Variable>

<Variable Class="Parameter">

<Id>1410472613473</Id>

<Name><![CDATA[cost]]></Name>

<X>50</X><Y>290</Y>

<Label><X>10</X><Y>0</Y></Label>

<PublicFlag>false</PublicFlag>

<PresentationFlag>true</PresentationFlag>

<ShowLabel>true</ShowLabel>

<Properties SaveInSnapshot="true" ModificatorType="STATIC">

<Type><![CDATA[double]]></Type>

<SdArray>false</SdArray>

<DefaultValue><![CDATA[NGO ? 0 : get_Main().cSurg]]></DefaultValue>

<ParameterEditor>

<Id>1410472613471</Id>

<Name><![CDATA[]]></Name>

<EditorContolType>TEXT_BOX</EditorContolType>

<MinSliderValue><![CDATA[0]]></MinSliderValue>

<MaxSliderValue><![CDATA[100]]></MaxSliderValue>

<DelimeterType>NO_DELIMETER</DelimeterType>

</ParameterEditor>

</Properties>

</Variable>

<Variable Class="Parameter">

<Id>1410472613476</Id>

<Name><![CDATA[nonmed]]></Name>

<X>50</X><Y>330</Y>

<Label><X>10</X><Y>0</Y></Label>

<PublicFlag>false</PublicFlag>

<PresentationFlag>true</PresentationFlag>

<ShowLabel>true</ShowLabel>

<Properties SaveInSnapshot="true" ModificatorType="STATIC">

<Type><![CDATA[double]]></Type>

<SdArray>false</SdArray>

<DefaultValue><![CDATA[NGO ? 0 : get_Main().mNonMed * get_Main().cSurg]]></DefaultValue>

<ParameterEditor>

<Id>1410472613474</Id>

<Name><![CDATA[]]></Name>

<EditorContolType>TEXT_BOX</EditorContolType>

<MinSliderValue><![CDATA[0]]></MinSliderValue>

<MaxSliderValue><![CDATA[100]]></MaxSliderValue>

<DelimeterType>NO_DELIMETER</DelimeterType>

</ParameterEditor>

</Properties>

</Variable>

</Variables>

<AgentLinks>

<AgentLink>

<Id>1392508625680</Id>

<Name><![CDATA[connections]]></Name>

<X>50</X><Y>-50</Y>

<Label><X>15</X><Y>0</Y></Label>

<PublicFlag>false</PublicFlag>

<PresentationFlag>true</PresentationFlag>

<ShowLabel>true</ShowLabel>

<HandleReceiveInConnections>false</HandleReceiveInConnections>

<AgentLinkType>COLLECTION_OF_LINKS</AgentLinkType>

<AgentLinkBidirectional>true</AgentLinkBidirectional>

<MessageType><![CDATA[Object]]></MessageType>

<LineStyle>SOLID</LineStyle>

<LineWidth>1</LineWidth>

<LineColor>-16777216</LineColor>

<LineZOrder>UNDER_AGENTS</LineZOrder>

<LineArrow>NONE</LineArrow>

<LineArrowPosition>END</LineArrowPosition>

</AgentLink>

</AgentLinks>

<ContainerLinks>

<ContainerLink>

<Id>1392508627986</Id>

<Name><![CDATA[main]]></Name>

<X>50</X><Y>-100</Y>

<Label><X>10</X><Y>0</Y></Label>

<PublicFlag>false</PublicFlag>

<PresentationFlag>true</PresentationFlag>

<ShowLabel>true</ShowLabel>

<ActiveObjectClass>

<PackageName><![CDATA[_14_01_21_trial_uganda_model]]></PackageName>

<ClassName><![CDATA[Main]]></ClassName>

</ActiveObjectClass>

</ContainerLink>

</ContainerLinks>

<Shapes>

<Rectangle>

<Id>1390766198907</Id>

<Name><![CDATA[rectangle]]></Name>

<X>-3</X><Y>-3</Y>

<Label><X>10</X><Y>10</Y></Label>

<PublicFlag>true</PublicFlag>

<PresentationFlag>true</PresentationFlag>

<ShowLabel>false</ShowLabel>

<DrawMode>SHAPE_DRAW_2D</DrawMode>

<AsObject>true</AsObject>

<EmbeddedIcon>false</EmbeddedIcon>

<Z>0</Z>

<ZHeight>10</ZHeight>

<LineWidth>1</LineWidth>

<LineColor/>

<LineMaterial>null</LineMaterial>

<LineStyle>SOLID</LineStyle>

<Width>6</Width>

<Height>6</Height>

<Rotation>0.0</Rotation>

<FillColor>-8388608</FillColor>

<FillMaterial>null</FillMaterial>

</Rectangle>

<Text>

<Id>1390766642406</Id>

<Name><![CDATA[text]]></Name>

<X>-10</X><Y>-10</Y>

<Label><X>0</X><Y>-10</Y></Label>

<PublicFlag>true</PublicFlag>

<PresentationFlag>true</PresentationFlag>

<ShowLabel>false</ShowLabel>

<DrawMode>SHAPE_DRAW_2D</DrawMode>

<VisibleCode><![CDATA[get_Main().displayRegNames]]></VisibleCode>

<AsObject>true</AsObject>

<EmbeddedIcon>false</EmbeddedIcon>

<Z>0</Z>

<Rotation>0.0</Rotation>

<RotationCode><![CDATA[-45]]></RotationCode>

<Color>-16777216</Color>

<Text><![CDATA[text]]></Text>

<TextCode><![CDATA[name]]></TextCode>

<Font>

<Name>SansSerif</Name>

<Size>12</Size>

<Style>0</Style>

</Font>

<Alignment>LEFT</Alignment>

</Text>

</Shapes>

</ActiveObjectClass>

<!-- ========= Active Object Class ======== -->

<ActiveObjectClass>

<Id>1390768734137</Id>

<Name><![CDATA[District]]></Name>

<ClientAreaTopLeft><X>0</X><Y>0</Y></ClientAreaTopLeft>

<PresentationTopGroupPersistent>true</PresentationTopGroupPersistent>

<IconTopGroupPersistent>true</IconTopGroupPersistent>

<StartupCode><![CDATA[jumpTo(longitude, latitude);]]></StartupCode>

<Generic>false</Generic>

<GenericParameters><![CDATA[T]]></GenericParameters>

<GenericParametersLabel><![CDATA[Generic parameters:]]></GenericParametersLabel>

<FlowChartsUsage>ENTITY</FlowChartsUsage>

<SamplesToKeep>100</SamplesToKeep>

<LimitNumberOfArrayElements>false</LimitNumberOfArrayElements>

<ElementsLimitValue>100</ElementsLimitValue>

<MakeDefaultViewArea>true</MakeDefaultViewArea>

<SceneGridColor/>

<SceneBackgroundColor/>

<AgentProperties>

<SpaceType>GIS</SpaceType>

<EnvironmentDefinesInitialLocation>true</EnvironmentDefinesInitialLocation>

<RotateAnimationTowardsMovement>true</RotateAnimationTowardsMovement>

<RotateAnimationVertically>false</RotateAnimationVertically>

<VelocityCode><![CDATA[10]]></VelocityCode>

<VelocityGISCode><![CDATA[10]]></VelocityGISCode>

</AgentProperties>

<EnvironmentProperties>

<EnableSteps>false</EnableSteps>

<StepDurationCode><![CDATA[1.0]]></StepDurationCode>

<SpaceType>CONTINUOUS</SpaceType>

<WidthCode><![CDATA[500]]></WidthCode>

<HeightCode><![CDATA[500]]></HeightCode>

<ZHeightCode><![CDATA[0]]></ZHeightCode>

<ColumnsCountCode><![CDATA[100]]></ColumnsCountCode>

<RowsCountCode><![CDATA[100]]></RowsCountCode>

<NeigborhoodType>MOORE</NeigborhoodType>

<LayoutType>USER_DEF</LayoutType>

<LayoutTypeApplyOnStartup>true</LayoutTypeApplyOnStartup>

<NetworkType>USER_DEF</NetworkType>

<NetworkTypeApplyOnStartup>true</NetworkTypeApplyOnStartup>

<ConnectionsPerAgentCode><![CDATA[2]]></ConnectionsPerAgentCode>

<ConnectionsRangeCode><![CDATA[50]]></ConnectionsRangeCode>

<NeighborLinkFractionCode><![CDATA[0.95]]></NeighborLinkFractionCode>

<MCode><![CDATA[10]]></MCode>

</EnvironmentProperties>

<DatasetsCreationProperties>

<AutoCreate>true</AutoCreate>

<RecurrenceCode><![CDATA[1]]></RecurrenceCode>

</DatasetsCreationProperties>

<ConnectionsId>1392508625681</ConnectionsId>

<Variables>

<Variable Class="PlainVariable">

<Id>1394323618140</Id>

<Name><![CDATA[agentValue]]></Name>

<X>250</X><Y>20</Y>

<Label><X>10</X><Y>0</Y></Label>

<PublicFlag>false</PublicFlag>

<PresentationFlag>true</PresentationFlag>

<ShowLabel>true</ShowLabel>

<Properties SaveInSnapshot="true" Constant="false" AccessType="public" StaticVariable="false">

<Type><![CDATA[double]]></Type>

</Properties>

</Variable>

<Variable Class="Parameter">

<Id>1390768809336</Id>

<Name><![CDATA[name]]></Name>

<X>50</X><Y>20</Y>

<Label><X>10</X><Y>0</Y></Label>

<PublicFlag>false</PublicFlag>

<PresentationFlag>true</PresentationFlag>

<ShowLabel>true</ShowLabel>

<Properties SaveInSnapshot="true" ModificatorType="STATIC">

<Type><![CDATA[String]]></Type>

<SdArray>false</SdArray>

<DefaultValue><![CDATA[]]></DefaultValue>

<ParameterEditor>

<Id>1390768809334</Id>

<Name><![CDATA[]]></Name>

<EditorContolType>TEXT_BOX</EditorContolType>

<MinSliderValue><![CDATA[0]]></MinSliderValue>

<MaxSliderValue><![CDATA[100]]></MaxSliderValue>

<DelimeterType>NO_DELIMETER</DelimeterType>

</ParameterEditor>

</Properties>

</Variable>

<Variable Class="Parameter">

<Id>1390768809339</Id>

<Name><![CDATA[priv]]></Name>

<Description><![CDATA[Is the hospital a private hospital?]]></Description>

<X>50</X><Y>70</Y>

<Label><X>10</X><Y>0</Y></Label>

<PublicFlag>false</PublicFlag>

<PresentationFlag>true</PresentationFlag>

<ShowLabel>true</ShowLabel>

<Properties SaveInSnapshot="true" ModificatorType="STATIC">

<Type><![CDATA[int]]></Type>

<SdArray>false</SdArray>

<DefaultValue><![CDATA[]]></DefaultValue>

<ParameterEditor>

<Id>1390768809337</Id>

<Name><![CDATA[]]></Name>

<EditorContolType>TEXT_BOX</EditorContolType>

<MinSliderValue><![CDATA[0]]></MinSliderValue>

<MaxSliderValue><![CDATA[100]]></MaxSliderValue>

<DelimeterType>NO_DELIMETER</DelimeterType>

</ParameterEditor>

</Properties>

</Variable>

<Variable Class="Parameter">

<Id>1390768809342</Id>

<Name><![CDATA[latitude]]></Name>

<X>50</X><Y>120</Y>

<Label><X>10</X><Y>0</Y></Label>

<PublicFlag>false</PublicFlag>

<PresentationFlag>true</PresentationFlag>

<ShowLabel>true</ShowLabel>

<Properties SaveInSnapshot="true" ModificatorType="STATIC">

<Type><![CDATA[double]]></Type>

<SdArray>false</SdArray>

<DefaultValue><![CDATA[]]></DefaultValue>

<ParameterEditor>

<Id>1390768809340</Id>

<Name><![CDATA[]]></Name>

<EditorContolType>TEXT_BOX</EditorContolType>

<MinSliderValue><![CDATA[0]]></MinSliderValue>

<MaxSliderValue><![CDATA[100]]></MaxSliderValue>

<DelimeterType>NO_DELIMETER</DelimeterType>

</ParameterEditor>

</Properties>

</Variable>

<Variable Class="Parameter">

<Id>1390768809345</Id>

<Name><![CDATA[longitude]]></Name>

<X>50</X><Y>170</Y>

<Label><X>10</X><Y>0</Y></Label>

<PublicFlag>false</PublicFlag>

<PresentationFlag>true</PresentationFlag>

<ShowLabel>true</ShowLabel>

<Properties SaveInSnapshot="true" ModificatorType="STATIC">

<Type><![CDATA[double]]></Type>

<SdArray>false</SdArray>

<DefaultValue><![CDATA[]]></DefaultValue>

<ParameterEditor>

<Id>1390768809343</Id>

<Name><![CDATA[]]></Name>

<EditorContolType>TEXT_BOX</EditorContolType>

<MinSliderValue><![CDATA[0]]></MinSliderValue>

<MaxSliderValue><![CDATA[100]]></MaxSliderValue>

<DelimeterType>NO_DELIMETER</DelimeterType>

</ParameterEditor>

</Properties>

</Variable>

<Variable Class="Parameter">

<Id>1410459521447</Id>

<Name><![CDATA[quality]]></Name>

<X>50</X><Y>210</Y>

<Label><X>10</X><Y>0</Y></Label>

<PublicFlag>false</PublicFlag>

<PresentationFlag>true</PresentationFlag>

<ShowLabel>true</ShowLabel>

<Properties SaveInSnapshot="true" ModificatorType="STATIC">

<Type><![CDATA[double]]></Type>

<SdArray>false</SdArray>

<DefaultValue><![CDATA[0]]></DefaultValue>

<ParameterEditor>

<Id>1410459521445</Id>

<Name><![CDATA[]]></Name>

<EditorContolType>TEXT_BOX</EditorContolType>

<MinSliderValue><![CDATA[0]]></MinSliderValue>

<MaxSliderValue><![CDATA[100]]></MaxSliderValue>

<DelimeterType>NO_DELIMETER</DelimeterType>

</ParameterEditor>

</Properties>

</Variable>

<Variable Class="Parameter">

<Id>1410472622323</Id>

<Name><![CDATA[NGO]]></Name>

<X>50</X><Y>250</Y>

<Label><X>10</X><Y>0</Y></Label>

<PublicFlag>false</PublicFlag>

<PresentationFlag>true</PresentationFlag>

<ShowLabel>true</ShowLabel>

<Properties SaveInSnapshot="true" ModificatorType="STATIC">

<Type><![CDATA[boolean]]></Type>

<SdArray>false</SdArray>

<DefaultValue><![CDATA[false]]></DefaultValue>

<ParameterEditor>

<Id>1410472622321</Id>

<Name><![CDATA[]]></Name>

<EditorContolType>CHECK_BOX</EditorContolType>

<MinSliderValue><![CDATA[0]]></MinSliderValue>

<MaxSliderValue><![CDATA[100]]></MaxSliderValue>

<DelimeterType>NO_DELIMETER</DelimeterType>

</ParameterEditor>

</Properties>

</Variable>

<Variable Class="Parameter">

<Id>1410472622326</Id>

<Name><![CDATA[cost]]></Name>

<X>50</X><Y>290</Y>

<Label><X>10</X><Y>0</Y></Label>

<PublicFlag>false</PublicFlag>

<PresentationFlag>true</PresentationFlag>

<ShowLabel>true</ShowLabel>

<Properties SaveInSnapshot="true" ModificatorType="STATIC">

<Type><![CDATA[double]]></Type>

<SdArray>false</SdArray>

<DefaultValue><![CDATA[NGO ? 0 : get_Main().cSurg]]></DefaultValue>

<ParameterEditor>

<Id>1410472622324</Id>

<Name><![CDATA[]]></Name>

<EditorContolType>TEXT_BOX</EditorContolType>

<MinSliderValue><![CDATA[0]]></MinSliderValue>

<MaxSliderValue><![CDATA[100]]></MaxSliderValue>

<DelimeterType>NO_DELIMETER</DelimeterType>

</ParameterEditor>

</Properties>

</Variable>

<Variable Class="Parameter">

<Id>1410472622329</Id>

<Name><![CDATA[nonmed]]></Name>

<X>50</X><Y>330</Y>

<Label><X>10</X><Y>0</Y></Label>

<PublicFlag>false</PublicFlag>

<PresentationFlag>true</PresentationFlag>

<ShowLabel>true</ShowLabel>

<Properties SaveInSnapshot="true" ModificatorType="STATIC">

<Type><![CDATA[double]]></Type>

<SdArray>false</SdArray>

<DefaultValue><![CDATA[NGO ? 0 : get_Main().mNonMed * get_Main().cSurg]]></DefaultValue>

<ParameterEditor>

<Id>1410472622327</Id>

<Name><![CDATA[]]></Name>

<EditorContolType>TEXT_BOX</EditorContolType>

<MinSliderValue><![CDATA[0]]></MinSliderValue>

<MaxSliderValue><![CDATA[100]]></MaxSliderValue>

<DelimeterType>NO_DELIMETER</DelimeterType>

</ParameterEditor>

</Properties>

</Variable>

</Variables>

<AgentLinks>

<AgentLink>

<Id>1392508625681</Id>

<Name><![CDATA[connections]]></Name>

<X>50</X><Y>-50</Y>

<Label><X>15</X><Y>0</Y></Label>

<PublicFlag>false</PublicFlag>

<PresentationFlag>true</PresentationFlag>

<ShowLabel>true</ShowLabel>

<HandleReceiveInConnections>false</HandleReceiveInConnections>

<AgentLinkType>COLLECTION_OF_LINKS</AgentLinkType>

<AgentLinkBidirectional>true</AgentLinkBidirectional>

<MessageType><![CDATA[Object]]></MessageType>

<LineStyle>SOLID</LineStyle>

<LineWidth>1</LineWidth>

<LineColor>-16777216</LineColor>

<LineZOrder>UNDER_AGENTS</LineZOrder>

<LineArrow>NONE</LineArrow>

<LineArrowPosition>END</LineArrowPosition>

</AgentLink>

</AgentLinks>

<ContainerLinks>

<ContainerLink>

<Id>1392508627987</Id>

<Name><![CDATA[main]]></Name>

<X>50</X><Y>-100</Y>

<Label><X>10</X><Y>0</Y></Label>

<PublicFlag>false</PublicFlag>

<PresentationFlag>true</PresentationFlag>

<ShowLabel>true</ShowLabel>

<ActiveObjectClass>

<PackageName><![CDATA[_14_01_21_trial_uganda_model]]></PackageName>

<ClassName><![CDATA[Main]]></ClassName>

</ActiveObjectClass>

</ContainerLink>

</ContainerLinks>

<Shapes>

<Rectangle>

<Id>1390768734139</Id>

<Name><![CDATA[rectangle]]></Name>

<X>-2</X><Y>-2</Y>

<Label><X>10</X><Y>10</Y></Label>

<PublicFlag>true</PublicFlag>

<PresentationFlag>true</PresentationFlag>

<ShowLabel>false</ShowLabel>

<DrawMode>SHAPE_DRAW_2D</DrawMode>

<AsObject>true</AsObject>

<EmbeddedIcon>false</EmbeddedIcon>

<Z>0</Z>

<ZHeight>10</ZHeight>

<LineWidth>1</LineWidth>

<LineColor/>

<LineMaterial>null</LineMaterial>

<LineStyle>SOLID</LineStyle>

<Width>4</Width>

<Height>4</Height>

<Rotation>0.0</Rotation>

<FillColor>-16777216</FillColor>

<FillMaterial>null</FillMaterial>

</Rectangle>

<Text>

<Id>1390768792008</Id>

<Name><![CDATA[text]]></Name>

<X>-10</X><Y>-10</Y>

<Label><X>0</X><Y>-10</Y></Label>

<PublicFlag>true</PublicFlag>

<PresentationFlag>true</PresentationFlag>

<ShowLabel>false</ShowLabel>

<DrawMode>SHAPE_DRAW_2D</DrawMode>

<VisibleCode><![CDATA[get_Main().displayDistNames]]></VisibleCode>

<AsObject>true</AsObject>

<EmbeddedIcon>false</EmbeddedIcon>

<Z>0</Z>

<Rotation>0.0</Rotation>

<RotationCode><![CDATA[-45]]></RotationCode>

<Color>-16777216</Color>

<Text><![CDATA[text]]></Text>

<TextCode><![CDATA[name ]]></TextCode>

<Font>

<Name>SansSerif</Name>

<Size>9</Size>

<Style>0</Style>

</Font>

<Alignment>LEFT</Alignment>

</Text>

</Shapes>

</ActiveObjectClass>

<!-- ========= Active Object Class ======== -->

<ActiveObjectClass>

<Id>1390777039087</Id>

<Name><![CDATA[Person]]></Name>

<Description><![CDATA[STARTUP ACTIONS

1. Jump to agent's location

2. Calculate education level

]]></Description>

<ClientAreaTopLeft><X>0</X><Y>0</Y></ClientAreaTopLeft>

<PresentationTopGroupPersistent>true</PresentationTopGroupPersistent>

<IconTopGroupPersistent>true</IconTopGroupPersistent>

<Import><![CDATA[import com.sun.tools.corba.se.idl.constExpr.Modulo;

import java.text.DecimalFormat;]]></Import>

<StartupCode><![CDATA[jumpTo(longitude,latitude);

educPrimary = randomTrue(0.514) ? 1:0;

educSecondary = randomTrue(0.312) ? 1:0;

educSecondary = max(0, educSecondary - educPrimary);

]]></StartupCode>

<Generic>false</Generic>

<GenericParameters><![CDATA[T]]></GenericParameters>

<GenericParametersLabel><![CDATA[Generic parameters:]]></GenericParametersLabel>

<FlowChartsUsage>ENTITY</FlowChartsUsage>

<SamplesToKeep>100</SamplesToKeep>

<LimitNumberOfArrayElements>false</LimitNumberOfArrayElements>

<ElementsLimitValue>100</ElementsLimitValue>

<MakeDefaultViewArea>true</MakeDefaultViewArea>

<SceneGridColor/>

<SceneBackgroundColor/>

<AgentProperties>

<SpaceType>GIS</SpaceType>

<EnvironmentDefinesInitialLocation>true</EnvironmentDefinesInitialLocation>

<RotateAnimationTowardsMovement>false</RotateAnimationTowardsMovement>

<RotateAnimationVertically>false</RotateAnimationVertically>

<VelocityCode><![CDATA[randomTrue(0.8) ? 9.306 : 1.341]]></VelocityCode>

<VelocityGISCode><![CDATA[10]]></VelocityGISCode>

<StatechartNames>

<Name>statechart</Name>

</StatechartNames>

</AgentProperties>

<EnvironmentProperties>

<EnableSteps>false</EnableSteps>

<StepDurationCode><![CDATA[1.0]]></StepDurationCode>

<SpaceType>CONTINUOUS</SpaceType>

<WidthCode><![CDATA[500]]></WidthCode>

<HeightCode><![CDATA[500]]></HeightCode>

<ZHeightCode><![CDATA[0]]></ZHeightCode>

<ColumnsCountCode><![CDATA[100]]></ColumnsCountCode>

<RowsCountCode><![CDATA[100]]></RowsCountCode>

<NeigborhoodType>MOORE</NeigborhoodType>

<LayoutType>USER_DEF</LayoutType>

<LayoutTypeApplyOnStartup>true</LayoutTypeApplyOnStartup>

<NetworkType>USER_DEF</NetworkType>

<NetworkTypeApplyOnStartup>true</NetworkTypeApplyOnStartup>

<ConnectionsPerAgentCode><![CDATA[2]]></ConnectionsPerAgentCode>

<ConnectionsRangeCode><![CDATA[50]]></ConnectionsRangeCode>

<NeighborLinkFractionCode><![CDATA[0.95]]></NeighborLinkFractionCode>

<MCode><![CDATA[10]]></MCode>

</EnvironmentProperties>

<DatasetsCreationProperties>

<AutoCreate>true</AutoCreate>

<RecurrenceCode><![CDATA[1]]></RecurrenceCode>

</DatasetsCreationProperties>

<ConnectionsId>1392508625683</ConnectionsId>

<Variables>

<Variable Class="PlainVariable">

<Id>1391291059148</Id>

<Name><![CDATA[yearBorn]]></Name>

<X>-180</X><Y>30</Y>

<Label><X>10</X><Y>0</Y></Label>

<PublicFlag>false</PublicFlag>

<PresentationFlag>false</PresentationFlag>

<ShowLabel>true</ShowLabel>

<Properties SaveInSnapshot="true" Constant="false" AccessType="public" StaticVariable="false">

<Type><![CDATA[double]]></Type>

<InitialValue><![CDATA[getYear()]]></InitialValue>

</Properties>

</Variable>

<Variable Class="PlainVariable">

<Id>1392314678038</Id>

<Name><![CDATA[TFR]]></Name>

<Description><![CDATA[Counts the total number of children the woman has had.]]></Description>

<X>-180</X><Y>50</Y>

<Label><X>10</X><Y>0</Y></Label>

<PublicFlag>false</PublicFlag>

<PresentationFlag>false</PresentationFlag>

<ShowLabel>true</ShowLabel>

<Properties SaveInSnapshot="true" Constant="false" AccessType="public" StaticVariable="false">

<Type><![CDATA[int]]></Type>

<InitialValue><![CDATA[0]]></InitialValue>

</Properties>

</Variable>

<Variable Class="PlainVariable">

<Id>1392412185915</Id>

<Name><![CDATA[age]]></Name>

<X>-180</X><Y>70</Y>

<Label><X>10</X><Y>0</Y></Label>

<PublicFlag>false</PublicFlag>

<PresentationFlag>false</PresentationFlag>

<ShowLabel>true</ShowLabel>

<Properties SaveInSnapshot="true" Constant="false" AccessType="public" StaticVariable="false">

<Type><![CDATA[int]]></Type>

<InitialValue><![CDATA[ageFunc()]]></InitialValue>

</Properties>

</Variable>

<Variable Class="PlainVariable">

<Id>1392666845084</Id>

<Name><![CDATA[dayIDie]]></Name>

<X>-180</X><Y>90</Y>

<Label><X>10</X><Y>0</Y></Label>

<PublicFlag>false</PublicFlag>

<PresentationFlag>false</PresentationFlag>

<ShowLabel>true</ShowLabel>

<Properties SaveInSnapshot="true" Constant="false" AccessType="public" StaticVariable="false">

<Type><![CDATA[int]]></Type>

</Properties>

</Variable>

<Variable Class="PlainVariable">

<Id>1392828923519</Id>

<Name><![CDATA[haveKidThisYear]]></Name>

<X>-180</X><Y>110</Y>

<Label><X>10</X><Y>0</Y></Label>

<PublicFlag>false</PublicFlag>

<PresentationFlag>false</PresentationFlag>

<ShowLabel>true</ShowLabel>

<Properties SaveInSnapshot="true" Constant="false" AccessType="public" StaticVariable="false">

<Type><![CDATA[boolean]]></Type>

<InitialValue><![CDATA[false]]></InitialValue>

</Properties>

</Variable>

<Variable Class="PlainVariable">

<Id>1393272368777</Id>

<Name><![CDATA[wealth]]></Name>

<X>-180</X><Y>130</Y>

<Label><X>10</X><Y>0</Y></Label>

<PublicFlag>false</PublicFlag>

<PresentationFlag>false</PresentationFlag>

<ShowLabel>true</ShowLabel>

<Properties SaveInSnapshot="true" Constant="false" AccessType="public" StaticVariable="false">

<Type><![CDATA[double]]></Type>

</Properties>

</Variable>

<Variable Class="PlainVariable">

<Id>1394131417694</Id>

<Name><![CDATA[chosenHospital]]></Name>

<X>-180</X><Y>260</Y>

<Label><X>10</X><Y>0</Y></Label>

<PublicFlag>false</PublicFlag>

<PresentationFlag>false</PresentationFlag>

<ShowLabel>true</ShowLabel>

<Properties SaveInSnapshot="true" Constant="false" AccessType="public" StaticVariable="false">

<Type><![CDATA[Agent]]></Type>

<InitialValue><![CDATA[null]]></InitialValue>

</Properties>

</Variable>

<Variable Class="PlainVariable">

<Id>1410467119351</Id>

<Name><![CDATA[denom]]></Name>

<Description><![CDATA[Denominator for the conditional logits]]></Description>

<X>-180</X><Y>280</Y>

<Label><X>10</X><Y>0</Y></Label>

<PublicFlag>false</PublicFlag>

<PresentationFlag>false</PresentationFlag>

<ShowLabel>true</ShowLabel>

<Properties SaveInSnapshot="true" Constant="false" AccessType="public" StaticVariable="false">

<Type><![CDATA[double]]></Type>

<InitialValue><![CDATA[1]]></InitialValue>

</Properties>

</Variable>

<Variable Class="PlainVariable">

<Id>1410554413691</Id>

<Name><![CDATA[pDieTx]]></Name>

<X>-300</X><Y>340</Y>

<Label><X>10</X><Y>0</Y></Label>

<PublicFlag>false</PublicFlag>

<PresentationFlag>false</PresentationFlag>

<ShowLabel>true</ShowLabel>

<Properties SaveInSnapshot="true" Constant="false" AccessType="public" StaticVariable="false">

<Type><![CDATA[double]]></Type>

<InitialValue><![CDATA[get_Main().periopMortality]]></InitialValue>

</Properties>

</Variable>

<Variable Class="PlainVariable">

<Id>1410741929539</Id>

<Name><![CDATA[destLong]]></Name>

<X>-180</X><Y>340</Y>

<Label><X>10</X><Y>0</Y></Label>

<PublicFlag>false</PublicFlag>

<PresentationFlag>false</PresentationFlag>

<ShowLabel>true</ShowLabel>

<Properties SaveInSnapshot="true" Constant="false" AccessType="public" StaticVariable="false">

<Type><![CDATA[double]]></Type>

</Properties>

</Variable>

<Variable Class="PlainVariable">

<Id>1410741940597</Id>

<Name><![CDATA[destLat]]></Name>

<X>-180</X><Y>360</Y>

<Label><X>10</X><Y>0</Y></Label>

<PublicFlag>false</PublicFlag>

<PresentationFlag>false</PresentationFlag>

<ShowLabel>true</ShowLabel>

<Properties SaveInSnapshot="true" Constant="false" AccessType="public" StaticVariable="false">

<Type><![CDATA[double]]></Type>

</Properties>

</Variable>

<Variable Class="PlainVariable">

<Id>1411060007129</Id>

<Name><![CDATA[hospIndex]]></Name>

<X>-180</X><Y>240</Y>

<Label><X>10</X><Y>0</Y></Label>

<PublicFlag>false</PublicFlag>

<PresentationFlag>false</PresentationFlag>

<ShowLabel>true</ShowLabel>

<Properties SaveInSnapshot="true" Constant="false" AccessType="public" StaticVariable="false">

<Type><![CDATA[int]]></Type>

</Properties>

</Variable>

<Variable Class="PlainVariable">

<Id>1411062245138</Id>

<Name><![CDATA[systCost]]></Name>

<X>-180</X><Y>380</Y>

<Label><X>10</X><Y>0</Y></Label>

<PublicFlag>false</PublicFlag>

<PresentationFlag>false</PresentationFlag>

<ShowLabel>true</ShowLabel>

<Properties SaveInSnapshot="true" Constant="false" AccessType="public" StaticVariable="false">

<Type><![CDATA[double]]></Type>

</Properties>

</Variable>

<Variable Class="PlainVariable">

<Id>1411062251191</Id>

<Name><![CDATA[persCost]]></Name>

<X>-180</X><Y>400</Y>

<Label><X>10</X><Y>0</Y></Label>

<PublicFlag>false</PublicFlag>

<PresentationFlag>false</PresentationFlag>

<ShowLabel>true</ShowLabel>

<Properties SaveInSnapshot="true" Constant="false" AccessType="public" StaticVariable="false">

<Type><![CDATA[double]]></Type>

</Properties>

</Variable>

<Variable Class="PlainVariable">

<Id>1411486434638</Id>

<Name><![CDATA[careSought]]></Name>

<X>-180</X><Y>220</Y>

<Label><X>10</X><Y>0</Y></Label>

<PublicFlag>false</PublicFlag>

<PresentationFlag>false</PresentationFlag>

<ShowLabel>true</ShowLabel>

<Properties SaveInSnapshot="true" Constant="false" AccessType="public" StaticVariable="false">

<Type><![CDATA[boolean]]></Type>

</Properties>

</Variable>

<Variable Class="PlainVariable">

<Id>1411498811780</Id>

<Name><![CDATA[educPrimary]]></Name>

<X>-300</X><Y>270</Y>

<Label><X>10</X><Y>0</Y></Label>

<PublicFlag>false</PublicFlag>

<PresentationFlag>false</PresentationFlag>

<ShowLabel>true</ShowLabel>

<Properties SaveInSnapshot="true" Constant="false" AccessType="public" StaticVariable="false">

<Type><![CDATA[int]]></Type>

</Properties>

</Variable>

<Variable Class="PlainVariable">

<Id>1411498827592</Id>

<Name><![CDATA[educSecondary]]></Name>

<X>-300</X><Y>290</Y>

<Label><X>10</X><Y>0</Y></Label>

<PublicFlag>false</PublicFlag>

<PresentationFlag>false</PresentationFlag>

<ShowLabel>true</ShowLabel>

<Properties SaveInSnapshot="true" Constant="false" AccessType="public" StaticVariable="false">

<Type><![CDATA[int]]></Type>

</Properties>

</Variable>

<Variable Class="PlainVariable">

<Id>1411508810126</Id>

<Name><![CDATA[cancerDeathDate]]></Name>

<X>-300</X><Y>360</Y>

<Label><X>10</X><Y>0</Y></Label>

<PublicFlag>false</PublicFlag>

<PresentationFlag>false</PresentationFlag>

<ShowLabel>true</ShowLabel>

<Properties SaveInSnapshot="true" Constant="false" AccessType="public" StaticVariable="false">

<Type><![CDATA[int]]></Type>

</Properties>

</Variable>

<Variable Class="PlainVariable">

<Id>1411509095341</Id>

<Name><![CDATA[txEndDate]]></Name>

<X>-300</X><Y>380</Y>

<Label><X>10</X><Y>0</Y></Label>

<PublicFlag>false</PublicFlag>

<PresentationFlag>false</PresentationFlag>

<ShowLabel>true</ShowLabel>

<Properties SaveInSnapshot="true" Constant="false" AccessType="public" StaticVariable="false">

<Type><![CDATA[int]]></Type>

<InitialValue><![CDATA[14]]></InitialValue>

</Properties>

</Variable>

<Variable Class="PlainVariable">

<Id>1412785884621</Id>

<Name><![CDATA[compl]]></Name>

<X>-300</X><Y>400</Y>

<Label><X>10</X><Y>0</Y></Label>

<PublicFlag>false</PublicFlag>

<PresentationFlag>true</PresentationFlag>

<ShowLabel>true</ShowLabel>

<Properties SaveInSnapshot="true" Constant="false" AccessType="public" StaticVariable="false">

<Type><![CDATA[boolean]]></Type>

</Properties>

</Variable>

<Variable Class="PlainVariable">

<Id>1412786019507</Id>

<Name><![CDATA[dieCompl]]></Name>

<X>-300</X><Y>420</Y>

<Label><X>10</X><Y>0</Y></Label>

<PublicFlag>false</PublicFlag>

<PresentationFlag>true</PresentationFlag>

<ShowLabel>true</ShowLabel>

<Properties SaveInSnapshot="true" Constant="false" AccessType="public" StaticVariable="false">

<Type><![CDATA[boolean]]></Type>

</Properties>

</Variable>

<Variable Class="PlainVariable">

<Id>1425306653405</Id>

<Name><![CDATA[region]]></Name>

<X>-300</X><Y>310</Y>

<Label><X>10</X><Y>0</Y></Label>

<PublicFlag>false</PublicFlag>

<PresentationFlag>true</PresentationFlag>

<ShowLabel>true</ShowLabel>

<Properties SaveInSnapshot="true" Constant="false" AccessType="public" StaticVariable="false">

<Type><![CDATA[int]]></Type>

</Properties>

</Variable>

<Variable Class="Parameter">

<Id>1390777266365</Id>

<Name><![CDATA[latitude]]></Name>

<X>-300</X><Y>30</Y>

<Label><X>10</X><Y>0</Y></Label>

<PublicFlag>false</PublicFlag>

<PresentationFlag>false</PresentationFlag>

<ShowLabel>true</ShowLabel>

<Properties SaveInSnapshot="true" ModificatorType="STATIC">

<Type><![CDATA[double]]></Type>

<SdArray>false</SdArray>

<DefaultValue><![CDATA[]]></DefaultValue>

<ParameterEditor>

<Id>1390777266363</Id>

<Name><![CDATA[]]></Name>

<EditorContolType>TEXT_BOX</EditorContolType>

<MinSliderValue><![CDATA[0]]></MinSliderValue>

<MaxSliderValue><![CDATA[100]]></MaxSliderValue>

<DelimeterType>NO_DELIMETER</DelimeterType>

</ParameterEditor>

</Properties>

</Variable>

<Variable Class="Parameter">

<Id>1390777266368</Id>

<Name><![CDATA[longitude]]></Name>

<X>-300</X><Y>50</Y>

<Label><X>10</X><Y>0</Y></Label>

<PublicFlag>false</PublicFlag>

<PresentationFlag>false</PresentationFlag>

<ShowLabel>true</ShowLabel>

<Properties SaveInSnapshot="true" ModificatorType="STATIC">

<Type><![CDATA[double]]></Type>

<SdArray>false</SdArray>

<DefaultValue><![CDATA[]]></DefaultValue>

<ParameterEditor>

<Id>1390777266366</Id>

<Name><![CDATA[]]></Name>

<EditorContolType>TEXT_BOX</EditorContolType>

<MinSliderValue><![CDATA[0]]></MinSliderValue>

<MaxSliderValue><![CDATA[100]]></MaxSliderValue>

<DelimeterType>NO_DELIMETER</DelimeterType>

</ParameterEditor>

</Properties>

</Variable>

<Variable Class="Parameter">

<Id>1391285093212</Id>

<Name><![CDATA[male]]></Name>

<X>-300</X><Y>90</Y>

<Label><X>10</X><Y>0</Y></Label>

<PublicFlag>false</PublicFlag>

<PresentationFlag>false</PresentationFlag>

<ShowLabel>true</ShowLabel>

<Properties SaveInSnapshot="true" ModificatorType="STATIC">

<Type><![CDATA[boolean]]></Type>

<SdArray>false</SdArray>

<DefaultValue><![CDATA[]]></DefaultValue>

<ParameterEditor>

<Id>1391285093210</Id>

<Name><![CDATA[]]></Name>

<EditorContolType>CHECK_BOX</EditorContolType>

<MinSliderValue><![CDATA[0]]></MinSliderValue>

<MaxSliderValue><![CDATA[100]]></MaxSliderValue>

<DelimeterType>NO_DELIMETER</DelimeterType>

</ParameterEditor>

</Properties>

</Variable>

<Variable Class="Parameter">

<Id>1392243565902</Id>

<Name><![CDATA[startState]]></Name>

<X>-300</X><Y>70</Y>

<Label><X>10</X><Y>0</Y></Label>

<PublicFlag>false</PublicFlag>

<PresentationFlag>false</PresentationFlag>

<ShowLabel>true</ShowLabel>

<Properties SaveInSnapshot="true" ModificatorType="STATIC">

<Type><![CDATA[int]]></Type>

<SdArray>false</SdArray>

<DefaultValue><![CDATA[2]]></DefaultValue>

<ParameterEditor>

<Id>1392243565900</Id>

<Name><![CDATA[]]></Name>

<EditorContolType>TEXT_BOX</EditorContolType>

<MinSliderValue><![CDATA[0]]></MinSliderValue>

<MaxSliderValue><![CDATA[100]]></MaxSliderValue>

<DelimeterType>NO_DELIMETER</DelimeterType>

</ParameterEditor>

</Properties>

</Variable>

<Variable Class="Parameter">

<Id>1393270526705</Id>

<Name><![CDATA[urban]]></Name>

<Description><![CDATA[urban vs. rural, based on the GPS and population Excel file]]></Description>

<X>-300</X><Y>190</Y>

<Label><X>10</X><Y>0</Y></Label>

<PublicFlag>false</PublicFlag>

<PresentationFlag>false</PresentationFlag>

<ShowLabel>true</ShowLabel>

<Properties SaveInSnapshot="true" ModificatorType="STATIC">

<Type><![CDATA[boolean]]></Type>

<SdArray>false</SdArray>

<DefaultValue><![CDATA[false]]></DefaultValue>

<ParameterEditor>

<Id>1393270526703</Id>

<Name><![CDATA[]]></Name>

<EditorContolType>CHECK_BOX</EditorContolType>

<MinSliderValue><![CDATA[0]]></MinSliderValue>

<MaxSliderValue><![CDATA[100]]></MaxSliderValue>

<DelimeterType>NO_DELIMETER</DelimeterType>

</ParameterEditor>

</Properties>

</Variable>

<Variable Class="Parameter">

<Id>1393272847392</Id>

<Name><![CDATA[quintile]]></Name>

<X>-300</X><Y>210</Y>

<Label><X>10</X><Y>0</Y></Label>

<PublicFlag>false</PublicFlag>

<PresentationFlag>false</PresentationFlag>

<ShowLabel>true</ShowLabel>

<Properties SaveInSnapshot="true" ModificatorType="STATIC">

<Type><![CDATA[int]]></Type>

<SdArray>false</SdArray>

<DefaultValue><![CDATA[1]]></DefaultValue>

<ParameterEditor>

<Id>1393272847390</Id>

<Name><![CDATA[]]></Name>

<EditorContolType>TEXT_BOX</EditorContolType>

<MinSliderValue><![CDATA[0]]></MinSliderValue>

<MaxSliderValue><![CDATA[100]]></MaxSliderValue>

<DelimeterType>NO_DELIMETER</DelimeterType>

</ParameterEditor>

</Properties>

</Variable>

<Variable Class="Parameter">

<Id>1393272866311</Id>

<Name><![CDATA[poor]]></Name>

<X>-300</X><Y>230</Y>

<Label><X>10</X><Y>0</Y></Label>

<PublicFlag>false</PublicFlag>

<PresentationFlag>false</PresentationFlag>

<ShowLabel>true</ShowLabel>

<Properties SaveInSnapshot="true" ModificatorType="STATIC">

<Type><![CDATA[boolean]]></Type>

<SdArray>false</SdArray>

<DefaultValue><![CDATA[false]]></DefaultValue>

<ParameterEditor>

<Id>1393272866309</Id>

<Name><![CDATA[]]></Name>

<EditorContolType>CHECK_BOX</EditorContolType>

<MinSliderValue><![CDATA[0]]></MinSliderValue>

<MaxSliderValue><![CDATA[100]]></MaxSliderValue>

<DelimeterType>NO_DELIMETER</DelimeterType>

</ParameterEditor>

</Properties>

</Variable>

<Variable Class="Parameter">

<Id>1393273143936</Id>

<Name><![CDATA[HOH]]></Name>

<X>-300</X><Y>250</Y>

<Label><X>10</X><Y>0</Y></Label>

<PublicFlag>false</PublicFlag>

<PresentationFlag>false</PresentationFlag>

<ShowLabel>true</ShowLabel>

<Properties SaveInSnapshot="true" ModificatorType="STATIC">

<Type><![CDATA[boolean]]></Type>

<SdArray>false</SdArray>

<DefaultValue><![CDATA[false]]></DefaultValue>

<ParameterEditor>

<Id>1393273143934</Id>

<Name><![CDATA[]]></Name>

<EditorContolType>CHECK_BOX</EditorContolType>

<MinSliderValue><![CDATA[0]]></MinSliderValue>

<MaxSliderValue><![CDATA[100]]></MaxSliderValue>

<DelimeterType>NO_DELIMETER</DelimeterType>

</ParameterEditor>

</Properties>

</Variable>

<Variable Class="CollectionVariable">

<Id>1410726476333</Id>

<Name><![CDATA[provs]]></Name>

<X>-180</X><Y>200</Y>

<Label><X>10</X><Y>0</Y></Label>

<PublicFlag>false</PublicFlag>

<PresentationFlag>false</PresentationFlag>

<ShowLabel>true</ShowLabel>

<Properties SaveInSnapshot="true" AccessType="public" StaticVariable="false">

<CollectionClass><![CDATA[java.util.ArrayList]]></CollectionClass>

<ElementClass><![CDATA[Agent]]></ElementClass>

<ValueElementClass><![CDATA[String]]></ValueElementClass>

</Properties>

</Variable>

<Variable Class="CollectionVariable">

<Id>1413044917252</Id>

<Name><![CDATA[NGOs]]></Name>

<X>-180</X><Y>180</Y>

<Label><X>10</X><Y>0</Y></Label>

<PublicFlag>false</PublicFlag>

<PresentationFlag>true</PresentationFlag>

<ShowLabel>true</ShowLabel>

<Properties SaveInSnapshot="true" AccessType="public" StaticVariable="false">

<CollectionClass><![CDATA[java.util.ArrayList]]></CollectionClass>

<ElementClass><![CDATA[Agent]]></ElementClass>

<ValueElementClass><![CDATA[String]]></ValueElementClass>

</Properties>

</Variable>

</Variables>

<StatechartElements>

<StatechartElement Class="State" ParentState="ROOT_NODE">

<Id>1391299759829</Id>

<Name><![CDATA[Alive]]></Name>

<X>40</X><Y>120</Y>

<Label><X>10</X><Y>10</Y></Label>

<PublicFlag>false</PublicFlag>

<PresentationFlag>true</PresentationFlag>

<ShowLabel>true</ShowLabel>

<Properties Width="1210" Height="650">

<EntryAction><![CDATA[age = ageFunc();

]]></EntryAction>

<FillColor>-655366</FillColor>

</Properties>

</StatechartElement>

<StatechartElement Class="State" ParentState="Alive" ParentStateId="1391299759829">

<Id>1391299800636</Id>

<Name><![CDATA[Infant]]></Name>

<X>60</X><Y>150</Y>

<Label><X>10</X><Y>10</Y></Label>

<PublicFlag>false</PublicFlag>

<PresentationFlag>true</PresentationFlag>

<ShowLabel>true</ShowLabel>

<Properties Width="60" Height="30">

<EntryAction><![CDATA[age = ageFunc();

]]></EntryAction>

<FillColor>-5185306</FillColor>

</Properties>

</StatechartElement>

<StatechartElement Class="State" ParentState="Alive" ParentStateId="1391299759829">

<Id>1391299880932</Id>

<Name><![CDATA[Male]]></Name>

<X>80</X><Y>290</Y>

<Label><X>10</X><Y>10</Y></Label>

<PublicFlag>false</PublicFlag>

<PresentationFlag>true</PresentationFlag>

<ShowLabel>true</ShowLabel>

<Properties Width="570" Height="450">

<FillColor>-5913612</FillColor>

</Properties>

</StatechartElement>

<StatechartElement Class="State" ParentState="Male" ParentStateId="1391299880932">

<Id>1391286875465</Id>

<Name><![CDATA[SickM]]></Name>

<X>250</X><Y>320</Y>

<Label><X>10</X><Y>10</Y></Label>

<PublicFlag>false</PublicFlag>

<PresentationFlag>true</PresentationFlag>

<ShowLabel>true</ShowLabel>

<Properties Width="380" Height="110">

<FillColor>-7357297</FillColor>

</Properties>

</StatechartElement>

<StatechartElement Class="State" ParentState="Male" ParentStateId="1391299880932">

<Id>1391284865450</Id>

<Name><![CDATA[HealthyM]]></Name>

<Description><![CDATA[Healthy and Idle--the model assumes healthy people don't travel]]></Description>

<X>100</X><Y>320</Y>

<Label><X>10</X><Y>10</Y></Label>

<PublicFlag>false</PublicFlag>

<PresentationFlag>true</PresentationFlag>

<ShowLabel>true</ShowLabel>

<Properties Width="100" Height="30">

<FillColor>-2693905</FillColor>

</Properties>

</StatechartElement>

<StatechartElement Class="FinalState" ParentState="Male" ParentStateId="1391299880932">

<Id>1391299968072</Id>

<Name><![CDATA[CancerDeadM]]></Name>

<X>600</X><Y>530</Y>

<Label><X>-35</X><Y>15</Y></Label>

<PublicFlag>false</PublicFlag>

<PresentationFlag>true</PresentationFlag>

<ShowLabel>true</ShowLabel>

<Properties>

<Action><![CDATA[/** Uncomment if you want to count dropping below a familial poverty line

If you do, the if statement below (for spouse and kids) should read

if(this.HOH && (famWealth - this.wealth <= povLine)

//calculate familial income and familial poverty line

double povLine = get_Main().povertyLine * get_Main().famSize;

double famWealth = this.wealth;

if(spouse.isConnected()) famWealth += spouse.getConnectedAgent().wealth;

if(kids.size()>0) {

for(Person k : this.kids.getConnections()) famWealth += k.wealth;

}

if(famWealth <= povLine) this.poor = true;

else(this.poor) = false;

**/

//make spouse HOH, add spouse to people made poor, and remove linkage to spouse

if(spouse.isConnected()) {

//add spouse to impoverishment lists (only if I am the HOH)

if(this.HOH){

spouse.getConnectedAgent().poor = true;

spouse.getConnectedAgent().quintile = 1;

switch(this.quintile) {

case 1:

get_Main().madePoorPoorest++;

if(!this.poor) get_Main().madePoorNewPoorest++;

break;

case 2:

get_Main().madePoorPoor++;

if(!this.poor) get_Main().madePoorNewPoor++;

break;

case 3:

get_Main().madePoorMiddle++;

if(!this.poor) get_Main().madePoorNewMiddle++;

break;

case 4:

get_Main().madePoorRich++;

if(!this.poor) get_Main().madePoorNewRich++;

break;

case 5:

get_Main().madePoorRichest++;

if(!this.poor) get_Main().madePoorNewRichest++;

break;

default: error("No such quintile! (CancerDeath MadePoor)");

}

}

//make spouse the new HOH and disconnect

this.spouse.getConnectedAgent().HOH = true;

this.spouse.disconnect();

}

//remove linkage to father

if(father.isConnected()) father.disconnect();

//remove linkage to mother

if(mother.isConnected()) mother.disconnect();

//make your kids poor and remove linkages to them

if(kids.size() > 0) {

//if I am the HOH, add my kids to the impoverishment lists

if(this.HOH){

int kidnum = kids.size();

for(Person k : this.kids.getConnections()) {

k.poor = true;

k.quintile = 1;

}

switch(this.quintile) {

case 1:

get_Main().madePoorPoorest+= kidnum;

if(!this.poor) get_Main().madePoorNewPoorest += kidnum;

break;

case 2:

get_Main().madePoorPoor+= kidnum;

if(!this.poor) get_Main().madePoorNewPoor += kidnum;

break;

case 3:

get_Main().madePoorMiddle+= kidnum;

if(!this.poor) get_Main().madePoorNewMiddle += kidnum;

break;

case 4:

get_Main().madePoorRich+= kidnum;

if(!this.poor) get_Main().madePoorNewRich += kidnum;

break;

case 5:

get_Main().madePoorRichest+= kidnum;

if(!this.poor) get_Main().madePoorNewRichest += kidnum;

break;

default: error("No such quintile! (CancerDeathKids MadePoor)");

}

}

//disconnect kids

if(male) for(Person k : kids.getConnections()) k.father.disconnect();

else for(Person k : kids.getConnections()) k.mother.disconnect();

kids.disconnectFromAll();

}

//remove linkages to fellow villagers

connections.disconnectFromAll();

//Add to the cancer deaths statistics

switch(this.quintile){

case 1:

get_Main().cancerDeathPoorest++;

break;

case 2:

get_Main().cancerDeathPoor++;

break;

case 3:

get_Main().cancerDeathMiddle++;

break;

case 4:

get_Main().cancerDeathRich++;

break;

case 5:

get_Main().cancerDeathRichest++;

break;

default: error("No such quintile!");

}

//and kill me

get_Main().DeadThisYear++;

get_Main().remove_persons(this);

]]></Action>

</Properties>

</StatechartElement>

<StatechartElement Class="State" ParentState="Alive" ParentStateId="1391299759829">

<Id>1391300108314</Id>

<Name><![CDATA[Under5]]></Name>

<X>230</X><Y>150</Y>

<Label><X>10</X><Y>10</Y></Label>

<PublicFlag>false</PublicFlag>

<PresentationFlag>true</PresentationFlag>

<ShowLabel>true</ShowLabel>

<Properties Width="100" Height="30">

<FillColor>-4144960</FillColor>

</Properties>

</StatechartElement>

<StatechartElement Class="Branch" ParentState="Alive" ParentStateId="1391299759829">

<Id>1391300283103</Id>

<Name><![CDATA[startWithCa]]></Name>

<X>230</X><Y>250</Y>

<Label><X>10</X><Y>0</Y></Label>

<PublicFlag>false</PublicFlag>

<PresentationFlag>true</PresentationFlag>

<ShowLabel>false</ShowLabel>

<Properties>

</Properties>

</StatechartElement>

<StatechartElement Class="Branch" ParentState="ROOT_NODE">

<Id>1391300849644</Id>

<Name><![CDATA[branch]]></Name>

<X>280</X><Y>40</Y>

<Label><X>10</X><Y>0</Y></Label>

<PublicFlag>false</PublicFlag>

<PresentationFlag>true</PresentationFlag>

<ShowLabel>false</ShowLabel>

<Properties>

</Properties>

</StatechartElement>

<StatechartElement Class="Branch" ParentState="ROOT_NODE">

<Id>1391300938409</Id>

<Name><![CDATA[modelInitiation]]></Name>

<X>400</X><Y>40</Y>

<Label><X>-40</X><Y>-15</Y></Label>

<PublicFlag>false</PublicFlag>

<PresentationFlag>true</PresentationFlag>

<ShowLabel>false</ShowLabel>

<Properties>

</Properties>

</StatechartElement>

<StatechartElement Class="State" ParentState="Alive" ParentStateId="1391299759829">

<Id>1391567760449</Id>

<Name><![CDATA[Female]]></Name>

<X>660</X><Y>290</Y>

<Label><X>10</X><Y>10</Y></Label>

<PublicFlag>false</PublicFlag>

<PresentationFlag>true</PresentationFlag>

<ShowLabel>true</ShowLabel>

<Properties Width="550" Height="450">

<FillColor>-2572328</FillColor>

</Properties>

</StatechartElement>

<StatechartElement Class="State" ParentState="Female" ParentStateId="1391567760449">

<Id>1391567760452</Id>

<Name><![CDATA[SickF]]></Name>

<X>840</X><Y>320</Y>

<Label><X>10</X><Y>10</Y></Label>

<PublicFlag>false</PublicFlag>

<PresentationFlag>true</PresentationFlag>

<ShowLabel>true</ShowLabel>

<Properties Width="340" Height="100">

<FillColor>-7357297</FillColor>

</Properties>

</StatechartElement>

<StatechartElement Class="State" ParentState="Female" ParentStateId="1391567760449">

<Id>1391567760454</Id>

<Name><![CDATA[HealthyF]]></Name>

<Description><![CDATA[Healthy and Idle--the model assumes healthy people don't travel]]></Description>

<X>690</X><Y>320</Y>

<Label><X>10</X><Y>10</Y></Label>

<PublicFlag>false</PublicFlag>

<PresentationFlag>true</PresentationFlag>

<ShowLabel>true</ShowLabel>

<Properties Width="100" Height="80">

<FillColor>-3851</FillColor>

</Properties>

</StatechartElement>

<StatechartElement Class="FinalState" ParentState="Female" ParentStateId="1391567760449">

<Id>1391567760456</Id>

<Name><![CDATA[cancerDeadF]]></Name>

<X>1160</X><Y>490</Y>

<Label><X>-35</X><Y>15</Y></Label>

<PublicFlag>false</PublicFlag>

<PresentationFlag>true</PresentationFlag>

<ShowLabel>true</ShowLabel>

<Properties>

<Action><![CDATA[/** Uncomment if you want to count dropping below a familial poverty line

If you do, the if statement below (for spouse and kids) should read

if(this.HOH && (famWealth - this.wealth <= povLine)

//calculate familial income and familial poverty line

double povLine = get_Main().povertyLine * get_Main().famSize; //average household size = 5

double famWealth = this.wealth;

if(spouse.isConnected()) famWealth += spouse.getConnectedAgent().wealth;

if(kids.size()>0) {

for(Person k : this.kids.getConnections()) famWealth += k.wealth;

}

if(famWealth <= povLine) this.poor = true;

else(this.poor) = false;

**/

//make spouse HOH, add spouse to people made poor, and remove linkage to spouse

if(spouse.isConnected()) {

//add spouse to impoverishment lists (only if I am the HOH)

if(this.HOH){

spouse.getConnectedAgent().poor = true;

spouse.getConnectedAgent().quintile = 1;

switch(this.quintile) {

case 1:

get_Main().madePoorPoorest++;

if(!this.poor) get_Main().madePoorNewPoorest++;

break;

case 2:

get_Main().madePoorPoor++;

if(!this.poor) get_Main().madePoorNewPoor++;

break;

case 3:

get_Main().madePoorMiddle++;

if(!this.poor) get_Main().madePoorNewMiddle++;

break;

case 4:

get_Main().madePoorRich++;

if(!this.poor) get_Main().madePoorNewRich++;

break;

case 5:

get_Main().madePoorRichest++;

if(!this.poor) get_Main().madePoorNewRichest++;

break;

default: error("No such quintile! (CancerDeath MadePoor)");

}

}

//make spouse the new HOH and disconnect

this.spouse.getConnectedAgent().HOH = true;

this.spouse.disconnect();

}

//remove linkage to father

if(father.isConnected()) father.disconnect();

//remove linkage to mother

if(mother.isConnected()) mother.disconnect();

//make your kids poor and remove linkages to them

if(kids.size() > 0) {

//if I am the HOH, add my kids to the impoverishment lists

if(this.HOH){

int kidnum = kids.size();

for(Person k : this.kids.getConnections()) {

k.poor = true;

k.quintile = 1;

}

switch(this.quintile) {

case 1:

get_Main().madePoorPoorest+= kidnum;

if(!this.poor) get_Main().madePoorNewPoorest += kidnum;

break;

case 2:

get_Main().madePoorPoor+= kidnum;

if(!this.poor) get_Main().madePoorNewPoor += kidnum;

break;

case 3:

get_Main().madePoorMiddle+= kidnum;

if(!this.poor) get_Main().madePoorNewMiddle += kidnum;

break;

case 4:

get_Main().madePoorRich+= kidnum;

if(!this.poor) get_Main().madePoorNewRich += kidnum;

break;

case 5:

get_Main().madePoorRichest+= kidnum;

if(!this.poor) get_Main().madePoorNewRichest += kidnum;

break;

default: error("No such quintile! (CancerDeathKids MadePoor)");

}

}

//disconnect kids

if(male) for(Person k : kids.getConnections()) k.father.disconnect();

else for(Person k : kids.getConnections()) k.mother.disconnect();

kids.disconnectFromAll();

}

//remove linkages to fellow villagers

connections.disconnectFromAll();

//Add to the cancer deaths statistics

switch(this.quintile){

case 1:

get_Main().cancerDeathPoorest++;

break;

case 2:

get_Main().cancerDeathPoor++;

break;

case 3:

get_Main().cancerDeathMiddle++;

break;

case 4:

get_Main().cancerDeathRich++;

break;

case 5:

get_Main().cancerDeathRichest++;

break;

default: error("No such quintile!");

}

//and kill me

get_Main().DeadThisYear++;

get_Main().remove_persons(this);

]]></Action>

</Properties>

</StatechartElement>

<StatechartElement Class="FinalState" ParentState="ROOT_NODE">

<Id>1391287000817</Id>

<Name><![CDATA[Dead]]></Name>

<X>1300</X><Y>350</Y>

<Label><X>10</X><Y>0</Y></Label>

<PublicFlag>false</PublicFlag>

<PresentationFlag>true</PresentationFlag>

<ShowLabel>true</ShowLabel>

<Properties>

<Action><![CDATA[//remove linkage to spouse

if(spouse.isConnected()) {

this.spouse.getConnectedAgent().HOH = true;

spouse.disconnect();

}

//remove linkage to father

if(father.isConnected()) father.disconnect();

//remove linkage to mother

if(mother.isConnected()) mother.disconnect();

//remove linkages to your kids

if(kids.getConnectionsNumber() > 0) {

if(male) {

for(Person k : kids.getConnections()) k.father.disconnect();

} else {

for(Person k : kids.getConnections()) k.mother.disconnect();

}

kids.disconnectFromAll();

}

//remove linkages to fellow villagers

connections.disconnectFromAll();

//and kill me

get_Main().remove_persons(this);

]]></Action>

</Properties>

</StatechartElement>

<StatechartElement Class="Branch" ParentState="Alive" ParentStateId="1391299759829">

<Id>1391567996847</Id>

<Name><![CDATA[startWithCa1]]></Name>

<X>830</X><Y>250</Y>

<Label><X>10</X><Y>0</Y></Label>

<PublicFlag>false</PublicFlag>

<PresentationFlag>true</PresentationFlag>

<ShowLabel>false</ShowLabel>

<Properties>

</Properties>

</StatechartElement>

<StatechartElement Class="Branch" ParentState="Alive" ParentStateId="1391299759829">

<Id>1391568138614</Id>

<Name><![CDATA[malefemale1]]></Name>

<X>520</X><Y>220</Y>

<Label><X>10</X><Y>0</Y></Label>

<PublicFlag>false</PublicFlag>

<PresentationFlag>true</PresentationFlag>

<ShowLabel>false</ShowLabel>

<Properties>

</Properties>

</StatechartElement>

<StatechartElement Class="State" ParentState="Alive" ParentStateId="1391299759829">

<Id>1392245582239</Id>

<Name><![CDATA[Youth]]></Name>

<X>400</X><Y>150</Y>

<Label><X>10</X><Y>10</Y></Label>

<PublicFlag>false</PublicFlag>

<PresentationFlag>true</PresentationFlag>

<ShowLabel>true</ShowLabel>

<Properties Width="100" Height="30">

<FillColor>-4684277</FillColor>

</Properties>

</StatechartElement>

<StatechartElement Class="Branch" ParentState="Male" ParentStateId="1391299880932">

<Id>1392854325945</Id>

<Name><![CDATA[migrateM]]></Name>

<X>150</X><Y>380</Y>

<Label><X>10</X><Y>0</Y></Label>

<PublicFlag>false</PublicFlag>

<PresentationFlag>true</PresentationFlag>

<ShowLabel>true</ShowLabel>

<Properties>

</Properties>

</StatechartElement>

<StatechartElement Class="State" ParentState="Male" ParentStateId="1391299880932">

<Id>1392854410803</Id>

<Name><![CDATA[MigratingM]]></Name>

<X>100</X><Y>410</Y>

<Label><X>10</X><Y>10</Y></Label>

<PublicFlag>false</PublicFlag>

<PresentationFlag>true</PresentationFlag>

<ShowLabel>true</ShowLabel>

<Properties Width="100" Height="30">

<EntryAction><![CDATA[

// 1/1000 emigrate from Uganda per year. 5.2 percent migrate somewhere in Uganda. So, 5.3% will enter this state

// of which (0.001/0.053 = 0.018867925) will emigrate

double s = uniform(0,1);

if(s > 0.018867925) { //move to somewhere else in Uganda

//When you move whole families, this has to change

this.connections.disconnectFromAll();

//end family ties

int rownum = 2;

int pick = 0;

double dispersion = get_Main().placementRadius/1.15/60/2;

double r = uniform(0,1);

if (r < 0.303941182) pick = (int) get_Main().rowPicker1(r);

else if (r < 0.495710003) pick = (int) get_Main().rowPicker2(r);

else if (r < 0.684744908) pick = (int) get_Main().rowPicker3(r);

else if (r < 0.866907023) pick = (int) get_Main().rowPicker4(r);

else pick = (int) get_Main().rowPicker5(r);

//find the right row

int c = pick + 20;

if(c == 22) c = 20;

for(int j = pick; j < c; j++) {

if(r > get_Main().GPSandPopulation.getCellNumericValue(1,j,10)) rownum = j;

else break;

}

//find the GPS coordinates and place the patient

double placementLat = get_Main().GPSandPopulation.getCellNumericValue(1,rownum,6);

double placementLong = get_Main().GPSandPopulation.getCellNumericValue(1,rownum,7);

double disperseLat = normal(dispersion*uniform(0.5,10),0);

double disperseLong = normal(dispersion*uniform(0.5,10),0);

int urban1_0 = (int) get_Main().GPSandPopulation.getCellNumericValue(1,rownum,11);

this.urban = (urban1_0 == 1) ? true : false;

if(get_Main().Uganda.getLayers()[1].findPoliticalArea(placementLong + disperseLong, placementLat + disperseLat) == null) { //makes sure the point is on the map

disperseLat = 0;

disperseLong = 0;

}

//move the agent and family. Reconnecting to the villages happens on arrival

moveTo(placementLong + disperseLong, placementLat + disperseLat);

this.latitude = placementLat + disperseLat;

this.longitude = placementLong + disperseLong;

//Person locus = getNearestAgent(get_Main().persons);

//for(Person connector : locus.connections.getConnections()) this.connections.connectTo(connector);

if(this.spouse.isConnected()) {

Person wife = this.spouse.getConnectedAgent();

wife.connections.disconnectFromAll();

wife.latitude = this.latitude + uniform(-5/1.15/60/2, 5/1.15/60/2);

wife.longitude = this.longitude + uniform(-5/1.15/60/2, 5/1.15/60/2);

wife.urban = (urban1_0 == 1) ? true : false;

wife.moveTo(wife.longitude, wife.latitude);

//for(Person d : this.connections.getConnections()) wife.connections.connectTo(d);

}

for(Person k : this.kids.getConnections()) {

k.connections.disconnectFromAll();

k.latitude = this.latitude + uniform(-2/1.15/60/2, 2/1.15/60/2);

k.longitude = this.longitude + uniform(-2/1.15/60/2, 2/1.15/60/2);

k.urban = (urban1_0 == 1) ? true : false;

k.moveTo(k.longitude, k.latitude);

//for(Person e : this.connections.getConnections()) k.connections.connectTo(e);

}

}

else { // Leave Uganda (1/1000)

//remove linkage to spouse

if(spouse.isConnected()) spouse.disconnect();

//remove linkage to father

if(father.isConnected()) father.disconnect();

//remove linkage to mother

if(mother.isConnected()) mother.disconnect();

//remove linkages to your kids

if(kids.getConnectionsNumber() > 0) {

if(male) {

for(Person k : kids.getConnections()) {

k.father.disconnect();

}

} else {

for(Person k : kids.getConnections()) {

k.mother.disconnect();

}

}

}

kids.disconnectFromAll();

//remove linkages to fellow villagers

connections.disconnectFromAll();

//and remove me

get_Main().remove_persons(this);

}]]></EntryAction>

<FillColor>-4565549</FillColor>

</Properties>

</StatechartElement>

<StatechartElement Class="Branch" ParentState="Female" ParentStateId="1391567760449">

<Id>1392912246951</Id>

<Name><![CDATA[migrateF]]></Name>

<X>740</X><Y>430</Y>

<Label><X>10</X><Y>0</Y></Label>

<PublicFlag>false</PublicFlag>

<PresentationFlag>true</PresentationFlag>

<ShowLabel>true</ShowLabel>

<Properties>

</Properties>

</StatechartElement>

<StatechartElement Class="State" ParentState="Female" ParentStateId="1391567760449">

<Id>1392912246953</Id>

<Name><![CDATA[MigratingF]]></Name>

<X>690</X><Y>460</Y>

<Label><X>10</X><Y>10</Y></Label>

<PublicFlag>false</PublicFlag>

<PresentationFlag>true</PresentationFlag>

<ShowLabel>true</ShowLabel>

<Properties Width="100" Height="30">

<EntryAction><![CDATA[// 1/1000 emigrate from Uganda per year. 5.2 percent migrate somewhere in Uganda. So, 5.3% will enter this state

// of which (0.001/0.053 = 0.018867925) will emigrate

double s = uniform(0,1);

if(s > 0.018867925) { //move to somewhere else in Uganda

//Disconnect from village connections

this.connections.disconnectFromAll();

//Pick where you're going

int rownum = 2;

int pick = 0;

double dispersion = get_Main().placementRadius/1.15/60/2;

double r = uniform(0,1);

if (r < 0.303941182) pick = (int) get_Main().rowPicker1(r);

else if (r < 0.495710003) pick = (int) get_Main().rowPicker2(r);

else if (r < 0.684744908) pick = (int) get_Main().rowPicker3(r);

else if (r < 0.866907023) pick = (int) get_Main().rowPicker4(r);

else pick = (int) get_Main().rowPicker5(r);

//find the right row

int c = pick + 20;

if(c == 22) c = 20;

for(int j = pick; j < c; j++) {

if(r > get_Main().GPSandPopulation.getCellNumericValue(1,j,10)) rownum = j;

else break;

}

//find the GPS coordinates and place the patient

double placementLat = get_Main().GPSandPopulation.getCellNumericValue(1,rownum,6);

double placementLong = get_Main().GPSandPopulation.getCellNumericValue(1,rownum,7);

double disperseLat = normal(dispersion*uniform(0.5,10),0);

double disperseLong = normal(dispersion*uniform(0.5,10),0);

if(get_Main().Uganda.getLayers()[1].findPoliticalArea(placementLong + disperseLong, placementLat + disperseLat) == null) { //makes sure the point is on the map

disperseLat = 0;

disperseLong = 0;

}

int urban1_0 = (int) get_Main().GPSandPopulation.getCellNumericValue(1,rownum,11);

this.urban = (urban1_0 == 1) ? true : false;

//move the agent and family. Reconnection to villages happens in the arrival transition

moveTo(placementLong + disperseLong, placementLat + disperseLat);

this.latitude = placementLat + disperseLat;

this.longitude = placementLong + disperseLong;

//Person locus = getNearestAgent(get_Main().persons);

//for(Person connector : locus.connections.getConnections()) this.connections.connectTo(connector);

if(this.spouse.isConnected()) {

Person husband = this.spouse.getConnectedAgent();

husband.connections.disconnectFromAll();

husband.latitude = this.latitude + uniform(-5/1.15/60/2, 5/1.15/60/2);

husband.longitude = this.longitude + uniform(-5/1.15/60/2, 5/1.15/60/2);

husband.urban = (urban1_0 == 1) ? true : false;

husband.moveTo(husband.longitude, husband.latitude);

//for(Person d : this.connections.getConnections()) wife.connections.connectTo(d);

}

for(Person k : this.kids.getConnections()) {

k.connections.disconnectFromAll();

k.latitude = this.latitude + uniform(-2/1.15/60/2, 2/1.15/60/2);

k.longitude = this.longitude + uniform(-2/1.15/60/2, 2/1.15/60/2);

k.urban = (urban1_0 == 1) ? true : false;

k.moveTo(k.longitude, k.latitude);

//for(Person e : this.connections.getConnections()) k.connections.connectTo(e);

}

}

else { // Leave Uganda (1/1000)

//remove linkage to spouse

if(spouse.isConnected()) spouse.disconnect();

//remove linkage to father

if(father.isConnected()) father.disconnect();

//remove linkage to mother

if(mother.isConnected()) mother.disconnect();

//remove linkages to your kids

if(kids.getConnectionsNumber() > 0) {

if(male) {

for(Person k : kids.getConnections()) {

k.father.disconnect();

}

} else {

for(Person k : kids.getConnections()) {

k.mother.disconnect();

}

}

}

kids.disconnectFromAll();

//remove linkages to fellow villagers

connections.disconnectFromAll();

//and remove me

get_Main().remove_persons(this);

}

/**

// 1/1000 emigrate from Uganda per year. 5.2 percent migrate somewhere in Uganda. So, 5.3% will enter this state

// of which (0.001/0.053 = 0.018867925) will emigrate

double s = uniform(0,1);

if(s > 0.018867925) { //move to somewhere else in Uganda

//When you move whole families, this has to change

this.disconnectFromAll();

if(spouse != null) {

spouse.spouse = null;

spouse = null;

}

if(father != null) {

father.kids.remove(this);

father = null;

}

if(mother != null) {

mother.kids.remove(this);

mother = null;

}

if(kids.size() != 0) {

if(male) for(Person k : kids) k.father = null;

else for(Person k : kids) k.mother = null;

kids.removeAll(kids);

}

//end family ties

int rownum = 2;

int pick = 0;

double dispersion = get_Main().placementRadius/1.15/60/2;

double r = uniform(0,1);

if (r < 0.303941182) pick = (int) get_Main().rowPicker1(r);

else if (r < 0.495710003) pick = (int) get_Main().rowPicker2(r);

else if (r < 0.684744908) pick = (int) get_Main().rowPicker3(r);

else if (r < 0.866907023) pick = (int) get_Main().rowPicker4(r);

else pick = (int) get_Main().rowPicker5(r);

//find the right row

int k = pick + 20;

if(k == 22) k = 20;

for(int j = pick; j < k; j++) {

if(r > get_Main().GPSandPopulation.getCellNumericValue(1,j,10)) rownum = j;

else break;

}

//find the GPS coordinates and place the patient

double placementLat = get_Main().GPSandPopulation.getCellNumericValue(1,rownum,6);

double placementLong = get_Main().GPSandPopulation.getCellNumericValue(1,rownum,7);

double disperseLat = normal(dispersion*uniform(0.5,5),0);

double disperseLong = normal(dispersion*uniform(0.5,5),0);

if(get_Main().Uganda.getLayers()[1].findPoliticalArea(placementLong + disperseLong, placementLat + disperseLat) == null) { //makes sure the point is on the map

disperseLat = 0;

disperseLong = 0;

}

moveTo(placementLong + disperseLong, placementLat + disperseLat);

}

else { // Leave Uganda (1/1000)

//remove linkage to spouse

if(spouse != null) spouse.spouse = null;

//remove linkage to father

if(father != null) father.kids.remove(this);

//remove linkage to mother

if(mother != null) mother.kids.remove(this);

//remove linkages to your kids

if(kids.size() != 0) {

if(male) {

for(Person k : kids) k.father = null;

} else {

for(Person k : kids) k.mother = null;

}

}

//remove linkages to fellow villagers

disconnectFromAll();

//and remove me

get_Main().remove_persons(this);

}

**/]]></EntryAction>

<FillColor>-4565549</FillColor>

</Properties>

</StatechartElement>

<StatechartElement Class="State" ParentState="SickM" ParentStateId="1391286875465">

<Id>1393893169443</Id>

<Name><![CDATA[TestM]]></Name>

<X>300</X><Y>330</Y>

<Label><X>10</X><Y>10</Y></Label>

<PublicFlag>false</PublicFlag>

<PresentationFlag>true</PresentationFlag>

<ShowLabel>true</ShowLabel>

<Properties Width="60" Height="30">

<FillColor>-6632142</FillColor>

</Properties>

</StatechartElement>

<StatechartElement Class="State" ParentState="SickM" ParentStateId="1391286875465">

<Id>1393893207860</Id>

<Name><![CDATA[TravelingM]]></Name>

<X>510</X><Y>330</Y>

<Label><X>10</X><Y>10</Y></Label>

<PublicFlag>false</PublicFlag>

<PresentationFlag>true</PresentationFlag>

<ShowLabel>true</ShowLabel>

<Properties Width="100" Height="30">

<EntryAction><![CDATA[/**

String type = this.chosenHospital.getClass().getSimpleName().toString();

String gender = this.male ? "Male" : "Female";

if(type.equals("Hospital")){

String name = ((Hospital) this.chosenHospital).name;

System.out.println("Gender: " + gender + ". RunCount: " + get_Main().runCount + ". Destination: " + name);

} else if(type.equals("Regional")) {

String name = ((Regional) this.chosenHospital).name;

System.out.println("Gender: " + gender + ". RunCount: " + get_Main().runCount + ". Destination: " + name);

} else if(type.equals("District")) {

String name = ((District) this.chosenHospital).name;

System.out.println("Gender: " + gender + ". RunCount: " + get_Main().runCount + ". Destination: " + name);

} else error( "Actual type: " + type + "...woooo");

**/

this.moveTo(this.destLong, this.destLat);]]></EntryAction>

<FillColor>-6632142</FillColor>

</Properties>

</StatechartElement>

<StatechartElement Class="State" ParentState="Male" ParentStateId="1391299880932">

<Id>1393893248502</Id>

<Name><![CDATA[TreatmentM]]></Name>

<X>300</X><Y>450</Y>

<Label><X>10</X><Y>10</Y></Label>

<PublicFlag>false</PublicFlag>

<PresentationFlag>true</PresentationFlag>

<ShowLabel>true</ShowLabel>

<Properties Width="100" Height="30">

<EntryAction><![CDATA[//Add the person to the treated queue

get_Main().cancerTx.add(this);

switch(quintile) {

case 1: get_Main().cancTxPoorest++;

break;

case 2: get_Main().cancTxPoor++;

break;

case 3: get_Main().cancTxMiddle++;

break;

case 4: get_Main().cancTxRich++;

break;

case 5: get_Main().cancTxRichest++;

break;

default: error("No such quintile (TreatmentM)");

}

//Determine how much the system costs and personal costs are

get_Main().systemCost = get_Main().systemCost + systCost;

double prior = this.wealth;

double post = this.wealth - (double) persCost;

if(persCost > 0) {

//Catastrophic expenditure? Add myself, my spouse, and my kids if I have them

if(post < prior * (1.0 - get_Main().catExpThreshold)) { //catExpThreshold = 0.1, so want to know if post <= 0.9*prior

switch(this.quintile) {

case 1:

get_Main().catExpPoorest++;

if(this.spouse.isConnected()) get_Main().catExpPoorest++;

get_Main().catExpPoorest += this.kids.size();

break;

case 2:

get_Main().catExpPoor++;

if(this.spouse.isConnected()) get_Main().catExpPoor++;

get_Main().catExpPoor += this.kids.size();

break;

case 3:

get_Main().catExpMiddle++;

if(this.spouse.isConnected()) get_Main().catExpMiddle++;

get_Main().catExpMiddle += this.kids.size();

break;

case 4:

get_Main().catExpRich++;

if(this.spouse.isConnected()) get_Main().catExpRich++;

get_Main().catExpRich += this.kids.size();

break;

case 5:

get_Main().catExpRichest++;

if(this.spouse.isConnected()) get_Main().catExpRichest++;

get_Main().catExpRichest += this.kids.size();

break;

default: error("No such quintile! (CatExp)");

}

}

// If post <= povLine, then switch on quintile.

// Add me to the appropriate list of poverty

// Get spouse and add her to the appropriate list of poverty

// Make spouse.poor = true and spouse.quintile = 1

// Get kids and add them to the appropriate list of poverty

// Make all kids.poor = true and all kids.quintile = 1

//Am I below the poverty line? (irrespective of whether or not I was before)?

if(post <= get_Main().povertyLine) {

//make myself and my spouse poor

this.poor = true;

this.quintile = 1;

if(this.spouse.isConnected()) {

this.spouse.getConnectedAgent().poor = true;

this.spouse.getConnectedAgent().quintile = 1;

}

//make my kids poor

if(this.kids.size() > 0) {

for(Person k : this.kids.getConnections()) {

k.poor = true;

k.quintile = 1;

}

}

//add myself and my family to the appropriate dataset, depending on whether I was already below the poverty line.

switch(this.quintile) {

case 1:

get_Main().madePoorPoorest++;

if(this.spouse.isConnected()) get_Main().madePoorPoorest++;

get_Main().madePoorPoorest += this.kids.size();

if(!this.poor) {

get_Main().madePoorNewPoorest++;

if(this.spouse.isConnected()) get_Main().madePoorNewPoorest++;

get_Main().madePoorNewPoorest += this.kids.size();

}

break;

case 2:

get_Main().madePoorPoor++;

if(this.spouse.isConnected()) get_Main().madePoorPoor++;

get_Main().madePoorPoor += this.kids.size();

if(!this.poor) {

get_Main().madePoorNewPoor++;

if(this.spouse.isConnected()) get_Main().madePoorNewPoor++;

get_Main().madePoorNewPoor += this.kids.size();

}

break;

case 3:

get_Main().madePoorMiddle++;

if(this.spouse.isConnected()) get_Main().madePoorMiddle++;

get_Main().madePoorMiddle += this.kids.size();

if(!this.poor) {

get_Main().madePoorNewMiddle++;

if(this.spouse.isConnected()) get_Main().madePoorNewMiddle++;

get_Main().madePoorNewMiddle += this.kids.size();

}

break;

case 4:

get_Main().madePoorRich++;

if(this.spouse.isConnected()) get_Main().madePoorRich++;

get_Main().madePoorRich += this.kids.size();

if(!this.poor) {

get_Main().madePoorNewRich++;

if(this.spouse.isConnected()) get_Main().madePoorNewRich++;

get_Main().madePoorNewRich += this.kids.size();

}

break;

case 5:

get_Main().madePoorRichest++;

if(this.spouse.isConnected()) get_Main().madePoorRichest++;

get_Main().madePoorRichest += this.kids.size();

if(!this.poor) {

get_Main().madePoorNewRichest++;

if(this.spouse.isConnected()) get_Main().madePoorNewRichest++;

get_Main().madePoorNewRichest += this.kids.size();

}

break;

default: error("No such quintile");

}

}

}

//Adjust my wealth, figure out my new quintile, and pass these both on to my family

this.wealth = post;

if(!this.poor) { //if I am not already poor, then calculate my new quintile and poverty flag

if(post <= get_Main().povertyLine) this.poor = true;

if(post > get_Main().cutoff4) this.quintile = 5;

else if(post > get_Main().cutoff3) this.quintile = 4;

else if(post > get_Main().cutoff2) this.quintile = 3;

else if(post > get_Main().cutoff1) this.quintile = 2;

else this.quintile = 1;

if(this.spouse.isConnected()) {

this.spouse.getConnectedAgent().quintile = this.quintile;

this.spouse.getConnectedAgent().poor = this.poor;

this.spouse.getConnectedAgent().wealth = this.wealth;

}

if(this.kids.size() > 0) {

for(Person k : this.kids.getConnections()) {

k.quintile = this.quintile;

k.poor = this.poor;

k.wealth = this.wealth;

}

}

}]]></EntryAction>

<FillColor>-6632142</FillColor>

</Properties>

</StatechartElement>

<StatechartElement Class="State" ParentState="Male" ParentStateId="1391299880932">

<Id>1393893469132</Id>

<Name><![CDATA[RecoveredM]]></Name>

<X>250</X><Y>580</Y>

<Label><X>10</X><Y>10</Y></Label>

<PublicFlag>false</PublicFlag>

<PresentationFlag>true</PresentationFlag>

<ShowLabel>true</ShowLabel>

<Properties Width="190" Height="150">

<FillColor>-657956</FillColor>

</Properties>

</StatechartElement>

<StatechartElement Class="State" ParentState="RecoveredM" ParentStateId="1393893469132">

<Id>1393893571694</Id>

<Name><![CDATA[ReturningM]]></Name>

<X>300</X><Y>600</Y>

<Label><X>10</X><Y>10</Y></Label>

<PublicFlag>false</PublicFlag>

<PresentationFlag>true</PresentationFlag>

<ShowLabel>true</ShowLabel>

<Properties Width="100" Height="30">

<EntryAction><![CDATA[this.moveTo(this.longitude, this.latitude);]]></EntryAction>

<FillColor>-2968436</FillColor>

</Properties>

</StatechartElement>

<StatechartElement Class="State" ParentState="RecoveredM" ParentStateId="1393893469132">

<Id>1393893581754</Id>

<Name><![CDATA[HomeM]]></Name>

<X>300</X><Y>680</Y>

<Label><X>10</X><Y>10</Y></Label>

<PublicFlag>false</PublicFlag>

<PresentationFlag>true</PresentationFlag>

<ShowLabel>true</ShowLabel>

<Properties Width="100" Height="30">

<FillColor>-2968436</FillColor>

</Properties>

</StatechartElement>

<StatechartElement Class="Branch" ParentState="Male" ParentStateId="1391299880932">

<Id>1393898994969</Id>

<Name><![CDATA[TxDeathM]]></Name>

<X>350</X><Y>530</Y>

<Label><X>10</X><Y>0</Y></Label>

<PublicFlag>false</PublicFlag>

<PresentationFlag>true</PresentationFlag>

<ShowLabel>false</ShowLabel>

<Properties>

<Action><![CDATA[//System.out.println(this.chosenHospital.getPopulation() + "\t" + get_Main().periopMortality + "\t" + this.pDieTx);]]></Action>

</Properties>

</StatechartElement>

<StatechartElement Class="State" ParentState="SickF" ParentStateId="1391567760452">

<Id>1394059475276</Id>

<Name><![CDATA[TestF]]></Name>

<X>890</X><Y>330</Y>

<Label><X>10</X><Y>10</Y></Label>

<PublicFlag>false</PublicFlag>

<PresentationFlag>true</PresentationFlag>

<ShowLabel>true</ShowLabel>

<Properties Width="50" Height="30">

<FillColor>-6632142</FillColor>

</Properties>

</StatechartElement>

<StatechartElement Class="State" ParentState="SickF" ParentStateId="1391567760452">

<Id>1394059475279</Id>

<Name><![CDATA[TravelingF]]></Name>

<X>1060</X><Y>330</Y>

<Label><X>10</X><Y>10</Y></Label>

<PublicFlag>false</PublicFlag>

<PresentationFlag>true</PresentationFlag>

<ShowLabel>true</ShowLabel>

<Properties Width="100" Height="30">

<EntryAction><![CDATA[/**

String type = this.chosenHospital.getClass().getSimpleName().toString();

String gender = this.male ? "Male" : "Female";

if(type.equals("Hospital")){

String name = ((Hospital) this.chosenHospital).name;

System.out.println("Gender: " + gender + ". RunCount: " + get_Main().runCount + ". Destination: " + name);

} else if(type.equals("Regional")) {

String name = ((Regional) this.chosenHospital).name;

System.out.println("Gender: " + gender + ". RunCount: " + get_Main().runCount + ". Destination: " + name);

} else if(type.equals("District")) {

String name = ((District) this.chosenHospital).name;

System.out.println("Gender: " + gender + ". RunCount: " + get_Main().runCount + ". Destination: " + name);

} else error( "Actual type: " + type + "...woooo");

**/

this.moveTo(this.destLong, this.destLat);]]></EntryAction>

<FillColor>-6632142</FillColor>

</Properties>

</StatechartElement>

<StatechartElement Class="State" ParentState="Female" ParentStateId="1391567760449">

<Id>1394059475281</Id>

<Name><![CDATA[TreatmentF]]></Name>

<X>890</X><Y>430</Y>

<Label><X>10</X><Y>10</Y></Label>

<PublicFlag>false</PublicFlag>

<PresentationFlag>true</PresentationFlag>

<ShowLabel>true</ShowLabel>

<Properties Width="100" Height="30">

<EntryAction><![CDATA[//Add the person to the treated queue

get_Main().cancerTx.add(this);

switch(quintile) {

case 1: get_Main().cancTxPoorest++;

break;

case 2: get_Main().cancTxPoor++;

break;

case 3: get_Main().cancTxMiddle++;

break;

case 4: get_Main().cancTxRich++;

break;

case 5: get_Main().cancTxRichest++;

break;

default: error("No such quintile (TreatmentF)");

}

//Determine how much the system costs and personal costs are

get_Main().systemCost = get_Main().systemCost + systCost;

double prior = this.wealth;

double post = this.wealth - persCost;

if(persCost > 0) {

//Catastrophic expenditure? Add myself, my spouse, and my kids if I have them

if(post < prior * (1.0 - get_Main().catExpThreshold)) { //catExpThreshold = 0.1, so want to know if post <= 0.9*prior

switch(this.quintile) {

case 1:

get_Main().catExpPoorest++;

if(this.spouse.isConnected()) get_Main().catExpPoorest++;

get_Main().catExpPoorest += this.kids.size();

break;

case 2:

get_Main().catExpPoor++;

if(this.spouse.isConnected()) get_Main().catExpPoor++;

get_Main().catExpPoor += this.kids.size();

break;

case 3:

get_Main().catExpMiddle++;

if(this.spouse.isConnected()) get_Main().catExpMiddle++;

get_Main().catExpMiddle += this.kids.size();

break;

case 4:

get_Main().catExpRich++;

if(this.spouse.isConnected()) get_Main().catExpRich++;

get_Main().catExpRich += this.kids.size();

break;

case 5:

get_Main().catExpRichest++;

if(this.spouse.isConnected()) get_Main().catExpRichest++;

get_Main().catExpRichest += this.kids.size();

break;

default: error("No such quintile! (CatExp)");

}

}

// If post <= povLine, then switch on quintile.

// Switch on whether I am poor or not (poorNum = 0 if not poor, 1 if poor)

// Add me to the appropriate list of poverty

// Get spouse and add her to the appropriate list of poverty

// Make spouse.poor = true and spouse.quintile = 1

// Get kids and add them to the appropriate list of poverty

// Make all kids.poor = true and all kids.quintile = 1

//Am I below the poverty line? (irrespective of whether or not I was before)?

if(post <= get_Main().povertyLine) {

//make myself and my spouse poor

this.poor = true;

this.quintile = 1;

if(this.spouse.isConnected()) {

this.spouse.getConnectedAgent().poor = true;

this.spouse.getConnectedAgent().quintile = 1;

}

//make my kids poor

if(this.kids.size() > 0) {

for(Person k : this.kids.getConnections()) {

k.poor = true;

k.quintile = 1;

}

}

//add myself and my family to the appropriate dataset, depending on whether I was already below the poverty line.

switch(this.quintile) {

case 1:

get_Main().madePoorPoorest++;

if(this.spouse.isConnected()) get_Main().madePoorPoorest++;

get_Main().madePoorPoorest += this.kids.size();

if(!this.poor) {

get_Main().madePoorNewPoorest++;

if(this.spouse.isConnected()) get_Main().madePoorNewPoorest++;

get_Main().madePoorNewPoorest += this.kids.size();

}

break;

case 2:

get_Main().madePoorPoor++;

if(this.spouse.isConnected()) get_Main().madePoorPoor++;

get_Main().madePoorPoor += this.kids.size();

if(!this.poor) {

get_Main().madePoorNewPoor++;

if(this.spouse.isConnected()) get_Main().madePoorNewPoor++;

get_Main().madePoorNewPoor += this.kids.size();

}

break;

case 3:

get_Main().madePoorMiddle++;

if(this.spouse.isConnected()) get_Main().madePoorMiddle++;

get_Main().madePoorMiddle += this.kids.size();

if(!this.poor) {

get_Main().madePoorNewMiddle++;

if(this.spouse.isConnected()) get_Main().madePoorNewMiddle++;

get_Main().madePoorNewMiddle += this.kids.size();

}

break;

case 4:

get_Main().madePoorRich++;

if(this.spouse.isConnected()) get_Main().madePoorRich++;

get_Main().madePoorRich += this.kids.size();

if(!this.poor) {

get_Main().madePoorNewRich++;

if(this.spouse.isConnected()) get_Main().madePoorNewRich++;

get_Main().madePoorNewRich += this.kids.size();

}

break;

case 5:

get_Main().madePoorRichest++;

if(this.spouse.isConnected()) get_Main().madePoorRichest++;

get_Main().madePoorRichest += this.kids.size();

if(!this.poor) {

get_Main().madePoorNewRichest++;

if(this.spouse.isConnected()) get_Main().madePoorNewRichest++;

get_Main().madePoorNewRichest += this.kids.size();

}

break;

default: error("No such quintile");

}

}

}

//Adjust my wealth, figure out my new quintile, and pass these both on to my family

this.wealth = post;

if(!this.poor) { //if I am not already poor, then calculate my new quintile and poverty flag

if(post <= get_Main().povertyLine) this.poor = true;

if(post > get_Main().cutoff4) this.quintile = 5;

else if(post > get_Main().cutoff3) this.quintile = 4;

else if(post > get_Main().cutoff2) this.quintile = 3;

else if(post > get_Main().cutoff1) this.quintile = 2;

else this.quintile = 1;

if(this.spouse.isConnected()) {

this.spouse.getConnectedAgent().quintile = this.quintile;

this.spouse.getConnectedAgent().poor = this.poor;

this.spouse.getConnectedAgent().wealth = this.wealth;

}

if(this.kids.size() > 0) {

for(Person k : this.kids.getConnections()) {

k.quintile = this.quintile;

k.poor = this.poor;

k.wealth = this.wealth;

}

}

}]]></EntryAction>

<FillColor>-6632142</FillColor>

</Properties>

</StatechartElement>

<StatechartElement Class="State" ParentState="Female" ParentStateId="1391567760449">

<Id>1394059475283</Id>

<Name><![CDATA[RecoveredF]]></Name>

<X>840</X><Y>570</Y>

<Label><X>10</X><Y>10</Y></Label>

<PublicFlag>false</PublicFlag>

<PresentationFlag>true</PresentationFlag>

<ShowLabel>true</ShowLabel>

<Properties Width="190" Height="140">

<FillColor>-657956</FillColor>

</Properties>

</StatechartElement>

<StatechartElement Class="State" ParentState="RecoveredF" ParentStateId="1394059475283">

<Id>1394059475285</Id>

<Name><![CDATA[ReturningF]]></Name>

<X>890</X><Y>590</Y>

<Label><X>10</X><Y>10</Y></Label>

<PublicFlag>false</PublicFlag>

<PresentationFlag>true</PresentationFlag>

<ShowLabel>true</ShowLabel>

<Properties Width="100" Height="30">

<EntryAction><![CDATA[this.moveTo(this.longitude, this.latitude);]]></EntryAction>

<FillColor>-2968436</FillColor>

</Properties>

</StatechartElement>

<StatechartElement Class="State" ParentState="RecoveredF" ParentStateId="1394059475283">

<Id>1394059475287</Id>

<Name><![CDATA[HomeF]]></Name>

<X>890</X><Y>670</Y>

<Label><X>10</X><Y>10</Y></Label>

<PublicFlag>false</PublicFlag>

<PresentationFlag>true</PresentationFlag>

<ShowLabel>true</ShowLabel>

<Properties Width="100" Height="30">

<FillColor>-2968436</FillColor>

</Properties>

</StatechartElement>

<StatechartElement Class="Branch" ParentState="Female" ParentStateId="1391567760449">

<Id>1394059475289</Id>

<Name><![CDATA[TxDeathF]]></Name>

<X>940</X><Y>490</Y>

<Label><X>10</X><Y>0</Y></Label>

<PublicFlag>false</PublicFlag>

<PresentationFlag>true</PresentationFlag>

<ShowLabel>false</ShowLabel>

<Properties>

</Properties>

</StatechartElement>

<StatechartElement Class="Branch" ParentState="SickM" ParentStateId="1391286875465">

<Id>1410547993448</Id>

<Name><![CDATA[doIGoM]]></Name>

<X>420</X><Y>340</Y>

<Label><X>10</X><Y>0</Y></Label>

<PublicFlag>false</PublicFlag>

<PresentationFlag>true</PresentationFlag>

<ShowLabel>false</ShowLabel>

<Properties>

</Properties>

</StatechartElement>

<StatechartElement Class="State" ParentState="SickM" ParentStateId="1391286875465">

<Id>1410548098252</Id>

<Name><![CDATA[IdleM]]></Name>

<X>370</X><Y>390</Y>

<Label><X>10</X><Y>10</Y></Label>

<PublicFlag>false</PublicFlag>

<PresentationFlag>true</PresentationFlag>

<ShowLabel>true</ShowLabel>

<Properties Width="100" Height="30">

<EntryAction><![CDATA[//String gender = this.male ? "Male" : "Female";

//System.out.println("Gender: " + gender + ". RunCount: " + get_Main().runCount + ". Destination: NULL");]]></EntryAction>

<FillColor>-6632142</FillColor>

</Properties>

</StatechartElement>

<StatechartElement Class="Branch" ParentState="SickF" ParentStateId="1391567760452">

<Id>1410550026703</Id>

<Name><![CDATA[branch1]]></Name>

<X>1000</X><Y>340</Y>

<Label><X>10</X><Y>0</Y></Label>

<PublicFlag>false</PublicFlag>

<PresentationFlag>true</PresentationFlag>

<ShowLabel>false</ShowLabel>

<Properties>

</Properties>

</StatechartElement>

<StatechartElement Class="State" ParentState="SickF" ParentStateId="1391567760452">

<Id>1410550032673</Id>

<Name><![CDATA[IdleF]]></Name>

<X>950</X><Y>370</Y>

<Label><X>10</X><Y>10</Y></Label>

<PublicFlag>false</PublicFlag>

<PresentationFlag>true</PresentationFlag>

<ShowLabel>true</ShowLabel>

<Properties Width="100" Height="30">

<EntryAction><![CDATA[//String gender = this.male ? "Male" : "Female";

//System.out.println("Gender: " + gender + ". RunCount: " + get_Main().runCount + ". Destination: NULL");]]></EntryAction>

<FillColor>-6632142</FillColor>

</Properties>

</StatechartElement>

<StatechartElement Class="Branch" ParentState="Male" ParentStateId="1391299880932">

<Id>1412785465830</Id>

<Name><![CDATA[complM]]></Name>

<X>470</X><Y>570</Y>

<Label><X>-20</X><Y>-20</Y></Label>

<PublicFlag>false</PublicFlag>

<PresentationFlag>true</PresentationFlag>

<ShowLabel>true</ShowLabel>

<Properties>

<Action><![CDATA[//determine whether a complication has happened

if(this.chosenHospital instanceof Hospital) {

Hospital h = (Hospital) this.chosenHospital;

if(h.statechart.isStateActive(h.Trip)) this.compl = randomTrue(get_Main().complSurgRegDist);

else this.compl = randomTrue(get_Main().complSurgHosp);

} else this.compl = randomTrue(get_Main().complSurgRegDist);

if(compl) {

this.persCost += get_Main().OOP * get_Main().cCompl;

this.systCost += (1.0 - get_Main().OOP) * get_Main().cCompl;

this.dieCompl = randomTrue(get_Main().pDieCompl);

}

]]></Action>

</Properties>

</StatechartElement>

<StatechartElement Class="Branch" ParentState="Female" ParentStateId="1391567760449">

<Id>1412786094778</Id>

<Name><![CDATA[complF]]></Name>

<X>1060</X><Y>540</Y>

<Label><X>-20</X><Y>-20</Y></Label>

<PublicFlag>false</PublicFlag>

<PresentationFlag>true</PresentationFlag>

<ShowLabel>true</ShowLabel>

<Properties>

<Action><![CDATA[//determine whether a complication has happened

if(this.chosenHospital instanceof Hospital) {

Hospital h = (Hospital) this.chosenHospital;

if(h.statechart.isStateActive(h.Trip)) this.compl = randomTrue(get_Main().complSurgRegDist);

else this.compl = randomTrue(get_Main().complSurgHosp);

} else this.compl = randomTrue(get_Main().complSurgRegDist);

if(compl) {

this.persCost += get_Main().OOP * get_Main().cCompl;

this.systCost += (1.0 - get_Main().OOP) * get_Main().cCompl;

this.dieCompl = randomTrue(get_Main().pDieCompl);

}

]]></Action>

</Properties>

</StatechartElement>

<StatechartElement Class="EntryPoint" ParentState="ROOT_NODE">

<Id>1391284856814</Id>

<Name><![CDATA[statechart]]></Name>

<X>280</X><Y>10</Y>

<Label><X>-65</X><Y>0</Y></Label>

<PublicFlag>false</PublicFlag>

<PresentationFlag>true</PresentationFlag>

<ShowLabel>true</ShowLabel>

<Points>

<Point><X>0</X><Y>0</Y></Point>

<Point><X>0</X><Y>20</Y></Point>

</Points>

<Properties Target="1391300849644">

</Properties>

</StatechartElement>

<StatechartElement Class="Transition" ParentState="Male" ParentStateId="1391299880932">

<Id>1391287074932</Id>

<Name><![CDATA[getCancerM]]></Name>

<X>200</X><Y>330</Y>

<Label><X>-5</X><Y>-15</Y></Label>

<PublicFlag>false</PublicFlag>

<PresentationFlag>true</PresentationFlag>

<ShowLabel>true</ShowLabel>

<Points>

<Point><X>0</X><Y>0</Y></Point>

<Point><X>50</X><Y>0</Y></Point>

</Points>

<Properties Source="1391284865450" Target="1391286875465" Trigger="rate">

<Action><![CDATA[

get_Main().cancerInc.add(this);

switch(quintile) {

case 1: get_Main().cancIncPoorest++;

break;

case 2: get_Main().cancIncPoor++;

break;

case 3: get_Main().cancIncMiddle++;

break;

case 4: get_Main().cancIncRich++;

break;

case 5: get_Main().cancIncRichest++;

break;

default: error("No such quintile (getCancerM)");

}

//pick type of cancer and mortality rate

int cancerType = (int) get_Main().selectCancer();

double rDieNoTx = 0;

switch(cancerType) {

case 1: rDieNoTx = 0.540612532;

break;

case 2: rDieNoTx = 0.763342565;

break;

case 3: rDieNoTx = 0.365270183;

break;

case 4: rDieNoTx = 1;

break;

case 5: rDieNoTx = 0.602530453;

break;

case 6: rDieNoTx = 0.580084419;

break;

case 7: rDieNoTx = 0.763342565;

break;

default: error("No such cancer!");

}

//calculate the day you die untreated

double p = rDieNoTx / (1 + rDieNoTx);

cancerDeathDate = geometric(p);]]></Action>

<Timeout><![CDATA[1]]></Timeout>

<Condition><![CDATA[true]]></Condition>

<Rate><![CDATA[get_Main().incMort.getCellNumericValue("Sheet1!C9")/172]]></Rate>

<MessageType><![CDATA[Object]]></MessageType>

<DefaultTransition>true</DefaultTransition>

<FilterType><![CDATA[unconditionally]]></FilterType>

<EqualsExpression><![CDATA["text"]]></EqualsExpression>

<SatisfiesExpression><![CDATA[true]]></SatisfiesExpression>

</Properties>

</StatechartElement>

<StatechartElement Class="Transition" ParentState="Male" ParentStateId="1391299880932">

<Id>1391287215346</Id>

<Name><![CDATA[cancerDeathM]]></Name>

<X>600</X><Y>430</Y>

<Label><X>-80</X><Y>65</Y></Label>

<PublicFlag>false</PublicFlag>

<PresentationFlag>true</PresentationFlag>

<ShowLabel>true</ShowLabel>

<Points>

<Point><X>0</X><Y>0</Y></Point>

<Point><X>0</X><Y>92</Y></Point>

</Points>

<Properties Source="1391286875465" Target="1391299968072" Trigger="timeout">

<Timeout><![CDATA[cancerDeathDate]]></Timeout>

<Condition><![CDATA[true]]></Condition>

<Rate><![CDATA[1/(get_Main().untreatedMeanSurvival)]]></Rate>

<MessageType><![CDATA[Object]]></MessageType>

<DefaultTransition>true</DefaultTransition>

<FilterType><![CDATA[unconditionally]]></FilterType>

<EqualsExpression><![CDATA["text"]]></EqualsExpression>

<SatisfiesExpression><![CDATA[true]]></SatisfiesExpression>

</Properties>

</StatechartElement>

<StatechartElement Class="Transition" ParentState="ROOT_NODE">

<Id>1391297639950</Id>

<Name><![CDATA[DOC]]></Name>

<Description><![CDATA[Everyone dies after they reach 100.

This NEEDS TO BACKOUT the total cancer mortality from GBD, so that actual cancer mortality can be incorporated.]]></Description>

<X>1250</X><Y>300</Y>

<Label><X>25</X><Y>-10</Y></Label>

<PublicFlag>false</PublicFlag>

<PresentationFlag>true</PresentationFlag>

<ShowLabel>true</ShowLabel>

<Points>

<Point><X>0</X><Y>0</Y></Point>

<Point><X>50</X><Y>0</Y></Point>

<Point><X>50</X><Y>42</Y></Point>

</Points>

<Properties Source="1391299759829" Target="1391287000817" Trigger="timeout">

<Action><![CDATA[get_Main().DeadThisYear++;

if(age<2) get_Main().infantDeaths++;]]></Action>

<Timeout><![CDATA[deathDate()]]></Timeout>

<Condition><![CDATA[true]]></Condition>

<Rate><![CDATA[age >= 100 ? 1000000 : (male ? get_Main().maleLifeTable(age) - get_Main().maleBackoutMort(age) : get_Main().femaleLifeTable(age) - get_Main().femaleBackoutMort(age))]]></Rate>

<MessageType><![CDATA[Object]]></MessageType>

<DefaultTransition>true</DefaultTransition>

<FilterType><![CDATA[unconditionally]]></FilterType>

<EqualsExpression><![CDATA["text"]]></EqualsExpression>

<SatisfiesExpression><![CDATA[true]]></SatisfiesExpression>

</Properties>

</StatechartElement>

<StatechartElement Class="Transition" ParentState="Alive" ParentStateId="1391299759829">

<Id>1391300151361</Id>

<Name><![CDATA[toU5]]></Name>

<X>120</X><Y>160</Y>

<Label><X>10</X><Y>0</Y></Label>

<PublicFlag>false</PublicFlag>

<PresentationFlag>true</PresentationFlag>

<ShowLabel>false</ShowLabel>

<Points>

<Point><X>0</X><Y>0</Y></Point>

<Point><X>110</X><Y>0</Y></Point>

</Points>

<Properties Source="1391299800636" Target="1391300108314" Trigger="condition">

<Timeout><![CDATA[1]]></Timeout>

<Condition><![CDATA[age >= 1]]></Condition>

<Rate><![CDATA[1]]></Rate>

<MessageType><![CDATA[Object]]></MessageType>

<DefaultTransition>true</DefaultTransition>

<FilterType><![CDATA[unconditionally]]></FilterType>

<EqualsExpression><![CDATA["text"]]></EqualsExpression>

<SatisfiesExpression><![CDATA[true]]></SatisfiesExpression>

</Properties>

</StatechartElement>

<StatechartElement Class="InitialStatePointer" ParentState="Male" ParentStateId="1391299880932">

<Id>1391300296466</Id>

<Name><![CDATA[initialState1]]></Name>

<X>130</X><Y>300</Y>

<Label><X>10</X><Y>0</Y></Label>

<PublicFlag>false</PublicFlag>

<PresentationFlag>true</PresentationFlag>

<ShowLabel>false</ShowLabel>

<Points>

<Point><X>0</X><Y>0</Y></Point>

<Point><X>0</X><Y>20</Y></Point>

</Points>

<Properties Target="1391284865450">

</Properties>

</StatechartElement>

<StatechartElement Class="Transition" ParentState="Alive" ParentStateId="1391299759829">

<Id>1391300318546</Id>

<Name><![CDATA[startSickM]]></Name>

<X>242</X><Y>250</Y>

<Label><X>8</X><Y>-10</Y></Label>

<PublicFlag>false</PublicFlag>

<PresentationFlag>true</PresentationFlag>

<ShowLabel>true</ShowLabel>

<Points>

<Point><X>0</X><Y>0</Y></Point>

<Point><X>78</X><Y>0</Y></Point>

<Point><X>78</X><Y>70</Y></Point>

</Points>

<Properties Source="1391300283103" Target="1391286875465" Trigger="timeout">

<Timeout><![CDATA[1]]></Timeout>

<Condition><![CDATA[randomTrue(get_Main().incMort.getCellNumericValue("Sheet1!G8")/100000) ]]></Condition>

<Rate><![CDATA[1]]></Rate>

<MessageType><![CDATA[Object]]></MessageType>

<DefaultTransition>false</DefaultTransition>

<FilterType><![CDATA[unconditionally]]></FilterType>

<EqualsExpression><![CDATA["text"]]></EqualsExpression>

<SatisfiesExpression><![CDATA[true]]></SatisfiesExpression>

</Properties>

</StatechartElement>

<StatechartElement Class="Transition" ParentState="Alive" ParentStateId="1391299759829">

<Id>1391300430872</Id>

<Name><![CDATA[startHealthyM]]></Name>

<X>218</X><Y>250</Y>

<Label><X>-78</X><Y>-10</Y></Label>

<PublicFlag>false</PublicFlag>

<PresentationFlag>true</PresentationFlag>

<ShowLabel>true</ShowLabel>

<Points>

<Point><X>0</X><Y>0</Y></Point>

<Point><X>-78</X><Y>0</Y></Point>

<Point><X>-78</X><Y>70</Y></Point>

</Points>

<Properties Source="1391300283103" Target="1391284865450" Trigger="timeout">

<Action><![CDATA[if(time() != 0) send("Migrate?", this);]]></Action>

<Timeout><![CDATA[1]]></Timeout>

<Condition><![CDATA[true]]></Condition>

<Rate><![CDATA[1]]></Rate>

<MessageType><![CDATA[Object]]></MessageType>

<DefaultTransition>true</DefaultTransition>

<FilterType><![CDATA[unconditionally]]></FilterType>

<EqualsExpression><![CDATA["text"]]></EqualsExpression>

<SatisfiesExpression><![CDATA[true]]></SatisfiesExpression>

</Properties>

</StatechartElement>

<StatechartElement Class="Transition" ParentState="Alive" ParentStateId="1391299759829">

<Id>1391300486160</Id>

<Name><![CDATA[toAdult]]></Name>

<X>460</X><Y>180</Y>

<Label><X>10</X><Y>0</Y></Label>

<PublicFlag>false</PublicFlag>

<PresentationFlag>true</PresentationFlag>

<ShowLabel>false</ShowLabel>

<Points>

<Point><X>0</X><Y>0</Y></Point>

<Point><X>0</X><Y>20</Y></Point>

<Point><X>48</X><Y>40</Y></Point>

</Points>

<Properties Source="1392245582239" Target="1391568138614" Trigger="condition">

<Timeout><![CDATA[uniform_discr(15,18)*365]]></Timeout>

<Condition><![CDATA[age > 15]]></Condition>

<Rate><![CDATA[1]]></Rate>

<MessageType><![CDATA[Object]]></MessageType>

<DefaultTransition>true</DefaultTransition>

<FilterType><![CDATA[unconditionally]]></FilterType>

<EqualsExpression><![CDATA["text"]]></EqualsExpression>

<SatisfiesExpression><![CDATA[true]]></SatisfiesExpression>

</Properties>

</StatechartElement>

<StatechartElement Class="Transition" ParentState="ROOT_NODE">

<Id>1391300889836</Id>

<Name><![CDATA[birth]]></Name>

<X>268</X><Y>40</Y>

<Label><X>-98</X><Y>-10</Y></Label>

<PublicFlag>false</PublicFlag>

<PresentationFlag>true</PresentationFlag>

<ShowLabel>true</ShowLabel>

<Points>

<Point><X>0</X><Y>0</Y></Point>

<Point><X>-168</X><Y>0</Y></Point>

<Point><X>-168</X><Y>80</Y></Point>

</Points>

<Properties Source="1391300849644" Target="1391299759829" Trigger="timeout">

<Timeout><![CDATA[1]]></Timeout>

<Condition><![CDATA[time() >= 1]]></Condition>

<Rate><![CDATA[1]]></Rate>

<MessageType><![CDATA[Object]]></MessageType>

<DefaultTransition>false</DefaultTransition>

<FilterType><![CDATA[unconditionally]]></FilterType>

<EqualsExpression><![CDATA["text"]]></EqualsExpression>

<SatisfiesExpression><![CDATA[true]]></SatisfiesExpression>

</Properties>

</StatechartElement>

<StatechartElement Class="Transition" ParentState="ROOT_NODE">

<Id>1391300954174</Id>

<Name><![CDATA[startingPop]]></Name>

<X>292</X><Y>40</Y>

<Label><X>13</X><Y>-10</Y></Label>

<PublicFlag>false</PublicFlag>

<PresentationFlag>true</PresentationFlag>

<ShowLabel>true</ShowLabel>

<Points>

<Point><X>0</X><Y>0</Y></Point>

<Point><X>96</X><Y>0</Y></Point>

</Points>

<Properties Source="1391300849644" Target="1391300938409" Trigger="timeout">

<Timeout><![CDATA[1]]></Timeout>

<Condition><![CDATA[true]]></Condition>

<Rate><![CDATA[1]]></Rate>

<MessageType><![CDATA[Object]]></MessageType>

<DefaultTransition>true</DefaultTransition>

<FilterType><![CDATA[unconditionally]]></FilterType>

<EqualsExpression><![CDATA["text"]]></EqualsExpression>

<SatisfiesExpression><![CDATA[true]]></SatisfiesExpression>

</Properties>

</StatechartElement>

<StatechartElement Class="Transition" ParentState="ROOT_NODE">

<Id>1391301263375</Id>

<Name><![CDATA[startU5]]></Name>

<Description><![CDATA[For model initiation

This assigns a uniform_discrete yearBorn of 0 to 5 years before model initiation]]></Description>

<X>388</X><Y>40</Y>

<Label><X>-168</X><Y>50</Y></Label>

<PublicFlag>false</PublicFlag>

<PresentationFlag>true</PresentationFlag>

<ShowLabel>true</ShowLabel>

<Points>

<Point><X>0</X><Y>0</Y></Point>

<Point><X>-118</X><Y>30</Y></Point>

<Point><X>-118</X><Y>110</Y></Point>

</Points>

<Properties Source="1391300938409" Target="1391300108314" Trigger="timeout">

<Action><![CDATA[yearBorn = roundToInt(getYear() - triangular(1,5,1));

age = ageFunc();

]]></Action>

<Timeout><![CDATA[1]]></Timeout>

<Condition><![CDATA[startAsAdult == false]]></Condition>

<Rate><![CDATA[1]]></Rate>

<MessageType><![CDATA[Object]]></MessageType>

<DefaultTransition>true</DefaultTransition>

<FilterType><![CDATA[unconditionally]]></FilterType>

<EqualsExpression><![CDATA["text"]]></EqualsExpression>

<SatisfiesExpression><![CDATA[true]]></SatisfiesExpression>

</Properties>

</StatechartElement>

<StatechartElement Class="Transition" ParentState="ROOT_NODE">

<Id>1391301365848</Id>

<Name><![CDATA[startAdult]]></Name>

<Description><![CDATA[For model initiation

This assigns a uniform_discrete yearBorn of 0 to 5 years before model initiation]]></Description>

<X>412</X><Y>40</Y>

<Label><X>58</X><Y>-10</Y></Label>

<PublicFlag>false</PublicFlag>

<PresentationFlag>true</PresentationFlag>

<ShowLabel>true</ShowLabel>

<Points>

<Point><X>0</X><Y>0</Y></Point>

<Point><X>108</X><Y>0</Y></Point>

<Point><X>108</X><Y>170</Y></Point>

</Points>

<Properties Source="1391300938409" Target="1391568138614" Trigger="timeout">

<Action><![CDATA[yearBorn = roundToInt(getYear() - exponential(0.2,18));

age = ageFunc();

]]></Action>

<Timeout><![CDATA[1]]></Timeout>

<Condition><![CDATA[startState == 2]]></Condition>

<Rate><![CDATA[1]]></Rate>

<MessageType><![CDATA[Object]]></MessageType>

<DefaultTransition>false</DefaultTransition>

<FilterType><![CDATA[unconditionally]]></FilterType>

<EqualsExpression><![CDATA["text"]]></EqualsExpression>

<SatisfiesExpression><![CDATA[true]]></SatisfiesExpression>

</Properties>

</StatechartElement>

<StatechartElement Class="InitialStatePointer" ParentState="Female" ParentStateId="1391567760449">

<Id>1391567760458</Id>

<Name><![CDATA[initialState2]]></Name>

<X>720</X><Y>300</Y>

<Label><X>10</X><Y>0</Y></Label>

<PublicFlag>false</PublicFlag>

<PresentationFlag>true</PresentationFlag>

<ShowLabel>false</ShowLabel>

<Points>

<Point><X>0</X><Y>0</Y></Point>

<Point><X>0</X><Y>20</Y></Point>

</Points>

<Properties Target="1391567760454">

</Properties>

</StatechartElement>

<StatechartElement Class="Transition" ParentState="Alive" ParentStateId="1391299759829">

<Id>1391568233108</Id>

<Name><![CDATA[isMale]]></Name>

<X>508</X><Y>220</Y>

<Label><X>-148</X><Y>-10</Y></Label>

<PublicFlag>false</PublicFlag>

<PresentationFlag>true</PresentationFlag>

<ShowLabel>true</ShowLabel>

<Points>

<Point><X>0</X><Y>0</Y></Point>

<Point><X>-278</X><Y>0</Y></Point>

<Point><X>-278</X><Y>20</Y></Point>

</Points>

<Properties Source="1391568138614" Target="1391300283103" Trigger="timeout">

<Action><![CDATA[

if(time() >= 1) {

boolean didIFindOne = false;

//find a wife if unmarried. 57% of urban ugandans are married and 67% of rural ugandans are.

if(!spouse.isConnected()) {

if(randomTrue(0.90)) {

for(Person p : this.connections.getConnections()) {

if (! p.male && !p.spouse.isConnected() && p != this.mother.getConnectedAgent()) {

spouse.connectTo(p); //if a person in the village is female and unmarried, marry her

p.jumpTo(this.longitude + uniform(-5/1.15/60/2,5/1.15/60/2),this.latitude + uniform(-5/1.15/60/2,5/1.15/60/2)); //and bring her to you

p.latitude = p.getLat();

p.longitude = p.getLon();

didIFindOne = true;

break;

}

}

}

}

//if you found a wife, become a HOH, make your village hers, and make her kids yours

if(didIFindOne) {

Person wife = this.spouse.getConnectedAgent();

this.HOH = true;

wife.HOH = false;

//if you haven't had wealth assigned, assign it

if(this.wealth == 0) {

double pickGini = this.urban ? get_Main().urbanGini : get_Main().ruralGini;

double pickGDP = 303700.0; //Uganda as a whole

if(this.urban) {

switch(this.region){

case 1:

pickGDP = 603800.0; //Central

break;

case 2:

pickGDP = 361200.0; //Northern

break;

case 3:

pickGDP = 361000.0; //Eastern

break;

case 4:

pickGDP = 479000.0; //Western

break;

case 5:

pickGDP = 959400.0; //Kampala

break;

default:

pickGDP = 660000.0; //urban Uganda as a whole

}

} else {

switch(this.region){

case 1:

pickGDP = 336800.0; //Central

break;

case 2:

pickGDP = 117200.0; //Northern

break;

case 3:

pickGDP = 151400.0; //Eastern

break;

case 4:

pickGDP = 282300.0; //Western

break;

case 5:

pickGDP = 222600.0; //Kampala

break;

default:

pickGDP = 222600.0; //rural Uganda as a whole

}

}

this.wealth = get_Main().startIncome(pickGDP, pickGini);

if(this.wealth <= get_Main().povertyLine) this.poor = true;

if(this.wealth > get_Main().cutoff4) this.quintile = 5;

else if(this.wealth > get_Main().cutoff3) this.quintile = 4;

else if(this.wealth > get_Main().cutoff2) this.quintile = 3;

else if(this.wealth > get_Main().cutoff1) this.quintile = 2;

else this.quintile = 1;

}

wife.wealth = 0;

wife.poor = this.poor;

wife.quintile = this.quintile;

wife.connections.disconnectFromAll();

for(Person c : this.connections.getConnections()) wife.connections.connectTo(c);

for(Person k : wife.kids.getConnections()) {

k.jumpTo(wife.longitude + uniform(-2/1.15/60/2,2/1.15/60/2), wife.latitude + uniform(-2/1.15/60/2,2/1.15/60/2));

k.latitude = k.getLat();

k.longitude = k.getLon();

k.father.connectTo(this); //this person becomes the kids' father

k.poor = this.poor;

k.quintile = this.quintile;

k.connections.disconnectFromAll();

for(Person v : this.connections.getConnections()) k.connections.connectTo(v);

}

}

}]]></Action>

<Timeout><![CDATA[1]]></Timeout>

<Condition><![CDATA[male]]></Condition>

<Rate><![CDATA[1]]></Rate>

<MessageType><![CDATA[Object]]></MessageType>

<DefaultTransition>false</DefaultTransition>

<FilterType><![CDATA[unconditionally]]></FilterType>

<EqualsExpression><![CDATA["text"]]></EqualsExpression>

<SatisfiesExpression><![CDATA[true]]></SatisfiesExpression>

</Properties>

</StatechartElement>

<StatechartElement Class="Transition" ParentState="Alive" ParentStateId="1391299759829">

<Id>1391568300185</Id>

<Name><![CDATA[isFemale]]></Name>

<X>532</X><Y>220</Y>

<Label><X>33</X><Y>-10</Y></Label>

<PublicFlag>false</PublicFlag>

<PresentationFlag>true</PresentationFlag>

<ShowLabel>true</ShowLabel>

<Points>

<Point><X>0</X><Y>0</Y></Point>

<Point><X>298</X><Y>0</Y></Point>

<Point><X>298</X><Y>20</Y></Point>

</Points>

<Properties Source="1391568138614" Target="1391567996847" Trigger="timeout">

<Action><![CDATA[get_Main().Females.add(this);

//find a husband if unmarried. 57% of urban ugandans are married and 67% of rural ugandans are.

if(time() >= 1) {

boolean didIFindOne = false;

if(!spouse.isConnected()) {

if(randomTrue(0.90)) {

for(Person p : this.connections.getConnections()) {

if (p.male && !p.spouse.isConnected() && p != this.father.getConnectedAgent()) {

spouse.connectTo(p); //if a person in the village is male and unmarried, marry him

this.jumpTo(p.longitude + uniform(-5/1.15/60/2,5/1.15/60/2),p.latitude + uniform(-5/1.15/60/2,5/1.15/60/2)); //and go to him

this.latitude = this.getLat();

this.longitude = this.getLon();

didIFindOne = true;

break;

}

}

}

}

//if you found a husband, he becomes a HOH and you make his village yours and make your kids his

if(didIFindOne) {

Person husband = this.spouse.getConnectedAgent();

husband.HOH = true;

this.HOH = false;

//if husband hasn't had wealth assigned, assign it

if(husband.wealth == 0) {

double pickGini = husband.urban ? get_Main().urbanGini : get_Main().ruralGini;

double pickGDP = 303700.0; //Uganda as a whole

if(husband.urban) {

switch(this.region){

case 1:

pickGDP = 603800.0; //Central

break;

case 2:

pickGDP = 361200.0; //Northern

break;

case 3:

pickGDP = 361000.0; //Eastern

break;

case 4:

pickGDP = 479000.0; //Western

break;

case 5:

pickGDP = 959400.0; //Kampala

break;

default:

pickGDP = 660000.0; //urban Uganda as a whole

}

} else {

switch(husband.region){

case 1:

pickGDP = 336800.0; //Central

break;

case 2:

pickGDP = 117200.0; //Northern

break;

case 3:

pickGDP = 151400.0; //Eastern

break;

case 4:

pickGDP = 282300.0; //Western

break;

case 5:

pickGDP = 222600.0; //Kampala

break;

default:

pickGDP = 222600.0; //rural Uganda as a whole

}

}

husband.wealth = get_Main().startIncome(pickGDP, pickGini);

if(husband.wealth <= get_Main().povertyLine) husband.poor = true;

if(husband.wealth > get_Main().cutoff4) husband.quintile = 5;

else if(husband.wealth > get_Main().cutoff3) husband.quintile = 4;

else if(husband.wealth > get_Main().cutoff2) husband.quintile = 3;

else if(husband.wealth > get_Main().cutoff1) husband.quintile = 2;

else this.quintile = 1;

}

this.poor = husband.poor;

this.quintile = husband.quintile;

this.connections.disconnectFromAll();

for(Person c : husband.connections.getConnections()) this.connections.connectTo(c);

for(Person k : this.kids.getConnections()) {

k.jumpTo(this.longitude + uniform(-2/1.15/60/2,2/1.15/60/2), this.latitude + uniform(-2/1.15/60/2,2/1.15/60/2));

k.latitude = k.getLat();

k.longitude = k.getLon();

k.father.connectTo(husband);

k.poor = husband.poor;

k.quintile = husband.quintile;

k.connections.disconnectFromAll();

for(Person v : husband.connections.getConnections()) k.connections.connectTo(v);

}

}

}]]></Action>

<Timeout><![CDATA[1]]></Timeout>

<Condition><![CDATA[true]]></Condition>

<Rate><![CDATA[1]]></Rate>

<MessageType><![CDATA[Object]]></MessageType>

<DefaultTransition>true</DefaultTransition>

<FilterType><![CDATA[unconditionally]]></FilterType>

<EqualsExpression><![CDATA["text"]]></EqualsExpression>

<SatisfiesExpression><![CDATA[true]]></SatisfiesExpression>

</Properties>

</StatechartElement>

<StatechartElement Class="Transition" ParentState="Alive" ParentStateId="1391299759829">

<Id>1391568913079</Id>

<Name><![CDATA[startSickF]]></Name>

<X>842</X><Y>250</Y>

<Label><X>8</X><Y>-10</Y></Label>

<PublicFlag>false</PublicFlag>

<PresentationFlag>true</PresentationFlag>

<ShowLabel>true</ShowLabel>

<Points>

<Point><X>0</X><Y>0</Y></Point>

<Point><X>68</X><Y>0</Y></Point>

<Point><X>68</X><Y>70</Y></Point>

</Points>

<Properties Source="1391567996847" Target="1391567760452" Trigger="timeout">

<Timeout><![CDATA[1]]></Timeout>

<Condition><![CDATA[randomTrue(get_Main().incMort.getCellNumericValue("Sheet1!M8")/100000)]]></Condition>

<Rate><![CDATA[1]]></Rate>

<MessageType><![CDATA[Object]]></MessageType>

<DefaultTransition>false</DefaultTransition>

<FilterType><![CDATA[unconditionally]]></FilterType>

<EqualsExpression><![CDATA["text"]]></EqualsExpression>

<SatisfiesExpression><![CDATA[true]]></SatisfiesExpression>

</Properties>

</StatechartElement>

<StatechartElement Class="Transition" ParentState="Alive" ParentStateId="1391299759829">

<Id>1391568976228</Id>

<Name><![CDATA[startHealthyF]]></Name>

<X>818</X><Y>250</Y>

<Label><X>-73</X><Y>-10</Y></Label>

<PublicFlag>false</PublicFlag>

<PresentationFlag>true</PresentationFlag>

<ShowLabel>true</ShowLabel>

<Points>

<Point><X>0</X><Y>0</Y></Point>

<Point><X>-88</X><Y>0</Y></Point>

<Point><X>-89</X><Y>70</Y></Point>

</Points>

<Properties Source="1391567996847" Target="1391567760454" Trigger="timeout">

<Action><![CDATA[if(time() != 0) send("Migrate?", this);]]></Action>

<Timeout><![CDATA[1]]></Timeout>

<Condition><![CDATA[true]]></Condition>

<Rate><![CDATA[1]]></Rate>

<MessageType><![CDATA[Object]]></MessageType>

<DefaultTransition>true</DefaultTransition>

<FilterType><![CDATA[unconditionally]]></FilterType>

<EqualsExpression><![CDATA["text"]]></EqualsExpression>

<SatisfiesExpression><![CDATA[true]]></SatisfiesExpression>

</Properties>

</StatechartElement>

<StatechartElement Class="Transition" ParentState="Female" ParentStateId="1391567760449">

<Id>1391569209309</Id>

<Name><![CDATA[cancerDeathF]]></Name>

<X>1160</X><Y>420</Y>

<Label><X>-80</X><Y>45</Y></Label>

<PublicFlag>false</PublicFlag>

<PresentationFlag>true</PresentationFlag>

<ShowLabel>true</ShowLabel>

<Points>

<Point><X>0</X><Y>0</Y></Point>

<Point><X>0</X><Y>62</Y></Point>

</Points>

<Properties Source="1391567760452" Target="1391567760456" Trigger="timeout">

<Timeout><![CDATA[cancerDeathDate]]></Timeout>

<Condition><![CDATA[true]]></Condition>

<Rate><![CDATA[1/get_Main().untreatedMeanSurvival]]></Rate>

<MessageType><![CDATA[Object]]></MessageType>

<DefaultTransition>true</DefaultTransition>

<FilterType><![CDATA[unconditionally]]></FilterType>

<EqualsExpression><![CDATA["text"]]></EqualsExpression>

<SatisfiesExpression><![CDATA[true]]></SatisfiesExpression>

</Properties>

</StatechartElement>

<StatechartElement Class="Transition" ParentState="Female" ParentStateId="1391567760449">

<Id>1391569238748</Id>

<Name><![CDATA[getCancerF]]></Name>

<X>790</X><Y>330</Y>

<Label><X>-5</X><Y>-15</Y></Label>

<PublicFlag>false</PublicFlag>

<PresentationFlag>true</PresentationFlag>

<ShowLabel>true</ShowLabel>

<Points>

<Point><X>0</X><Y>0</Y></Point>

<Point><X>50</X><Y>0</Y></Point>

</Points>

<Properties Source="1391567760454" Target="1391567760452" Trigger="rate">

<Action><![CDATA[

get_Main().cancerInc.add(this);

switch(quintile) {

case 1: get_Main().cancIncPoorest++;

break;

case 2: get_Main().cancIncPoor++;

break;

case 3: get_Main().cancIncMiddle++;

break;

case 4: get_Main().cancIncRich++;

break;

case 5: get_Main().cancIncRichest++;

break;

default: error("No such quintile (getCancerF)");

}

//pick type of cancer and mortality rate

int cancerType = (int) get_Main().selectCancer();

double rDieNoTx = 0;

switch(cancerType) {

case 1: rDieNoTx = 0.540612532;

break;

case 2: rDieNoTx = 0.763342565;

break;

case 3: rDieNoTx = 0.365270183;

break;

case 4: rDieNoTx = 1;

break;

case 5: rDieNoTx = 0.602530453;

break;

case 6: rDieNoTx = 0.580084419;

break;

case 7: rDieNoTx = 0.763342565;

break;

default: error("No such cancer!");

}

//calculate the day you die untreated

double p = rDieNoTx / (1 + rDieNoTx);

cancerDeathDate = geometric(p);]]></Action>

<Timeout><![CDATA[1]]></Timeout>

<Condition><![CDATA[true]]></Condition>

<Rate><![CDATA[get_Main().incMort.getCellNumericValue("Sheet1!I9")/172]]></Rate>

<MessageType><![CDATA[Object]]></MessageType>

<DefaultTransition>true</DefaultTransition>

<FilterType><![CDATA[unconditionally]]></FilterType>

<EqualsExpression><![CDATA["text"]]></EqualsExpression>

<SatisfiesExpression><![CDATA[true]]></SatisfiesExpression>

</Properties>

</StatechartElement>

<StatechartElement Class="Transition" ParentState="Male" ParentStateId="1391299880932">

<Id>1391607255170</Id>

<Name><![CDATA[findWife]]></Name>

<X>80</X><Y>680</Y>

<Label><X>20</X><Y>10</Y></Label>

<PublicFlag>false</PublicFlag>

<PresentationFlag>true</PresentationFlag>

<ShowLabel>true</ShowLabel>

<Points>

<Point><X>0</X><Y>0</Y></Point>

<Point><X>33</X><Y>41</Y></Point>

<Point><X>50</X><Y>60</Y></Point>

</Points>

<Properties Source="1391299880932" Target="1391299880932" Trigger="rate">

<Action><![CDATA[//look for a wife once every three months on average

for(Person p : this.connections.getConnections()) {

if (! p.male && !p.spouse.isConnected()) {

spouse.connectTo(p); //if a person in the village is female and unmarried, marry her

p.jumpTo(this.longitude + uniform(-5/1.15/60/2,5/1.15/60/2),this.latitude + uniform(-5/1.15/60/2,5/1.15/60/2)); //and bring her to you

p.latitude = p.getLat();

p.longitude = p.getLon();

break;

}

}

//if you found a wife, become a HOH, move her to your village and make her kids yours

if(this.spouse.isConnected()) {

Person wife = this.spouse.getConnectedAgent();

this.HOH = true;

wife.HOH = false;

//if you haven't had wealth assigned, assign it

if(this.wealth == 0) {

double pickGini = this.urban ? get_Main().urbanGini : get_Main().ruralGini;

double pickGDP = 303700.0; //Uganda as a whole

if(this.urban) {

switch(this.region){

case 1:

pickGDP = 603800.0; //Central

break;

case 2:

pickGDP = 361200.0; //Northern

break;

case 3:

pickGDP = 361000.0; //Eastern

break;

case 4:

pickGDP = 479000.0; //Western

break;

case 5:

pickGDP = 959400.0; //Kampala

break;

default:

pickGDP = 660000.0; //urban Uganda as a whole

}

} else {

switch(this.region){

case 1:

pickGDP = 336800.0; //Central

break;

case 2:

pickGDP = 117200.0; //Northern

break;

case 3:

pickGDP = 151400.0; //Eastern

break;

case 4:

pickGDP = 282300.0; //Western

break;

case 5:

pickGDP = 222600.0; //Kampala

break;

default:

pickGDP = 222600.0; //rural Uganda as a whole

}

}

this.wealth = get_Main().startIncome(pickGDP, pickGini);

if(this.wealth <= get_Main().povertyLine) this.poor = true;

if(this.wealth > get_Main().cutoff4) this.quintile = 5;

else if(this.wealth > get_Main().cutoff3) this.quintile = 4;

else if(this.wealth > get_Main().cutoff2) this.quintile = 3;

else if(this.wealth > get_Main().cutoff1) this.quintile = 2;

else this.quintile = 1;

}

wife.wealth = this.wealth;

wife.poor = this.poor;

wife.quintile = this.quintile;

wife.connections.disconnectFromAll();

for(Person c : this.connections.getConnections()) wife.connections.connectTo(c);

for(Person k : wife.kids.getConnections()) {

k.jumpTo(wife.longitude + uniform(-2/1.15/60/2,2/1.15/60/2), wife.latitude + uniform(-2/1.15/60/2,2/1.15/60/2));

k.latitude = k.getLat();

k.longitude = k.getLon();

k.poor = this.poor;

k.wealth = 0;

k.quintile = this.quintile;

k.connections.disconnectFromAll();

for(Person v : this.connections.getConnections()) k.connections.connectTo(v);

}

}]]></Action>

<Timeout><![CDATA[182]]></Timeout>

<Condition><![CDATA[true]]></Condition>

<Rate><![CDATA[4/365]]></Rate>

<Guard><![CDATA[!spouse.isConnected();]]></Guard>

<MessageType><![CDATA[Object]]></MessageType>

<DefaultTransition>true</DefaultTransition>

<FilterType><![CDATA[unconditionally]]></FilterType>

<EqualsExpression><![CDATA["text"]]></EqualsExpression>

<SatisfiesExpression><![CDATA[true]]></SatisfiesExpression>

</Properties>

</StatechartElement>

<StatechartElement Class="Transition" ParentState="Alive" ParentStateId="1391299759829">

<Id>1392245698834</Id>

<Name><![CDATA[transition]]></Name>

<X>330</X><Y>160</Y>

<Label><X>10</X><Y>0</Y></Label>

<PublicFlag>false</PublicFlag>

<PresentationFlag>true</PresentationFlag>

<ShowLabel>false</ShowLabel>

<Points>

<Point><X>0</X><Y>0</Y></Point>

<Point><X>70</X><Y>0</Y></Point>

</Points>

<Properties Source="1391300108314" Target="1392245582239" Trigger="condition">

<Timeout><![CDATA[1]]></Timeout>

<Condition><![CDATA[age >= 6]]></Condition>

<Rate><![CDATA[1]]></Rate>

<MessageType><![CDATA[Object]]></MessageType>

<DefaultTransition>true</DefaultTransition>

<FilterType><![CDATA[unconditionally]]></FilterType>

<EqualsExpression><![CDATA["text"]]></EqualsExpression>

<SatisfiesExpression><![CDATA[true]]></SatisfiesExpression>

</Properties>

</StatechartElement>

<StatechartElement Class="Transition" ParentState="ROOT_NODE">

<Id>1392245818447</Id>

<Name><![CDATA[startYouth]]></Name>

<X>412</X><Y>40</Y>

<Label><X>13</X><Y>50</Y></Label>

<PublicFlag>false</PublicFlag>

<PresentationFlag>true</PresentationFlag>

<ShowLabel>true</ShowLabel>

<Points>

<Point><X>0</X><Y>0</Y></Point>

<Point><X>78</X><Y>30</Y></Point>

<Point><X>78</X><Y>110</Y></Point>

</Points>

<Properties Source="1391300938409" Target="1392245582239" Trigger="timeout">

<Action><![CDATA[

yearBorn = getYear() - roundToInt(triangular(6,14,6)); //min max mode

age = ageFunc();

]]></Action>

<Timeout><![CDATA[1]]></Timeout>

<Condition><![CDATA[startState == 1]]></Condition>

<Rate><![CDATA[1]]></Rate>

<MessageType><![CDATA[Object]]></MessageType>

<DefaultTransition>false</DefaultTransition>

<FilterType><![CDATA[unconditionally]]></FilterType>

<EqualsExpression><![CDATA["text"]]></EqualsExpression>

<SatisfiesExpression><![CDATA[true]]></SatisfiesExpression>

</Properties>

</StatechartElement>

<StatechartElement Class="Transition" ParentState="HealthyF" ParentStateId="1391567760454">

<Id>1392317498304</Id>

<Name><![CDATA[haveKidinitial]]></Name>

<Description><![CDATA[Transition is only triggered at the initial population of the model, to create the original families]]></Description>

<X>750</X><Y>320</Y>

<Label><X>30</X><Y>15</Y></Label>

<PublicFlag>false</PublicFlag>

<PresentationFlag>true</PresentationFlag>

<ShowLabel>false</ShowLabel>

<Points>

<Point><X>0</X><Y>0</Y></Point>

<Point><X>20</X><Y>20</Y></Point>

<Point><X>40</X><Y>40</Y></Point>

</Points>

<Properties Source="1391567760454" Target="1391567760454" Trigger="message">

<Action><![CDATA[//ASSUMPTION is that women only have kids when they don't have cancer

for(int i = 0; i < msg; i++) {

Person k = get_Main().add_persons(this.latitude, this.longitude, randomTrue(0.5), randomTrue(get_Main().propU5/get_Main().propKids) ? 0 : 1, this.urban, this.quintile, this.poor, false);

//latitude, longitude, male?, starting state (0 = U5, 1 = Youth, 2 = Adult), urban?, quintile, poor?, HOH?

k.latitude = k.latitude + uniform(-2/1.15/60/2,2/1.15/60/2);

k.longitude = k.longitude + + uniform(-2/1.15/60/2,2/1.15/60/2);

k.wealth = this.wealth;

k.jumpTo(k.longitude, k.latitude);

this.kids.connectTo(k);

k.mother.connectTo(this);

if(this.spouse.isConnected()) {

Person husband = this.spouse.getConnectedAgent();

husband.kids.connectTo(k);

k.father.connectTo(husband);

}

k.connections.disconnectFromAll();

for(Person c : this.connections.getConnections()) k.connections.connectTo(c);

if(k.spouse.isConnected()) k.spouse.disconnect();

//augment TFR and infant mortality rate

TFR = TFR + 1;

get_Main().liveBirths++;

//remove hospital connections

Collection<Agent> c = new LinkedList<Agent>();

for(Agent a : k.connections.getConnections()) if(get_Main().hospitals.contains(a) || get_Main().districts.contains(a) || get_Main().regionals.contains(a)) k.connections.disconnectFrom(a);

}

]]></Action>

<Timeout><![CDATA[1]]></Timeout>

<Condition><![CDATA[true]]></Condition>

<Rate><![CDATA[0.000507655]]></Rate>

<MessageType><![CDATA[Integer]]></MessageType>

<DefaultTransition>true</DefaultTransition>

<FilterType><![CDATA[satisfies]]></FilterType>

<EqualsExpression><![CDATA["text"]]></EqualsExpression>

<SatisfiesExpression><![CDATA[msg > 0]]></SatisfiesExpression>

</Properties>

</StatechartElement>

<StatechartElement Class="Transition" ParentState="Alive" ParentStateId="1391299759829">

<Id>1392412589469</Id>

<Name><![CDATA[yearly]]></Name>

<Description><![CDATA[Does the following things yearly

1. Recalculates age

NO LONGER:

Recalculates when/if the agent is going to die of other causes

a) Determines whether it's time to calculate a new transition (ie, does it at years

0, 1, 5, 10, etc)

b) Takes a draw from a geometric distribution with probability given in the

GBD mortality table.

c) If that length of time is longer than the time before the next age transition,

discards this draw

d) Populates the timeout into the DOC transition]]></Description>

<X>1190</X><Y>120</Y>

<Label><X>-25</X><Y>10</Y></Label>

<PublicFlag>false</PublicFlag>

<PresentationFlag>true</PresentationFlag>

<ShowLabel>true</ShowLabel>

<Points>

<Point><X>0</X><Y>0</Y></Point>

<Point><X>60</X><Y>40</Y></Point>

</Points>

<Properties Source="1391299759829" Target="1391299759829" Trigger="timeout">

<Action><![CDATA[//recalculate age and populate histogram

age = ageFunc();

]]></Action>

<Timeout><![CDATA[365]]></Timeout>

<Condition><![CDATA[true]]></Condition>

<Rate><![CDATA[1]]></Rate>

<MessageType><![CDATA[Object]]></MessageType>

<DefaultTransition>true</DefaultTransition>

<FilterType><![CDATA[unconditionally]]></FilterType>

<EqualsExpression><![CDATA["text"]]></EqualsExpression>

<SatisfiesExpression><![CDATA[true]]></SatisfiesExpression>

</Properties>

</StatechartElement>

<StatechartElement Class="InitialStatePointer" ParentState="Alive" ParentStateId="1391299759829">

<Id>1392412902794</Id>

<Name><![CDATA[initialState]]></Name>

<X>90</X><Y>210</Y>

<Label><X>10</X><Y>0</Y></Label>

<PublicFlag>false</PublicFlag>

<PresentationFlag>true</PresentationFlag>

<ShowLabel>false</ShowLabel>

<Points>

<Point><X>0</X><Y>0</Y></Point>

<Point><X>0</X><Y>-30</Y></Point>

</Points>

<Properties Target="1391299800636">

</Properties>

</StatechartElement>

<StatechartElement Class="Transition" ParentState="HealthyF" ParentStateId="1391567760454">

<Id>1392843299813</Id>

<Name><![CDATA[haveKid]]></Name>

<X>690</X><Y>350</Y>

<Label><X>15</X><Y>20</Y></Label>

<PublicFlag>false</PublicFlag>

<PresentationFlag>true</PresentationFlag>

<ShowLabel>true</ShowLabel>

<Points>

<Point><X>0</X><Y>0</Y></Point>

<Point><X>10</X><Y>18</Y></Point>

<Point><X>30</X><Y>50</Y></Point>

</Points>

<Properties Source="1391567760454" Target="1391567760454" Trigger="rate">

<Action><![CDATA[//ASSUMPTION is that women only have kids when they don't have cancer

//if you are unmarried AND aren't a head of household, you become a head of household and get your own income

if(!this.spouse.isConnected() && this.HOH == false) {

this.HOH = true;

double pickGini = this.urban ? get_Main().urbanGini : get_Main().ruralGini;

double pickGDP = 303700.0; //Uganda as a whole

if(this.urban) {

switch(this.region){

case 1:

pickGDP = 603800.0; //Central

break;

case 2:

pickGDP = 361200.0; //Northern

break;

case 3:

pickGDP = 361000.0; //Eastern

break;

case 4:

pickGDP = 479000.0; //Western

break;

case 5:

pickGDP = 959400.0; //Kampala

break;

default:

pickGDP = 660000.0; //urban Uganda as a whole

}

} else {

switch(this.region){

case 1:

pickGDP = 336800.0; //Central

break;

case 2:

pickGDP = 117200.0; //Northern

break;

case 3:

pickGDP = 151400.0; //Eastern

break;

case 4:

pickGDP = 282300.0; //Western

break;

case 5:

pickGDP = 222600.0; //Kampala

break;

default:

pickGDP = 222600.0; //rural Uganda as a whole

}

}

this.wealth = get_Main().startIncome(pickGDP, pickGini);

if(this.wealth <= get_Main().povertyLine) this.poor = true;

if(this.wealth > get_Main().cutoff4) this.quintile = 5;

else if(this.wealth > get_Main().cutoff3) this.quintile = 4;

else if(this.wealth > get_Main().cutoff2) this.quintile = 3;

else if(this.wealth > get_Main().cutoff1) this.quintile = 2;

else this.quintile = 1;

}

//make a kid

Person k = get_Main().add_persons(this.latitude, this.longitude, randomTrue(0.5), randomTrue(get_Main().propU5/get_Main().propKids) ? 0 : 1, this.urban, this.quintile, this.poor, false);

//latitude, longitude, male?, starting state (0 = U5, 1 = Youth, 2 = Adult), urban?, quintile, poor?, HOH?

k.latitude = k.latitude + uniform(-5/1.15/60/2,5/1.15/60/2);

k.longitude = k.longitude + + uniform(-5/1.15/60/2,5/1.15/60/2);

k.wealth = this.wealth;

k.jumpTo(k.longitude, k.latitude);

this.kids.connectTo(k);

k.mother.connectTo(this);

if(this.spouse.isConnected()) {

Person husband = this.spouse.getConnectedAgent();

husband.kids.connectTo(k);

k.father.connectTo(husband);

}

k.connections.disconnectFromAll();

for(Person c : this.connections.getConnections()) k.connections.connectTo(c);

if(k.spouse.isConnected()) k.spouse.disconnect();

//augment TFR and infant mortality rate

TFR = TFR + 1;

get_Main().liveBirths++;

//remove hospital connections

Collection<Agent> c = new LinkedList<Agent>();

for(Agent a : k.connections.getConnections()) if(get_Main().hospitals.contains(a) || get_Main().districts.contains(a) || get_Main().regionals.contains(a)) k.connections.disconnectFrom(a);

]]></Action>

<Timeout><![CDATA[1]]></Timeout>

<Condition><![CDATA[true]]></Condition>

<Rate><![CDATA[get_Main().fertility(this.age)/1.02]]></Rate>

<Guard><![CDATA[age < 51]]></Guard>

<MessageType><![CDATA[Object]]></MessageType>

<DefaultTransition>true</DefaultTransition>

<FilterType><![CDATA[unconditionally]]></FilterType>

<EqualsExpression><![CDATA["text"]]></EqualsExpression>

<SatisfiesExpression><![CDATA[true]]></SatisfiesExpression>

</Properties>

</StatechartElement>

<StatechartElement Class="Transition" ParentState="Male" ParentStateId="1391299880932">

<Id>1392854368249</Id>

<Name><![CDATA[doIMigrateM]]></Name>

<X>150</X><Y>350</Y>

<Label><X>10</X><Y>0</Y></Label>

<PublicFlag>false</PublicFlag>

<PresentationFlag>true</PresentationFlag>

<ShowLabel>false</ShowLabel>

<Points>

<Point><X>0</X><Y>0</Y></Point>

<Point><X>0</X><Y>20</Y></Point>

</Points>

<Properties Source="1391284865450" Target="1392854325945" Trigger="rate">

<Timeout><![CDATA[365]]></Timeout>

<Condition><![CDATA[true]]></Condition>

<Rate><![CDATA[1/365]]></Rate>

<MessageType><![CDATA[Object]]></MessageType>

<DefaultTransition>true</DefaultTransition>

<FilterType><![CDATA[unconditionally]]></FilterType>

<EqualsExpression><![CDATA["text"]]></EqualsExpression>

<SatisfiesExpression><![CDATA[true]]></SatisfiesExpression>

</Properties>

</StatechartElement>

<StatechartElement Class="Transition" ParentState="Male" ParentStateId="1391299880932">

<Id>1392854499979</Id>

<Name><![CDATA[migrateYesM]]></Name>

<X>150</X><Y>390</Y>

<Label><X>10</X><Y>0</Y></Label>

<PublicFlag>false</PublicFlag>

<PresentationFlag>true</PresentationFlag>

<ShowLabel>false</ShowLabel>

<Points>

<Point><X>0</X><Y>0</Y></Point>

<Point><X>0</X><Y>20</Y></Point>

</Points>

<Properties Source="1392854325945" Target="1392854410803" Trigger="timeout">

<Timeout><![CDATA[1]]></Timeout>

<Condition><![CDATA[randomTrue(0.053)]]></Condition>

<Rate><![CDATA[1]]></Rate>

<MessageType><![CDATA[Object]]></MessageType>

<DefaultTransition>false</DefaultTransition>

<FilterType><![CDATA[unconditionally]]></FilterType>

<EqualsExpression><![CDATA["text"]]></EqualsExpression>

<SatisfiesExpression><![CDATA[true]]></SatisfiesExpression>

</Properties>

</StatechartElement>

<StatechartElement Class="Transition" ParentState="Male" ParentStateId="1391299880932">

<Id>1392854539941</Id>

<Name><![CDATA[migrateNoM]]></Name>

<X>138</X><Y>380</Y>

<Label><X>10</X><Y>0</Y></Label>

<PublicFlag>false</PublicFlag>

<PresentationFlag>true</PresentationFlag>

<ShowLabel>false</ShowLabel>

<Points>

<Point><X>0</X><Y>0</Y></Point>

<Point><X>-18</X><Y>0</Y></Point>

<Point><X>-18</X><Y>-30</Y></Point>

</Points>

<Properties Source="1392854325945" Target="1391284865450" Trigger="timeout">

<Timeout><![CDATA[1]]></Timeout>

<Condition><![CDATA[true]]></Condition>

<Rate><![CDATA[1]]></Rate>

<MessageType><![CDATA[Object]]></MessageType>

<DefaultTransition>true</DefaultTransition>

<FilterType><![CDATA[unconditionally]]></FilterType>

<EqualsExpression><![CDATA["text"]]></EqualsExpression>

<SatisfiesExpression><![CDATA[true]]></SatisfiesExpression>

</Properties>

</StatechartElement>

<StatechartElement Class="Transition" ParentState="Male" ParentStateId="1391299880932">

<Id>1392854627213</Id>

<Name><![CDATA[stopMigrateM]]></Name>

<X>100</X><Y>430</Y>

<Label><X>10</X><Y>0</Y></Label>

<PublicFlag>false</PublicFlag>

<PresentationFlag>true</PresentationFlag>

<ShowLabel>false</ShowLabel>

<Points>

<Point><X>0</X><Y>0</Y></Point>

<Point><X>-10</X><Y>0</Y></Point>

<Point><X>-10</X><Y>-90</Y></Point>

<Point><X>0</X><Y>-90</Y></Point>

</Points>

<Properties Source="1392854410803" Target="1391284865450" Trigger="arrival">

<Action><![CDATA[

this.connections.disconnectFromAll();

Person locus = getNearestAgent(get_Main().persons);

if(this.distanceTo(locus) <= get_Main().placementRadius * 1609.34) {

for(Person connector : locus.connections.getConnections()) this.connections.connectTo(connector);

}

if(this.spouse.isConnected()) {

Person wife = this.spouse.getConnectedAgent();

wife.connections.disconnectFromAll();

for(Person d : this.connections.getConnections()) wife.connections.connectTo(d);

}

for(Person k : this.kids.getConnections()) {

k.connections.disconnectFromAll();

for(Person e : this.connections.getConnections()) k.connections.connectTo(e);

}

]]></Action>

<Timeout><![CDATA[1]]></Timeout>

<Condition><![CDATA[true]]></Condition>

<Rate><![CDATA[1]]></Rate>

<MessageType><![CDATA[Object]]></MessageType>

<DefaultTransition>true</DefaultTransition>

<FilterType><![CDATA[unconditionally]]></FilterType>

<EqualsExpression><![CDATA["text"]]></EqualsExpression>

<SatisfiesExpression><![CDATA[true]]></SatisfiesExpression>

</Properties>

</StatechartElement>

<StatechartElement Class="Transition" ParentState="Female" ParentStateId="1391567760449">

<Id>1392912246955</Id>

<Name><![CDATA[doIMigrateF]]></Name>

<X>740</X><Y>400</Y>

<Label><X>10</X><Y>0</Y></Label>

<PublicFlag>false</PublicFlag>

<PresentationFlag>true</PresentationFlag>

<ShowLabel>false</ShowLabel>

<Points>

<Point><X>0</X><Y>0</Y></Point>

<Point><X>0</X><Y>20</Y></Point>

</Points>

<Properties Source="1391567760454" Target="1392912246951" Trigger="rate">

<Timeout><![CDATA[365]]></Timeout>

<Condition><![CDATA[true]]></Condition>

<Rate><![CDATA[1/365]]></Rate>

<MessageType><![CDATA[Object]]></MessageType>

<DefaultTransition>true</DefaultTransition>

<FilterType><![CDATA[unconditionally]]></FilterType>

<EqualsExpression><![CDATA["text"]]></EqualsExpression>

<SatisfiesExpression><![CDATA[true]]></SatisfiesExpression>

</Properties>

</StatechartElement>

<StatechartElement Class="Transition" ParentState="Female" ParentStateId="1391567760449">

<Id>1392912246957</Id>

<Name><![CDATA[migrateYesF]]></Name>

<X>740</X><Y>440</Y>

<Label><X>10</X><Y>0</Y></Label>

<PublicFlag>false</PublicFlag>

<PresentationFlag>true</PresentationFlag>

<ShowLabel>false</ShowLabel>

<Points>

<Point><X>0</X><Y>0</Y></Point>

<Point><X>0</X><Y>20</Y></Point>

</Points>

<Properties Source="1392912246951" Target="1392912246953" Trigger="timeout">

<Timeout><![CDATA[1]]></Timeout>

<Condition><![CDATA[randomTrue(0.053)]]></Condition>

<Rate><![CDATA[1]]></Rate>

<MessageType><![CDATA[Object]]></MessageType>

<DefaultTransition>false</DefaultTransition>

<FilterType><![CDATA[unconditionally]]></FilterType>

<EqualsExpression><![CDATA["text"]]></EqualsExpression>

<SatisfiesExpression><![CDATA[true]]></SatisfiesExpression>

</Properties>

</StatechartElement>

<StatechartElement Class="Transition" ParentState="Female" ParentStateId="1391567760449">

<Id>1392912246959</Id>

<Name><![CDATA[migrateNoF]]></Name>

<X>728</X><Y>430</Y>

<Label><X>10</X><Y>0</Y></Label>

<PublicFlag>false</PublicFlag>

<PresentationFlag>true</PresentationFlag>

<ShowLabel>false</ShowLabel>

<Points>

<Point><X>0</X><Y>0</Y></Point>

<Point><X>-18</X><Y>0</Y></Point>

<Point><X>-18</X><Y>-30</Y></Point>

</Points>

<Properties Source="1392912246951" Target="1391567760454" Trigger="timeout">

<Timeout><![CDATA[1]]></Timeout>

<Condition><![CDATA[true]]></Condition>

<Rate><![CDATA[1]]></Rate>

<MessageType><![CDATA[Object]]></MessageType>

<DefaultTransition>true</DefaultTransition>

<FilterType><![CDATA[unconditionally]]></FilterType>

<EqualsExpression><![CDATA["text"]]></EqualsExpression>

<SatisfiesExpression><![CDATA[true]]></SatisfiesExpression>

</Properties>

</StatechartElement>

<StatechartElement Class="Transition" ParentState="Female" ParentStateId="1391567760449">

<Id>1392912246961</Id>

<Name><![CDATA[stopMigrateF]]></Name>

<X>690</X><Y>480</Y>

<Label><X>10</X><Y>0</Y></Label>

<PublicFlag>false</PublicFlag>

<PresentationFlag>true</PresentationFlag>

<ShowLabel>false</ShowLabel>

<Points>

<Point><X>0</X><Y>0</Y></Point>

<Point><X>-10</X><Y>0</Y></Point>

<Point><X>-10</X><Y>-90</Y></Point>

<Point><X>0</X><Y>-90</Y></Point>

</Points>

<Properties Source="1392912246953" Target="1391567760454" Trigger="arrival">

<Action><![CDATA[

this.connections.disconnectFromAll();

Person locus = getNearestAgent(get_Main().persons);

if(this.distanceTo(locus) <= get_Main().placementRadius * 1609.34) {

for(Person connector : locus.connections.getConnections()) this.connections.connectTo(connector);

}

if(this.spouse.isConnected()) {

Person husband = this.spouse.getConnectedAgent();

husband.connections.disconnectFromAll();

for(Person d : this.connections.getConnections()) husband.connections.connectTo(d);

}

for(Person k : this.kids.getConnections()) {

k.connections.disconnectFromAll();

for(Person e : this.connections.getConnections()) k.connections.connectTo(e);

}]]></Action>

<Timeout><![CDATA[1]]></Timeout>

<Condition><![CDATA[true]]></Condition>

<Rate><![CDATA[1]]></Rate>

<MessageType><![CDATA[Object]]></MessageType>

<DefaultTransition>true</DefaultTransition>

<FilterType><![CDATA[unconditionally]]></FilterType>

<EqualsExpression><![CDATA["text"]]></EqualsExpression>

<SatisfiesExpression><![CDATA[true]]></SatisfiesExpression>

</Properties>

</StatechartElement>

<StatechartElement Class="InitialStatePointer" ParentState="SickM" ParentStateId="1391286875465">

<Id>1393898850115</Id>

<Name><![CDATA[initialState3]]></Name>

<X>270</X><Y>340</Y>

<Label><X>10</X><Y>0</Y></Label>

<PublicFlag>false</PublicFlag>

<PresentationFlag>true</PresentationFlag>

<ShowLabel>false</ShowLabel>

<Points>

<Point><X>0</X><Y>0</Y></Point>

<Point><X>30</X><Y>0</Y></Point>

</Points>

<Properties Target="1393893169443">

<Action><![CDATA[

]]></Action>

</Properties>

</StatechartElement>

<StatechartElement Class="Transition" ParentState="Male" ParentStateId="1391299880932">

<Id>1393898905698</Id>

<Name><![CDATA[txSuccessM]]></Name>

<X>350</X><Y>540</Y>

<Label><X>-70</X><Y>5</Y></Label>

<PublicFlag>false</PublicFlag>

<PresentationFlag>true</PresentationFlag>

<ShowLabel>true</ShowLabel>

<Points>

<Point><X>0</X><Y>0</Y></Point>

<Point><X>0</X><Y>30</Y></Point>

<Point><X>108</X><Y>30</Y></Point>

</Points>

<Properties Source="1393898994969" Target="1412785465830" Trigger="timeout">

<Timeout><![CDATA[1]]></Timeout>

<Condition><![CDATA[true]]></Condition>

<Rate><![CDATA[1]]></Rate>

<MessageType><![CDATA[Object]]></MessageType>

<DefaultTransition>true</DefaultTransition>

<FilterType><![CDATA[unconditionally]]></FilterType>

<EqualsExpression><![CDATA["text"]]></EqualsExpression>

<SatisfiesExpression><![CDATA[true]]></SatisfiesExpression>

</Properties>

</StatechartElement>

<StatechartElement Class="InitialStatePointer" ParentState="RecoveredM" ParentStateId="1393893469132">

<Id>1393898917845</Id>

<Name><![CDATA[initialState4]]></Name>

<X>270</X><Y>610</Y>

<Label><X>10</X><Y>0</Y></Label>

<PublicFlag>false</PublicFlag>

<PresentationFlag>true</PresentationFlag>

<ShowLabel>false</ShowLabel>

<Points>

<Point><X>0</X><Y>0</Y></Point>

<Point><X>30</X><Y>0</Y></Point>

</Points>

<Properties Target="1393893571694">

</Properties>

</StatechartElement>

<StatechartElement Class="Transition" ParentState="RecoveredM" ParentStateId="1393893469132">

<Id>1393898931032</Id>

<Name><![CDATA[arriveHomeM]]></Name>

<X>350</X><Y>630</Y>

<Label><X>5</X><Y>20</Y></Label>

<PublicFlag>false</PublicFlag>

<PresentationFlag>true</PresentationFlag>

<ShowLabel>true</ShowLabel>

<Points>

<Point><X>0</X><Y>0</Y></Point>

<Point><X>0</X><Y>50</Y></Point>

</Points>

<Properties Source="1393893571694" Target="1393893581754" Trigger="arrival">

<Timeout><![CDATA[1]]></Timeout>

<Condition><![CDATA[true]]></Condition>

<Rate><![CDATA[1]]></Rate>

<MessageType><![CDATA[Object]]></MessageType>

<DefaultTransition>true</DefaultTransition>

<FilterType><![CDATA[unconditionally]]></FilterType>

<EqualsExpression><![CDATA["text"]]></EqualsExpression>

<SatisfiesExpression><![CDATA[true]]></SatisfiesExpression>

</Properties>

</StatechartElement>

<StatechartElement Class="Transition" ParentState="Male" ParentStateId="1391299880932">

<Id>1393898950577</Id>

<Name><![CDATA[arriveHospitalM]]></Name>

<X>560</X><Y>360</Y>

<Label><X>-140</X><Y>95</Y></Label>

<PublicFlag>false</PublicFlag>

<PresentationFlag>true</PresentationFlag>

<ShowLabel>true</ShowLabel>

<Points>

<Point><X>0</X><Y>0</Y></Point>

<Point><X>0</X><Y>110</Y></Point>

<Point><X>-110</X><Y>110</Y></Point>

<Point><X>-160</X><Y>110</Y></Point>

</Points>

<Properties Source="1393893207860" Target="1393893248502" Trigger="arrival">

<Action><![CDATA[double p = (double) 1/(1d+get_Main().meanTxLength);

this.txEndDate = geometric(p);]]></Action>

<Timeout><![CDATA[1]]></Timeout>

<Condition><![CDATA[true]]></Condition>

<Rate><![CDATA[1]]></Rate>

<MessageType><![CDATA[Object]]></MessageType>

<DefaultTransition>true</DefaultTransition>

<FilterType><![CDATA[unconditionally]]></FilterType>

<EqualsExpression><![CDATA["text"]]></EqualsExpression>

<SatisfiesExpression><![CDATA[true]]></SatisfiesExpression>

</Properties>

</StatechartElement>

<StatechartElement Class="Transition" ParentState="Male" ParentStateId="1391299880932">

<Id>1393899064373</Id>

<Name><![CDATA[txResultM]]></Name>

<Description><![CDATA[Assumes that treatment takes on average 2 weeks]]></Description>

<X>350</X><Y>480</Y>

<Label><X>10</X><Y>0</Y></Label>

<PublicFlag>false</PublicFlag>

<PresentationFlag>true</PresentationFlag>

<ShowLabel>false</ShowLabel>

<Points>

<Point><X>0</X><Y>0</Y></Point>

<Point><X>0</X><Y>15</Y></Point>

<Point><X>0</X><Y>40</Y></Point>

</Points>

<Properties Source="1393893248502" Target="1393898994969" Trigger="timeout">

<Action><![CDATA[//get the hospital type and the mortality multiplier

double a = 1;

double qual = 0;

double twoweek = 0;

boolean NGO = false;

if (this.chosenHospital instanceof Regional) a = get_Main().mRegMort * 20; //if regional, get regional multiplier

else if(this.chosenHospital instanceof District) a = get_Main().mDistMort * 5; //if district, get district multiplier

else {

qual = ((Hospital) this.chosenHospital).quality; //if Hospital, determine hospital quality (quality increases above 5.9 with the NGOs

twoweek = ((Hospital) this.chosenHospital).twoWeek; //determine if a two-week trip is present

NGO = ((Hospital) this.chosenHospital).NGO;

}

if(NGO) { //if a hospital is an NGO, determine whether it's a two-week trip or not

double r = beta(20, 5); //mean = 0.8

double t = get_Main().mMortMercyShips *(1.0 - twoweek) + get_Main().mMortTwoWeek * twoweek; //determines what my mortality rate SHOULD be

a = r * t / 0.8; //will return a value that is, on average, t (since, on average, r = 0.8)

}

//this.pDieTx = beta(10*a*get_Main().periopMortality, 10*(1.0 - a*get_Main().periopMortality));

if (this.chosenHospital instanceof Regional) this.pDieTx = beta(5,5);

else if(this.chosenHospital instanceof District) this.pDieTx = beta(5,5);

/* Note that this is not just the immediate post-op mortality, but also encompasses the inability of some places to treat the cancer */]]></Action>

<Timeout><![CDATA[txEndDate]]></Timeout>

<Condition><![CDATA[true]]></Condition>

<Rate><![CDATA[1/14 //Assumes that treatment takes on average 2 weeks]]></Rate>

<MessageType><![CDATA[Object]]></MessageType>

<DefaultTransition>true</DefaultTransition>

<FilterType><![CDATA[unconditionally]]></FilterType>

<EqualsExpression><![CDATA["text"]]></EqualsExpression>

<SatisfiesExpression><![CDATA[true]]></SatisfiesExpression>

</Properties>

</StatechartElement>

<StatechartElement Class="Transition" ParentState="Male" ParentStateId="1391299880932">

<Id>1393899113968</Id>

<Name><![CDATA[txDeathM]]></Name>

<X>362</X><Y>530</Y>

<Label><X>58</X><Y>-10</Y></Label>

<PublicFlag>false</PublicFlag>

<PresentationFlag>true</PresentationFlag>

<ShowLabel>true</ShowLabel>

<Points>

<Point><X>0</X><Y>0</Y></Point>

<Point><X>68</X><Y>0</Y></Point>

<Point><X>68</X><Y>-80</Y></Point>

<Point><X>68</X><Y>-110</Y></Point>

</Points>

<Properties Source="1393898994969" Target="1410548098252" Trigger="timeout">

<Timeout><![CDATA[1]]></Timeout>

<Condition><![CDATA[randomTrue(this.pDieTx)]]></Condition>

<Rate><![CDATA[1]]></Rate>

<MessageType><![CDATA[Object]]></MessageType>

<DefaultTransition>false</DefaultTransition>

<FilterType><![CDATA[unconditionally]]></FilterType>

<EqualsExpression><![CDATA["text"]]></EqualsExpression>

<SatisfiesExpression><![CDATA[true]]></SatisfiesExpression>

</Properties>

</StatechartElement>

<StatechartElement Class="Transition" ParentState="SickM" ParentStateId="1391286875465">

<Id>1393900038994</Id>

<Name><![CDATA[seekCareM]]></Name>

<X>360</X><Y>340</Y>

<Label><X>0</X><Y>-15</Y></Label>

<PublicFlag>false</PublicFlag>

<PresentationFlag>true</PresentationFlag>

<ShowLabel>true</ShowLabel>

<Points>

<Point><X>0</X><Y>0</Y></Point>

<Point><X>48</X><Y>0</Y></Point>

</Points>

<Properties Source="1393893169443" Target="1410547993448" Trigger="timeout">

<Action><![CDATA[seekCare();

]]></Action>

<Timeout><![CDATA[0]]></Timeout>

<Condition><![CDATA[//Logit model for whether healthcare is sought, after Lavy and Germain 1994

]]></Condition>

<Rate><![CDATA[1]]></Rate>

<MessageType><![CDATA[Object]]></MessageType>

<DefaultTransition>true</DefaultTransition>

<FilterType><![CDATA[unconditionally]]></FilterType>

<EqualsExpression><![CDATA["text"]]></EqualsExpression>

<SatisfiesExpression><![CDATA[true]]></SatisfiesExpression>

</Properties>

</StatechartElement>

<StatechartElement Class="InitialStatePointer" ParentState="SickF" ParentStateId="1391567760452">

<Id>1394059475291</Id>

<Name><![CDATA[initialState5]]></Name>

<X>860</X><Y>340</Y>

<Label><X>10</X><Y>0</Y></Label>

<PublicFlag>false</PublicFlag>

<PresentationFlag>true</PresentationFlag>

<ShowLabel>false</ShowLabel>

<Points>

<Point><X>0</X><Y>0</Y></Point>

<Point><X>30</X><Y>0</Y></Point>

</Points>

<Properties Target="1394059475276">

</Properties>

</StatechartElement>

<StatechartElement Class="Transition" ParentState="Female" ParentStateId="1391567760449">

<Id>1394059475293</Id>

<Name><![CDATA[txSuccessF]]></Name>

<X>940</X><Y>500</Y>

<Label><X>-65</X><Y>10</Y></Label>

<PublicFlag>false</PublicFlag>

<PresentationFlag>true</PresentationFlag>

<ShowLabel>true</ShowLabel>

<Points>

<Point><X>0</X><Y>0</Y></Point>

<Point><X>0</X><Y>40</Y></Point>

<Point><X>108</X><Y>40</Y></Point>

</Points>

<Properties Source="1394059475289" Target="1412786094778" Trigger="timeout">

<Timeout><![CDATA[1]]></Timeout>

<Condition><![CDATA[true]]></Condition>

<Rate><![CDATA[1]]></Rate>

<MessageType><![CDATA[Object]]></MessageType>

<DefaultTransition>true</DefaultTransition>

<FilterType><![CDATA[unconditionally]]></FilterType>

<EqualsExpression><![CDATA["text"]]></EqualsExpression>

<SatisfiesExpression><![CDATA[true]]></SatisfiesExpression>

</Properties>

</StatechartElement>

<StatechartElement Class="InitialStatePointer" ParentState="RecoveredF" ParentStateId="1394059475283">

<Id>1394059475295</Id>

<Name><![CDATA[initialState6]]></Name>

<X>860</X><Y>600</Y>

<Label><X>10</X><Y>0</Y></Label>

<PublicFlag>false</PublicFlag>

<PresentationFlag>true</PresentationFlag>

<ShowLabel>false</ShowLabel>

<Points>

<Point><X>0</X><Y>0</Y></Point>

<Point><X>30</X><Y>0</Y></Point>

</Points>

<Properties Target="1394059475285">

</Properties>

</StatechartElement>

<StatechartElement Class="Transition" ParentState="RecoveredF" ParentStateId="1394059475283">

<Id>1394059475297</Id>

<Name><![CDATA[arriveHomeF]]></Name>

<X>940</X><Y>620</Y>

<Label><X>5</X><Y>20</Y></Label>

<PublicFlag>false</PublicFlag>

<PresentationFlag>true</PresentationFlag>

<ShowLabel>true</ShowLabel>

<Points>

<Point><X>0</X><Y>0</Y></Point>

<Point><X>0</X><Y>50</Y></Point>

</Points>

<Properties Source="1394059475285" Target="1394059475287" Trigger="arrival">

<Timeout><![CDATA[1]]></Timeout>

<Condition><![CDATA[true]]></Condition>

<Rate><![CDATA[1]]></Rate>

<MessageType><![CDATA[Object]]></MessageType>

<DefaultTransition>true</DefaultTransition>

<FilterType><![CDATA[unconditionally]]></FilterType>

<EqualsExpression><![CDATA["text"]]></EqualsExpression>

<SatisfiesExpression><![CDATA[true]]></SatisfiesExpression>

</Properties>

</StatechartElement>

<StatechartElement Class="Transition" ParentState="Female" ParentStateId="1391567760449">

<Id>1394059475299</Id>

<Name><![CDATA[arriveHospitalF]]></Name>

<X>1110</X><Y>360</Y>

<Label><X>-105</X><Y>75</Y></Label>

<PublicFlag>false</PublicFlag>

<PresentationFlag>true</PresentationFlag>

<ShowLabel>true</ShowLabel>

<Points>

<Point><X>0</X><Y>0</Y></Point>

<Point><X>0</X><Y>90</Y></Point>

<Point><X>-80</X><Y>90</Y></Point>

<Point><X>-120</X><Y>90</Y></Point>

</Points>

<Properties Source="1394059475279" Target="1394059475281" Trigger="arrival">

<Action><![CDATA[double p = (double) 1d/(1d+get_Main().meanTxLength);

this.txEndDate = geometric(p);]]></Action>

<Timeout><![CDATA[1]]></Timeout>

<Condition><![CDATA[true]]></Condition>

<Rate><![CDATA[1]]></Rate>

<MessageType><![CDATA[Object]]></MessageType>

<DefaultTransition>true</DefaultTransition>

<FilterType><![CDATA[unconditionally]]></FilterType>

<EqualsExpression><![CDATA["text"]]></EqualsExpression>

<SatisfiesExpression><![CDATA[true]]></SatisfiesExpression>

</Properties>

</StatechartElement>

<StatechartElement Class="Transition" ParentState="Female" ParentStateId="1391567760449">

<Id>1394059475301</Id>

<Name><![CDATA[txResultF]]></Name>

<Description><![CDATA[Assumes that treatment takes on average 14 days]]></Description>

<X>940</X><Y>460</Y>

<Label><X>10</X><Y>0</Y></Label>

<PublicFlag>false</PublicFlag>

<PresentationFlag>true</PresentationFlag>

<ShowLabel>false</ShowLabel>

<Points>

<Point><X>0</X><Y>0</Y></Point>

<Point><X>0</X><Y>20</Y></Point>

</Points>

<Properties Source="1394059475281" Target="1394059475289" Trigger="timeout">

<Action><![CDATA[//get the hospital type and the mortality multiplier

double a = 1;

double qual = 0;

double twoweek = 0;

boolean NGO = false;

if (this.chosenHospital instanceof Regional) a = get_Main().mRegMort * 20; //if regional, get regional multiplier

else if(this.chosenHospital instanceof District) a = get_Main().mDistMort * 5; //if district, get district multiplier

else {

qual = ((Hospital) this.chosenHospital).quality; //if Hospital, determine hospital quality (quality increases above 5.9 with the NGOs

twoweek = ((Hospital) this.chosenHospital).twoWeek; //determine if a two-week trip is present

NGO = ((Hospital) this.chosenHospital).NGO;

}

if(NGO) { //if a hospital is an NGO, determine whether it's a two-week trip or not

double r = beta(20, 5); //mean = 0.8

double t = get_Main().mMortMercyShips *(1.0 - twoweek) + get_Main().mMortTwoWeek * twoweek; //determines what my mortality rate SHOULD be

a = r * t / 0.8; //will return a value that is, on average, t (since, on average, r = 0.8)

}

//this.pDieTx = beta(10*a*get_Main().periopMortality, 10*(1.0 - a*get_Main().periopMortality));

if (this.chosenHospital instanceof Regional) this.pDieTx = beta(5,5);

else if(this.chosenHospital instanceof District) this.pDieTx = beta(5,5);

/* Note that this is not just the immediate post-op mortality, but also encompasses the inability of some places to treat the cancer */]]></Action>

<Timeout><![CDATA[txEndDate]]></Timeout>

<Condition><![CDATA[true]]></Condition>

<Rate><![CDATA[1/14]]></Rate>

<MessageType><![CDATA[Object]]></MessageType>

<DefaultTransition>true</DefaultTransition>

<FilterType><![CDATA[unconditionally]]></FilterType>

<EqualsExpression><![CDATA["text"]]></EqualsExpression>

<SatisfiesExpression><![CDATA[true]]></SatisfiesExpression>

</Properties>

</StatechartElement>

<StatechartElement Class="Transition" ParentState="Female" ParentStateId="1391567760449">

<Id>1394059475303</Id>

<Name><![CDATA[txDeathF]]></Name>

<X>952</X><Y>490</Y>

<Label><X>43</X><Y>-10</Y></Label>

<PublicFlag>false</PublicFlag>

<PresentationFlag>true</PresentationFlag>

<ShowLabel>true</ShowLabel>

<Points>

<Point><X>0</X><Y>0</Y></Point>

<Point><X>68</X><Y>0</Y></Point>

<Point><X>68</X><Y>-60</Y></Point>

<Point><X>68</X><Y>-90</Y></Point>

</Points>

<Properties Source="1394059475289" Target="1410550032673" Trigger="timeout">

<Timeout><![CDATA[1]]></Timeout>

<Condition><![CDATA[randomTrue(this.pDieTx)]]></Condition>

<Rate><![CDATA[1]]></Rate>

<MessageType><![CDATA[Object]]></MessageType>

<DefaultTransition>false</DefaultTransition>

<FilterType><![CDATA[unconditionally]]></FilterType>

<EqualsExpression><![CDATA["text"]]></EqualsExpression>

<SatisfiesExpression><![CDATA[true]]></SatisfiesExpression>

</Properties>

</StatechartElement>

<StatechartElement Class="Transition" ParentState="SickF" ParentStateId="1391567760452">

<Id>1394059475305</Id>

<Name><![CDATA[seekCareF]]></Name>

<X>940</X><Y>340</Y>

<Label><X>0</X><Y>-10</Y></Label>

<PublicFlag>false</PublicFlag>

<PresentationFlag>true</PresentationFlag>

<ShowLabel>true</ShowLabel>

<Points>

<Point><X>0</X><Y>0</Y></Point>

<Point><X>48</X><Y>0</Y></Point>

</Points>

<Properties Source="1394059475276" Target="1410550026703" Trigger="timeout">

<Action><![CDATA[seekCare();]]></Action>

<Timeout><![CDATA[0]]></Timeout>

<Condition><![CDATA[true]]></Condition>

<Rate><![CDATA[1]]></Rate>

<MessageType><![CDATA[Object]]></MessageType>

<DefaultTransition>true</DefaultTransition>

<FilterType><![CDATA[unconditionally]]></FilterType>

<EqualsExpression><![CDATA["text"]]></EqualsExpression>

<SatisfiesExpression><![CDATA[true]]></SatisfiesExpression>

</Properties>

</StatechartElement>

<StatechartElement Class="Transition" ParentState="SickM" ParentStateId="1391286875465">

<Id>1410548069803</Id>

<Name><![CDATA[GoM]]></Name>

<X>432</X><Y>340</Y>

<Label><X>10</X><Y>0</Y></Label>

<PublicFlag>false</PublicFlag>

<PresentationFlag>true</PresentationFlag>

<ShowLabel>false</ShowLabel>

<Points>

<Point><X>0</X><Y>0</Y></Point>

<Point><X>78</X><Y>0</Y></Point>

</Points>

<Properties Source="1410547993448" Target="1393893207860" Trigger="timeout">

<Timeout><![CDATA[1]]></Timeout>

<Condition><![CDATA[careSought]]></Condition>

<Rate><![CDATA[1]]></Rate>

<MessageType><![CDATA[Object]]></MessageType>

<DefaultTransition>false</DefaultTransition>

<FilterType><![CDATA[unconditionally]]></FilterType>

<EqualsExpression><![CDATA["text"]]></EqualsExpression>

<SatisfiesExpression><![CDATA[true]]></SatisfiesExpression>

</Properties>

</StatechartElement>

<StatechartElement Class="Transition" ParentState="SickM" ParentStateId="1391286875465">

<Id>1410548112273</Id>

<Name><![CDATA[transition1]]></Name>

<X>420</X><Y>350</Y>

<Label><X>10</X><Y>0</Y></Label>

<PublicFlag>false</PublicFlag>

<PresentationFlag>true</PresentationFlag>

<ShowLabel>false</ShowLabel>

<Points>

<Point><X>0</X><Y>0</Y></Point>

<Point><X>0</X><Y>40</Y></Point>

</Points>

<Properties Source="1410547993448" Target="1410548098252" Trigger="timeout">

<Timeout><![CDATA[1]]></Timeout>

<Condition><![CDATA[hospIndex > 900]]></Condition>

<Rate><![CDATA[1]]></Rate>

<MessageType><![CDATA[Object]]></MessageType>

<DefaultTransition>true</DefaultTransition>

<FilterType><![CDATA[unconditionally]]></FilterType>

<EqualsExpression><![CDATA["text"]]></EqualsExpression>

<SatisfiesExpression><![CDATA[true]]></SatisfiesExpression>

</Properties>

</StatechartElement>

<StatechartElement Class="Transition" ParentState="SickF" ParentStateId="1391567760452">

<Id>1410550042909</Id>

<Name><![CDATA[transition2]]></Name>

<X>1000</X><Y>340</Y>

<Label><X>10</X><Y>0</Y></Label>

<PublicFlag>false</PublicFlag>

<PresentationFlag>true</PresentationFlag>

<ShowLabel>false</ShowLabel>

<Points>

<Point><X>0</X><Y>10</Y></Point>

<Point><X>0</X><Y>30</Y></Point>

</Points>

<Properties Source="1410550026703" Target="1410550032673" Trigger="timeout">

<Timeout><![CDATA[1]]></Timeout>

<Condition><![CDATA[hospIndex > 900]]></Condition>

<Rate><![CDATA[1]]></Rate>

<MessageType><![CDATA[Object]]></MessageType>

<DefaultTransition>true</DefaultTransition>

<FilterType><![CDATA[unconditionally]]></FilterType>

<EqualsExpression><![CDATA["text"]]></EqualsExpression>

<SatisfiesExpression><![CDATA[true]]></SatisfiesExpression>

</Properties>

</StatechartElement>

<StatechartElement Class="Transition" ParentState="SickF" ParentStateId="1391567760452">

<Id>1410550060662</Id>

<Name><![CDATA[transition3]]></Name>

<X>1012</X><Y>340</Y>

<Label><X>10</X><Y>0</Y></Label>

<PublicFlag>false</PublicFlag>

<PresentationFlag>true</PresentationFlag>

<ShowLabel>false</ShowLabel>

<Points>

<Point><X>0</X><Y>0</Y></Point>

<Point><X>48</X><Y>0</Y></Point>

</Points>

<Properties Source="1410550026703" Target="1394059475279" Trigger="timeout">

<Timeout><![CDATA[1]]></Timeout>

<Condition><![CDATA[careSought]]></Condition>

<Rate><![CDATA[1]]></Rate>

<MessageType><![CDATA[Object]]></MessageType>

<DefaultTransition>false</DefaultTransition>

<FilterType><![CDATA[unconditionally]]></FilterType>

<EqualsExpression><![CDATA["text"]]></EqualsExpression>

<SatisfiesExpression><![CDATA[true]]></SatisfiesExpression>

</Properties>

</StatechartElement>

<StatechartElement Class="Transition" ParentState="Male" ParentStateId="1391299880932">

<Id>1412785516301</Id>

<Name><![CDATA[transition4]]></Name>

<X>470</X><Y>580</Y>

<Label><X>10</X><Y>0</Y></Label>

<PublicFlag>false</PublicFlag>

<PresentationFlag>true</PresentationFlag>

<ShowLabel>false</ShowLabel>

<Points>

<Point><X>0</X><Y>0</Y></Point>

<Point><X>0</X><Y>80</Y></Point>

<Point><X>-30</X><Y>80</Y></Point>

</Points>

<Properties Source="1412785465830" Target="1393893469132" Trigger="timeout">

<Timeout><![CDATA[1]]></Timeout>

<Condition><![CDATA[true]]></Condition>

<Rate><![CDATA[1]]></Rate>

<MessageType><![CDATA[Object]]></MessageType>

<DefaultTransition>true</DefaultTransition>

<FilterType><![CDATA[unconditionally]]></FilterType>

<EqualsExpression><![CDATA["text"]]></EqualsExpression>

<SatisfiesExpression><![CDATA[true]]></SatisfiesExpression>

</Properties>

</StatechartElement>

<StatechartElement Class="Transition" ParentState="Male" ParentStateId="1391299880932">

<Id>1412785557713</Id>

<Name><![CDATA[transition5]]></Name>

<X>482</X><Y>570</Y>

<Label><X>10</X><Y>0</Y></Label>

<PublicFlag>false</PublicFlag>

<PresentationFlag>true</PresentationFlag>

<ShowLabel>false</ShowLabel>

<Points>

<Point><X>0</X><Y>0</Y></Point>

<Point><X>118</X><Y>0</Y></Point>

<Point><X>118</X><Y>-32</Y></Point>

</Points>

<Properties Source="1412785465830" Target="1391299968072" Trigger="timeout">

<Timeout><![CDATA[1]]></Timeout>

<Condition><![CDATA[dieCompl]]></Condition>

<Rate><![CDATA[1]]></Rate>

<MessageType><![CDATA[Object]]></MessageType>

<DefaultTransition>false</DefaultTransition>

<FilterType><![CDATA[unconditionally]]></FilterType>

<EqualsExpression><![CDATA["text"]]></EqualsExpression>

<SatisfiesExpression><![CDATA[true]]></SatisfiesExpression>

</Properties>

</StatechartElement>

<StatechartElement Class="Transition" ParentState="Female" ParentStateId="1391567760449">

<Id>1412786120980</Id>

<Name><![CDATA[transition6]]></Name>

<X>1060</X><Y>550</Y>

<Label><X>10</X><Y>0</Y></Label>

<PublicFlag>false</PublicFlag>

<PresentationFlag>true</PresentationFlag>

<ShowLabel>false</ShowLabel>

<Points>

<Point><X>0</X><Y>0</Y></Point>

<Point><X>0</X><Y>90</Y></Point>

<Point><X>-30</X><Y>90</Y></Point>

</Points>

<Properties Source="1412786094778" Target="1394059475283" Trigger="timeout">

<Timeout><![CDATA[1]]></Timeout>

<Condition><![CDATA[true]]></Condition>

<Rate><![CDATA[1]]></Rate>

<MessageType><![CDATA[Object]]></MessageType>

<DefaultTransition>true</DefaultTransition>

<FilterType><![CDATA[unconditionally]]></FilterType>

<EqualsExpression><![CDATA["text"]]></EqualsExpression>

<SatisfiesExpression><![CDATA[true]]></SatisfiesExpression>

</Properties>

</StatechartElement>

<StatechartElement Class="Transition" ParentState="Female" ParentStateId="1391567760449">

<Id>1412786141112</Id>

<Name><![CDATA[transition7]]></Name>

<X>1072</X><Y>540</Y>

<Label><X>10</X><Y>0</Y></Label>

<PublicFlag>false</PublicFlag>

<PresentationFlag>true</PresentationFlag>

<ShowLabel>false</ShowLabel>

<Points>

<Point><X>0</X><Y>0</Y></Point>

<Point><X>88</X><Y>0</Y></Point>

<Point><X>88</X><Y>-42</Y></Point>

</Points>

<Properties Source="1412786094778" Target="1391567760456" Trigger="timeout">

<Timeout><![CDATA[1]]></Timeout>

<Condition><![CDATA[dieCompl]]></Condition>

<Rate><![CDATA[1]]></Rate>

<MessageType><![CDATA[Object]]></MessageType>

<DefaultTransition>false</DefaultTransition>

<FilterType><![CDATA[unconditionally]]></FilterType>

<EqualsExpression><![CDATA["text"]]></EqualsExpression>

<SatisfiesExpression><![CDATA[true]]></SatisfiesExpression>

</Properties>

</StatechartElement>

</StatechartElements>

<Functions>

<Function AccessType="default" StaticFunction="false">

<ReturnModificator>RETURNS_VALUE</ReturnModificator>

<ReturnType><![CDATA[int]]></ReturnType>

<Id>1391291089746</Id>

<Name><![CDATA[ageFunc]]></Name>

<X>-80</X><Y>50</Y>

<Label><X>10</X><Y>0</Y></Label>

<PublicFlag>false</PublicFlag>

<PresentationFlag>false</PresentationFlag>

<ShowLabel>true</ShowLabel>

<Body><![CDATA[return (int) (getYear() - yearBorn);]]></Body>

</Function>

<Function AccessType="default" StaticFunction="false">

<ReturnModificator>RETURNS_VALUE</ReturnModificator>

<ReturnType><![CDATA[int]]></ReturnType>

<Id>1392670533650</Id>

<Name><![CDATA[deathDate]]></Name>

<Description><![CDATA[Calculate when the patient will die of other causes

STILL NEED TO BACK OUT CANCER MORTALITY]]></Description>

<X>-80</X><Y>70</Y>

<Label><X>10</X><Y>0</Y></Label>

<PublicFlag>false</PublicFlag>

<PresentationFlag>false</PresentationFlag>

<ShowLabel>true</ShowLabel>

<Body><![CDATA[//pick death date

int day = 1;

if(time() == 0) { //for starting population, pick based on age from the InitLifeTable

double p = male ? get_Main().maleInitLifeTable(age) : get_Main().femaleInitLifeTable(age);

day = geometric(p*get_Main().deathAdjust);

}

else {

double p = male ? get_Main().maleLifeTable(0) : get_Main().femaleLifeTable(0);

int draw = geometric(p*get_Main().deathAdjust);

if(draw <= 365) day = draw;

else {

day = day + 364;

p = male ? get_Main().maleLifeTable(1) : get_Main().femaleLifeTable(1);

draw = geometric(p*get_Main().deathAdjust);

if(draw <= 365.25 * 4) day = day + draw;

else {

day = day + 4 * 365;

deathPick: for(int a = 5; a < 101; a += 5) {

p = male ? get_Main().maleLifeTable(a) : get_Main().femaleLifeTable(a);

draw = (a != 100) ? geometric(p*get_Main().deathAdjust) : 1;

if(draw < 365.25 * 5) {

day = day + draw;

break deathPick;

}

else day = day + 365 * 5;

}

}

}

}

return day;]]></Body>

</Function>

<Function AccessType="default" StaticFunction="false">

<ReturnModificator>VOID</ReturnModificator>

<ReturnType><![CDATA[double]]></ReturnType>

<Id>1394135397387</Id>

<Name><![CDATA[seekCare]]></Name>

<X>-80</X><Y>30</Y>

<Label><X>10</X><Y>0</Y></Label>

<PublicFlag>false</PublicFlag>

<PresentationFlag>false</PresentationFlag>

<ShowLabel>true</ShowLabel>

<Body><![CDATA[/* Multinomial nested logit model for whether care is sought,

following Ssewanyana 2006 and McFadden 1977 */

/**

We assume nested multinomial logit, with two nests: No care, (Public or NGO). From Ssewanyana, sigma for the care nest = 0.348

The probability of an individual choosing not to get care is

Pn0 = exp(V[0]) / ( exp(V[0]) + ( exp(V[pub]/sigma) + exp(V[NGO]/sigma) )^sigma )

The denominator is the same for all choices. Therefore, the probability that individual n chooses care at a public hospital is

Pnpub = exp(V[pub]/sigma) * ( exp(V[pub]/sigma) + exp(V[NGO]/sigma) )^(sigma - 1) / _denom_

and

PnNGO = exp(V[NGO]/sigma) * ( exp(V[pub]/sigma) + exp(V[NGO]/sigma) )^(sigma - 1) / _denom_

Given that you choose to seek care, the probability of choosing a public facility reduces to

exp(V[pub]/sigma) / ( exp(V[pub]/sigma) + exp(V[NGO]/sigma) )

NOTE: when no NGOs exist, this reduces to a non-nested MNL model, and all the sigmas drop out:

Pn0 = exp(V[0]) / ( exp(V[0]) + exp(V[pub]) )

Pnpub = exp(V[pub]) / ( exp(V[0]) + exp(V[pub]) )

So, what this code will do is:

1) determine the cost, distance, quality for the nearest non-NGO hospital, and calculate V[pub]

2) determine the cost, distance, quality for the nearest NGO hospital, and calculate V[NGO]

3) determine whether NGOs exist

a) if not, calculate pNoCare and pCarePub, and set pCareNGO = 0

b) if so, calculate pNoCare, pCarePub, and pCareNGO

4) randomly assign the individual to NoCare, CarePub, and CareNGO

a) if CareNGO, and more than one NGO platform is selected, the actual NGO chosen will be completely at random

b) if CarePub, then a second logit model will be performed, assuming independence of all the possible hospitals, to choose where care is sought.

NOTE: step 4 may have to be rethought, now that the nearest hospital is being used.

**/

//Get Main calls

double shape = get_Main().urbanGini;

double OOP = get_Main().OOP;

double vMult = get_Main().vouchersMult; //0 if vouchers, 1 if not

boolean TS = get_Main().TS;

double xe = 1; //exchange rate (2007) shillings per dollar, PPP = 630.41.

//Get all hospitals and determine whether NGOs exist

provs.clear();

provs.addAll(get_Main().allHospitalsNGO);

int totalHosps = get_Main().allHospitals.size();

int totalHospsNGO = get_Main().allHospitalsNGO.size();

boolean boolNGO = (totalHospsNGO > totalHosps);

//GET COEFFICIENTS AND INDIVIDUAL CHARACTERISTICS

//Get coefficients

//Beta (Individual)

double[] indivPublicArray = new double[7]; //constant, male, age 22, age 50, urban, primary educ, secondary educ

double[] indivPrivateArray = new double[7];

indivPublicArray[0] = get_Main().betas.getCellNumericValue(1, 23, 3); //constant

indivPrivateArray[0] = get_Main().betas.getCellNumericValue(1, 23, 4);

indivPublicArray[1] = get_Main().betas.getCellNumericValue(1, 24, 3); //male

indivPrivateArray[1] = get_Main().betas.getCellNumericValue(1, 24, 4);

indivPublicArray[2] = get_Main().betas.getCellNumericValue(1, 11, 3); //age 22

indivPrivateArray[2] = get_Main().betas.getCellNumericValue(1, 11, 4);

indivPublicArray[3] = get_Main().betas.getCellNumericValue(1, 12, 3); //age 50

indivPrivateArray[3] = get_Main().betas.getCellNumericValue(1, 12, 4);

indivPublicArray[4] = get_Main().betas.getCellNumericValue(1, 18, 3); //urban

indivPrivateArray[4] = get_Main().betas.getCellNumericValue(1, 18, 4);

indivPublicArray[5] = get_Main().betas.getCellNumericValue(1, 27, 3); //primary education

indivPrivateArray[5] = get_Main().betas.getCellNumericValue(1, 27, 4);

indivPublicArray[6] = get_Main().betas.getCellNumericValue(1, 28, 3); //secondary education

indivPrivateArray[6] = get_Main().betas.getCellNumericValue(1, 28, 4);

//Phi (Provider)

double distancePublic = get_Main().betas.getCellNumericValue(1, 7, 8);

double distancePrivate = get_Main().betas.getCellNumericValue(1, 7, 9);

//double nursesPublic = get_Main().betas.getCellNumericValue(5,8,8); //DATA NOT AVAILABLE

//double nursesPrivate = get_Main().betas.getCellNumericValue(5,8,9);

//double doctorsPublic = get_Main().betas.getCellNumericValue(5,9,8);

//double doctorsPrivate = get_Main().betas.getCellNumericValue(5,9,9);

double qualityPublic = get_Main().betas.getCellNumericValue(1,10,8) + get_Main().betas.getCellNumericValue(1,9,8); //this combines the prop_doctor and quality coefficients

double qualityPrivate = get_Main().betas.getCellNumericValue(1,10,9) + get_Main().betas.getCellNumericValue(1,9,9);

//Alpha (Income)

double alpha1Public = get_Main().betas.getCellNumericValue(1,7,13);

double alpha1Private = get_Main().betas.getCellNumericValue(1,7,14);

double alpha2Public = get_Main().betas.getCellNumericValue(1,8,13);

double alpha2Private = get_Main().betas.getCellNumericValue(1,8,14);

//Sigma (correlation)

double sigma = get_Main().betas.getCellNumericValue(1,1,12);

//Individual characteristics

double gender = this.male ? 1.0 : 0.0;

double age22 = (this.age < 50) ? 1.0 : 0.0;

age22 = (this.age < 22) ? 0.0 : age22;

double age50 = (this.age >= 50) ? 1.0 : 0.0;

double urb = this.urban ? 1.0 : 0.0;

double inc = (this.wealth + 1) * xe; //prevents zero income, which will error out below

double primary = (double) this.educPrimary;

double secondary = (double) this.educSecondary;

//Get hospitals and populate their data into a matrix

int[] priv = new int[totalHospsNGO]; //indices = hospitals;

double[] quality = new double[totalHospsNGO];

double[] distance = new double[totalHospsNGO];

double[] costarray = new double[totalHospsNGO];

double[] utility = new double[totalHospsNGO];

double[] cdf = new double[totalHospsNGO];

double[] NGO = new double[totalHospsNGO];

double[] indx = new double[totalHospsNGO];

String[] hName = new String[totalHospsNGO];

for(Agent h : provs){

if(h instanceof Hospital) {

int i = provs.indexOf(h);

priv[i] = ((Hospital) h).priv;

quality[i] = ((Hospital) h).quality;

distance[i] = this.distanceTo(((Hospital) h))/1000; //distance is expressed in meters and must be converted to km

costarray[i] = OOP * gamma(shape, ((Hospital) h).cost/shape + 0.0001) * xe + vMult * gamma(shape, ((Hospital) h).nonmed/shape + 0.0001) * xe;

NGO[i] = ((Hospital) h).NGO ? 1.0 : 0.0;

indx[i] = i;

hName[i] = ((Hospital) h).name;

} else if(h instanceof Regional) {

int i = provs.indexOf(h);

priv[i] = ((Regional) h).priv;

quality[i] = TS ? ((Regional) h).quality + 0.27 : ((Regional) h).quality; //this adds a doctor to each hospital

distance[i] = this.distanceTo(((Regional) h))/1000; //distance is expressed in meters and must be converted to km

costarray[i] = OOP * gamma(shape, ((Regional) h).cost/shape + 0.0001) * xe + vMult * gamma(shape, ((Regional) h).nonmed/shape + 0.0001) * xe;

NGO[i] = ((Regional) h).NGO ? 1.0 : 0.0;

indx[i] = i;

hName[i] = ((Regional) h).name;

} else {

int i = provs.indexOf(h);

priv[i] = ((District) h).priv;

quality[i] = TS ? ((District) h).quality * 1.706 + 0.27 : ((District) h).quality; //this increases quality to the level of regionals and adds a doctor

distance[i] = this.distanceTo(((District) h))/1000; //distance is expressed in meters and must be converted to km

costarray[i] = OOP * gamma(shape, ((District) h).cost/shape) * xe + vMult * gamma(shape, ((District) h).nonmed/shape + 0.0001) * xe;

NGO[i] = ((District) h).NGO ? 1.0 : 0.0;

indx[i] = i;

hName[i] = ((District) h).name;

}

}

//get the distance, cost, and quality of the nearest non-NGO hospital

double sum = 1000000;

int nearestIndex = 0;

for(int i = 0; i < totalHospsNGO; i++) if(NGO[i] == 0) { //only add if not an NGO

if(distance[i] < sum) {

sum = distance[i];

nearestIndex = i;

}

}

double meanDistNoNGO = sum;

double meanQualNoNGO = quality[nearestIndex];

double meanCostNoNGO = costarray[nearestIndex];

//get the distance, cost, and quality of the nearest NGO hospital

sum = 1000000;

nearestIndex = 0;

for(int i = 0; i < totalHospsNGO; i++) if(NGO[i] == 1) { //only add if an NGO

if(distance[i] < sum) {

sum = distance[i];

nearestIndex = i;

}

}

double meanDistNGO = boolNGO ? sum : 1000000;

double meanQualNGO = boolNGO ? quality[nearestIndex] : 0;

double meanCostNGO = boolNGO ? costarray[nearestIndex] : 1000000;

//calculate V[pub]

double V_0 = 0.0;

double V_public = indivPublicArray[0] + alpha1Public * (-meanCostNoNGO/inc) - alpha2Public * (2*(meanCostNoNGO/inc)*log(inc)) +

distancePublic*meanDistNoNGO + qualityPublic*meanQualNoNGO + indivPublicArray[1]*gender + indivPublicArray[2]*age22 +

indivPublicArray[3]*age50 + indivPublicArray[4]*urb + indivPublicArray[5]*primary + indivPublicArray[6]*secondary;

//if NGOs exist, calculate V[NGO]

double V_ngo = 0.0;

if(boolNGO) V_ngo = indivPrivateArray[0] + alpha1Public * (-meanCostNGO/inc) - alpha2Private * (2*(meanCostNGO/inc)*log(inc)) +

distancePrivate*meanDistNGO + qualityPrivate*meanQualNGO + indivPrivateArray[1]*gender + indivPrivateArray[2]*age22 +

indivPrivateArray[3]*age50 + indivPrivateArray[4]*urb + indivPrivateArray[5]*primary + indivPrivateArray[6]*secondary;

//calculate probabilities of care

double pNoCare = 0.0;

double pCarePub = 0.0;

double pCareNGO = 0.0;

if(!boolNGO) { //when NGOs don't exist

double a = exp(V_0) / ( exp(V_0) + exp(V_public) );

pNoCare = beta(10.0 * a, 10.0 * (1.0 - a));

pCarePub = (1.0 - pNoCare);

//pCarePub = exp(V_public) / ( exp(V_0) + exp(V_public) );

} else {

double denom = exp(V_0) + pow((exp(V_public/sigma) + exp(V_ngo/sigma)),sigma);

double numer = pow((exp(V_public/sigma) + exp(V_ngo/sigma)), (sigma - 1.0));

double a = exp(V_public/sigma) * numer / denom;

double b = exp(V_ngo/sigma) * numer / denom;

double c = exp(V_0) / denom;

pNoCare = beta(10.0 * c, 10.0 * (1.0 - c));

pCarePub = min(beta(10.0 * a, 10.0 * (1.0 - a)), 1.0 - pCarePub);

pCareNGO = max(0.0, 1.0 - pCarePub - pNoCare);

}

//determine if care is sought and where

double r = uniform();

String nameHosp = "—";

if(r < pNoCare ) {

careSought = false;

this.chosenHospital = null;

} else if (r < pNoCare + pCareNGO) {

careSought = true;

NGOs.clear();

NGOs.addAll(get_Main().allHospitalsNGO);

NGOs.removeAll(get_Main().allHospitals);

switch(NGOs.size()) {

case 0:

error("No NGOs. seekCare()");

break;

case 1:

this.chosenHospital = NGOs.get(0);

get_Main().ngoSurgeries++;

break;

case 2:

if(randomTrue(0.5)) this.chosenHospital = NGOs.get(0);

else this.chosenHospital = NGOs.get(1);

get_Main().ngoSurgeries++;

break;

case 3:

if(randomTrue(0.33333)) this.chosenHospital = NGOs.get(0);

else {

if(randomTrue(0.5)) this.chosenHospital = NGOs.get(1);

else this.chosenHospital = NGOs.get(2);

}

get_Main().ngoSurgeries++;

break;

default:

error("Too many NGOs. seekCare()");

break;

}

Hospital x = (Hospital) this.chosenHospital;

this.destLong = ((Hospital) x).longitude;

this.destLat = ((Hospital) x).latitude;

this.systCost = ((Hospital) x).cost * (1.0 - OOP) + (1.0 - vMult) * ((Hospital) x).nonmed;

this.persCost = get_Main().vouchersMult * ((Hospital) x).nonmed + ((Hospital) x).cost * OOP;

nameHosp = x.name;

} else {

careSought = true;

for(int i = 0; i < indx.length; i++){

double pub = 1.0 - (double) priv[i];

double qual = quality[i];

double dist = distance[i];

double cost = costarray[i];

double eV = Math.exp(indivPublicArray[0] + alpha1Public * (-cost/inc) - alpha2Public * (2*(cost/inc)*log(inc)) +

distancePublic*dist + qualityPublic*qual + indivPublicArray[1]*gender + indivPublicArray[2]*age22 +

indivPublicArray[3]*age50 + indivPublicArray[4]*urb + indivPublicArray[5]*primary + indivPublicArray[6]*secondary);

utility[i] = eV * (1.0 - NGO[i]) * 1E200 * pub;

}

this.denom = 0;

for(int row = 0; row < utility.length; row++) this.denom += utility[row];

for(int row = 0; row < utility.length; row++){

if(row == 0) {

cdf[row] = utility[row] / this.denom;

} else {

double a = max((utility[row] / this.denom), 1E-09); //prevents same-value rows in the distribution (errors java out)

cdf[row] = cdf[row - 1] + a;

}

}

//Choose where you go

hospChoice.setArgumentsAndValues(cdf, indx);

hospIndex = roundToInt(hospChoice.get(uniform()));

Agent x = provs.get(hospIndex);

if(x instanceof Hospital) {

this.destLong = ((Hospital) x).longitude;

this.destLat = ((Hospital) x).latitude;

this.systCost = ((Hospital) x).cost * (1.0 - OOP) + (1.0 - vMult) * ((Hospital) x).nonmed;

this.persCost = vMult * ((Hospital) x).nonmed + ((Hospital) x).cost * OOP;

this.chosenHospital = x;

nameHosp = ((Hospital) x).name;

} else if(x instanceof Regional) {

this.destLong = ((Regional) x).longitude;

this.destLat = ((Regional) x).latitude;

this.systCost = ((Regional) x).cost * (1.0 - OOP) + (1.0 - vMult) * ((Regional) x).nonmed;

this.persCost = vMult * ((Regional) x).nonmed + ((Regional) x).cost * OOP;

this.chosenHospital = x;

nameHosp = ((Regional) x).name;

} else {

this.destLong = ((District) x).longitude;

this.destLat = ((District) x).latitude;

this.systCost = ((District) x).cost * (1.0 - OOP) + (1.0 - vMult) * ((District) x).nonmed;

this.persCost = vMult * ((District) x).nonmed + ((District) x).cost * OOP;

this.chosenHospital = x;

nameHosp = ((District) x).name;

}

}

get_Main().persCost += this.persCost;

//System.out.println(boolNGO);

//System.out.println(indivPublicArray[0] + "\t"+ (-meanCostNoNGO/inc) +"\t"+ (2*(meanCostNoNGO/inc)*log(inc)) + "\t" + meanDistNoNGO + "\t" + meanQualNoNGO + "\t" + gender + "\t" + age22 +

// "\t" + age50 + "\t" + urb + "\t" + primary + "\t" + secondary);

//System.out.println(pCareNGO + "\t" + pCarePub + "\t" + pNoCare + "\t" + nameHosp + "\t" + meanQualNoNGO + "\t" + meanQualNGO + "\t" + meanCostNoNGO + "\t" + meanCostNGO + "\t" + meanDistNoNGO + "\t" + meanDistNGO + "\t" + this.wealth);

/*

System.out.println("Name\tUtility\tCalculated Pub\tCalculated Priv");

for(int i = 0; i < indx.length; i++) System.out.println(hName[i] + "\t" + utility[i] + "\t" + (Math.exp((1.0 - (double) priv[i])*(indivPublicArray[0] + alpha1Public * (-costarray[i]/inc) -

alpha2Public * (2*(costarray[i]/inc)*log(inc)) +

distancePublic*distance[i] + qualityPublic*quality[i] + indivPublicArray[1]*gender + indivPublicArray[2]*age22 +

indivPublicArray[3]*age50 + indivPublicArray[4]*urb + indivPublicArray[5]*primary + indivPublicArray[6]*secondary))) + "\t" +

(Math.exp(((double) priv[i])*(indivPublicArray[0] + alpha1Public * (-costarray[i]/inc) -

alpha2Public * (2*(costarray[i]/inc)*log(inc)) +

distancePublic*distance[i] + qualityPublic*quality[i] + indivPublicArray[1]*gender + indivPublicArray[2]*age22 +

indivPublicArray[3]*age50 + indivPublicArray[4]*urb + indivPublicArray[5]*primary + indivPublicArray[6]*secondary))));

*/

/*

System.out.println("Name\tNGO\tPub\tCost\tWealth\tDistance\tQuality\tgender\tage22\tage50\turban\tprimary\tsecondary\tAlpha1 term\tAlpha2 term\tDist term\tQual term\tGender Term\tAge22 term\tAge50 term\tUrb term\tPrimary term\tSecondary term\tAL sum\t\tAlpha1 term\tAlpha2 term\tDist term\tQual term\tGender Term\tAge22 term\tAge50 term\tUrb term\tPrimary term\tSecondary term\tAL sum\teV\teV with Private\tV Private\tUTILITY\tcdf");

for(int i = 0; i < indx.length; i++) System.out.println(hName[i] + "\t" + NGO[i] + "\t" + (1.0 - (double) priv[i]) + "\t" + costarray[i] + "\t" + inc + "\t" + distance[i] + "\t" +

quality[i] + "\t" + gender + "\t" + age22 + "\t" + age50 + "\t" + urb + "\t" + primary + "\t" + secondary + "\t" +

alpha1Public * (-costarray[i]/inc) + "\t" + alpha2Public * (2*(-costarray[i]/inc)*log(inc)) + "\t" +

distancePublic*distance[i] + "\t" + qualityPublic*quality[i] + "\t" + indivPublicArray[1]*gender + "\t" + indivPublicArray[2]*age22 +"\t" +

indivPublicArray[3]*age50 + "\t" + indivPublicArray[4]*urb + "\t" + indivPublicArray[5]*primary + "\t" + indivPublicArray[6]*secondary + "\t" +

(indivPublicArray[0] + alpha1Public * (-costarray[i]/inc) -

alpha2Public * (2*(costarray[i]/inc)*log(inc)) +

distancePublic*distance[i] + qualityPublic*quality[i] + indivPublicArray[1]*gender + indivPublicArray[2]*age22 +

indivPublicArray[3]*age50 + indivPublicArray[4]*urb + indivPublicArray[5]*primary + indivPublicArray[6]*secondary) + "\t" + "\t" +

alpha1Private * (-costarray[i]/inc) + "\t" + alpha2Private * (2*(-costarray[i]/inc)*log(inc)) + "\t" +

distancePrivate*distance[i] + "\t" + qualityPrivate*quality[i] + "\t" + indivPrivateArray[1]*gender + "\t" + indivPrivateArray[2]*age22 +"\t" +

indivPrivateArray[3]*age50 + "\t" + indivPrivateArray[4]*urb + "\t" + indivPrivateArray[5]*primary + "\t" + indivPrivateArray[6]*secondary + "\t" +

((double) priv[i] * (indivPrivateArray[0] + alpha1Private * (-costarray[i]/inc) -

alpha2Private * (2*(costarray[i]/inc)*log(inc)) +

distancePrivate*distance[i] + qualityPrivate*quality[i] + indivPrivateArray[1]*gender + indivPrivateArray[2]*age22 +

indivPrivateArray[3]*age50 + indivPrivateArray[4]*urb + indivPrivateArray[5]*primary + indivPrivateArray[6]*secondary)) + "\t" +

((1.0 - NGO[i]) * 1E200 * exp((1.0 - (double) priv[i]) * (indivPublicArray[0] + alpha1Public * (-costarray[i]/inc) -

alpha2Public * (2*(costarray[i]/inc)*log(inc)) +

distancePublic*distance[i] + qualityPublic*quality[i] + indivPublicArray[1]*gender + indivPublicArray[2]*age22 +

indivPublicArray[3]*age50 + indivPublicArray[4]*urb + indivPublicArray[5]*primary + indivPublicArray[6]*secondary))) + "\t" +

((1.0 - NGO[i]) * 1E200 * exp((1.0 - (double) priv[i]) * (indivPublicArray[0] + alpha1Public * (-costarray[i]/inc) -

alpha2Public * (2*(costarray[i]/inc)*log(inc)) +

distancePublic*distance[i] + qualityPublic*quality[i] + indivPublicArray[1]*gender + indivPublicArray[2]*age22 +

indivPublicArray[3]*age50 + indivPublicArray[4]*urb + indivPublicArray[5]*primary + indivPublicArray[6]*secondary) +

((double) priv[i])*(indivPrivateArray[0] + alpha1Private * (-costarray[i]/inc) - alpha2Private * (2*(costarray[i]/inc)*log(inc)) +

distancePrivate*distance[i] + qualityPrivate*quality[i] + indivPrivateArray[1]*gender + indivPrivateArray[2]*age22 +

indivPrivateArray[3]*age50 + indivPrivateArray[4]*urb + indivPrivateArray[5]*primary + indivPrivateArray[6]*secondary))) + "\t" +

(indivPrivateArray[0] + alpha1Private * (-costarray[i]/inc) - alpha2Private * (2*(costarray[i]/inc)*log(inc)) +

distancePrivate*distance[i] + qualityPrivate*quality[i] + indivPrivateArray[1]*gender + indivPrivateArray[2]*age22 +

indivPrivateArray[3]*age50 + indivPrivateArray[4]*urb + indivPrivateArray[5]*primary + indivPrivateArray[6]*secondary) + "\t" +

utility[i] + "\t" + cdf[i]);

*/

/*

System.out.println("Name\tNGO\tPub\tCost\tWealth\tDistance\tQuality\tgender\tage22\tage50\turban\tprimary\tsecondary\tAlpha1 term\tAlpha2 term\tDist term\tQual term\tGender Term\tAge22 term\tAge50 term\tUrb term\tPrimary term\tSecondary term\tAL sum\t\teV\tUTILITY\tcdf");

for(int i = 0; i < indx.length; i++) System.out.println(hName[i] + "\t" + NGO[i] + "\t" + (1.0 - (double) priv[i]) + "\t" + costarray[i] + "\t" + inc + "\t" + distance[i] + "\t" +

quality[i] + "\t" + gender + "\t" + age22 + "\t" + age50 + "\t" + urb + "\t" + primary + "\t" + secondary + "\t" +

alpha1Public * (-costarray[i]/inc) + "\t" + alpha2Public * (2*(-costarray[i]/inc)*log(inc)) + "\t" +

distancePublic*distance[i] + "\t" + qualityPublic*quality[i] + "\t" + indivPublicArray[1]*gender + "\t" + indivPublicArray[2]*age22 +"\t" +

indivPublicArray[3]*age50 + "\t" + indivPublicArray[4]*urb + "\t" + indivPublicArray[5]*primary + "\t" + indivPublicArray[6]*secondary + "\t" +

(indivPublicArray[0] + alpha1Public * (-costarray[i]/inc) -

alpha2Public * (2*(costarray[i]/inc)*log(inc)) +

distancePublic*distance[i] + qualityPublic*quality[i] + indivPublicArray[1]*gender + indivPublicArray[2]*age22 +

indivPublicArray[3]*age50 + indivPublicArray[4]*urb + indivPublicArray[5]*primary + indivPublicArray[6]*secondary) + "\t" + "\t" +

((1.0 - NGO[i]) * 1E200 * exp(indivPublicArray[0] + alpha1Public * (-costarray[i]/inc) - alpha2Public * (2*(costarray[i]/inc)*log(inc)) +

distancePublic*distance[i] + qualityPublic*quality[i] + indivPublicArray[1]*gender + indivPublicArray[2]*age22 +

indivPublicArray[3]*age50 + indivPublicArray[4]*urb + indivPublicArray[5]*primary + indivPublicArray[6]*secondary)) + "\t" +

utility[i] + "\t" + cdf[i]);

*/

/*

System.out.println("Constant\tAlpha1\tAlpha2\tDistance\tQuality\tGender\tAge22\tAge50\tUrban\tPrimary\tSecondary");

System.out.println(indivPrivateArray[0] + "\t" + alpha1Private + "\t" + alpha2Private + "\t" + distancePrivate + "\t" + qualityPrivate + "\t" + indivPrivateArray[1] + "\t" +

indivPrivateArray[2] + "\t" + indivPrivateArray[3] + "\t" + indivPrivateArray[4] + "\t" + indivPrivateArray[5] + "\t" + indivPrivateArray[6]);

*/

]]></Body>

</Function>

</Functions>

<TableFunctions>

<TableFunction AccessType="public" StaticFunction="true">

<Id>1410544752435</Id>

<Name><![CDATA[hospChoice]]></Name>

<X>-180</X><Y>300</Y>

<Label><X>10</X><Y>0</Y></Label>

<PublicFlag>false</PublicFlag>

<PresentationFlag>false</PresentationFlag>

<ShowLabel>true</ShowLabel>

<InterpolationMethod>STEP</InterpolationMethod>

<OutOfRangeBehaviour>NEAREST</OutOfRangeBehaviour>

<OutOfRangeCustomValue><![CDATA[0.0]]></OutOfRangeCustomValue>

<ApproximationOrder><![CDATA[1]]></ApproximationOrder>

// arguments and values

<Argument><![CDATA[0.0]]></Argument>

<Value><![CDATA[0.0]]></Value>

</TableFunction>

</TableFunctions>

<AgentLinks>

<AgentLink>

<Id>1392508625683</Id>

<Name><![CDATA[connections]]></Name>

<X>50</X><Y>-50</Y>

<Label><X>15</X><Y>0</Y></Label>

<PublicFlag>false</PublicFlag>

<PresentationFlag>true</PresentationFlag>

<ShowLabel>true</ShowLabel>

<HandleReceiveInConnections>false</HandleReceiveInConnections>

<AgentLinkType>COLLECTION_OF_LINKS</AgentLinkType>

<AgentReferenceId>1390777039087</AgentReferenceId>

<AgentLinkBidirectional>true</AgentLinkBidirectional>

<MessageType><![CDATA[Object]]></MessageType>

<StatechartNames>

<Name>statechart</Name>

</StatechartNames>

<LineStyle>SOLID</LineStyle>

<LineWidth>1</LineWidth>

<LineColor>1358954496</LineColor>

<LineZOrder>UNDER_AGENTS</LineZOrder>

<LineArrow>NONE</LineArrow>

<LineArrowPosition>END</LineArrowPosition>

</AgentLink>

<AgentLink>

<Id>1393014720110</Id>

<Name><![CDATA[father]]></Name>

<X>170</X><Y>-50</Y>

<Label><X>15</X><Y>0</Y></Label>

<PublicFlag>false</PublicFlag>

<PresentationFlag>true</PresentationFlag>

<ShowLabel>true</ShowLabel>

<HandleReceiveInConnections>false</HandleReceiveInConnections>

<AgentLinkType>SINGLE_LINK</AgentLinkType>

<AgentReferenceId>1390777039087</AgentReferenceId>

<AgentLinkBidirectional>false</AgentLinkBidirectional>

<MessageType><![CDATA[Object]]></MessageType>

<LineStyle>SOLID</LineStyle>

<LineWidth>1</LineWidth>

<LineColor>-16777216</LineColor>

<LineZOrder>UNDER_AGENTS</LineZOrder>

<LineArrow>NONE</LineArrow>

<LineArrowPosition>END</LineArrowPosition>

</AgentLink>

<AgentLink>

<Id>1393014811619</Id>

<Name><![CDATA[mother]]></Name>

<X>170</X><Y>-30</Y>

<Label><X>15</X><Y>0</Y></Label>

<PublicFlag>false</PublicFlag>

<PresentationFlag>true</PresentationFlag>

<ShowLabel>true</ShowLabel>

<HandleReceiveInConnections>false</HandleReceiveInConnections>

<AgentLinkType>SINGLE_LINK</AgentLinkType>

<AgentReferenceId>1390777039087</AgentReferenceId>

<AgentLinkBidirectional>false</AgentLinkBidirectional>

<MessageType><![CDATA[Object]]></MessageType>

<LineStyle>SOLID</LineStyle>

<LineWidth>1</LineWidth>

<LineColor>-16777216</LineColor>

<LineZOrder>UNDER_AGENTS</LineZOrder>

<LineArrow>NONE</LineArrow>

<LineArrowPosition>END</LineArrowPosition>

</AgentLink>

<AgentLink>

<Id>1393014824783</Id>

<Name><![CDATA[spouse]]></Name>

<X>170</X><Y>-70</Y>

<Label><X>15</X><Y>0</Y></Label>

<PublicFlag>false</PublicFlag>

<PresentationFlag>true</PresentationFlag>

<ShowLabel>true</ShowLabel>

<HandleReceiveInConnections>false</HandleReceiveInConnections>

<AgentLinkType>SINGLE_LINK</AgentLinkType>

<AgentReferenceId>1390777039087</AgentReferenceId>

<AgentLinkBidirectional>true</AgentLinkBidirectional>

<BidirectionalPeerId>1393014824783</BidirectionalPeerId>

<MessageType><![CDATA[Object]]></MessageType>

<LineStyle>SOLID</LineStyle>

<LineWidth>1</LineWidth>

<LineColor>-16777216</LineColor>

<LineZOrder>UNDER_AGENTS</LineZOrder>

<LineArrow>NONE</LineArrow>

<LineArrowPosition>END</LineArrowPosition>

</AgentLink>

<AgentLink>

<Id>1393014857459</Id>

<Name><![CDATA[kids]]></Name>

<X>250</X><Y>-70</Y>

<Label><X>15</X><Y>0</Y></Label>

<PublicFlag>false</PublicFlag>

<PresentationFlag>true</PresentationFlag>

<ShowLabel>true</ShowLabel>

<HandleReceiveInConnections>false</HandleReceiveInConnections>

<AgentLinkType>COLLECTION_OF_LINKS</AgentLinkType>

<AgentReferenceId>1390777039087</AgentReferenceId>

<AgentLinkBidirectional>false</AgentLinkBidirectional>

<MessageType><![CDATA[Object]]></MessageType>

<LineStyle>SOLID</LineStyle>

<LineWidth>1</LineWidth>

<LineColor>-8388608</LineColor>

<LineZOrder>UNDER_AGENTS</LineZOrder>

<LineArrow>NONE</LineArrow>

<LineArrowPosition>END</LineArrowPosition>

</AgentLink>

</AgentLinks>

<ContainerLinks>

<ContainerLink>

<Id>1392508627988</Id>

<Name><![CDATA[main]]></Name>

<X>50</X><Y>-100</Y>

<Label><X>10</X><Y>0</Y></Label>

<PublicFlag>false</PublicFlag>

<PresentationFlag>true</PresentationFlag>

<ShowLabel>true</ShowLabel>

<ActiveObjectClass>

<PackageName><![CDATA[_14_01_21_trial_uganda_model]]></PackageName>

<ClassName><![CDATA[Main]]></ClassName>

</ActiveObjectClass>

</ContainerLink>

</ContainerLinks>

<Shapes>

<Oval>

<Id>1390777097352</Id>

<Name><![CDATA[oval]]></Name>

<X>0</X><Y>0</Y>

<Label><X>0</X><Y>0</Y></Label>

<PublicFlag>true</PublicFlag>

<PresentationFlag>true</PresentationFlag>

<ShowLabel>false</ShowLabel>

<DrawMode>SHAPE_DRAW_2D</DrawMode>

<OnClickCode><![CDATA[if(get_Main().selectedPerson != this) {

get_Main().selectedPerson = this;

for(Person p : get_Main().persons) {

p.oval.setVisible(false);

p.connections.getLinkToAgentMyAnimationSettings().setVisible(false);

}

this.oval.setVisible(true);

this.connections.getLinkToAgentMyAnimationSettings().setVisible(true);

for(Person c : this.connections.getConnections()) {

c.oval.setVisible(true);

c.oval.setFillColor(gray);

}

if(this.spouse.isConnected()) {

this.spouse.getConnectedAgent().oval.setVisible(true);

this.spouse.getConnectedAgent().oval.setFillColor(purple);

}

if(this.father.isConnected()) {

this.father.getConnectedAgent().oval.setVisible(true);

this.father.getConnectedAgent().oval.setFillColor(black);

}

if(this.mother.isConnected()) {

this.mother.getConnectedAgent().oval.setVisible(true);

this.mother.getConnectedAgent().oval.setFillColor(pink);

}

for(Person k : this.kids.getConnections()) {

k.oval.setVisible(true);

k.oval.setFillColor(brown);

}

}

else {

get_Main().selectedPerson = null;

for(Person p : get_Main().persons) {

p.oval.setVisible(true);

p.connections.getLinkToAgentMyAnimationSettings().setVisible(true);

}

}

return true;]]></OnClickCode>

<AsObject>true</AsObject>

<EmbeddedIcon>false</EmbeddedIcon>

<Z>0</Z>

<ZHeight>10</ZHeight>

<LineWidth>1</LineWidth>

<LineColor/>

<LineMaterial>null</LineMaterial>

<LineStyle>SOLID</LineStyle>

<RadiusX>1</RadiusX>

<RadiusXCode><![CDATA[get_Main().selectedPerson == this ? 4 : (this.statechart.isStateActive(this.Male) || this.statechart.isStateActive(this.Female) ? 3 : 2)]]></RadiusXCode>

<RadiusY>1</RadiusY>

<RadiusYCode><![CDATA[get_Main().selectedPerson == this ? 2 : 1]]></RadiusYCode>

<Rotation>0.0</Rotation>

<FillColor>453017600</FillColor>

<FillMaterial>null</FillMaterial>

</Oval>

</Shapes>

</ActiveObjectClass>

</ActiveObjectClasses>

<DifferentialEquationsMethod>EULER</DifferentialEquationsMethod>

<MixedEquationsMethod>RK45_NEWTON</MixedEquationsMethod>

<AlgebraicEquationsMethod>MODIFIED_NEWTON</AlgebraicEquationsMethod>

<AbsoluteAccuracy>1.0E-5</AbsoluteAccuracy>

<FixedTimeStep>0.0010</FixedTimeStep>

<RelativeAccuracy>1.0E-5</RelativeAccuracy>

<TimeAccuracy>1.0E-5</TimeAccuracy>

<Experiments>

<!-- ========= Simulation Experiment ======== -->

<SimulationExperiment ActiveObjectClassId="1390440632827">

<Id>1390440632837</Id>

<Name><![CDATA[Simulation]]></Name>

<ClientAreaTopLeft><X>0</X><Y>0</Y></ClientAreaTopLeft>

<PresentationTopGroupPersistent>true</PresentationTopGroupPersistent>

<IconTopGroupPersistent>true</IconTopGroupPersistent>

<Frame>

<X>0</X>

<Y>0</Y>

<Width>1200</Width>

<Height>1070</Height>

<Maximized>true</Maximized>

<CloseConfirmation>false</CloseConfirmation>

</Frame>

<CommandLineArguments><![CDATA[]]></CommandLineArguments>

<MaximumMemory>2048</MaximumMemory>

<RandomNumberGenerationType>fixedSeed</RandomNumberGenerationType>

<CustomGeneratorCode>new Random()</CustomGeneratorCode>

<SeedValue>738721</SeedValue>

<SelectionModeForSimultaneousEvents>LIFO</SelectionModeForSimultaneousEvents>

<VmArgs><![CDATA[]]></VmArgs>

<LoadRootFromSnapshot>false</LoadRootFromSnapshot>

<SnapshotFile></SnapshotFile>

<Variables>

<Variable Class="PlainVariable">

<Id>1391187479022</Id>

<Name><![CDATA[popSize]]></Name>

<X>-150</X><Y>50</Y>

<Label><X>10</X><Y>0</Y></Label>

<PublicFlag>false</PublicFlag>

<PresentationFlag>true</PresentationFlag>

<ShowLabel>true</ShowLabel>

<Properties SaveInSnapshot="true" Constant="false" AccessType="public" StaticVariable="false">

<Type><![CDATA[int]]></Type>

<InitialValue><![CDATA[1000]]></InitialValue>

</Properties>

</Variable>

<Variable Class="PlainVariable">

<Id>1391626744072</Id>

<Name><![CDATA[speed]]></Name>

<X>-150</X><Y>80</Y>

<Label><X>10</X><Y>0</Y></Label>

<PublicFlag>false</PublicFlag>

<PresentationFlag>true</PresentationFlag>

<ShowLabel>true</ShowLabel>

<Properties SaveInSnapshot="true" Constant="false" AccessType="public" StaticVariable="false">

<Type><![CDATA[int]]></Type>

<InitialValue><![CDATA[4]]></InitialValue>

</Properties>

</Variable>

<Variable Class="PlainVariable">

<Id>1412535434905</Id>

<Name><![CDATA[twoWeek]]></Name>

<X>-150</X><Y>130</Y>

<Label><X>10</X><Y>0</Y></Label>

<PublicFlag>false</PublicFlag>

<PresentationFlag>true</PresentationFlag>

<ShowLabel>true</ShowLabel>

<Properties SaveInSnapshot="true" Constant="false" AccessType="public" StaticVariable="false">

<Type><![CDATA[boolean]]></Type>

</Properties>

</Variable>

<Variable Class="PlainVariable">

<Id>1412535454941</Id>

<Name><![CDATA[vouchers]]></Name>

<X>-150</X><Y>240</Y>

<Label><X>10</X><Y>0</Y></Label>

<PublicFlag>false</PublicFlag>
[truncated: 86,173 more chars]
